# Small Molecule Amiloride Modulates Oncogenic RNA Alternative Splicing to Devitalize Human Cancer Cells

**DOI:** 10.1371/journal.pone.0018643

**Published:** 2011-06-09

**Authors:** Jan-Gowth Chang, Den-Mei Yang, Wen-Hsin Chang, Lu-Ping Chow, Wen-Ling Chan, Hui-Hua Lin, Hsien-Da Huang, Ya-Sian Chang, Cheng-Hao Hung, Wen-Kuang Yang

**Affiliations:** 1 Department of Medical Research, University Hospital, Kaohsiung Medical University, Kaohsiung, Taiwan; 2 Graduate Institute of Clinical Medicine, Kaohsiung Medical University, Kaohsiung, Taiwan; 3 Graduate Institute of Medicine, College of Medicine, Kaohsiung Medical University, Kaohsiung, Taiwan; 4 Cell/Gene Therapy Research Laboratory, Department of Medical Research, China Medical University Hospital, Taichung, Taiwan; 5 Graduate Institute of Biochemistry and Molecular Biology, College of Medicine, National Taiwan University, Taipei, Taiwan; 6 Institute of Bioinformatics and Systems Biology, National Chiao Tung University, Hsin-Chu, Taiwan; 7 Department of Biological Science and Technology, National Chiao Tung University, Hsin-Chu, Taiwan; 8 Departments of Biochemistry and Medicine, China Medical University, Taichung, Taiwan; 9 Cancer Center, Kaohsiung Medical University Hospital, Kaohsiung, Taiwan; Univesity of Texas Southwestern Medical Center at Dallas, United States of America

## Abstract

Alternative splicing involves differential exon selection of a gene transcript to generate mRNA and protein isoforms with structural and functional diversity. Abnormal alternative splicing has been shown to be associated with malignant phenotypes of cancer cells, such as chemo-resistance and invasive activity. Screening small molecules and drugs for modulating RNA splicing in human hepatocellular carcinoma cell line Huh-7, we discovered that amiloride, distinct from four pH-affecting amiloride analogues, could “normalize” the splicing of *BCL-X, HIPK3* and *RON/MISTR1* transcripts. Our proteomic analyses of amiloride-treated cells detected hypo-phosphorylation of splicing factor SF2/ASF, and decreased levels of SRp20 and two un-identified SR proteins. We further observed decreased phosphorylation of AKT, ERK1/2 and PP1, and increased phosphorylation of p38 and JNK, suggesting that amiloride treatment down-regulates kinases and up-regulates phosphatases in the signal pathways known to affect splicing factor protein phosphorylation. These amiloride effects of “normalized” oncogenic RNA splicing and splicing factor hypo-phosphorylation were both abrogated by pre-treatment with a PP1 inhibitor. Global exon array of amiloride-treated Huh-7 cells detected splicing pattern changes involving 584 exons in 551 gene transcripts, many of which encode proteins playing key roles in ion transport, cellular matrix formation, cytoskeleton remodeling, and genome maintenance. Cellular functional analyses revealed subsequent invasion and migration defects, cell cycle disruption, cytokinesis impairment, and lethal DNA degradation in amiloride-treated Huh-7 cells. Other human solid tumor and leukemic cells, but not a few normal cells, showed similar amiloride-altered RNA splicing with devitalized consequence. This study thus provides mechanistic underpinnings for exploiting small molecule modulation of RNA splicing for cancer therapeutics.

## Introduction

Proteome complexity is expanded by alternative splicing (AS), a process involving differential exon inclusion or exclusion of the same pre-mRNA molecules to produce various mRNA and protein isoforms [1], [2]. The AS process is controlled by two highly conserved protein families: arginine/serine dipeptide repeats (SR)-related proteins and heterogenous nuclear ribonucleoproteins (hnRNP)-related proteins [3], [4]. SR proteins contain a modular bipartite structure including one or two RNA recognition motifs at the N terminus and a RS domain rich in arginine/serine dipeptide repeats at the C-terminus [5], [6]. The function of the N-terminal motifs in SR proteins involves sequence-specific binding to exonic splicing exhancers. The C-terminal RS domain serves as a general activation domain for splicing by linking to a heterogeneous RNA binding motif [6]. SR proteins regulate the selection of AS sites, which diverge from canonical splice sites, therefore promoting inclusion or exclusion of alternative exons. On the other hand, some hnRNPs, such as hnRNPA1, may antagonize the AS function of SR proteins through binding to exonic splicing silencer elements [7].

AS therefore regulates gene expression by generating discrete protein isoforms involved in distinct functions of cellular events, such as apoptosis, sex determination, axon guidance, cell excitation, and cell contraction [8], [9]. AS also plays a pivotal role in the development of human hereditary disorders and cancer [10], [11], [12], [13], [14]. Aberrant AS of proto-oncogenes and/or tumor suppressor genes may contribute to cellular malignant phenotypes, such as resistance to apoptosis, promotion of invasion and metastasis, and stimulation of tumor angiogenesis [11], [12], [13], [14]. Postulating that modulating these cancer-related genes could have profound impact on their pathogenic activities, we have been searching for small molecules or drugs with modulating effects on AS. In this study, we have found that amiloride, a well-known diuretic, can alter oncogenic AS in human hepatocellular carcinoma Huh-7 cells as well as in several other human cancer and leukemic cell lines, and hence explore the underlying molecular and cellular mechanisms for possible therapeutic implications.

## Results

### Amiloride “normalizes” the isoform RNA splicing of *BCL-X, HIPK3* and *RON/MISTR1* in Huh-7 cells

**Figure 1:**
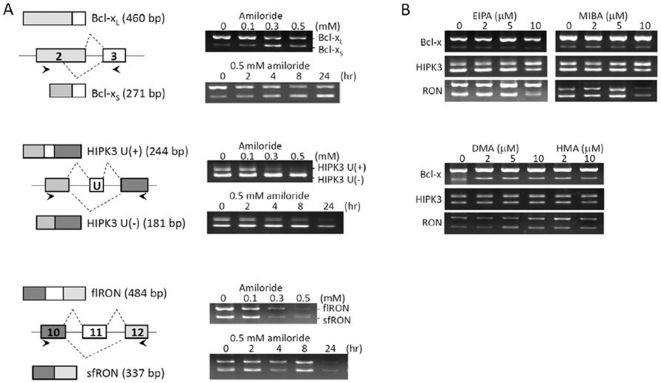
Modification of splicing patterns of *BCL-X, HIPK3* and *RON* gene transcripts by amiloride. RNA was extracted from Huh-7 cells exposed to growth medium containing amiloride or other pHi modulator amiloride derivatives and examined for the oncogenic RNA alternative splicing by RT-PCR, as described in Materials and Methods. **Panel (A)** shows that amiloride increased the use of upstream alternative 5′-splice site within exon 2 that yields the apoptotic BCL-XS isoform (upper row), increased the exon 11 (U exon) skipping of HIPK3 that increases the apoptotic isoform (middle row), and slightly increased the exon 11 skipping of RON at 0.5 mM concentration (lower row). **Panel (B)** shows that splicing patterns of the tested genes were not significantly affected by four amiloride derivatives, including 5-(N-ethyl-N-isopropyl)-amiloride or EIPA; 5-(N-methyl-N-isobutyl)amiloride or MIBA; 5-(N,N-dimethyl)amiloride or DMA; and 5-(N,N-hexamethylene)amiloride or HMA, at equivalent concentrations of pHi modulation.

It has recently become clear that unbalanced RNA splicing of certain genes is associated with malignant properties of human cancer cells [11], [12], [13], e.g., resistance to chemotherapy and radiotherapy with *BCL-X,* onco-developmentally undifferentiated state with *HIPK3,* and invasive metastatic potentials with *RON/MISTR1*. In hepatocellular carcinoma Huh-7 cells, *BCL-X* is alternatively spliced into anti-apoptotic large RNA isoform, *BCL-XL,* more than pro-apoptotic small RNA isoform, *BCL-XS* [15]; *HIPK3* is spliced into exon 11-excluded U (-) as well as exon 11-included U (+) isoforms for interaction with Fas/FADD to modulate apoptosis in the developmental process [16]; and *RON/MISTR1* is spliced into 5-kb full-length (*flRON*) RNA and 2-kb exon 11-excluded *RON* (*sfRON*) RNA isoforms, the latter of which has been correlated with epithelial to mesenchymal transition in cancer cell invasion and metastasis [17], [18]. After screening more than 500 small molecules and drugs, including those in clinical use, we found that amiloride exerted a potent effect on AS of these gene transcripts (Figure 1A). Huh-7 cells treated with amiloride showed: (a) relative decrease of *BCL-XL* and increase of *BCL-XS* RNA isoforms; (b) relative maintenance of *HIPK3* U (-) and decrease of *HIPK3* U (+) isoforms; and (c) decrease of both exon 11 (+) *flRON* and exon 11 (-) *sfRON* RNA isoforms. Such effects of amiloride were dose- and time-dependent, being detectable by 2 hours and marked after 24 hours of incubation with 0.5 mM of amiloride. These observations suggested increased utilization of the upstream alternative 5′-splice site within exon 2 of *BCL-X*, increased exon 11 (U) exclusion of *HIPK3*, and decreased splicing complexes formed for production of both *RON/MISTR1* exon 11 (+) and exon 11 (-) isoforms. Thus, amiloride could modulate the AS of these three gene transcripts towards less malignant “normalized” patterns, similar to the normal ones in previous reports [15], [16], [17], [18].

Because amiloride is a prototype intracellular pH (pHi) modulator drug, we examined four amiloride derivatives at equivalent or greater pHi-affecting doses. We observed that these derivatives induced no similar “normalizing” effects on *BCL-X*, *HIPK3,* and *RON/MISTR1* AS patterns in Huh-7 cells (Figure 1B). By contrast, in a previous study [19] we observed that 5-(N-ehyl-N-isopropyl) amiloride (EIPA) but not amiloride modulated AS by decreasing the pathogenic exon7 exclusion of *SMN2* transcripts of hereditary spinal muscular atrophy cells. These paradoxical observations demonstrate the complexities of AS mechanisms and also suggest that the splicing site selection in *BCL-X*, *HIPK3,* and *RON/MISTR1* transcripts in amiloride-treated Huh-7 cells is mediated through cell-specific splicing mechanism(s) rather than simply intracellular pH change.

### Proteomic detection of mostly hypo-phosphorylated splicing factor SF2/ASF in the cytoplasm and nucleus of amiloride-treated cells

**Figure 2:**
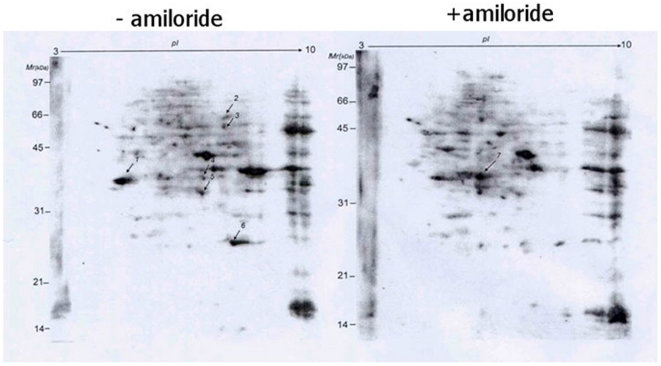
Amiloride effects on cellular phospho-serine proteins. 2D-gel electrophoresis analysis of proteins extracted from Huh-7 cells without (left) and with (right) 0.5mM amiloride treatment for 24 hours showed significant changes for at least ten spots, of which seven (indicated by arrows) were isolated for nano-LC-MS/MS spectrometry analysis and protein identification, as shown in Table 1.

**Figure 3:**
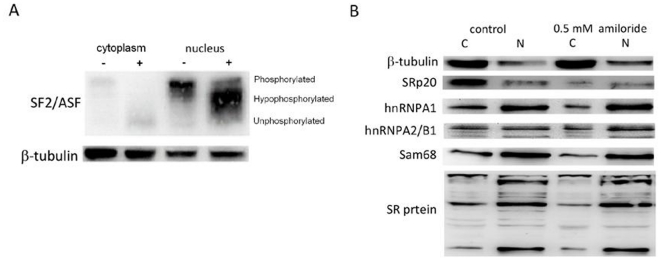
Effects of amiloride treatment on phosphorylation of SR-related proteins. Western blot analysis of subcellular fractions of Huh-7 cells with and without 0.5 mM amiloride 24-hour treatment was performed using specific antibodies against various splicing protein factors and β-tubulin. (**Panel A**) The amiloride treatment increased the dephosphorylated SF2/ASF forms in both cytoplasmic and nuclear fractions. (**Panel B**) The amiloride treatment decreased the expression of SRp20 and two un-identified phosphorylated SR proteins in both C (cytoplasmic) and N (nuclear) cellular fractions.

As phosphorylation status of SR protein components in the AS complexes is known to affect protein-protein interaction for the splicing function, we next used 2D-gel electrophoresis to analyze differential expression of serine-phosphorylated proteins. We found more than ten proteins with quantitative changes after amiloride treatment (Figure 2). Choosing seven of these protein spots for proteomic identification by mass spectrometry (Table 1), we found one of them to be ASF/SF2, a splicing factor known to be involved in AS of the *RON* transcript [20]. To verify this finding, we performed western blots of ASF/SF2 and found markedly reduced ASF/SF2 phosphorylation in both the cytoplasm and nucleus of amiloride-treated Huh-7 cells (Figure 3A). Analysis of other protein constituents of the splicing complexes (such as SRp20, hnRNPA1, hnRNPA2/B1, Sam68 and common SR proteins) showed that SRp20 and two un-identified phosphorylated SR proteins were also reduced in the cytoplasm and nucleus after amiloride treatment (Figure 3B). These results imply that amiloride modulates the AS of *BCL-X*, *HIPK3,* and *RON/MISTR1* through hypo-phosphorylation of ASF/SF2 and decreased expression of SRp20 and some other SR proteins.

**Table 1:** Proteomic identification of 7 selected phosphoryl-serine proteins down- or up-regulated in amiloride-treated Huh-7 cells.

Spot no.	Swissport accession no.	Protein name	Mascot score	Theoretical PI/Mr(Da)	Sequence coverage	Unique peptides	Direction of regulation
1	07955	Splicing factor, arginine/serine-rich 1	302	5.6/31,848	26%	^17^YVGNLPPDIR^27^ ^26^TKDIEDVFYK^37^ ^51^GGPPFAFVEFEDPR^64^ ^74^DGYDYDGYR^82^ ^83^LRVEFPR^89^ ^122^VVVSGLPPSGSWQDLK^137^ ^154^DGTGVVEFVR^163^	Down
2	P25705	ATP synthase alpha chain, mitochondrial precursor	216	9.1/59,174	13%	^46^TGTAEMSSILEER^58^ ^50^ILGADTSVDLEETGR^73^ ^74^VLSIGDGIAR^83^ ^134^TGAIVDVPVGEELLGR^149^ ^150^VVDALGNAIDGK^161^ ^195^AVDSLVPIGR^204^	Down
3	P25705	ATP synthase alpha chain, mitochondrial precursor	763	9.1/59,174	39%	^45^TGTAEMSSILEER^58^ ^59^ILGADTSVDLEETGR^73^ ^74^VLSIGDGIAR^83^ ^89^NVQAEEMVEFSSGLK^103^ ^104^GMSLNLEPDNVGVVVFGNDK^123^ ^133^RTGAIVDVPVGEELLGR^149^ ^150^VVDALGNAIDGKGPIGSK^167^ ^183^ISVREPMQTGIK^194^ ^195^AVDSLVPIGR^204^ ^208^ELIIGDR^214^ ^219^TSIAIDTIINQK^230^ ^254^STVAQLVK^261^ ^306^HALIIYDDLSK^316^ ^323^QMSLLLR^329^ ^335^EAYPGDVFYLHSR^347^ ^403^GIRPAINVGLSVSR^416^ ^427^QVAGTMKLELAQYR^441^	Down
4	P70355	Annexin A2	627	7.5/38,449	39%	^10^LSLEGDHSTPPSAYGSVK^27^ ^25^AYTNFDAER^36^ ^37^DALNIETAIK^46^ ^49^GVDEVTIVNILTNR^62^ ^68^QDIAFAYQR^76^ ^104^TPAQYDASELK^114^ ^152^TDLEKDIISDTSGDFR^167^ ^178^RAEDGSVIDYELIDQDAR^196^ ^198^DLYDAGVK^203^ ^313^SLYYYIQQDTK^323^	Down
5	P32322	Pyrroline-5-carboxylate reductase 1	477	7.1/33,340	47%	^30^IMASSPDMDLAITVSALRK^47^ ^108^LSAFRPAPR^118^ ^120^CMTNTPVVVR^129^ ^130^EGATVYATGTHAQVEDGR^147^ ^205^LGAQALLGAAK^215^ ^216^MLLHSEQHPGQLK^228^ ^229^DNVSSPGGATIHALHVLESGGFR^251^ ^252^SLLINAVEASCIR^264^ ^267^ELQSMADQEQVSPAAIKK^284^ ^290^VKLDSPAGTALSPSGHTK^307^	Down
6	Q06830	Peroxiredoxin-1	266	8.2/22,096	41%	^17^ATAVMPDGQFK^27^ ^28^DISLSDYK^35^ ^94^QGGLGPMNIPLVSDPK^109^ ^111^TIAQDYGVLK^120^ ^121^ADEGISFR^128^ ^129^GLFIIDDK^136^ ^141^QITVNDLPVGR^151^ ^159^LVQAFQFTDK^168^	Down
7	P19623	Spermidine synthase	176	5.3/33,803	17%	^48^YQDILVFR55 ^97^VLIIGGGDGGVLR^109^ ^136^FLPGMAIGYSSSK^148^ ^187^ESYYQLMK^194^ ^286^AAFVLPEFAR^295^	Up

### Amiloride down-regulates phosphorylation of AKT kinase and PP1 phosphatase

**Figure 4:**
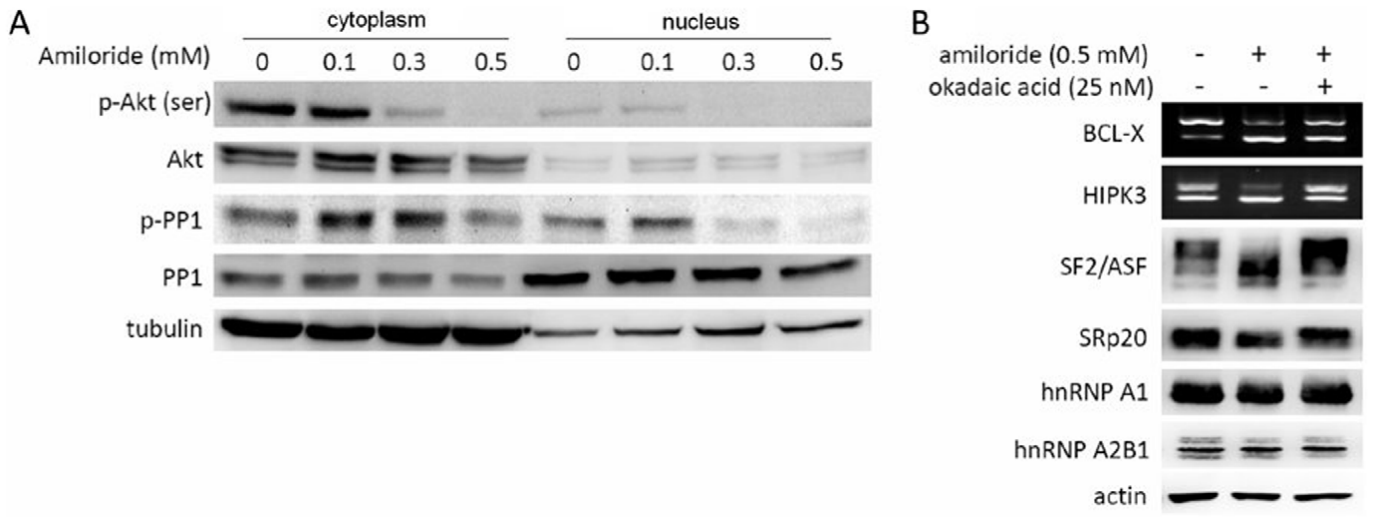
Phosphorylation of Akt kinase (activation) and PP1 phosphatase (inactivation) in relation to amiloride-altered AS mechanisms. (**Panel A**). Western blots show significant decreases of cytoplasmic p-Akt, nuclear p-Akt and nuclear p-PP1 in Huh-7 cells treated with amiloride at 0.3 mM and above. (**Panel B**) RT-PCR and western blot analyses show that pre-treatment with a PP1 inhibitor, okadaic acid, abrogated the amiloride effects on the BCL-X and HIPK3 oncogenic RNA splicing, the phosphorylation of ASF2/ASF splicing factor and the level of SRp20 in Huh-7 cells.

Recent studies have shown that amiloride inhibits phosphorylation of kinases and phosphatases associated with the PI3K-AKT pathway [21], [22], [23], and that AKT kinase and PP1 phosphatase may regulate the activity of SR proteins [23], [24], [25], [26], [27], [28]. We therefore determined the effects of amiloride on the phosphorylated forms of Akt kinase and related phosphatases in Huh-7 cells. Decreased levels of the Ser(473) p-AKT and the Thr(320) p-PP1 were observed in the cytoplasm and nucleus, suggesting inhibition of AKT kinase activity and activation of PP1 phosphatase activity (Figure 4A). To determine whether inactivation of Akt kinase per se would play a role in regulating the alternative splicing, Huh7 cells were treated with PI3K-AKT pathway inhibitors, including LY294002, wortmannin and triciribine; 1 µM triciribine but not LY294002 or wortmannin decreased the level of p-Akt. However, triciribine at this concentration exerted no effect on the alternative splicing of *BCL-X* and *HIPK3* transcripts (Figure S1). Interestingly, pre-treatment of the cells with okadaic acid to inhibit PP1 phosphatase activity abrogated the effects of amiloride on *BCL-X* and *HIPK3* splicing, as well as effects of amiloride on the dephosphorylation of SF2/ASF and decreasing of the SRp20 level, but okadaic acid exerted no definite effect on the expression of hnRNP A1 and hnRNP A2B1 (Figure 4B). Taken together, these results suggest that PP1 plays an important role in modulating phosporylation of SR splicing factors and hence the modulation of oncogenic AS in Huh-7 cells after amiloride treatment.

### Genome-wide exon-array detection of altered RNA splicing of many functional genes in amiloride-treated cells

**Figure 5:**
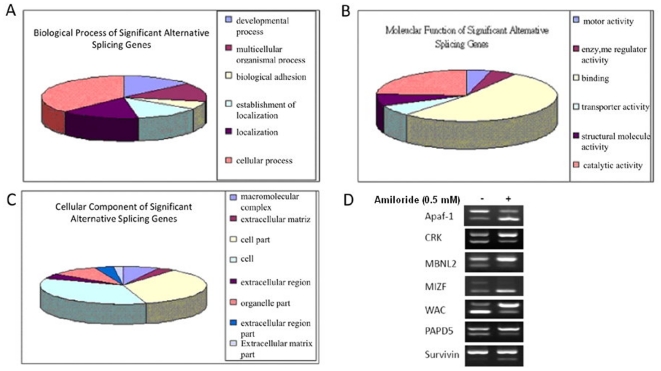
Gene ontology distribution of 495 protein-encoding gene transcripts with amiloride-modulated AS patterns in Huh-7 cells. Bioinformatic analyses of the 495 gene transcripts with amiloride-modified alternative splicing, detected by the genome-wide exon-array measurements, were performed according to gene ontology categories of Biological Process (**A**), Molecular Function (**B**) and Cellular Component (**C**). Amiloride-altered AS patterns of *APAF1, CRK,MBNL2, MIZF,WAC, PAPD5* and *SU*R*VIVIN* specific protein-encoding gene transcripts detected by the exon-array analysis were validated by RT-PCR of spliced RNA isoforms (**Panel D**).

It is possible that the effects of amiloride on the phosphorylation and intracellular localization of splicing factors and mRNA export [29] could affect splicing of other gene transcripts in addition to *BCL-X, HIPK3* and *RON/MISTR1*. Using gene array chips (Affymetrix GeneChip® Human Exon 1.0 ST Array of >518000 exons of 42974 genes) for exon array analysis (set parameters of correlation coefficient ≥0.7, splicing index ≤−1.585 , and log_2_ ratio ≤−1.585), we found that amiloride influenced the splicing patterns of 551 genes involving at least 584 exons, which included 495 known protein-coding genes involving 526 exons (Table S1; MIAME accession number #GSE24581). These 495 known protein-coding genes could be classified into functional categories involving cytoskeleton remodeling, cell adhesion, ion transport, transcription factors, immune response, and muscle contraction (Figure 5A-C and Table S2). To verify these exon array results, we selected seven (*APF-1, CRK, MBNL2, MIZF, WAC, PAPDS* and *survivin*) for analysis by RT-PCR and indeed confirmed distinct AS pattern alterations of these gene transcripts in amiloride-treated cells (Figure 5D).

At detailed gene ontology (GO) levels, the RNA transcripts showing amiloride-affected AS included:**a** **).** 4 of 5 genes (80.0%) of “microfilament motor activity” (GO 0000146), and 28/132 (21.2%) of genes of “motor activity” (GO 0003774); **b)**. 5 of 7 (71.4%) of “cation:chloride symporter activity” (GO 0015377), 5/18 (27.8%) of “anion-cation symporter” (GO 0015296), 3/10 (30.0% ) of “sodium potassium exchanging ATPase” (GO 0005391), and 2/8 (25.0%) of inorganic anion exchange activity” (GO 0005452) ; **c)**. 2 of 3 (66.7%) nitric- oxide synthase activity (GO 0004517); **d)** 16/29 genes (62.1%) of “extracellular matrix structural constituent conferring tensile strength” (GO 0030020), 38/346 genes (11.0%) of “cytoskeletal protein binding” (GO 0030020), 2/7 (28.6%) of collagen binding (GO 0005518); 2/8 (25.0%) of “myosin binding” (GO 0017022), and others. Of 213 apoptosis-related genes, 6 (*CCAR1, EP3000, KIAA1967, PRKDC, and PRPF8*) showed altered alternative splicing by amiloride. Of 608 cancer-related genes, 32 including *RON/MISTR1, ATM* (ataxia teleangiectasis mutated) and *FANCM* (Fanconi anemia M protein) were affected by amiloride. As *ATM* plays an important role in DNA damage repair while *FANCM* is involved in the assembly of Fanc protein complexes that maintain genome integrity and stability [30], altered AS of these two gene transcripts by amiloride may result in chromatin degradation, as verified in our subsequent experiments. However, *RON/MISTR1*, but not *BCL-X* and *HIPK3*, was among the 495 genes detected to have amiloride-altered splicing patterns by exon array analysis, presumably because of the stringent quantitative criteria set, and/or short spliced sequences of *BCL-X*.

Using known consensus nucleotide sequences of splicing regulatory elements, we further identified possible splicing enhancer and silencer elements in the alternative splicing exon, 5′-flanking intron and 3′-flanking intron regions of the 495 genes affected by amiloride (Table S3). These splicing cis-elements are the binding sites of ASF/SF2, SRp55 and other unknown regulatory factors. During interactions of SF2/ASF and other SR protein factors with cis-elements in the splicing complexes, the functional state of protein-protein binding complexes may be modulated by the phosphorylation status of the proteins [21], [23], [31], [32]. This implies that the altered splicing of these gene transcripts was mediated through amiloride-induced hypo-phosphorylation of SF2/ASF and other SR proteins.

### Decreased cell mobility/invasion activities and depleted cytoskeletal structures following AS changes of involved genes after amiloride exposure

**Figure 6:**
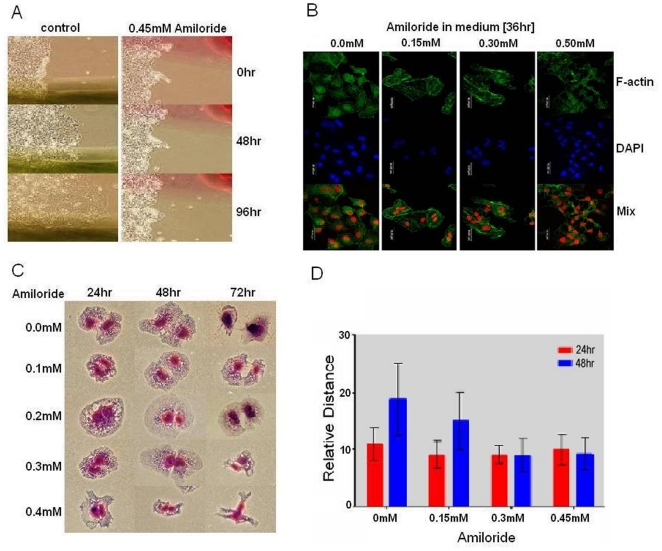
Cell migration and post-mitotic cytokinesis disorders subsequent to amiloride-altered AS of related functional gene transcripts. (**Panel A**). Cell migration was determined in confluent Huh-7 cell monolayers scraped with a plastic scraper, exposed to growth medium with or without 0.45 mM amiloride for 48 hours, rinsed 2x with PBS and onward fed growth medium without amiloride. Serial microscopic photographs of same areas (marked green or red on the bottom side of dishes) taken at 48 and 96 hours show complete inhibition of cell migration from the edge of amiloride-treated monolayer. (**Panel B**) Cells exposed to the indicated concentrations of amiloride in growth medium for 36 hours were fixed with paraformaldehyde, permealized and stained with DAPI and FITC-phalloidin for observation by confocal microscopy. DAPI-stained nuclei are magenta-pseudocolored in merged confocal photographs. (**Panel C**) Inhibitory effect of amiloride on cytokinesis was observed with mitotic Huh-7 cells shaken off from the dish, re-plated in growth medium containing different concentrations of amiloride. The dividing daughter cells were fixed at 24, 48 and 72 hours, stained and photographed microscopically. (**Panel D**) Measurement of distances between the two nuclei of dividing or divided daughter cells shows that amiloride inhibits post-mitotic cytokinesis and separation of daughter cells. The diameter of the cell nucleus is set as 10. The average and standard deviation of each sample point are from 15 to 20 daughter cell pairs.

Because altered *RON/MISTR1* splicing mediated by SF2/ASF has been shown to result in increased mobility and invasive activities of cells [20], we examined the functional impact of amiloride-induced decrease of exon 11 (+) fl*RON* and exon 11 (-) sf*RON* isoforms (Figure 1C) and decrease of exon 13 and exon 20 inclusion detected by exon array analysis (Table S1). Migration of cells from a scraped monolayer edge was impaired by 0.4 mM amiloride (Figure 6A). Fluorescent phalloidin binding demonstrated alteration of F-actin cytoskeletal scaffolds from a peri-nuclear network structure in control cells to a cell-surface distribution with an apparently rigid enclosure of multiple nuclei in amiloride-treated cells, and also severe depletion of cytoplasmic contents, including F-actin, after exposure to 0.4 mM or higher concentrations of amiloride (Figure 6B). These observations of impaired cell mobility and abnormal cytoplasmic organization of F-actin are consistent with our exon array findings of altered RNA splicing of many key interacting protein components of the extracellular matrix, including collagen families for cytoskeletal networks as well as myosin families and their binding proteins (Table S1), in addition to *RON/MSTIR* and associated regulatory molecules.

### Perturbation of cell cycle progression and mitotic cytokinesis by amiloride

**Figure 7:**
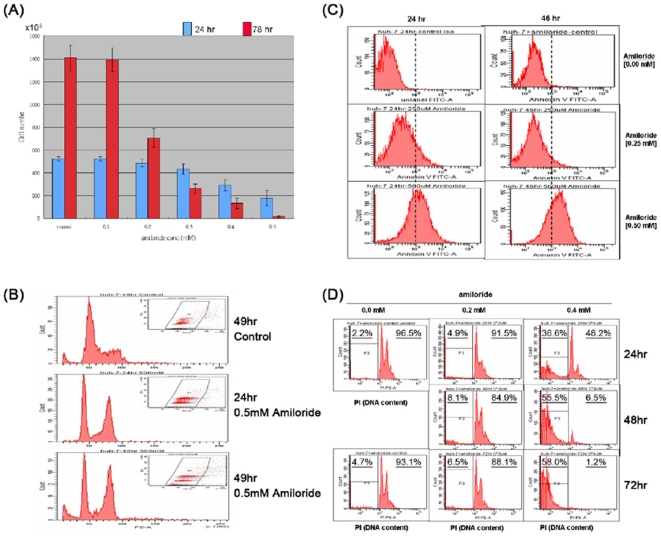
Growth inhibitory and apoptotic/cytotoxic consequences of amiloride-induced AS alterations. (**Panel A**) Huh-7 cells plated in 6-well dishes at 3×10^5^ cells per well overnight were changed to growth medium containing various concentrations of amiloride; cell numbers were determined one and three days later. Cell counts at 24 hours show growth inhibition by amiloride at 0.4 mM and above. Further cell count decreases were evident at 72 hours due to cell death and detachment in medium containing amiloride at 0.3 mM and above. (**Panel B**) Cytofluorometric analysis shows amiloride inhibition of cell cycle progression with accumulation of G2/M phase cells. (**Panel C**) Apoptosis is evident by cytofluorometric detection of annexinV binding in Huh-7 cells treated with 0.5 mM of amiloride. (**Panel D**) Marked nuclear DNA degradation was observed in Huh-7 cells treated with 0.4 mM amiloride but not with 0.2 mM amiloride, particularly noted at 48 and 72 hours.

Our exon array analysis of amiloride-treated Huh-7 cells (Tables S1 & S2) detected alterations of RNA splicing predominantly of proteins involved in cytokinesis, associated with solute transport, and functions of actin, microtubules, and cytokinesis-related kinases [33]. In addition, the altered RNA splicing of *CENPE* (centromere protein E 312kDa), *CEP250* (centrosomal protein 250kDa) and *CEP192* (centrosomal protein 192kDa) would be expected to result in inefficient chromatid separation during the cell cycle. Indeed, proliferation of Huh-7 cells was inhibited by 0.3 mM amiloride or higher in the growth medium (Figure 7A). Furthermore, there was an accumulation of G2/M tetraploid cells beginning at 24 hours, becoming marked at 48 hours (Figure 7B). By light microscopic examination, there were many bi-nucleated and late-stage anaphase cells that were presumably the G2/M tetraploid cell population observed by fluorocytometry. During cell cycle progression and daughter cell separation in the presence of varying concentrations of amiloride, mitotic cells exposed to amiloride at 0.3 mM or higher developed into bi-nucleated forms at 24 hours but subsequently failed to generate separated daughter cells (Figure 6C and 6D). These three observations imply that through altering the RNA AS of genes, amiloride causes disturbance of cytokinesis by inhibiting mitotic cells from passing through late anaphase processes of abscission, division, and separation of daughter cells. Finally, we confirmed a previous report that Huh-7 cells express Prominin/AC133/CD133, an immunological marker of the “cancer stem-like” cells [34], and further found that amiloride at 0.3 mM or more down-regulated CD133 expression Huh-7 cells (Figure S2-A), resulting in suppression of the number and size of cell colony formations (Figure S2-B).

### Cell death with severe DNA degradation subsequent to amiloride-induced altered splicing of functional molecules in apoptosis and DNA repair pathways

In amiloride-treated cells, splicing changes of *BCL-X* and *HIPK3* RNAs (Figure 1A) would predict altered cellular apoptosis mechanisms, and altered AS of *ATM* and *FANCM* RNAs (Table S1) would alter DNA repair and cellular genome protection mechanisms. Huh-7 cells culture in the presence of 0.3 to 0.5 mM amiloride demonstrated not only lack of cell growth but also subsequent loss of initially plated Huh-7 cells (Figure 7A). Apoptosis was evident by increased percentages of annexinV binding cells after exposure to 0.3–0.5 mM amiloride for 24 or 46 hours (Figure 7C). Furthermore, fluorocytometric analysis of cellular DNA showed extensive nuclear DNA fragmentation and degradation in more cells than those demonstrated to be undergoing apoptosis; after exposure to 0.4 mM amiloride, only 48.2%, 6.5%, and 1.2% of Huh-7 cells had intact cellular DNA profiles after 24, 48, and 72 hours respectively (Figure 7D).

### Similar modulating effects of amiloride on oncognic RNA splicing in other human solid tumor and leukemic cell lines

To determine the generality of amiloride effects observed in Huh-7 cells, we used additional 10 established human cancer and leukemia cell lines, including 2 hepatoma HepG2 and Hep3B; rhabdomyosarcoma TE671; 3 glioblastoma multiforme 8401, ASCG13T1xm and ASCG7T(4); 2 colon cancer invasive cell lines HT29 and COH; and 3 leukemias K562, HL60 and Molt4 cells, and a normal bone marrow-derived mesenchymal stromal line KP-hMSC as well as a few normal blood lymphocyte cultures, to perform various fore-mentioned experiments. Our results showed that in general amiloride tended to affect the malignant phenotype-associated AS more than the normal AS in the cell. First, essentially as in Huh-7 cells (Figure 1), we found amiloride-modulated “normalization” of the abornal *BCL-X* and *HIPK3* splicing patterns in hepatoma Hep G2, rhabdomyosarcoma TE671, glioblastoma 8401, leukemias K562, HL60 and Molt4 (e.g. supplementary data of [35]). Most significantly, amiloride could modulate the transcription/splicing of *BCR-ABL* fusion gene and re-sensitize the chemoresistant *Bcr-Abl*T3151 mutant cells to imatinib [35]. On the contrary, using amiloride up to 2 mM in the growth medium, we detected no alteration in the *BCL-X* and *HIPK3* splicing patterns in cultured activated normal peripheral blood mononuclear cells (supplementary data of [35]) nor in immortalized bone marrow stromal KP-hMSC cells. Consistently, amiloride in the range of 0.02 mM-0.5 mM was cytotoxic to cancer cells but not to normal cells in culture (Figure S3 A & B). Using a 72-hour *in vitro* culture assay, we found that the 50% growth inhibiting doses of amiloride were approximately 0.02 mM for TE671, 0.10 mM for K562, 0.20 mM for Huh-7, 0.3 mM for two other HCC Hep-G2 and Hep-3B, and 0.2–0.4 mM for the three glioblastoma multiforme lines but >3 mM or unmeasurable for the normal cell lines (Figure. S3-B). Further, we performed global exon array analysis on myeloid leukemia blasts K562 and glioblastoma 8401, with and without amiloride treatment, for comparison with Huh-7 (Tables S1, S2 and S3). Cross-matching of the 200 top-scored amiloride-modulated genes of each of the three cell lines revealed a rather common feature of amiloride-modulated splicing, namely that 187 genes or 93.5% of these 200 were the same for the three cells; 188 (187 plus *DNAH8*) for Huh-7 and 8401; 189 (187 plus *LRP2/RYR2*) for Huh-7 and K562; and 190 (187 plus *ABL1, ColSA2, NAALAD2*) for 8401 and A549 (Figure S4-A). The GO analysis of the 187 common genes according to the biological processes (Figure S4-B), molecular functions (Figure S4-C) and cell components (Figure S4-D) categories showed percentage distribution similar to the amiloride-modulated 495 genes of Huh-7 cells (Figure 5), implying that, in K562 and 8401 cells as in Huh-7 cells, amiloride-modulated AS causes similar effects on the cytoskeletal structure, cytokinesis, and DNA repair, as well as on the apoptosis mechanism and the solute transport system.

## Discussion

This study is based on our initial finding that amiloride changed the alternative RNA splicing of *BCL-X, HIPK3* and *RON/MISTR1* transcripts from oncogenic patterns towards normal patterns in human hepatocellular carcinoma Huh-7 cells. In follow-up experiments, we observed hypo-phosphorylation of SR splicing factors, especially SF2/ASF, as well as in upstream protein kinases and phosphatases, suggesting that amiloride may affect the functional state of exon inclusion/exclusion complexes. Global exon analysis indeed revealed alterations of AS in many gene transcripts of cellular networks. As a consequence, amiloride-treated Huh-7 cells showed decreased migration/invasion cytokinetic capabilities, retarded cell cycle progression with mitotic defects, increased apoptotic signs, severe cellular DNA damages and ultimate cell death. Exploring subsequently, we have now obtained results that amiloride treatment can produce similar AS modulation and devitalizing effects on several other human malignant solid cancers and leukemic cell lines in addition to hepatocellular carcinoma Huh-7, but apparently not on a few cultured normal cells we tested (Figures S3 and S4).

### A distinct property of amiloride in modulating AS-mediated malignant phenotypes

Amiloride, 3,5-diamino-6-chloro-N-(diaminomethylene) pyrazinecarboxamide monohydrochloride, was developed in the 1960's [36] and has been a drug used clinically for treating edema and hypertension based on its sodium transport and fluid homeostasis effects [37]. Therefore, we initially thought that its effects on oncogenic RNA splicing would be mediated by changes in intracellular pH and ion movement. However, no similar AS alterations were detected in Huh-7 cells treated with four more potent pHi-affecting amiloride analogues, including EIPA, which we previously showed to modulate the pathogenic *SMN2* transcript in cells of spinal muscular atrophy patients. Thus, the ability to alter AS of oncogenic RNAs appears to be a distinct property of amiloride.

As AS is controlled by highly conserved SR family proteins [1], [3], [5] and plays an important role in the proteomic complexity of the cell, it is of interest to determine whether this property of amiloride is applicable to various other human malignant cancer types and abnormal cells with altered AS of various genes. In subsequent investigations, we have found that amiloride exerts very similar if not common effects on AS of cellular gene transcripts in the diverse context of malignant cell types examined. For example, in myelogenous blasts K562, amiloride not only “normalized” the abonormal *BCL-X* and *HIPK3* splicing but also modulated the alternate splicing of *BCR-ABL* transcript and re-sensitized the chemoresistant Bcr-AblT3151 mutant cells to imatinib [35].

### Mechanism of AS alterations by amiloride

By inference from a previous report [20], our observation of hypo-phosphorylation of SR protein splicing factors, particularly SF2/ASF and SRp20, may explain the modified alternative splicing of *RON/MSTIR* in amiloride-treated cells. SR proteins are nuclear phosphoproteins localized to nuclear speckles [6]. The RS domain of SR proteins determines the subcellular localization by acting as a nuclear localization signal in interaction with the transportin-SR, a nuclear import receptor for SR proteins [38]. The extensive serine phosphorylation of the RS domain is also important for regulating the activities and localization of SR proteins [39]. Protein kinases involved in the phosphorylation or dephosphorylation of SR-related proteins have been found to affect nuclear location of SR proteins and hence regulation of AS [39], [40]. Our finding of un-phosphorylated and hypo-phosphorylated ASF/SF2 in amiloride-treated cancer cells therefore implicates the inhibition of protein kinases or stimulation of protein phosphatases. It has been well documented that amiloride can inhibit the activities of tyrosine kinase, serine kinase and phosphatase through non-specific kinase inhibition [22], [24], [25], [26], [27], [28]. Amiloride-mediated inhibition of kinases can be relieved by their ATP substrate with kinetics of competitive inhibition [41]; however, the possibility of amiloride directly acting as an ATP substrate inhibitor in kinase reactions, thus causing SR protein hypo-phosphorylation, remains to be investigated. On the other hand, we have confirmed previous reports that amiloride can inhibit or alter the phosphorylation of key molecules in the PI3K-AKT pathway [22]. AKT has been reported to regulate the activity of SR proteins [24],[27]; and there are several consensus motifs for AKT phosphorylation in the RS domain of SR proteins. However, we were unable to alter the oncogenic RNA splicing of *BCL-X* and *HIPK3* by chemical inhibitors of the PI3K-AKT pathway (Figure S1). However, we found that pre-treatment with okadaic acid, a PP1 inhibitor, did abrogate the amiloride-altered splicing of *RON* and *HIPK3* transcripts as well as preventing the hypo-phosphorylation of ASF/SF2 and SRP20 SR proteins (Figure 4). We further found that amiloride decreased the phosphorylation of ERK 1 and 2, PPI phosphatase, but caused up-regulation of phosphorylation of P38 and JNK. It remains to be determined definitively whether or not and how these signaling pathways would mediate the amiloride effects in modulating RNA splicing processes by affecting protein phosphorylation of AS regulatory factors.

### Global proteo-genomic approaches to explore drug targeting of AS modifications

Regulatory mechanisms of AS have been most commonly explored by using cis-element-containing minigenes and/or transfected trans-acting recombinant SR factors and related regulatory proteins. Nevertheless, with the recent estimate that AS may occur in RNA transcripts from 74 - 88% of human genes [42], [43], pursuit at a global level would be required to elucidate the physiological and/or pathological significance of altered AS and the correlated regulatory networks. Many prior studies of the cellular and molecular biology of cancer have reported the occurrence of aberrant AS of important regulatory genes without mutation; however, genome-wide analyses of aberrant AS in various cancer phenotypes, including tumor initiation and malignant progression, remains incomplete [43]. In this study of the Huh-7 cell model of human cancer, we initially exploited known “oncogenic” AS and then used a combination of biochemical, proteomic, genomic and bioinformatic approaches to address pertinent questions of drug modulation in terms of the AS mechanisms. The 2-dimensional gel blotting and proteomic techniques were able to elucidate drug-induced serine phosphorylation changes of SR proteins and related factors. The use of exon array data for computational nucleotide sequence search within the exons and their5′ and 3′ flanking introns, further defined the location and degeneracy of binding and splicing site selection by individual regulatory factors (Table S3). However, this approach could determine only the steady-state AS isoforms at a fixed 24-hour time-point but not kinetic changes, thus was likely to miss AS modification of gene transcripts with slow turn-over rates. Also, in certain databases, ATM and FANC-M were annotated with DNA repair function but not listed in this gene ontology category. Despite these minor technical complications, the combined exon array and computational analysis proved better than prospective biochemical analysis to assess and predict the cellular biology outcome of amiloride-treated Huh-7 cells. This approach demonstrated that more extensive cell death in addition to apoptosis occurred as a consequence of modified AS of DNA repair protein factors, in addition to anti-apoptosis factors like *BCL-XL* isoform (Figure 7).

Modification of AS in many cytoskeletal network genes in addition to *RON* also explains the marked inhibition of invasive activity and cell cycle cytokinesis (Figure 6). Perhaps most significantly, the present approach revealed that amiloride could affect the AS of some genes (Tables S1 & S2), such as *ATM* and *FANCM*, mutation of which is associated with leukemogenesis. Our additional exon array experiments indicated that many, if not most, of the amiloride-affected 495 known protein-coding mRNAs in Huh cells were also modulated by amiloride in another human solid tumor and a leukemic cell line (Fig. S4). It would be of interest to investigate if any of the 187 genes commonly modulated by amiloride in Huh-7, A549 and 8401 cells would participate in the development of the malignant properties and thus could be exploited as target(s) for drug discovery. As to the question whether or not the *in vitro* amiloride effects found in this study could be reproduced *in vivo* for direct cancer therapy, it should be noted that the 0.02 mM to 0.5 mM amiloride concentrations, which could alter oncogenic RNA splicing to devitalize to human cancer cells but not some human normal cells we tested, are 1- to 3-order higher than peripheral blood plasma levels achieved by doses of amiloride administered clinically for anti-hypertensive purpose. Systemic animal tumor model studies with pharmacological approaches and further translational research would be worthwhile to address these critical problems concerning clinical application of amiloride for cancer therapy.

## Materials and Methods

### Cell culture and drug treatment

Huh-7 human hepatocellular carcinoma (HCC) cell line, Huh-7, was purchased through Food Industry Research and Development Institute (Hsinchu, Taiwan). Other established cell lines originally from ATCC (Rockville, MD) were HCC HepG2 (HB8065) and Hep3B (HB8064), colon cancer HT29 (HTB-38) Inv3 clone [44], promyeolocytic leukemia HL60 (CCL-240) and acute lymphoblastic leukemia Molt4 (CRL-1582). Two lines of glioblastoma multiforme, ASCG13T1xm and ASCG7T-4, and a liver-metastatic colon cancer line COW[45] of Chinese patients' origin were established by one of us (DMY). Our stocks of chronic myeloid blasts K562[46], glioblastoma multiforme 8401[47] and a normal bone marrow mesenchymal cell line KP-hMSC[48] were from original investigators, the late Dr. Bismark Lozzio, Dr.Harn and one of us (WKY), respectively. Rhabdomyosarcoma cell line,TE671, was from Dr. LJ Chang (University of Florida).

The monolayer of Huh-7 cells was grown in DMEM, other solid tumor monolayers in MEM and leukemic cell suspension in RPMI-1640 (plus 0.05 mM mercaptoethanol), all supplemented with 10% FBS, 100 U/ml penicillin, and 100 µg/ml streptomycin (Invitrogen Inc, USA), and maintained at 37°C in a humidified 5% CO_2_ incubator. All chemical reagents including amiloride, amiloride derivatives, okadaic acid, AKT pathway inhibitors and cell culture grade anhydride DMSO were obtained from Sigma Chemical Co., USA. Stock solution of amiloride (250 mM) was prepared in DMSO. For dose response experiments, we treated cells (1x10^6^/ml plated on a 100-mm dish overnight) with various concentrations of drugs in the growth medium for 24 hours. For kinetic studies, we added 0.5 mM amiloride to the medium and harvested the cells at various time points.

### RNA isolation, reverse transcription and PCR amplification (RT-PCR)

RNA isolation from cultured cells and RT-PCR analysis were performed as described [19], [49]. For analysis of *BCL-X*, *HIPK3* U-exon, and *RON* alternatively spliced mRNA isoforms, PCR was performed with the following primer pairs:

*BCL-X* forward, 5′-GAGGCAGGCGACGAGTTTGAA-3′,

*BCL-X* reverse, 5′-TGGGAGGGTAGAGTGGATGGT-3′;

*HIPK3* forward, 5′-AGCCTGCCACTACCAAGAAA-3′,

*HIPK3* reverse, 5′-CAGCAATTTCTTGCCTCTCC-3′;

*RON* forward, 5′-GATGGAGCTGCTGGCTTTAC-3′,

*RON* reverse, 5′-ATACCAAGGAGCGTGCTCTG-3′.

The PCR was performed with a denaturing step at 94°C for 5 minutes, then 35 cycles of 30 seconds at 94°C, 30 seconds at 56°C (*BCL-*X), 58°C (*HIPK3* u-exon), or 54°C (*RON*) and 1 minute at 72°C, followed by a final 5 minutes at 72°C. The PCR products were separated on 2.5% agarose gel and the intensity of the PCR products were analyzed by LabWorks Image Acquisition and Analysis Software (UVP BioImaging Systems). DNA gel bands of these RT-PCR products were isolated for sequencing to verify the authenticity of spliced isoforms.

### Protein extracts and Western blotting

The cytoplasmic and nuclear fractions of cells were isolated by using NE-PER Reagent (Pierce Inc., USA) as described previously [49]. Total protein extracts were isolated by using a lysis buffer solution containing 50 mM Tris-HCl (pH 7.5), 137 mM sodium chloride, 1 mM EDTA, 1% Nonidet P-40, 10% glycerol, 0.1 mM sodium orthovanadate, 10 mM sodium pyrophosphate, 20 mM β-glycerophosphate, 50 mM sodium fluoride, 1 mM phenylmethylsulfonyl fluoride, 2 µM leupeptin, and 2 µg/ml aprotinin. The protein samples were separated by SDS-PAGE and then transferred to polyvinylidene fluoride membranes (Millipore). The membrane was blocked wi th 5% BSA and then exposed to the appropriate concentrations of primary antibodies at 4°C overnight, followed by horseradish peroxidase-conjugated secondary antibody for detection by enzyme chemiluminescence kit (Amersham Inc., USA). Intensity of the signals was measured by using the LabWorks software (UVP BioImaging Systems).

### Proteomic analysis of serine phosphorylation proteins

100 µg of protein extracts were mixed with sample buffer, which contained 9M urea, 0.5%(v/v) Triton X100, 10 mM DTT, 0.01% (w/v) bromophenol bule, and 2% (v/v) pH 3–10 ampholytes, and incubated for 30 minutes at 37°C; insoluble material was removed by centrifugation at 14,000 rpm for 10 minutes at 25°C. First-dimensional IEF was performed at 20°C in 13-cm immobilized pI 3–10 linear gradient strips at 50 µA/strip and for 24,500 V/h using the IPGphor system (Amersham Biosciences). The strips were first rehydrated for 6 hours at 25°C in rehydration buffer containing 8M urea, 0.5%(v/v) Triton X100, 10 mM DTT, 0.01% (w/v) Orange G, and 0.5% (v/v) pH 3–10 ampholytes. After focusing, the strips were immediately equilibrated for 15 minutes at 25°C with gentle shaking in 10 ml of 50 mM Tris-HCl (pH 8.8), 6 M urea, 50% (w/v) glycerol, 2% (w/v) SDS, and 0.01% (w/v) bromophenol blue. The second dimension electrophoresis was performed on 12.5% SDS gels in a Hofer DALTTM tank for 4 h at 20 mA per gel. We next performed western blot with anti-phosphoserine antibody and recorded the signal intensity by using the LabWorks software. Computerized 2-D gel analysis (spot detection, spot editing, pattern matching, and data analysis) was performed using the ImageMasterTM Platinum v5.0 software package. Protein spot changes showing increase or decrease of >50 % in magnitude were scored between the treated and the control sample pair.

### Mass spectrometry and protein identification

Protein spots were excised from preparative gels with a pipette tip, de-stained and in-gel digested as described [49] with some modifications. Briefly, the spots were de-stained using 50 mM NH4HCO3 in 50% acetonitrile and dried in a Speed Vac concentrator. The protein was then digested by incubation overnight at 37°C with sequencing grade trypsin (Promega, Madison, WI, USA) in 50 mM NH4HCO3, pH 7.8. The resulting peptides were extracted sequentially with 100 µl of 1% TFA and 100 µl of 0.1% TFA/60% acetonitrile and the combined extracts lyophilized, re-suspended in 10µl of 5% acetonitrile in 0.5% formic acid. Tryptic digest samples were loaded onto a C18 pre-concentration column (5 mm×250 um, PepMap C18, LC Packings, Amsterdam, Netherlands), and the peptides separated on a reverse phase nano-column (15 cm×75 cm, LC Packings). Peptide separation was performed using a 60-minute linear gradient of 5 to 95% acetonitrile in 0.5% formic acid at a flow rate of 200 µl/min. Peptides were characterized using a Qstar XL Q-TOF mass spectrometer (Applied Biosystems). For protein identification, the peaklist-generating software was mascot.d11 version 1.6b23 of Analyst^R^QSv1.1 build 9865, with the default parameter, in Mascot MS/MS Ions Search version 2.1.04 (Mascot Daemon, Matrix Science) using trypsin for enzyme specificity / permission of 2 missed cleavages / variable modification of carbmidomethyl (C) and oxidation (M) / 0.3 Da of mass tolerance for precursor ions and 0.5 Da of mass tolerance for fragment ions; the search against the Swissport protein database (NCBInr 2006.01.20) covered 6555851 sequences of the *Homo sapiens* subset, with Mascot score larger than 50 as the cut-off score/expectation value for accepting individual MS/MS spectrum. As our dataset was small, no estimation of false positive rate or redundancy of peptide matching multiple members of a protein family was performed. Individual proteins identified by the search, particularly the SF2/ASF phosphoprotein isoforms, were verified by specific immunoblot (western) analysis.

### Sample preparation and hybridization on Affymetrix Exon Chip

We used the RNeasy Mini kit (Qiagen Inc., USA) to isolate total RNA according to the manufacturer's instructions and verified the RNA quality by using RNA 6000 NanoAssay on an 2100 Bioanalyzer (Agilent). Two µg of total RNA, labeled according to the GeneChip® Whole Transcript Sense Target Labeling Assay manual of the manufacturer (Affymetrix) was hybridized to Human Exon 1.0 ST Arrays (Affymetrix) for 16 hours at 45°C. The hybridized arrays were washed and stained on a GeneChip Fluidics Station 450 and scanned on a GCS3000 Scanner (Affymetrix).

### Microarray data normalization

The values of the hybridized probesets were normalized and analyzed for gene expression using dChip2006 software (http://biosun1.harvard.edu/~cli/dchip2006.exe). The GeneChip® Human Exon 1.0 ST microarray system has only perfect match probes, but no mismatch (MM) probes available to perform data normalization. Instead of the MM probes, we inferred and removed the existing systematic biases based on the observed intensities of the background probes (BGP) that were pre-designed by Affymetrix. The raw data have been deposited in a MIAME compliant database (GEO accession number #GSE24581).

### Pre-processing and finding putative AS events from Exon Chip

Single probe set mappings were performed over NCBI RefSeqs and the exon-intron genomic coordinates of each known human transcript were downloaded from UCSC Table Browser version hg18 [50]. We applied three criteria to filter the putative AS events from the exon chip. First, Pearson's correlation coefficient was used to compare the gene expression profiles between control and amiloride samples. Second and third, we used the normalized values of array intensities of exon expression for calculating the splicing index, as previously described[51], and also for obtaining the log2 ratio. The 3 criteria are combined to filter the putative AS events.

### Identification of over-represented GO Terms

We used the Gene Ontology (GO) resource of the database DAVID [52] to identify common functions shared by genes identified by high-throughput gene expression methods. The hypergeometric test [53] was applied to calculate the significance of enrichment (*P*) for a given over-representation GO category by 
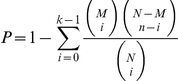
, where N is the total number of genes in the background distribution, M is the number of genes within the distribution that are annotated to the node of interest, n is the size of the list of interesting genes and K is the number of genes within that list which are annotated to the node.

### Computational analysis of regulatory factors in AS mechanisms

The sequences within the alternatively spliced exon and its flanking 5′ and 3′ introns werer analyzed for deciphering the regulatory mechanisms of AS. The motif discovery tool, MEME[54], was employed to search for motif sequences related to the SR proteins and other regulatory factors of the AS mechanisms. The DNA motifs discovered in the genes showing modified RNA AS were listed according to the intronic or exonic regions and the consensus nucleotide sequence patterns or position matrices for data storage and analysis. A nucleotide sequence logo [55] was created for each motif by alignment of multiple consensus and consensus-like sequences to provide a more detailed precise description than a consensus sequence of the binding site of a regulatory factor involved in the modified AS.

### Multi-color fluorocytometry

Cells treated with or without amiloride were collected at various time intervals and washed once with cold PBS by centrifugation (300 xg for 5 min at 4°C). The harvested cells were incubated in 0.05 ml of PBS containing an APC-labeled monoclonal antibody, CD133/Prominin (BD Pharmingen USA), for 30 min in the dark and then added fluorescein-labeled annexinV and propidium iodide for another 30 min of incubation. After washing once with cold PBS, the triply stained cells were analyzed in a flow fluorocytometer with BD FACSDiVa software (BD Biosciences) to distinguish and quantify the bound fluorescent antibody, the propidium-stained dead cells and the annexinV-binding apoptotic cells. For simultaneous measurement of apoptosis and cellular DNA content, cells incubated with FITC-labeled annexinV were washed, suspended and fixed in 70% ethanol at −20°C for 3 hr. The fixed cells were precipitated, washed, and stained in 10 µg/ml propidium iodide and 0.2 mg/ml ribonuclease (Sigma Co.). The bound annexinV and propidium-labeled DNA content of individual cells were analyzed for apoptosis and cell cycle effects by flow fluorocytometry.

### Confocal and light microscopy of cytokinesis

Mitotic cells shaken off the rapidly growing monolayer cultures were pelleted by centrifugation, suspended at 1.5x104/ml in growth medium with or without amiloride and plated on 6-well dishes or 4-chamber glass slide dishes (1-ml per well or chamber). After 24, 48 and 72 hours of incubation, the cells were fixed in 2% paraformadehyde at 4°C overnight and rinsed several times with cold PBS. For examination of actin-associated cytoskeleton, cells on slide chambers were permealized with 0.1% triton X-100/0.1 % BSA in PBS, stained with FITC-labeled phalloidin (1 µg/ml) and DAPI (0.1 µg/ml) in PBS and examined under fluorescent microscopy. Confocal cell images were taken with a Leica CCD camera using Leica Image manager 50 software. For light microscopic observation of post-mitotic cytokinesis, paraformaldehyde-fixed PBS-washed cells on 6-well dishes were rinsed with 50% methanol and 100% methanol before Wright-Giemsa staining. Cell images were recorded by a digital camera (DP70) equipped on an inverted microscope (Olympus). The distance between two daughter nuclei was measured manually using equally magnified images on computer screen or hard-copy prints. The average and standard deviation was calculated from data of more than 10 individual cells (dividing or divided) per sample to analyze the cytokinetic migration.

## Supporting Information

Figure S1 **Effects of PI3K pathway inhibitors on SF2/ASF and isoforms of HIPK3 and BCL-X in Huh-7 cells.**
(TIF)

Figure S2 **Inhibitory effects of amiloride treatment on CD133 expression and clonogenicity of Huh-7 cells.**
(TIF)

Figure S3 **Cytotoxic and growth inhibitory effects of amiloride on various human cancer leukemia and normal cells in vitro.**
(TIF)

Figure S4 **Characterization of common genes subject to amiloride modulation of RNA alternative splicing in three human neoplastic cell lines.**
(TIF)

Table S1 **Top scoring candidate gene transcripts showing amiloride-altered alternative splicing in Huh-7 cells. The raw data have been deposited in a MIAME compliant database (GEO accession number #GSE24581).**
(PDF)

Table S2 **The significant GO terms of genes with alternative RNA splicing altered in amiloride-treated Huh-7 cells.**
(PDF)

Table S3 **The splicing factor-binding exonic and intronic elements of genes showing altered alternative RNA splicing in amiloride-treated Huh-7 cells.**
(PDF)

## References

[1] Maniatis T, Tasic B (2002). Alternative pre-mRNA splicing and proteome expansion in metazoans.. Nature.

[2] Stetefeld J, Ruegg MA (2005). Structural and functional diversity generated by alternative mRNA splicing.. Trends Biochem Sci.

[3] Black DL (2003). Mechanisms of alternative pre-messenger RNA splicing.. Annu Rev Biochem.

[4] Matlin AJ, Clark F, Smith CW (2005). Understanding alternative splicing: towards a cellular code.. Nat Rev Mol Cell Biol.

[5] Fu XD (1995). The superfamily of arginine/serine-rich splicing factors.. RNA.

[6] Hertel KJ, Graveley BR (2005). RS domains contact the pre-mRNA throughout spliceosome assembly.. Trends Biochem Sci.

[7] Pozzoli U, Sironi M (2005). Silencers regulate both constitutive and alternative splicing events in mammals.. Cell Mol Life Sci.

[8] Schwerk C, Schulze-Osthoff K (2005). Regulation of apoptosis by alternative pre-mRNA splicing.. Mol Cell.

[9] Stamm S, Ben-Ari S, Rafalska I, Tang Y, Zhang Z (2005). Function of alternative splicing.. Gene.

[10] Caceres JF, Kornblihtt AR (2002). Alternative splicing: multiple control mechanisms and involvement in human disease.. Trends Genet.

[11] Pajares MJ, Ezponda T, Catena R, Calvo A, Pio R (2007). Alternative splicing: an emerging topic in molecular and clinical oncology.. Lancet Oncol.

[12] Srebrow A, Kornblihtt AR (2006). The connection between splicing and cancer.. J Cell Sci.

[13] Venables JP (2006). Unbalanced alternative splicing and its significance in cancer.. Bioessays.

[14] Wang GS, Cooper TA (2007). Splicing in disease: disruption of the splicing code and the decoding machinery.. Nat Rev Genet.

[15] Takehara T, Liu X, Fujimoto J, Friedman SL, Takahashi H (2001). Expression and role of Bcl-xL in human hepatocellular carcinomas.. Hepatology.

[16] Curtin JF, Cotter TG (2004). JNK regulates HIPK3 expression and promotes resistance to Fas-mediated apoptosis in DU 145 prostate carcinoma cells.. J Biol Chem.

[17] Bardella C, Costa B, Maggiora P, Patane S, Olivero M (2004). Truncated RON tyrosine kinase drives tumor cell progression and abrogates cell-cell adhesion through E-cadherin transcriptional repression.. Cancer Res.

[18] Chen Q, Seol DW, Carr B, Zarnegar R (1997). Co-expression and regulation of Met and Ron proto-oncogenes in human hepatocellular carcinoma tissues and cell lines.. Hepatology.

[19] Yuo CY, Lin HH, Chang YS, Yang WK, Chang JG (2008). 5-(N-ethyl-N-isopropyl)-amiloride enhances SMN2 exon 7 inclusion and protein expression in spinal muscular atrophy cells.. Ann Neurol.

[20] Ghigna C, Giordano S, Shen H, Benvenuto F, Castiglioni F (2005). Cell motility is controlled by SF2/ASF through alternative splicing of the Ron protooncogene.. Mol Cell.

[21] Cao W, Jamison SF, Garcia-Blanco MA (1997). Both phosphorylation and dephosphorylation of ASF/SF2 are required for pre-mRNA splicing in vitro.. RNA.

[22] Kim KM, Lee YJ (2005). Amiloride augments TRAIL-induced apoptotic death by inhibiting phosphorylation of kinases and phosphatases associated with the P13K-Akt pathway.. Oncogene.

[23] Koizumi J, Okamoto Y, Onogi H, Mayeda A, Krainer AR (1999). The subcellular localization of SF2/ASF is regulated by direct interaction with SR protein kinases (SRPKs).. J Biol Chem.

[24] Blaustein M, Pelisch F, Tanos T, Munoz MJ, Wengier D (2005). Concerted regulation of nuclear and cytoplasmic activities of SR proteins by AKT.. Nat Struct Mol Biol.

[25] Cardinali B, Cohen PT, Lamond AI (1994). Protein phosphatase 1 can modulate alternative 5′ splice site selection in a HeLa splicing extract.. FEBS Lett.

[26] Misteli T, Spector DL (1996). Serine/threonine phosphatase 1 modulates the subnuclear distribution of pre-mRNA splicing factors.. Mol Biol Cell.

[27] Patel NA, Kaneko S, Apostolatos HS, Bae SS, Watson JE (2005). Molecular and genetic studies imply Akt-mediated signaling promotes protein kinase C beta II alternative splicing via phosphorylation of serine/arginine-rich splicing factor SRp40.. Journal of Biological Chemistry.

[28] Pelisch F, Blaustein M, Kornblihtt AR, Srebrow A (2005). Cross-talk between signaling pathways regulates alternative splicing - A novel role for JNK.. Journal of Biological Chemistry.

[29] Huang Y, Yario TA, Steitz JA (2004). A molecular link between SR protein dephosphorylation and mRNA export.. Proc Natl Acad Sci U S A.

[30] Medhurst AL, Laghmani EH, Steltenpool J, Ferrer M, Fontaine C (2006). Evidence for subcomplexes in the Fanconi anemia pathway.. Blood.

[31] Xiao SH, Manley JL (1997). Phosphorylation of the ASF/SF2 RS domain affects both protein-protein and protein-RNA interactions and is necessary for splicing.. Genes Dev.

[32] Ngo JCK, Giang K, Chakrabarti S, Ma CT, Huynh N (2008). A sliding docking interaction is essential for sequential and processive phosphorylation of an SR protein by SRPK1.. Molecular Cell.

[33] Skop AR, Liu HB, Yates J, Meyer BJ, Heald R (2004). Dissection of the mammalian midbody proteome reveals conserved cytokinesis mechanisms.. Science.

[34] Suetsugi A, Nagaki M, Aoki H, Motohashi T, Kunisada T (2006). Characterization of CD133(+) hepatocellular carcinoma cells as cancer stem/progenitor cells.. Biochemical and Biophysical Research Communications.

[35] Chang WH, Liu TC, Yang WK, Lee CC, Lin YH, et al. Amiloride modulates alternative splicing in leukemic cells and resensitizes Bcr-AblT315I mutant cells to imatinib.. Cancer Res.

[36] Bull MB, Laragh JH (1968). Amiloride. A potassium-sparing natriuretic agent.. Circulation.

[37] Swift PA, MacGregor GA (2004). The epithelial sodium channel in hypertension: genetic heterogeneity and implications for treatment with amiloride.. Am J Pharmacogenomics.

[38] Yun CY, Velazquez-Dones AL, Lyman SK, Fu XD (2003). Phosphorylation-dependent and -independent nuclear import of RS domain-containing splicing factors and regulators.. Journal of Biological Chemistry.

[39] Prasad J, Colwill K, Pawson T, Manley JL (1999). The protein kinase Clk/Sty directly modulates SR protein activity: both hyper- and hypophosphorylation inhibit splicing.. Mol Cell Biol.

[40] Gui JF, Lane WS, Fu XD (1994). A serine kinase regulates intracellular localization of splicing factors in the cell cycle.. Nature.

[41] Davis RJ, Czech MP (1985). Amiloride directly inhibits growth factor receptor tyrosine kinase activity.. Journal of Biological Chemistry.

[42] Johnson JM, Castle J, Garrett-Engele P, Kan Z, Loerch PM (2003). Genome-wide survey of human alternative pre-mRNA splicing with exon junction microarrays.. Science.

[43] Ben-Dov C, Hartmann B, Lundgren J, Valcarcel J (2008). Genome-wide analysis of alternative pre-mRNA splicing.. Journal of Biological Chemistry.

[44] Chen WS, Wei SJ, Liu JM, Hsiao M, Kou-Lin J (2001). Tumor invasiveness and liver metastasis of colon cancer cells correlated with cyclooxygenase-2 (COX-2) expression and inhibited by a COX-2-selective inhibitor, etodolac.. Int J Cancer.

[45] Juang SH, Wei SJ, Hung YM, Hsu CY, Yang DM (2004). IFN-beta induces caspase-mediated apoptosis by disrupting mitochondria in human advanced stage colon cancer cell lines.. J Interferon Cytokine Res.

[46] Lozzio BB, Lozzio CB (1977). Properties of the K562 cell line derived from a patient with chronic myeloid leukemia.. Int J Cancer.

[47] Harn HJ, Lee HS, Ho LI, Lee WH, Ding JH (1994). Selective expression of CD44 messenger RNA splice variants in four high grade human brain tumour cell lines.. Biochem Mol Biol Int.

[48] Hung SC, Yang DM, Chang CF, Lin RJ, Wang JS (2004). Immortalization without neoplastic transformation of human mesenchymal stem cells by transduction with HPV16 E6/E7 genes.. Int J Cancer.

[49] Chan CH, Ko CC, Chang JG, Chen SF, Wu MS (2006). Subcellular and functional proteomic analysis of the cellular responses induced by Helicobacter pylori.. Mol Cell Proteomics.

[50] Karolchik D, Hinrichs AS, Furey TS, Roskin KM, Sugnet CW (2004). The UCSC Table Browser data retrieval tool.. Nucleic Acids Res.

[51] Srinivasan K, Shiue L, Hayes JD, Centers R, Fitzwater S, et al. (2005). Detection and measurement of alternative splicing using splicing-sensitive microarrays.. Methods.

[52] Dennis G, Sherman BT, Hosack DA, Yang J, Gao W (2003). DAVID: Database for Annotation, Visualization, and Integrated Discovery.. Genome Biol.

[53] Boyle EI, Weng S, Gollub J, Jin H, Botstein D (2004). GO::TermFinder--open source software for accessing Gene Ontology information and finding significantly enriched Gene Ontology terms associated with a list of genes.. Bioinformatics.

[54] Bailey TL, Williams N, Misleh C, Li WW (2006). MEME: discovering and analyzing DNA and protein sequence motifs.. Nucleic Acids Res.

[55] Crooks GE, Hon G, Chandonia JM, Brenner SE (2004). WebLogo: a sequence logo generator.. Genome Res.

