# Supplementary figures and images for: Small Molecule Amiloride Modulates Oncogenic RNA Alternative Splicing to Devitalize Human Cancer Cells
Source: PLoS One. 2011 Jun 9;6(6):e18643. doi: 10.1371/journal.pone.0018643 (PMC3111415; doi:10.1371/journal.pone.0018643)

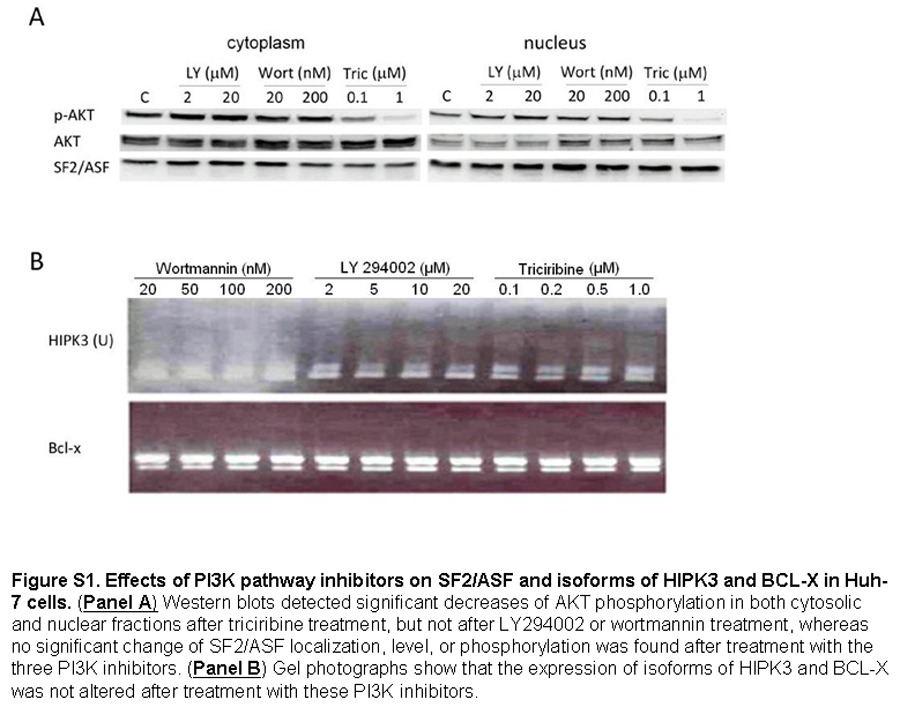

Supplement: Figure S1 — Effects of PI3K pathway inhibitors on SF2/ASF and isoforms of HIPK3 and BCL-X in Huh-7 cells. (TIF) [file pone.0018643.s001.tif]

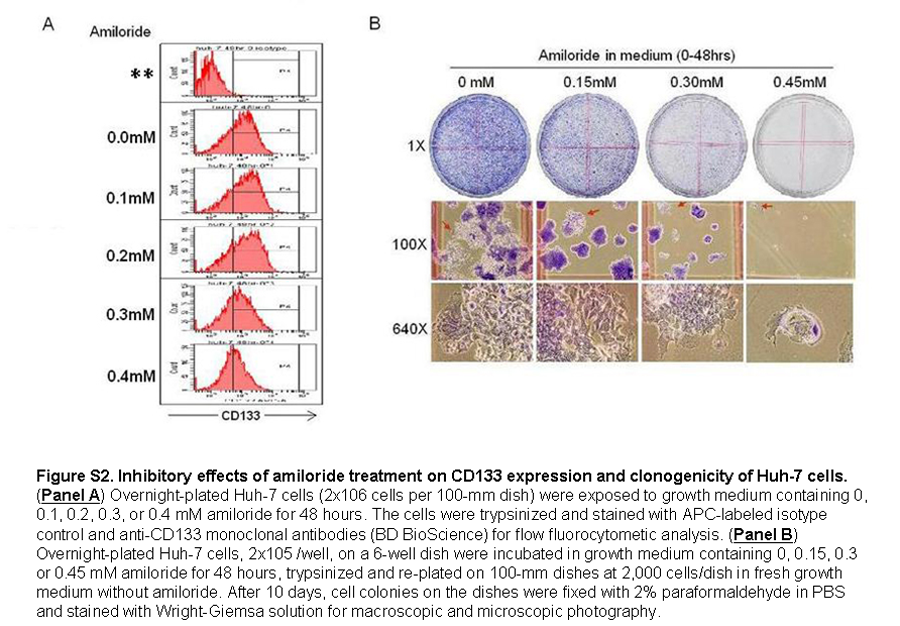

Supplement: Figure S2 — Inhibitory effects of amiloride treatment on CD133 expression and clonogenicity of Huh-7 cells. (TIF) [file pone.0018643.s002.tif]

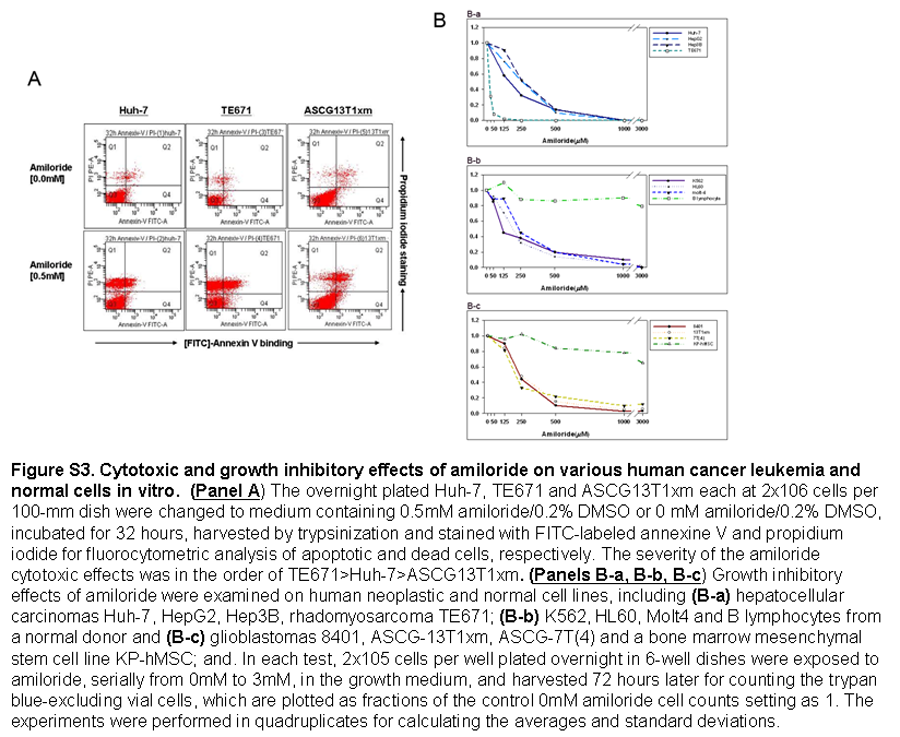

Supplement: Figure S3 — Cytotoxic and growth inhibitory effects of amiloride on various human cancer leukemia and normal cells in vitro. (TIF) [file pone.0018643.s003.tif]

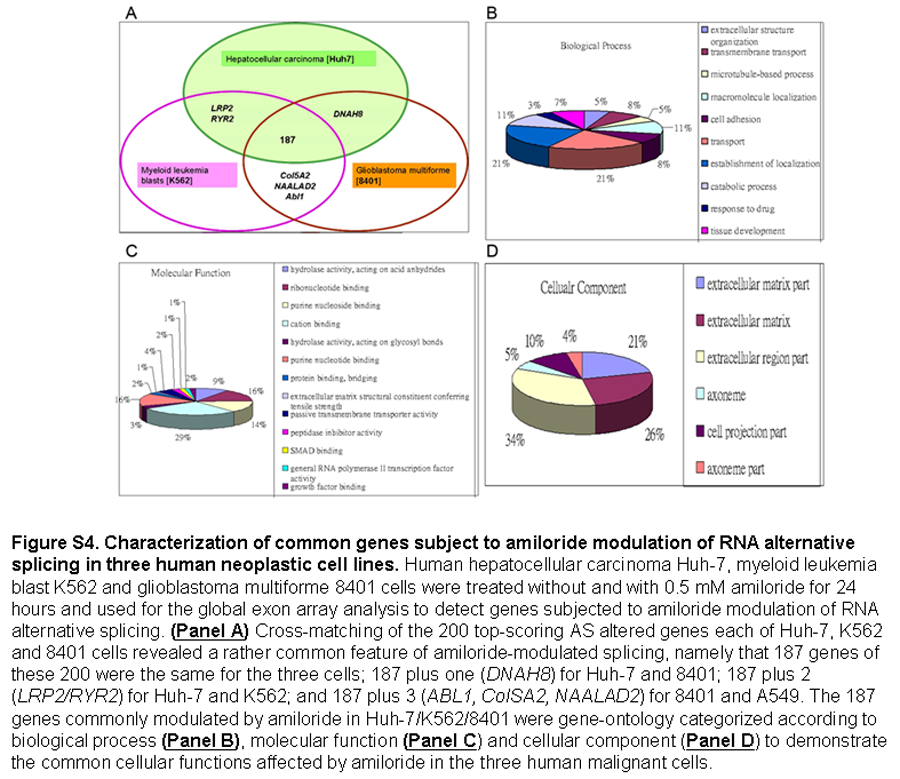

Supplement: Figure S4 — Characterization of common genes subject to amiloride modulation of RNA alternative splicing in three human neoplastic cell lines. (TIF) [file pone.0018643.s004.tif]
