# Supplementary material for: Small Molecule Amiloride Modulates Oncogenic RNA Alternative Splicing to Devitalize Human Cancer Cells
Source: PLoS One. 2011 Jun 9;6(6):e18643. doi: 10.1371/journal.pone.0018643 (PMC3111415; doi:10.1371/journal.pone.0018643)
Supplement: Table S1 — Top scoring candidate gene transcripts showing amiloride-altered alternative splicing in Huh-7 cells. The raw data have been deposited in a MIAME compliant database (GEO accession number #GSE24581). (PDF) [file pone.0018643.s005.pdf]

Supplementary Table 1. Top scoring candidates of alternative splicing events.

AS_Exon: alternative splicing exon. Splicing_index: represents the log ratio of the exon intensities between the two samples after normalization to the gene intensities in each condition. Log_2_ratio=$Log_2(Exon_{amiloride\text{-}treatment}/Exon_{control})$. Correlation_Coefficient: comparing the gene expression profiles between control and amiloride-treatment samples.

Gene_Symbol	Gene_Description	AS_Exon	Splicing_index	Log_2_ratio	Correlation_Coefficent
ABCC2	Canalicular multispecific organic anion transporter 1 (ATP-binding cassette sub-family C member 2) (Multidrug resistance-associated protein 2) (Canalicular multidrug resistance protein). [Source:Uniprot/SWISSPROT;Acc:Q92887]	28	-2.16	-2.35	0.724
ABCC4	Multidrug resistance-associated protein 4 (ATP-binding cassette sub- family C member 4) (MRP/cMOAT-related ABC transporter) (Multi-specific organic anion transporter-B) (MOAT-B). [Source:Uniprot/SWISSPROT;Acc:O15439]	28	-1.961	-1.923	0.718
ACACA	Acetyl-CoA carboxylase 1 (EC 6.4.1.2) (ACC-alpha) [Includes: Biotin carboxylase (EC 6.3.4.14)]. [Source:Uniprot/SWISSPROT;Acc:Q13085]	4	-2.524	-2.74	0.724
ACMSD	2-amino-3-carboxymuconate-6-semialdehyde decarboxylase (EC 4.1.1.45). [Source:Uniprot/SWISSPROT;Acc:Q8TDX5]	10	-2.135	-2.431	0.975
ACO1	Iron-responsive element-binding protein 1 (IRE-BP 1) (Iron regulatory protein 1) (IRP1) (Ferritin repressor protein) (Aconitate hydratase) (EC 4.2.1.3) (Citrate hydro-lyase) (Aconitase). [Source:Uniprot/SWISSPROT;Acc:P21399]	20	-1.782	-1.709	0.849
ACOT12	Acyl-coenzyme A thioesterase 12 (EC 3.1.2.1) (Acyl-CoA thioesterase 12) (Acyl-CoA thioester hydrolase 12) (Cytoplasmic acetyl-CoA hydrolase 1) (CACH-1) (hCACH-1) (START domain-containing protein 12) (StARD12). [Source:Uniprot/SWISSPROT;Acc:Q8WYK0]	10	-1.594	-1.767	0.939

Gene_Symbol	Gene_Description	AS_Exon	Splicing_index	Log_2_ratio	Correlation_Coefficent
ACSL6	Long-chain-fatty-acid--CoA ligase 6 (EC 6.2.1.3) (Long-chain acyl-CoA synthetase 6) (LACS 6). [Source:Uniprot/SWISSPROT;Acc:Q9UKU0]	14	-1.724	-1.606	0.953
ACSS1	Acetyl-coenzyme A synthetase 2-like, mitochondrial precursor (EC 6.2.1.1) (Acetate--CoA ligase 2) (Acetyl-CoA synthetase 2) (Acyl- CoA synthetase short-chain family member 1). [Source:Uniprot/SWISSPROT;Acc:Q9NUB1]	13	-1.938	-2.165	0.78
ADAM32	ADAM 32 precursor (A disintegrin and metalloproteinase domain 32). [Source:Uniprot/SWISSPROT;Acc:Q8TC27]	5	-1.892	-1.873	0.877
ADAMTS10	ADAMTS-10 precursor (EC 3.4.24.-) (A disintegrin and metalloproteinase with thrombospondin motifs 10) (ADAM-TS 10) (ADAM-TS10) (Fragment). [Source:Uniprot/SWISSPROT;Acc:Q9H324]	20	-1.691	-1.704	0.861
ADAMTS12	ADAMTS-12 precursor (EC 3.4.24.-) (A disintegrin and metalloproteinase with thrombospondin motifs 12) (ADAM-TS 12) (ADAM-TS12). [Source:Uniprot/SWISSPROT;Acc:P58397]	20	-1.707	-1.722	0.858
ADCK5	Uncharacterized aarF domain-containing protein kinase 5 (EC 2.7.-.-). [Source:Uniprot/SWISSPROT;Acc:Q3MIX3]	5	-2.021	-1.861	0.898
ADRBK2	Beta-adrenergic receptor kinase 2 (EC 2.7.11.15) (Beta-ARK-2) (G- protein-coupled receptor kinase 3). [Source:Uniprot/SWISSPROT;Acc:P35626]	18	-1.666	-1.798	0.729
AFF3	AF4/FMR2 family member 3 (Protein LAF-4) (Lymphoid nuclear protein related to AF4). [Source:Uniprot/SWISSPROT;Acc:P51826]	4	-1.604	-1.999	0.707
AHI1	Jouberin (Abelson helper integration site 1 protein homolog) (AHI-1). [Source:Uniprot/SWISSPROT;Acc:Q8N157]	20	-1.835	-1.768	0.712
AIM1	Absent in melanoma 1 protein. [Source:Uniprot/SWISSPROT;Acc:Q9Y4K1]	5	-2.027	-1.89	0.894

Gene_Symbol	Gene_Description	AS_Exon	Splicing_index	Log_2_ratio	Correlation_Coefficent
AKT3	RAC-gamma serine/threonine-protein kinase (EC 2.7.11.1) (RAC-PK-gamma) (Protein kinase Akt-3) (Protein kinase B, gamma) (PKB gamma) (STK-2). [Source:Uniprot/SWISSPROT;Acc:Q9Y243]	15	-3.397	-3.183	0.886
ALDH8A1	aldehyde dehydrogenase 8A1 isoform 2 [Source:RefSeq_peptide;Acc:NP_739577]	1	-1.604	-1.869	0.77
ALMS1	Alstrom syndrome protein 1. [Source:Uniprot/SWISSPROT;Acc:Q8TCU4]	20	-2.203	-2.069	0.912
ALS2	Alsin (Amyotrophic lateral sclerosis protein 2). [Source:Uniprot/SWISSPROT;Acc:Q96Q42]	20	-1.772	-1.928	0.924
ALS2CR12	Amyotrophic lateral sclerosis 2 chromosomal region candidate gene 12 protein. [Source:Uniprot/SWISSPROT;Acc:Q96Q35]	10	-2.096	-2.34	0.961
ANKHD1	Eukaryotic translation initiation factor 4E-binding protein 3 (4E-BP3) (eIF4E-binding protein 3). [Source:Uniprot/SWISSPROT;Acc:O60516]	20	-1.765	-1.7	0.76
ANKRD27	Ankyrin repeat domain-containing protein 27 (VPS9 domain-containing protein). [Source:Uniprot/SWISSPROT;Acc:Q96NW4]	20	-1.749	-1.822	0.842
ANKS1B	cajalin 2 isoform a [Source:RefSeq_peptide;Acc:NP_690001]	20	-1.744	-1.766	0.854
ANKS6	Ankyrin repeat and SAM domain-containing protein 6 (Sterile alpha motif domain-containing protein 6) (Ankyrin repeat domain-containing protein 14). [Source:Uniprot/SWISSPROT;Acc:Q68DC2]	5	-1.83	-1.833	0.805
ARFGEF1	Brefeldin A-inhibited guanine nucleotide-exchange protein 1 (Brefeldin A-inhibited GEP 1) (p200 ARF-GEP1) (p200 ARF guanine nucleotide exchange factor). [Source:Uniprot/SWISSPROT;Acc:Q9Y6D6]	28	-1.942	-1.964	0.782
ARFGEF2	Brefeldin A-inhibited guanine nucleotide-exchange protein 2 (Brefeldin A-inhibited GEP 2). [Source:Uniprot/SWISSPROT;Acc:Q9Y6D5]	28	-1.868	-2.061	0.818
ARHGAP17	Rho GTPase-activating protein 17 (Rho-type GTPase-activating protein 17) (RhoGAP interacting with CIP4 homologs protein 1) (RICH-1). [Source:Uniprot/SWISSPROT;Acc:Q68EM7]	20	-1.815	-1.952	0.853

Gene_Symbol	Gene_Description	AS_Exon	Splicing_index	Log_2_ratio	Correlation_Coefficent
ARHGAP27	Rho GTPase activating protein 27 [Source:RefSeq_peptide;Acc:NP_954976]	5	-1.864	-1.669	0.909
ARHGEF10L	Rho guanine nucleotide exchange factor (GEF) 10-like isoform 2 [Source:RefSeq_peptide;Acc:NP_001011722]	20	-1.95	-1.942	0.769
ARID1B	AT-rich interactive domain-containing protein 1B (ARID domain- containing protein 1B) (Osa homolog 2) (hOsa2) (p250R) (BRG1-binding protein hELD/OSA1) (BRG1-associated factor 250b) (BAF250B). [Source:Uniprot/SWISSPROT;Acc:Q8NFD5]	20	-1.596	-1.794	0.934
ARMC4	Armadillo repeat-containing protein 4. [Source:Uniprot/SWISSPROT;Acc:Q5T2S8]	20	-1.918	-1.962	0.712
ASAH2	Neutral ceramidase (EC 3.5.1.23) (NCDase) (N-CDase) (Acylsphingosine deacylase 2) (N-acylsphingosine amidohydrolase 2) (BCDase) (LCDase) (hCD) [Contains: Neutral ceramidase soluble form]. [Source:Uniprot/SWISSPROT;Acc:Q9NR71]	20	-1.755	-1.833	0.874
ATM	Serine-protein kinase ATM (EC 2.7.11.1) (Ataxia telangiectasia mutated) (A-T, mutated). [Source:Uniprot/SWISSPROT;Acc:Q13315]	20	-1.83	-1.922	0.845
ATP12A	Potassium-transporting ATPase alpha chain 2 (EC 3.6.3.10) (Proton pump) (Non-gastric H(+)/K(+) ATPase subunit alpha). [Source:Uniprot/SWISSPROT;Acc:P54707]	20	-2.166	-2.097	0.871
ATP1A2	Sodium/potassium-transporting ATPase alpha-2 chain precursor (EC 3.6.3.9) (Sodium pump 2) (Na(+)/K(+) ATPase 2). [Source:Uniprot/SWISSPROT;Acc:P50993]	20	-2.163	-2.011	0.898
ATP1A3	Sodium/potassium-transporting ATPase alpha-3 chain (EC 3.6.3.9) (Sodium pump 3) (Na(+)/K(+) ATPase 3) (Alpha(III)). [Source:Uniprot/SWISSPROT;Acc:P13637]	20	-1.95	-1.87	0.866

Gene_Symbol	Gene_Description	AS_Exon	Splicing_index	Log_2_ratio	Correlation_Coefficent
ATP1A4	Sodium/potassium-transporting ATPase alpha-4 chain (EC 3.6.3.9) (Sodium pump 4) (Na(+)/K(+) ATPase 4). [Source:Uniprot/SWISSPROT;Acc:Q13733]	20	-1.859	-1.804	0.852
ATP2A1	Sarcoplasmic/endoplasmic reticulum calcium ATPase 1 (EC 3.6.3.8) (Calcium pump 1) (SERCA1) (SR Ca(2+)-ATPase 1) (Calcium-transporting ATPase sarcoplasmic reticulum type, fast twitch skeletal muscle isoform) (Endoplasmic reticulum class 1/2 Ca(2+) ATPase). [Source:Uniprot/SWISSPROT;Acc:O14983]	20	-1.858	-1.825	0.851
ATP6V0A4	Vacuolar proton translocating ATPase 116 kDa subunit a isoform 4 (V- ATPase 116 kDa isoform a4) (Vacuolar proton translocating ATPase 116 kDa subunit a kidney isoform). [Source:Uniprot/SWISSPROT;Acc:Q9HBG4]	20	-1.697	-1.71	0.883
ATP8A1	Probable phospholipid-transporting ATPase IA (EC 3.6.3.1) (Chromaffin granule ATPase II) (ATPase class I type 8A member 1). [Source:Uniprot/SWISSPROT;Acc:Q9Y2Q0]	28	-2.557	-2.479	0.852
ATRX	Transcriptional regulator ATRX (EC 3.6.1.-) (ATP-dependent helicase ATRX) (X-linked helicase II) (X-linked nuclear protein) (XNP) (Znf- HX). [Source:Uniprot/SWISSPROT;Acc:P46100]	20	-1.805	-1.865	0.842
BAI1	Brain-specific angiogenesis inhibitor 1 precursor. [Source:Uniprot/SWISSPROT;Acc:O14514]	20	-1.87	-2.134	0.95
BAI2	Brain-specific angiogenesis inhibitor 2 precursor. [Source:Uniprot/SWISSPROT;Acc:O60241]	20	-1.591	-1.807	0.93
BAI3	Brain-specific angiogenesis inhibitor 3 precursor. [Source:Uniprot/SWISSPROT;Acc:O60242]	20	-1.946	-2.18	0.933
BAT5	Protein BAT5 (HLA-B-associated transcript 5) (Protein G5). [Source:Uniprot/SWISSPROT;Acc:O95870]	20	-2.047	-2.155	0.846
BBS5	Bardet-Biedl syndrome 5 protein. [Source:Uniprot/SWISSPROT;Acc:Q8N3I7]	10	-2.085	-2.416	0.978

Gene_Symbol	Gene_Description	AS_Exon	Splicing_index	Log_2_ratio	Correlation_Coefficent
BCAS3	Breast carcinoma amplified sequence 3 (GAOB1) (Maab1 protein). [Source:Uniprot/SWISSPROT;Acc:Q9H6U6]	20	-1.774	-1.727	0.739
BIRC6	Baculoviral IAP repeat-containing protein 6 (Ubiquitin-conjugating BIR domain enzyme apollon). [Source:Uniprot/SWISSPROT;Acc:Q9NR09]	20	-1.822	-1.841	0.845
BIRC6	Baculoviral IAP repeat-containing protein 6 (Ubiquitin-conjugating BIR domain enzyme apollon). [Source:Uniprot/SWISSPROT;Acc:Q9NR09]	61	-1.694	-1.713	0.845
BMX	Cytoplasmic tyrosine-protein kinase BMX (EC 2.7.10.2) (Bone marrow tyrosine kinase gene in chromosome X protein) (Epithelial and endothelial tyrosine kinase) (ETK) (NTK38). [Source:Uniprot/SWISSPROT;Acc:P51813]	20	-2.033	-2.181	0.913
BRCA1	Breast cancer type 1 susceptibility protein (RING finger protein 53). [Source:Uniprot/SWISSPROT;Acc:P38398]	20	-1.658	-1.8	0.927
BRIP1	Fanconi anemia group J protein (EC 3.6.1.-) (ATP-dependent RNA helicase BRIP1) (Protein FACJ) (BRCA1-interacting protein C-terminal helicase 1) (BRCA1-interacting protein 1) (BRCA1-associated C-terminal helicase 1). [Source:Uniprot/SWISSPROT;Acc:Q9BX63]	20	-1.704	-1.819	0.796
BUB1	Mitotic checkpoint serine/threonine-protein kinase BUB1 (EC 2.7.11.1) (hBUB1) (BUB1A). [Source:Uniprot/SWISSPROT;Acc:O43683]	20	-1.608	-1.641	0.905
BUB1B	Mitotic checkpoint serine/threonine-protein kinase BUB1 beta (EC 2.7.11.1) (hBUBR1) (MAD3/BUB1-related protein kinase) (Mitotic checkpoint kinase MAD3L) (SSK1). [Source:Uniprot/SWISSPROT;Acc:O60566]	20	-1.901	-1.787	0.907
C10orf18	Uncharacterized protein C10orf18. [Source:Uniprot/SPTREMBL;Acc:Q5VWN6]	20	-1.93	-2.055	0.73
C10orf76	Uncharacterized protein C10orf76. [Source:Uniprot/SWISSPROT;Acc:Q5T2E6]	20	-1.848	-1.92	0.845

Gene_Symbol	Gene_Description	AS_Exon	Splicing_index	Log_2_ratio	Correlation_Coefficent
C10orf92	Uncharacterized protein C10orf92. [Source:Uniprot/SWISSPROT;Acc:Q8IYW2]	20	-1.737	-1.839	0.759
C11orf11	Uncharacterized protein C11orf11	20	-1.767	-1.739	0.882
C14orf103	Uncharacterized protein C14orf103. [Source:Uniprot/SWISSPROT;Acc:Q96BY7]	20	-1.714	-1.768	0.761
C14orf135	Uncharacterized protein C14orf135 precursor (Hepatitis C virus F protein-binding protein 2) (HCV F protein-binding protein 2). [Source:Uniprot/SWISSPROT;Acc:Q63HM2]	1	-1.635	-1.883	0.869
C17orf27	Uncharacterized protein C17orf27	20	-1.753	-2.072	0.939
C19orf15	Uncharacterized protein C19orf15 precursor. [Source:Uniprot/SWISSPROT;Acc:Q6ZRH7]	20	-1.941	-2.131	0.927
C1orf112	Uncharacterized protein C1orf112. [Source:Uniprot/SWISSPROT;Acc:Q9NSG2]	20	-1.785	-1.866	0.831
C20orf12	Ankyrin repeat-containing protein C20orf12. [Source:Uniprot/SWISSPROT;Acc:Q9NVP4]	20	-2.037	-2.083	0.832
C20orf132	Uncharacterized protein C20orf132	10	-1.967	-2.037	0.871
C20orf133	C20orf133 protein. [Source:Uniprot/SPTREMBL;Acc:Q495E0]	10	-1.827	-2.073	0.876
C20orf175	Uncharacterized protein C20orf175. [Source:Uniprot/SWISSPROT;Acc:Q96MK2]	10	-1.759	-1.811	0.906
C20orf23	Kinesin-like motor protein C20orf23 (Sorting nexin-23). [Source:Uniprot/SWISSPROT;Acc:Q96L93]	20	-1.712	-1.747	0.763
C3orf44	C3orf44 protein. [Source:Uniprot/SPTREMBL;Acc:Q8NCQ6]	10	-2.162	-2.386	0.981
C6orf107	UHRF1-binding protein 1 (Ubiquitin-like containing PHD and RING finger domains 1-binding protein 1) (ICBP90-binding protein 1). [Source:Uniprot/SWISSPROT;Acc:Q6BDS2]	20	-1.846	-1.915	0.741
C8ORFK23	C8ORFK23 protein	20	-1.907	-2.023	0.93
C9orf39	Uncharacterized protein C9orf39. [Source:Uniprot/SWISSPROT;Acc:Q9NXG0]	20	-1.959	-1.906	0.748
C9orf52	C9orf52 protein (Chromosome 9 open reading frame 52). [Source:Uniprot/SPTREMBL;Acc:Q8IXZ6]	18	-1.647	-1.829	0.729

Gene_Symbol	Gene_Description	AS_Exon	Splicing_index	Log_2_ratio	Correlation_Coefficent
C9orf93	Uncharacterized protein C9orf93. [Source:Uniprot/SWISSPROT;Acc:Q6TFL3]	5	-1.951	-1.947	0.876
CACHD1	cache domain containing 1 [Source:RefSeq_peptide;Acc:NP_065976]	20	-1.894	-2.037	0.918
CACNA1A	Voltage-dependent P/Q-type calcium channel subunit alpha-1A (Voltage- gated calcium channel subunit alpha Cav2.1) (Calcium channel, L type, alpha-1 polypeptide isoform 4) (Brain calcium channel I) (BI). [Source:Uniprot/SWISSPROT;Acc:O00555]	20	-1.799	-1.855	0.89
CACNA1B	Voltage-dependent N-type calcium channel subunit alpha-1B (Voltage- gated calcium channel subunit alpha Cav2.2) (Calcium channel, L type, alpha-1 polypeptide isoform 5) (Brain calcium channel III) (BIII). [Source:Uniprot/SWISSPROT;Acc:Q00975]	20	-1.652	-1.717	0.88
CACNA1C	Voltage-dependent L-type calcium channel subunit alpha-1C (Voltage- gated calcium channel subunit alpha Cav1.2) (Calcium channel, L type, alpha-1 polypeptide, isoform 1, cardiac muscle). [Source:Uniprot/SWISSPROT;Acc:Q13936]	20	-1.764	-1.798	0.891
CACNA1D	Voltage-dependent L-type calcium channel subunit alpha-1D (Voltage- gated calcium channel subunit alpha Cav1.3) (Calcium channel, L type, alpha-1 polypeptide, isoform 2). [Source:Uniprot/SWISSPROT;Acc:Q01668]	20	-1.874	-1.9	0.9
CACNA1E	Voltage-dependent R-type calcium channel subunit alpha-1E (Voltage- gated calcium channel subunit alpha Cav2.3) (Calcium channel, L type, alpha-1 polypeptide, isoform 6) (Brain calcium channel II) (BII). [Source:Uniprot/SWISSPROT;Acc:Q15878]	20	-1.766	-1.792	0.899
CACNA1F	Voltage-dependent L-type calcium channel subunit alpha-1F (Voltage- gated calcium channel subunit alpha Cav1.4). [Source:Uniprot/SWISSPROT;Acc:O60840]	20	-1.69	-1.808	0.851

Gene_Symbol	Gene_Description	AS_Exon	Splicing_index	Log_2_ratio	Correlation_Coefficent
CACNA1S	Voltage-dependent L-type calcium channel subunit alpha-1S (Voltage- gated calcium channel subunit alpha Cav1.1) (Calcium channel, L type, alpha-1 polypeptide, isoform 3, skeletal muscle). [Source:Uniprot/SWISSPROT;Acc:Q13698]	20	-1.805	-1.839	0.893
CACNA2D1	Dihydropyridine-sensitive L-type calcium channel subunits alpha- 2/delta precursor [Contains: L-type calcium channel subunit alpha-2; L-type calcium channel subunit delta]. [Source:Uniprot/SWISSPROT;Acc:P54289]	20	-1.842	-1.816	0.905
CACNB2	Voltage-dependent L-type calcium channel subunit beta-2 (CAB2) (Calcium channel voltage-dependent subunit beta 2) (Lambert-Eaton myasthenic syndrome antigen B) (MYSB). [Source:Uniprot/SWISSPROT;Acc:Q08289]	20	-2.255	-2.135	0.921
CAD	CAD protein [Includes: Glutamine-dependent carbamoyl-phosphate synthase (EC 6.3.5.5); Aspartate carbamoyltransferase (EC 2.1.3.2); Dihydroorotase (EC 3.5.2.3)]. [Source:Uniprot/SWISSPROT;Acc:P27708]	20	-1.685	-1.713	0.899
CAMK2G	Calcium/calmodulin-dependent protein kinase type II gamma chain (EC 2.7.11.17) (CaM-kinase II gamma chain) (CaM kinase II gamma subunit) (CaMK-II subunit gamma). [Source:Uniprot/SWISSPROT;Acc:Q13555]	20	-1.656	-1.672	0.851
CARD14	Caspase recruitment domain-containing protein 14 (CARD-containing MAGUK protein 2) (Carma 2). [Source:Uniprot/SWISSPROT;Acc:Q9BXL6]	20	-1.983	-2.073	0.843
CASKIN1	Caskin-1 (CASK-interacting protein 1). [Source:Uniprot/SWISSPROT;Acc:Q8WXD9]	20	-1.815	-2.072	0.923
CASKIN2	Caskin-2. [Source:Uniprot/SWISSPROT;Acc:Q8WXE0]	20	-1.853	-2.095	0.916
CASZ1	Castor homolog 1 zinc finger protein (Castor-related protein) (Zinc finger protein 693). [Source:Uniprot/SWISSPROT;Acc:Q86V15]	20	-1.852	-1.95	0.74

Gene_Symbol	Gene_Description	AS_Exon	Splicing_index	Log_2_ratio	Correlation_Coefficent
CC2D1A	Coiled-coil and C2 domain-containing protein 1A (Five repressor element under dual repression-binding protein 1) (FRE under dual repression-binding protein 1) (Freud-1) (Putative NF-kappa-B- activating protein 023N). [Source:Uniprot/SWISSPROT;Acc:Q6P1N0]	20	-2.012	-2.013	0.777
CCAR1	Cell division cycle and apoptosis regulator protein 1 (Cell cycle and apoptosis regulatory protein 1) (CARP-1) (Death inducer with SAP domain). [Source:Uniprot/SWISSPROT;Acc:Q8IX12]	20	-1.784	-1.732	0.748
CCDC40	CCDC40 protein. [Source:Uniprot/SPTREMBL;Acc:Q6PE47]	20	-2.036	-2.135	0.769
CCDC46	Coiled-coil domain-containing protein 46. [Source:Uniprot/SWISSPROT;Acc:Q8N8E3]	5	-1.994	-1.82	0.904
CD2BP2	CD2 antigen cytoplasmic tail-binding protein 2 (CD2 cytoplasmic domain-binding protein) (CD2 tail-binding protein). [Source:Uniprot/SWISSPROT;Acc:O95400]	6	-2.716	-2.419	0.972
CDC42BPG	Serine/threonine-protein kinase MRCK gamma (EC 2.7.11.1) (CDC42- binding protein kinase gamma) (Myotonic dystrophy kinase-related CDC42-binding kinase gamma) (Myotonic dystrophy protein kinase-like alpha) (MRCK gamma) (DMPK-like gamma). [Source:Uniprot/SWISSPROT;Acc:Q6DT37]	20	-1.647	-1.796	0.78
CDH23	Cadherin-23 precursor (Otocadherin). [Source:Uniprot/SWISSPROT;Acc:Q9H251]	61	-2.895	-3.109	0.945
CDH5	Cadherin-5 precursor (Vascular endothelial-cadherin) (VE-cadherin) (7B4 antigen) (CD144 antigen). [Source:Uniprot/SWISSPROT;Acc:P33151]	9	-3.071	-2.874	0.968
CDK5RAP2	CDK5 regulatory subunit-associated protein 2 (CDK5 activator-binding protein C48) (Centrosome-associated protein 215). [Source:Uniprot/SWISSPROT;Acc:Q96SN8]	20	-1.741	-1.828	0.778
CDKL5	Cyclin-dependent kinase-like 5 (EC 2.7.11.22) (Serine/threonine- protein kinase 9). [Source:Uniprot/SWISSPROT;Acc:O76039]	20	-1.906	-1.945	0.919

Gene_Symbol	Gene_Description	AS_Exon	Splicing_index	Log_2_ratio	Correlation_Coefficent
CDON	Cell adhesion molecule-related/down-regulated by oncogenes precursor. [Source:Uniprot/SWISSPROT;Acc:Q4KMG0]	20	-1.731	-1.729	0.806
CENPE	centromere protein E [Source:RefSeq_peptide;Acc:NP_001804]	28	-2.116	-2.192	0.841
CENTD3	Centaurin-delta 3 (Cnt-d3) (Arf-GAP, Rho-GAP, ankyrin repeat and pleckstrin homology domain-containing protein 3). [Source:Uniprot/SWISSPROT;Acc:Q8WWN8]	1	-1.706	-1.893	0.967
CEP192	centrosomal protein 192kDa [Source:RefSeq_peptide;Acc:NP_115518]	20	-1.761	-1.821	0.744
CEP250	Centrosome-associated protein CEP250 (Centrosomal protein 2) (Centrosomal Nek2-associated protein 1) (C-Nap1). [Source:Uniprot/SWISSPROT;Acc:Q9BV73]	28	-1.897	-2.032	0.76
CGN	Cingulin. [Source:Uniprot/SWISSPROT;Acc:Q9P2M7]	20	-1.807	-2.034	0.927
CHD8	Chromodomain-helicase-DNA-binding protein 8 (EC 3.6.1.-) (ATP- dependent helicase CHD8) (CHD-8) (Helicase with SNF2 domain 1). [Source:Uniprot/SWISSPROT;Acc:Q9HCK8]	20	-1.736	-1.908	0.928
CHL1	Neural cell adhesion molecule L1-like protein precursor (Close homolog of L1). [Source:Uniprot/SWISSPROT;Acc:O00533]	28	-2.437	-2.506	0.725
CLCA1	chloride channel, calcium activated, family member 1 precursor [Source:RefSeq_peptide;Acc:NP_001276]	10	-1.63	-1.725	0.906
CLEC7A	C-type lectin domain family 7 member A (Dendritic cell-associated C- type lectin 1) (DC-associated C-type lectin 1) (Dectin-1) (Beta-glucan receptor) (C-type lectin superfamily member 12). [Source:Uniprot/SWISSPROT;Acc:Q9BXN2]	1	-1.664	-1.936	0.772
CLTC	Clathrin heavy chain 1 (CLH-17). [Source:Uniprot/SWISSPROT;Acc:Q00610]	28	-1.951	-1.892	0.886
CMYA3	cardiomyopathy associated 3 isoform 1 [Source:RefSeq_peptide;Acc:NP_689594]	10	-2.347	-2.631	0.982

Gene_Symbol	Gene_Description	AS_Exon	Splicing_index	Log_2_ratio	Correlation_Coefficent
CNGB1	Cyclic nucleotide-gated cation channel 4 (CNG channel 4) (CNG-4) (CNG4) (Cyclic nucleotide-gated cation channel modulatory subunit). [Source:Uniprot/SWISSPROT;Acc:Q14028]	28	-1.988	-2.216	0.725
CNTN2	Contactin-2 precursor (Axonin-1) (Axonal glycoprotein TAG-1) (Transient axonal glycoprotein 1) (TAX-1). [Source:Uniprot/SWISSPROT;Acc:Q02246]	20	-1.597	-1.671	0.92
CNTN3	Contactin-3 precursor (Brain-derived immunoglobulin superfamily protein 1) (BIG-1) (Plasmacytoma-associated neuronal glycoprotein). [Source:Uniprot/SWISSPROT;Acc:Q9P232]	20	-1.698	-1.731	0.837
CNTNAP5	contactin associated protein-like 5 isoform 1 [Source:RefSeq_peptide;Acc:NP_570129]	10	-2.241	-2.427	0.958
COG7	Conserved oligomeric Golgi complex component 7. [Source:Uniprot/SWISSPROT;Acc:P83436]	10	-3.154	-3.217	0.819
COL11A2	Collagen alpha-2(XI) chain precursor. [Source:Uniprot/SWISSPROT;Acc:P13942]	28	-1.749	-2.155	0.731
COL11A2	Collagen alpha-2(XI) chain precursor. [Source:Uniprot/SWISSPROT;Acc:P13942]	10	-1.678	-2.085	0.731
COL12A1	Collagen alpha-1(XII) chain precursor. [Source:Uniprot/SWISSPROT;Acc:Q99715]	28	-1.933	-2.179	0.706
COL12A1	Collagen alpha-1(XII) chain precursor. [Source:Uniprot/SWISSPROT;Acc:Q99715]	10	-1.841	-2.086	0.706
COL16A1	Collagen alpha-1(XVI) chain precursor. [Source:Uniprot/SWISSPROT;Acc:Q07092]	10	-1.873	-2.229	0.777
COL16A1	Collagen alpha-1(XVI) chain precursor. [Source:Uniprot/SWISSPROT;Acc:Q07092]	28	-1.837	-2.193	0.777
COL17A1	Collagen alpha-1(XVII) chain (Bullous pemphigoid antigen 2) (180 kDa bullous pemphigoid antigen 2). [Source:Uniprot/SWISSPROT;Acc:Q9UMD9]	10	-1.858	-2.28	0.922
COL17A1	Collagen alpha-1(XVII) chain (Bullous pemphigoid antigen 2) (180 kDa bullous pemphigoid antigen 2). [Source:Uniprot/SWISSPROT;Acc:Q9UMD9]	28	-1.764	-2.186	0.922
COL1A1	Collagen alpha-1(I) chain precursor. [Source:Uniprot/SWISSPROT;Acc:P02452]	28	-2.136	-2.44	0.773

Gene_Symbol	Gene_Description	AS_Exon	Splicing_index	Log_2_ratio	Correlation_Coefficent
COL1A2	Collagen alpha-2(I) chain precursor. [Source:Uniprot/SWISSPROT;Acc:P08123]	28	-2.209	-2.409	0.796
COL20A1	Collagen alpha-1(XX) chain precursor. [Source:Uniprot/SWISSPROT;Acc:Q9P218]	20	-1.832	-2.097	0.919
COL22A1	collagen, type XXII, alpha 1 [Source:RefSeq_peptide;Acc:NP_690848]	35	-1.629	-1.697	0.882
COL2A1	Collagen alpha-1(II) chain precursor [Contains: Chondrocalcin]. [Source:Uniprot/SWISSPROT;Acc:P02458]	28	-2.073	-2.306	0.803
COL3A1	Collagen alpha-1(III) chain precursor. [Source:Uniprot/SWISSPROT;Acc:P02461]	28	-2.233	-2.475	0.722
COL4A1	Collagen alpha-1(IV) chain precursor (Arresten). [Source:Uniprot/SWISSPROT;Acc:P02462]	28	-2.16	-2.443	0.739
COL4A2	Collagen alpha-2(IV) chain precursor [Contains: Canstatin]. [Source:Uniprot/SWISSPROT;Acc:P08572]	28	-2.21	-2.484	0.706
COL4A3	Collagen alpha-3(IV) chain precursor (Goodpasture antigen) [Contains: Tumstatin]. [Source:Uniprot/SWISSPROT;Acc:Q01955]	28	-2.253	-2.515	0.735
COL4A4	Collagen alpha-4(IV) chain precursor. [Source:Uniprot/SWISSPROT;Acc:P53420]	28	-2.24	-2.511	0.723
COL4A5	Collagen alpha-5(IV) chain precursor. [Source:Uniprot/SWISSPROT;Acc:P29400]	28	-2.101	-2.362	0.702
COL5A1	Collagen alpha-1(V) chain precursor. [Source:Uniprot/SWISSPROT;Acc:P20908]	28	-1.998	-2.396	0.754
COL5A2	Collagen alpha-2(V) chain precursor. [Source:Uniprot/SWISSPROT;Acc:P05997]	28	-2.067	-2.25	0.735
COL7A1	Collagen alpha-1(VII) chain precursor (Long-chain collagen) (LC collagen). [Source:Uniprot/SWISSPROT;Acc:Q02388]	28	-1.696	-2.03	0.863
COL9A1	Collagen alpha-1(IX) chain precursor. [Source:Uniprot/SWISSPROT;Acc:P20849]	28	-1.822	-2.204	0.714
COL9A1	Collagen alpha-1(IX) chain precursor. [Source:Uniprot/SWISSPROT;Acc:P20849]	10	-1.945	-2.327	0.714
COL9A2	Collagen alpha-2(IX) chain precursor. [Source:Uniprot/SWISSPROT;Acc:Q14055]	28	-1.707	-2.089	0.717

Gene_Symbol	Gene_Description	AS_Exon	Splicing_index	Log_2_ratio	Correlation_Coefficent
COL9A2	Collagen alpha-2(IX) chain precursor. [Source:Uniprot/SWISSPROT;Acc:Q14055]	10	-1.652	-2.034	0.717
COMP	Cartilage oligomeric matrix protein precursor (COMP). [Source:Uniprot/SWISSPROT;Acc:P49747]	10	-1.674	-1.801	0.934
CORO7	Coronin-7 (70 kDa WD repeat tumor rejection antigen homolog). [Source:Uniprot/SWISSPROT;Acc:P57737]	20	-1.991	-2.08	0.858
CPNE4	Copine-4 (Copine IV) (Copine-8). [Source:Uniprot/SWISSPROT;Acc:Q96A23]	10	-1.978	-2.193	0.966
CPNE5	Copine-5 (Copine V). [Source:Uniprot/SWISSPROT;Acc:Q9HCH3]	20	-1.922	-2.147	0.914
CRHR2	Corticotropin-releasing factor receptor 2 precursor (CRF-R 2) (CRF2) (Corticotropin-releasing hormone receptor 2) (CRH-R 2). [Source:Uniprot/SWISSPROT;Acc:Q13324]	10	-1.905	-2.146	0.971
CSMD2	CUB and sushi domain-containing protein 2 (CUB and sushi multiple domains protein 2). [Source:Uniprot/SWISSPROT;Acc:Q7Z408]	63	-1.712	-1.96	0.833
CSMD2	CUB and sushi domain-containing protein 2 (CUB and sushi multiple domains protein 2). [Source:Uniprot/SWISSPROT;Acc:Q7Z408]	28	-2.173	-2.42	0.833
CSMD3	CUB and sushi domain-containing protein 3 precursor (CUB and sushi multiple domains protein 3). [Source:Uniprot/SWISSPROT;Acc:Q7Z407]	28	-2.233	-2.464	0.826
CTCFL	Transcriptional repressor CTCFL (CCCTC-binding factor) (Brother of the regulator of imprinted sites) (Zinc finger protein CTCF-T) (CTCF paralog). [Source:Uniprot/SWISSPROT;Acc:Q8NI51]	10	-1.586	-2.01	0.939
CTPS2	CTP synthase 2 (EC 6.3.4.2) (UTP--ammonia ligase 2) (CTP synthetase 2). [Source:Uniprot/SWISSPROT;Acc:Q9NRF8]	20	-1.717	-1.833	0.872
CTTNBP2	Cortactin-binding protein 2 (CortBP2). [Source:Uniprot/SWISSPROT;Acc:Q8WZ74]	20	-1.828	-1.879	0.8
CXorf20	Uncharacterized protein CXorf20. [Source:Uniprot/SWISSPROT;Acc:Q8NDZ0]	10	-1.77	-1.901	0.97

Gene_Symbol	Gene_Description	AS_Exon	Splicing_index	Log_2_ratio	Correlation_Coefficent
CXorf43	chromosome X open reading frame 43 (CXorf43), mRNA [Source:RefSeq_dna;Acc:NM_144657]	10	-1.74	-2.018	0.982
DCBLD2	Discoidin, CUB and LCCL domain-containing protein 2 precursor (Endothelial and smooth muscle cell-derived neuropilin-like protein) (CUB, LCCL and coagulation factor V/VIII-homology domains protein 1). [Source:Uniprot/SWISSPROT;Acc:Q96PD2]	10	-2.032	-2.059	0.976
DCHS2	dachsous 2 isoform 1 [Source:RefSeq_peptide;Acc:NP_060109]	20	-1.807	-1.88	0.765
DCLRE1C	Artemis protein (EC 3.1.-.-) (DNA cross-link repair 1C protein) (SNM1- like protein) (A-SCID protein) (hSNM1C). [Source:Uniprot/SWISSPROT;Acc:Q96SD1]	1	-1.691	-1.912	0.891
DDX21	Nucleolar RNA helicase 2 (EC 3.6.1.-) (Nucleolar RNA helicase II) (Nucleolar RNA helicase Gu) (RH II/Gu) (Gu-alpha) (DEAD box protein 21). [Source:Uniprot/SWISSPROT;Acc:Q9NR30]	8	-2.101	-2.133	0.826
DDX54	ATP-dependent RNA helicase DDX54 (EC 3.6.1.-) (DEAD box protein 54) (ATP-dependent RNA helicase DP97). [Source:Uniprot/SWISSPROT;Acc:Q8TDD1]	20	-1.706	-1.83	0.844
DENND1A	DENN domain-containing protein 1A. [Source:Uniprot/SWISSPROT;Acc:Q8TEH3]	20	-1.918	-2.11	0.92
DGKK	Diacylglycerol kinase kappa (EC 2.7.1.107) (Diglyceride kinase kappa) (DGK-kappa) (DAG kinase kappa) (142 kDa diacylglycerol kinase). [Source:Uniprot/SWISSPROT;Acc:Q5KSL6]	10	-1.81	-1.906	0.794
DHX35	Probable ATP-dependent RNA helicase DHX35 (EC 3.6.1.-) (DEAH box protein 35). [Source:Uniprot/SWISSPROT;Acc:Q9H5Z1]	20	-1.61	-1.685	0.853
DHX37	Probable ATP-dependent RNA helicase DHX37 (EC 3.6.1.-) (DEAH box protein 37). [Source:Uniprot/SWISSPROT;Acc:Q8IY37]	20	-1.721	-1.922	0.915
DIP13B		20	-1.951	-2.019	0.735
DIP2B	DIP2 disco-interacting protein 2 homolog B [Source:RefSeq_peptide;Acc:NP_775873]	20	-1.636	-1.827	0.928

Gene_Symbol	Gene_Description	AS_Exon	Splicing_index	Log_2_ratio	Correlation_Coefficent
DKFZp667G2110		10	-1.601	-1.744	0.945
DMD	Dystrophin. [Source:Uniprot/SWISSPROT;Acc:P11532]	44	-3.604	-3.616	0.922
DNAH1	dynein, axonemal, heavy polypeptide 1 [Source:RefSeq_peptide;Acc:NP_056327]	71	-2.605	-2.596	0.904
DNAH10	dynein, axonemal, heavy polypeptide 10 isoform 1 [Source:RefSeq_peptide;Acc:NP_001077369]	46	-3.605	-3.767	0.812
DNAH3	dynein, axonemal, heavy polypeptide 3 [Source:RefSeq_peptide;Acc:NP_060009]	20	-1.707	-1.802	0.804
DNAH5	Ciliary dynein heavy chain 5 (Axonemal beta dynein heavy chain 5) (HL1). [Source:Uniprot/SWISSPROT;Acc:Q8TE73]	63	-2.101	-2.133	0.937
DNAH7	axonemal dynein heavy chain 7 [Source:RefSeq_peptide;Acc:NP_061720]	20	-1.585	-1.782	0.815
DNAH7	axonemal dynein heavy chain 7 [Source:RefSeq_peptide;Acc:NP_061720]	61	-3.359	-3.556	0.815
DNAH8	Ciliary dynein heavy chain 8 (Axonemal beta dynein heavy chain 8). [Source:Uniprot/SWISSPROT;Acc:Q96JB1]	85	-4.506	-4.566	0.868
DNAJA5	DnaJ (Hsp40) homolog, subfamily C, member 21	10	-1.778	-1.912	0.924
DNHD2	CDNA FLJ40427 fis, clone TESTI2039113. [Source:Uniprot/SPTREMBL;Acc:Q8N7R9]	5	-2.05	-1.857	0.912
DPP10	Inactive dipeptidyl peptidase 10 (Dipeptidyl peptidase X) (Dipeptidyl peptidase-like protein 2) (DPL2) (Dipeptidyl peptidase IV-related protein 3) (DPRP-3). [Source:Uniprot/SWISSPROT;Acc:Q8N608]	20	-1.886	-2.114	0.92
DPP8	Dipeptidyl peptidase 8 (EC 3.4.14.5) (Dipeptidyl peptidase VIII) (DP8) (Prolyl dipeptidase DPP8) (Dipeptidyl peptidase IV-related protein 1) (DPRP-1). [Source:Uniprot/SWISSPROT;Acc:Q6V1X1]	20	-1.908	-1.934	0.74
DRB1	MHC class I antigen DRB1*11	10	-2.267	-2.543	0.987
DSCAML1	Down syndrome cell adhesion molecule-like protein 1 precursor (Down syndrome cell adhesion molecule 2). [Source:Uniprot/SWISSPROT;Acc:Q8TD84]	20	-2.072	-2.165	0.89

Gene_Symbol	Gene_Description	AS_Exon	Splicing_index	Log_2_ratio	Correlation_Coefficent
DST	Bullous pemphigoid antigen 1, isoforms 1/2/3/4/5/8 (230 kDa bullous pemphigoid antigen) (BPA) (Hemidesmosomal plaque protein) (Dystonia musculorum protein) (Dystonin) (Fragment). [Source:Uniprot/SWISSPROT;Acc:Q03001]	61	-3.036	-3.104	0.96
DST	Bullous pemphigoid antigen 1, isoforms 1/2/3/4/5/8 (230 kDa bullous pemphigoid antigen) (BPA) (Hemidesmosomal plaque protein) (Dystonia musculorum protein) (Dystonin) (Fragment). [Source:Uniprot/SWISSPROT;Acc:Q03001]	20	-1.794	-1.862	0.96
DUOX2	Dual oxidase 2 precursor (EC 1.6.3.1) (EC 1.11.1.-) (NADPH oxidase/peroxidase DUOX2) (NADPH thyroid oxidase 2) (Thyroid oxidase 2) (NADH/NADPH thyroid oxidase p138-tox) (p138 thyroid oxidase) (Large NOX 2) (Long NOX 2). [Source:Uniprot/SWISSPROT;Acc:Q9NRD8]	20	-1.697	-1.718	0.9
EMR1	EGF-like module-containing mucin-like hormone receptor-like 1 precursor (Cell surface glycoprotein EMR1) (EMR1 hormone receptor). [Source:Uniprot/SWISSPROT;Acc:Q14246]	5	-2.02	-1.932	0.887
ENG	Endoglin precursor (CD105 antigen). [Source:Uniprot/SWISSPROT;Acc:P17813]	5	-1.894	-1.892	0.783
ENO2	Gamma-enolase (EC 4.2.1.11) (2-phospho-D-glycerate hydro-lyase) (Neural enolase) (Neuron-specific enolase) (NSE) (Enolase 2). [Source:Uniprot/SWISSPROT;Acc:P09104]	5	-1.639	-1.845	0.961
ENPEP	Glutamyl aminopeptidase (EC 3.4.11.7) (EAP) (Aminopeptidase A) (APA) (Differentiation antigen gp160) (CD249 antigen). [Source:Uniprot/SWISSPROT;Acc:Q07075]	5	-1.884	-1.776	0.867
EP300	Histone acetyltransferase p300 (EC 2.3.1.48) (E1A-associated protein p300). [Source:Uniprot/SWISSPROT;Acc:Q09472]	5	-1.781	-1.659	0.835

Gene_Symbol	Gene_Description	AS_Exon	Splicing_index	Log_2_ratio	Correlation_Coefficent
EP400	E1A-binding protein p400 (EC 3.6.1.-) (p400 kDa SWI2/SNF2-related protein) (Domino homolog) (hDomino) (CAG repeat protein 32) (Trinucleotide repeat-containing gene 12 protein). [Source:Uniprot/SWISSPROT;Acc:Q96L91]	20	-1.748	-2.056	0.906
EP400	E1A-binding protein p400 (EC 3.6.1.-) (p400 kDa SWI2/SNF2-related protein) (Domino homolog) (hDomino) (CAG repeat protein 32) (Trinucleotide repeat-containing gene 12 protein). [Source:Uniprot/SWISSPROT;Acc:Q96L91]	49	-2.539	-2.848	0.906
EPS8L1	Epidermal growth factor receptor kinase substrate 8-like protein 1 (Epidermal growth factor receptor pathway substrate 8-related protein 1) (EPS8-like protein 1). [Source:Uniprot/SWISSPROT;Acc:Q8TE68]	20	-1.708	-1.811	0.781
EXOC2	Exocyst complex component 2 (Exocyst complex component Sec5). [Source:Uniprot/SWISSPROT;Acc:Q96KP1]	20	-1.813	-1.875	0.715
FAM40B	Protein FAM40B. [Source:Uniprot/SWISSPROT;Acc:Q9ULQ0]	20	-1.988	-2.192	0.923
FAM48A	Protein FAM48A (p38-interacting protein) (p38IP). [Source:Uniprot/SWISSPROT;Acc:Q8NEM7]	20	-1.706	-1.663	0.758
FAM62B	Protein FAM62B (Extended synaptotagmin-2) (Chr2Syt). [Source:Uniprot/SWISSPROT;Acc:A0FGR8]	20	-1.777	-2.006	0.938
FAM65A	family with sequence similarity 65, member A (FAM65A), mRNA [Source:RefSeq_dna;Acc:NM_024519]	20	-1.737	-1.921	0.88
FANCM	Fanconi anemia group M protein (EC 3.6.1.-) (ATP-dependent RNA helicase FANCM) (Protein FACM) (Fanconi anemia-associated polypeptide of 250 kDa) (FAAP250) (Protein Hef ortholog). [Source:Uniprot/SWISSPROT;Acc:Q8IYD8]	20	-1.827	-2.025	0.929
FGD2	FYVE, RhoGEF and PH domain-containing protein 2 (Zinc finger FYVE domain-containing protein 4). [Source:Uniprot/SWISSPROT;Acc:Q7Z6J4]	1	-3.668	-3.647	0.833

Gene_Symbol	Gene_Description	AS_Exon	Splicing_index	Log_2_ratio	Correlation_Coefficent
FLJ10357	hypothetical protein **FLJ10357**	20	-1.627	-1.713	0.73
FLJ13231	hypothetical protein FLJ13231	20	-1.667	-1.779	0.918
FLJ20035	hypothetical protein FLJ20035	20	-1.619	-1.686	0.767
FLJ20433	hypothetical protein FLJ20433	20	-1.645	-1.761	0.731
FLJ31438	hypothetical protein FLJ31438	10	-2.223	-2.469	0.97
FLJ32310	hypothetical protein FLJ32310	10	-1.824	-1.865	0.912
FLJ36748	hypothetical protein FLJ36748	10	-1.62	-1.776	0.926
FLJ38020	hypothetical protein FLJ38020	1	-1.597	-1.819	0.888
FMNL1	Formin-like protein 1 (Leukocyte formin) (CLL-associated antigen KW- 13). [Source:Uniprot/SWISSPROT;Acc:O95466]	20	-1.908	-1.832	0.931
FRMD4A	FERM domain-containing protein 4A. [Source:Uniprot/SWISSPROT;Acc:Q9P2Q2]	20	-1.718	-1.854	0.784
FTS	Fused toes protein homolog	1	-1.598	-1.838	0.881
GNPTAB	N-acetylglucosamine-1-phosphotransferase subunits alpha/beta precursor (EC 2.7.8.17) (GlcNAc-1-phosphotransferase alpha/beta subunits) (UDP- N-acetylglucosamine-1-phosphotransferase alpha/beta subunits) (Stealth protein GNPTAB) [Contains: N-acetylglucosam [Source:Uniprot/SWISSPROT;Acc:Q3T906]	20	-1.85	-1.933	0.855
GON4L	GON-4-like protein (GON-4 homolog). [Source:Uniprot/SWISSPROT;Acc:Q3T8J9]	20	-1.84	-1.803	0.851
GPAM	Glycerol-3-phosphate acyltransferase, mitochondrial precursor (EC 2.3.1.15) (GPAT). [Source:Uniprot/SWISSPROT;Acc:Q9HCL2]	20	-1.836	-2.146	0.902
GPR98	G-protein coupled receptor 98 precursor (Monogenic audiogenic seizure susceptibility protein 1 homolog) (Very large G-protein coupled receptor 1) (Usher syndrome type-2C protein). [Source:Uniprot/SWISSPROT;Acc:Q8WXG9]	20	-1.748	-1.839	0.914
GRAMD1A	GRAM domain containing 1A [Source:RefSeq_peptide;Acc:NP_065946]	20	-1.712	-1.85	0.884
GRAMD1B	GRAM domain containing 1B [Source:RefSeq_peptide;Acc:NP_065767]	20	-1.881	-2.061	0.923

Gene_Symbol	Gene_Description	AS_Exon	Splicing_index	Log_2_ratio	Correlation_Coefficent
HACE1	E3 ubiquitin-protein ligase HACE1 (EC 6.3.2.-) (HECT domain and ankyrin repeat-containing E3 ubiquitin-protein ligase 1). [Source:Uniprot/SWISSPROT;Acc:Q8IYU2]	20	-1.8	-2.058	0.937
HDAC10	Histone deacetylase 10 (HD10). [Source:Uniprot/SWISSPROT;Acc:Q969S8]	20	-1.636	-1.77	0.825
HEATR1	HEAT repeat-containing protein 1 (Protein BAP28). [Source:Uniprot/SWISSPROT;Acc:Q9H583]	20	-1.919	-1.856	0.759
HECW2	E3 ubiquitin-protein ligase HECW2 (EC 6.3.2.-) (HECT, C2 and WW domain-containing protein 2) (NEDD4-like ubiquitin-protein ligase 2). [Source:Uniprot/SWISSPROT;Acc:Q9P2P5]	20	-1.845	-2.122	0.898
HECW2	E3 ubiquitin-protein ligase HECW2 (EC 6.3.2.-) (HECT, C2 and WW domain-containing protein 2) (NEDD4-like ubiquitin-protein ligase 2). [Source:Uniprot/SWISSPROT;Acc:Q9P2P5]	3	-1.634	-1.91	0.898
HERC6	Probable E3 ubiquitin-protein ligase HERC6 (EC 6.3.2.-) (HECT domain and RCC1-like domain-containing protein 6). [Source:Uniprot/SWISSPROT;Acc:Q8IVU3]	20	-1.713	-1.812	0.801
HHIP	Hedgehog-interacting protein precursor (HHIP) (HIP). [Source:Uniprot/SWISSPROT;Acc:Q96QV1]	1	-1.625	-1.86	0.893
HSPG2	Basement membrane-specific heparan sulfate proteoglycan core protein precursor (HSPG) (Perlecan) (PLC). [Source:Uniprot/SWISSPROT;Acc:P98160]	4	-2.478	-2.608	0.92
HYDIN	Hydrocephalus-inducing protein homolog. [Source:Uniprot/SWISSPROT;Acc:Q4G0P3]	20	-1.685	-1.809	0.91
ICA1L	Islet cell autoantigen 1-like protein (Amyotrophic lateral sclerosis 2 chromosomal region candidate gene 15 protein). [Source:Uniprot/SWISSPROT;Acc:Q8NDH6]	10	-1.97	-2.17	0.963
IFT122	Intraflagellar transport 122 homolog (WD repeat protein 10). [Source:Uniprot/SWISSPROT;Acc:Q9HBG6]	20	-1.83	-1.84	0.725

Gene_Symbol	Gene_Description	AS_Exon	Splicing_index	Log_2_ratio	Correlation_Coefficent
IL31RA	Interleukin-31 receptor A precursor (IL-31RA) (Cytokine receptor-like 3) (Gp130-like monocyte receptor) (GLM-R) (HGLM-R) (Gp130-like receptor). [Source:Uniprot/SWISSPROT;Acc:Q8NI17]	10	-1.835	-1.992	0.95
INADL	InaD-like protein (Inadl protein) (hINADL) (Pals1-associated tight junction protein) (Protein associated to tight junctions). [Source:Uniprot/SWISSPROT;Acc:Q8NI35]	28	-1.854	-1.892	0.716
INOC1	Putative DNA helicase INO80 complex homolog 1 (EC 3.6.1.-) (hINO80). [Source:Uniprot/SWISSPROT;Acc:Q9ULG1]	20	-1.657	-1.688	0.836
INTS2	Integrator complex subunit 2 (Int2). [Source:Uniprot/SWISSPROT;Acc:Q9H0H0]	20	-1.791	-1.989	0.942
IPO11	Importin-11 (Imp11) (Ran-binding protein 11) (RanBP11). [Source:Uniprot/SWISSPROT;Acc:Q9UI26]	20	-1.594	-1.586	0.798
ITSN2	Intersectin-2 (SH3 domain-containing protein 1B) (SH3P18) (SH3P18-like WASP-associated protein). [Source:Uniprot/SWISSPROT;Acc:Q9NZM3]	20	-1.601	-1.678	0.883
KHDRBS2	KH domain-containing, RNA-binding, signal transduction-associated protein 2 [Source:RefSeq_peptide;Acc:NP_689901]	5	-1.635	-1.894	0.972
KIAA1109	CDNA: FLJ21404 fis, clone COL03835. [Source:Uniprot/SPTREMBL;Acc:Q9H742]	20	-1.752	-1.811	0.887
KIAA1217	KIAA1217 (KIAA1217), mRNA [Source:RefSeq_dna;Acc:NM_019590]	20	-1.617	-1.753	0.832
KIAA1244	KIAA1244 (KIAA1244), mRNA [Source:RefSeq_dna;Acc:NM_020340]	20	-2.027	-2.159	0.928
KIAA1303	KIAA1303	20	-1.949	-2.117	0.936
KIAA1324	KIAA1324 (KIAA1324), mRNA [Source:RefSeq_dna;Acc:NM_020775]	20	-1.884	-2.133	0.936
KIAA1344	Thioredoxin domain-containing protein KIAA1344 precursor. [Source:Uniprot/SWISSPROT;Acc:Q9P2K2]	20	-1.928	-2.152	0.919
KIAA1345	KIAA1345	20	-1.884	-2.101	0.937

Gene_Symbol	Gene_Description	AS_Exon	Splicing_index	Log_2_ratio	Correlation_Coefficent
KIAA1432	KIAA1432 (KIAA1432), mRNA [Source:RefSeq_dna;Acc:NM_020829]	20	-1.622	-1.826	0.943
KIAA1468	KIAA1468 (KIAA1468), mRNA [Source:RefSeq_dna;Acc:NM_020854]	20	-1.802	-1.977	0.919
KIAA1524	p90 autoantigen [Source:RefSeq_peptide;Acc:NP_065941]	20	-1.7	-1.785	0.888
KIAA1604	KIAA1604	20	-2.008	-2.077	0.904
KIAA1622	HEAT-like repeat-containing protein isoform 1 [Source:RefSeq_peptide;Acc:NP_478144]	20	-1.863	-2.044	0.922
KIAA1797	KIAA1797 (KIAA1797), mRNA [Source:RefSeq_dna;Acc:NM_017794]	20	-1.915	-2.013	0.795
KIAA1967	p30 DBC protein [Source:RefSeq_peptide;Acc:NP_066997]	20	-1.722	-1.813	0.913
KIDINS220	kinase D-interacting substrate of 220 kDa	20	-1.723	-1.901	0.944
KIF13B	Kinesin-like protein KIF13B (Kinesin-like protein GAKIN). [Source:Uniprot/SWISSPROT;Acc:Q9NQT8]	14	-1.638	-1.588	0.933
KIF1A	Kinesin-like protein KIF1A (Axonal transporter of synaptic vesicles) (Microtubule-based motor KIF1A) (UNC-104-and KIF1A-related protein) (hUNC-104). [Source:Uniprot/SWISSPROT;Acc:Q12756]	20	-1.76	-1.921	0.854
KTN1	Kinectin (Kinesin receptor) (CG-1 antigen). [Source:Uniprot/SWISSPROT;Acc:Q86UP2]	4	-1.609	-1.778	0.731
L1CAM	Neural cell adhesion molecule L1 precursor (N-CAM L1) (CD171 antigen). [Source:Uniprot/SWISSPROT;Acc:P32004]	4	-1.656	-2.008	0.713
LARP2	La ribonucleoprotein domain family member 2 isoform 1 [Source:RefSeq_peptide;Acc:NP_060548]	20	-2.07	-2.073	0.703
LOC129881	hypothetical protein **LOC129881**	5	-1.752	-2.065	0.983
LOC130940	hypothetical protein LOC130940	10	-2.264	-2.471	0.97
LOC130951	hypothetical protein LOC130951	10	-1.936	-2.161	0.97
LOC133308	hypothetical protein LOC133308	10	-1.638	-1.623	0.952
LOC202134	hypothetical protein LOC202134	5	-1.954	-1.861	0.902
LOC728343	hypothetical protein LOC728343	66	-2.281	-2.416	0.853

Gene_Symbol	Gene_Description	AS_Exon	Splicing_index	Log_2_ratio	Correlation_Coefficent
LOC728343	hypothetical protein LOC728343	61	-3.485	-3.619	0.853
LPIN3	Lipin-3 (Lipin 3-like). [Source:Uniprot/SWISSPROT;Acc:Q9BQK8]	20	-1.844	-2.05	0.944
LRCH2	Leucine-rich repeat and calponin homology domain-containing protein 2. [Source:Uniprot/SWISSPROT;Acc:Q5VUJ6]	20	-1.91	-2.124	0.893
LRP1	Low-density lipoprotein receptor-related protein 1 precursor (LRP) (Alpha-2-macroglobulin receptor) (A2MR) (Apolipoprotein E receptor) (APOER) (CD91 antigen). [Source:Uniprot/SWISSPROT;Acc:Q07954]	4	-3.449	-3.582	0.794
LRRC16	Leucine-rich repeat-containing protein 16. [Source:Uniprot/SPTREMBL;Acc:Q5VZK9]	20	-1.73	-1.757	0.753
LRRC45	Leucine-rich repeat-containing protein 45. [Source:Uniprot/SWISSPROT;Acc:Q96CN5]	5	-1.895	-1.764	0.908
LRRIQ1	leucine-rich repeats and IQ motif containing 1 isoform 2 [Source:RefSeq_peptide;Acc:NP_115541]	20	-1.842	-1.92	0.812
LRRK2	Leucine-rich repeat serine/threonine-protein kinase 2 (EC 2.7.11.1) (Dardarin). [Source:Uniprot/SWISSPROT;Acc:Q5S007]	28	-2.423	-2.524	0.854
LYST	Lysosomal-trafficking regulator (Beige homolog). [Source:Uniprot/SWISSPROT;Acc:Q99698]	28	-2.282	-2.375	0.993
MAGEC3	Melanoma-associated antigen C3 (MAGE-C3 antigen) (Hepatocellular carcinoma-associated antigen 2). [Source:Uniprot/SWISSPROT;Acc:Q8TD91]	10	-1.666	-1.899	0.976
MAMDC4	apical early endosomal glycoprotein precursor [Source:RefSeq_peptide;Acc:NP_996803]	18	-1.674	-1.717	0.909
MAP4K1	Mitogen-activated protein kinase kinase kinase kinase 1 (EC 2.7.11.1) (MAPK/ERK kinase kinase kinase 1) (MEK kinase kinase 1) (MEKKK 1) (Hematopoietic progenitor kinase). [Source:Uniprot/SWISSPROT;Acc:Q92918]	28	-1.931	-2.062	0.763

Gene_Symbol	Gene_Description	AS_Exon	Splicing_index	Log_2_ratio	Correlation_Coefficent
MARK2	Serine/threonine-protein kinase MARK2 (EC 2.7.11.1) (MAP/microtubule affinity-regulating kinase 2) (ELKL motif kinase) (EMK1) (PAR1 homolog). [Source:Uniprot/SWISSPROT;Acc:Q7KZI7]	5	-1.858	-1.631	0.913
MCTP2	multiple C2-domains with two transmembrane regions 2 [Source:RefSeq_peptide;Acc:NP_060819]	20	-1.785	-1.942	0.719
MDH1B	malate dehydrogenase 1B, NAD (soluble) [Source:RefSeq_peptide;Acc:NP_001034934]	10	-1.95	-2.159	0.973
MIB1	E3 ubiquitin-protein ligase MIB1 (EC 6.3.2.-) (Mind bomb homolog 1) (DAPK-interacting protein 1) (DIP-1) (Zinc finger ZZ type with ankyrin repeat domain protein 2). [Source:Uniprot/SWISSPROT;Acc:Q86YT6]	20	-1.852	-2.043	0.924
MINK1	Misshapen-like kinase 1 (EC 2.7.11.1) (Mitogen-activated protein kinase kinase kinase kinase 6) (MAPK/ERK kinase kinase kinase 6) (MEK kinase kinase 6) (MEKKK 6) (Misshapen/NIK-related kinase) (GCK family kinase MiNK). [Source:Uniprot/SWISSPROT;Acc:Q8N4C8]	20	-1.658	-1.634	0.892
MLL3	Myeloid/lymphoid or mixed-lineage leukemia protein 3 homolog (EC 2.1.1.43) (Histone-lysine N-methyltransferase, H3 lysine-4 specific MLL3) (Homologous to ALR protein). [Source:Uniprot/SWISSPROT;Acc:Q8NEZ4]	20	-1.859	-1.905	0.866
MLLT6	Protein AF-17. [Source:Uniprot/SWISSPROT;Acc:P55198]	20	-1.896	-1.885	0.752
MMEL1	Membrane metallo-endopeptidase-like 1 (EC 3.4.24.11) (Membrane metallo-endopeptidase-like 2) (Neprilysin-2) (Neprilysin II) (NL2) (NEPII) (NEP2(m)) [Contains: Membrane metallo-endopeptidase-like 1, soluble form (Neprilysin-2 secreted) (NEP2(s))]. [Source:Uniprot/SWISSPROT;Acc:Q495T6]	20	-2.088	-2.187	0.858
MPP4	MAGUK p55 subfamily member 4 (Discs large homolog 6) (Amyotrophic lateral sclerosis 2 chromosomal region candidate gene 5 protein). [Source:Uniprot/SWISSPROT;Acc:Q96JB8]	20	-1.749	-1.907	0.85

Gene_Symbol	Gene_Description	AS_Exon	Splicing_index	Log_2_ratio	Correlation_Coefficent
MST1R	Macrophage-stimulating protein receptor precursor (EC 2.7.10.1) (MSP receptor) (p185-Ron) (CD136 antigen) (CDw136) [Contains: Macrophage-stimulating protein receptor alpha chain; Macrophage-stimulating protein receptor beta chain]. [Source:Uniprot/SWISSPROT;Acc:Q04912]	20	-1.998	-2.074	0.802
MTHFD1	C-1-tetrahydrofolate synthase, cytoplasmic (C1-THF synthase) [Includes: Methylenetetrahydrofolate dehydrogenase (EC 1.5.1.5); Methenyltetrahydrofolate cyclohydrolase (EC 3.5.4.9); Formyltetrahydrofolate synthetase (EC 6.3.4.3)]. [Source:Uniprot/SWISSPROT;Acc:P11586]	20	-1.777	-1.814	0.836
MTR	Methionine synthase (EC 2.1.1.13) (5-methyltetrahydrofolate-- homocysteine methyltransferase) (Methionine synthase, vitamin-B12 dependent) (MS). [Source:Uniprot/SWISSPROT;Acc:Q99707]	20	-1.849	-1.997	0.888
MUC2	Mucin-2 precursor (Intestinal mucin-2). [Source:Uniprot/SWISSPROT;Acc:Q02817]	20	-1.941	-2.092	0.841
MUC4	Mucin-4 precursor (Pancreatic adenocarcinoma mucin) (Testis mucin) (Ascites sialoglycoprotein) (ASGP) (Tracheobronchial mucin) [Contains: Mucin-4 alpha chain (Ascites sialoglycoprotein 1) (ASGP-1); Mucin-4 beta chain (Ascites sialoglycoprotein 2) (ASGP-2) [Source:Uniprot/SWISSPROT;Acc:Q99102]	20	-1.934	-2.016	0.804
MUC6	Mucin-6 precursor (Gastric mucin-6). [Source:Uniprot/SWISSPROT;Acc:Q6W4X9]	20	-1.954	-2.062	0.828
MYBPC1	Myosin-binding protein C, slow-type (Slow MyBP-C) (C-protein, skeletal muscle slow isoform). [Source:Uniprot/SWISSPROT;Acc:Q00872]	6	-2.855	-2.989	0.73
MYBPC1	Myosin-binding protein C, slow-type (Slow MyBP-C) (C-protein, skeletal muscle slow isoform). [Source:Uniprot/SWISSPROT;Acc:Q00872]	20	-1.936	-2.069	0.73

Gene_Symbol	Gene_Description	AS_Exon	Splicing_index	Log_2_ratio	Correlation_Coefficent
MYBPC3	Myosin-binding protein C, cardiac-type (Cardiac MyBP-C) (C-protein, cardiac muscle isoform). [Source:Uniprot/SWISSPROT;Acc:Q14896]	20	-1.882	-2.034	0.718
MYBPC3	Myosin-binding protein C, cardiac-type (Cardiac MyBP-C) (C-protein, cardiac muscle isoform). [Source:Uniprot/SWISSPROT;Acc:Q14896]	6	-2.895	-3.047	0.718
MYH1	Myosin-1 (Myosin heavy chain 1) (Myosin heavy chain 2x) (MyHC-2x) (Myosin heavy chain, skeletal muscle, adult 1) (Myosin heavy chain IIx/d) (MyHC-IIx/d). [Source:Uniprot/SWISSPROT;Acc:P12882]	20	-1.914	-2.055	0.739
MYH1	Myosin-1 (Myosin heavy chain 1) (Myosin heavy chain 2x) (MyHC-2x) (Myosin heavy chain, skeletal muscle, adult 1) (Myosin heavy chain IIx/d) (MyHC-IIx/d). [Source:Uniprot/SWISSPROT;Acc:P12882]	6	-2.918	-3.06	0.739
MYH10	Myosin-10 (Myosin heavy chain 10) (Myosin heavy chain, nonmuscle IIb) (Nonmuscle myosin heavy chain IIb) (NMMHC II-b) (NMMHC-IIB) (Cellular myosin heavy chain, type B) (Nonmuscle myosin heavy chain-B) (NMMHC- B). [Source:Uniprot/SWISSPROT;Acc:P35580]	6	-2.652	-2.922	0.747
MYH10	Myosin-10 (Myosin heavy chain 10) (Myosin heavy chain, nonmuscle IIb) (Nonmuscle myosin heavy chain IIb) (NMMHC II-b) (NMMHC-IIB) (Cellular myosin heavy chain, type B) (Nonmuscle myosin heavy chain-B) (NMMHC- B). [Source:Uniprot/SWISSPROT;Acc:P35580]	20	-1.711	-1.981	0.747
MYH11	Myosin-11 (Myosin heavy chain 11) (Myosin heavy chain, smooth muscle isoform) (SMMHC). [Source:Uniprot/SWISSPROT;Acc:P35749]	20	-1.916	-2.047	0.833
MYH2	Myosin-2 (Myosin heavy chain 2) (Myosin heavy chain 2a) (MyHC-2a) (Myosin heavy chain, skeletal muscle, adult 2) (Myosin heavy chain IIa) (MyHC-IIa). [Source:Uniprot/SWISSPROT;Acc:Q9UKX2]	20	-1.918	-2.092	0.728
MYH2	Myosin-2 (Myosin heavy chain 2) (Myosin heavy chain 2a) (MyHC-2a) (Myosin heavy chain, skeletal muscle, adult 2) (Myosin heavy chain IIa) (MyHC-IIa). [Source:Uniprot/SWISSPROT;Acc:Q9UKX2]	6	-2.933	-3.107	0.728

Gene_Symbol	Gene_Description	AS_Exon	Splicing_index	Log_2_ratio	Correlation_Coefficent
MYH3	Myosin-3 (Myosin heavy chain 3) (Myosin heavy chain, fast skeletal muscle, embryonic) (Muscle embryonic myosin heavy chain) (SMHCE). [Source:Uniprot/SWISSPROT;Acc:P11055]	6	-2.928	-3.067	0.734
MYH3	Myosin-3 (Myosin heavy chain 3) (Myosin heavy chain, fast skeletal muscle, embryonic) (Muscle embryonic myosin heavy chain) (SMHCE). [Source:Uniprot/SWISSPROT;Acc:P11055]	20	-1.959	-2.098	0.734
MYH4	Myosin-4 (Myosin heavy chain 4) (Myosin heavy chain 2b) (MyHC-2b) (Myosin heavy chain, skeletal muscle, fetal) (Myosin heavy chain IIb) (MyHC-IIb). [Source:Uniprot/SWISSPROT;Acc:Q9Y623]	20	-1.879	-2.018	0.737
MYH4	Myosin-4 (Myosin heavy chain 4) (Myosin heavy chain 2b) (MyHC-2b) (Myosin heavy chain, skeletal muscle, fetal) (Myosin heavy chain IIb) (MyHC-IIb). [Source:Uniprot/SWISSPROT;Acc:Q9Y623]	6	-2.929	-3.068	0.737
MYH6	Myosin-6 (Myosin heavy chain 6) (Myosin heavy chain, cardiac muscle alpha isoform) (MyHC-alpha). [Source:Uniprot/SWISSPROT;Acc:P13533]	6	-2.887	-3.036	0.733
MYH6	Myosin-6 (Myosin heavy chain 6) (Myosin heavy chain, cardiac muscle alpha isoform) (MyHC-alpha). [Source:Uniprot/SWISSPROT;Acc:P13533]	20	-1.904	-2.054	0.733
MYH7	Myosin-7 (Myosin heavy chain 7) (Myosin heavy chain, cardiac muscle beta isoform) (MyHC-beta) (Myosin heavy chain slow isoform) (MyHC- slow). [Source:Uniprot/SWISSPROT;Acc:P12883]	20	-1.922	-2.061	0.734
MYH7	Myosin-7 (Myosin heavy chain 7) (Myosin heavy chain, cardiac muscle beta isoform) (MyHC-beta) (Myosin heavy chain slow isoform) (MyHC- slow). [Source:Uniprot/SWISSPROT;Acc:P12883]	6	-2.86	-3	0.734
MYH7B	myosin, heavy polypeptide 7B, cardiac muscle, beta [Source:RefSeq_peptide;Acc:NP_065935]	20	-1.753	-2.083	0.927
MYH8	Myosin-8 (Myosin heavy chain 8) (Myosin heavy chain, skeletal muscle, perinatal) (MyHC-perinatal). [Source:Uniprot/SWISSPROT;Acc:P13535]	6	-2.966	-3.11	0.738

Gene_Symbol	Gene_Description	AS_Exon	Splicing_index	Log_2_ratio	Correlation_Coefficent
MYH8	Myosin-8 (Myosin heavy chain 8) (Myosin heavy chain, skeletal muscle, perinatal) (MyHC-perinatal). [Source:Uniprot/SWISSPROT;Acc:P13535]	20	-1.941	-2.085	0.738
MYH9	Myosin-9 (Myosin heavy chain 9) (Myosin heavy chain, nonmuscle IIa) (Nonmuscle myosin heavy chain IIa) (NMMHC II-a) (NMMHC-IIA) (Cellular myosin heavy chain, type A) (Nonmuscle myosin heavy chain-A) (NMMHC- A). [Source:Uniprot/SWISSPROT;Acc:P35579]	20	-1.622	-1.856	0.751
MYH9	Myosin-9 (Myosin heavy chain 9) (Myosin heavy chain, nonmuscle IIa) (Nonmuscle myosin heavy chain IIa) (NMMHC II-a) (NMMHC-IIA) (Cellular myosin heavy chain, type A) (Nonmuscle myosin heavy chain-A) (NMMHC- A). [Source:Uniprot/SWISSPROT;Acc:P35579]	6	-2.625	-2.859	0.751
MYLK	Myosin light chain kinase, smooth muscle (EC 2.7.11.18) (MLCK) (Telokin) (Kinase-related protein) (KRP). [Source:Uniprot/SWISSPROT;Acc:Q15746]	20	-1.964	-2.048	0.832
MYO10	Myosin-X (Unconventional myosin-10). [Source:Uniprot/SWISSPROT;Acc:Q9HD67]	20	-1.896	-2.058	0.843
MYO1A	Myosin-Ia (Brush border myosin I) (BBM-I) (BBMI) (Myosin I heavy chain) (MIHC). [Source:Uniprot/SWISSPROT;Acc:Q9UBC5]	20	-2.04	-2.022	0.798
MYO1C	Myosin-Ic (Myosin I beta) (MMI-beta) (MMIb). [Source:Uniprot/SWISSPROT;Acc:O00159]	20	-1.922	-1.938	0.826
MYO1D	Myosin-Id. [Source:Uniprot/SWISSPROT;Acc:O94832]	20	-1.888	-1.864	0.783
MYO1E	Myosin-Ie (Myosin-Ic). [Source:Uniprot/SWISSPROT;Acc:Q12965]	20	-1.817	-1.793	0.799
MYO1F	Myosin-If (Myosin-Ie). [Source:Uniprot/SWISSPROT;Acc:O00160]	20	-1.902	-1.942	0.812
MYO5A	Myosin-Va (Dilute myosin heavy chain, non-muscle) (Myosin-12) (Myosin heavy chain 12) (Myoxin). [Source:Uniprot/SWISSPROT;Acc:Q9Y4I1]	20	-1.945	-2.067	0.826
MYO5B	Myosin-Vb. [Source:Uniprot/SWISSPROT;Acc:Q9ULV0]	20	-1.893	-2.004	0.826

Gene_Symbol	Gene_Description	AS_Exon	Splicing_index	Log_2_ratio	Correlation_Coefficent
MYO6	Myosin-VI (Unconventional myosin VI). [Source:Uniprot/SWISSPROT;Acc:Q9UM54]	20	-1.921	-1.971	0.835
MYO7A	Myosin-VIIa. [Source:Uniprot/SWISSPROT;Acc:Q13402]	20	-1.89	-2.015	0.843
MYO7B	myosin VIIB [Source:RefSeq_peptide;Acc:NP_001073996]	20	-1.723	-1.86	0.842
MYO9A	myosin IXA [Source:RefSeq_peptide;Acc:NP_008832]	20	-1.904	-2.048	0.838
MYT1	Protein-L-isoaspartate O-methyltransferase domain-containing protein 2. [Source:Uniprot/SWISSPROT;Acc:Q9NV79]	20	-2.05	-2.07	0.805
NAALAD2	N-acetylated-alpha-linked acidic dipeptidase 2 (EC 3.4.17.21) (N- acetylated-alpha-linked acidic dipeptidase II) (NAALADase II). [Source:Uniprot/SWISSPROT;Acc:Q9Y3Q0]	15	-2.326	-1.646	0.88
NAALADL1	N-acetylated-alpha-linked acidic dipeptidase-like protein (EC 3.4.17.21) (NAALADase L) (Ileal dipeptidylpeptidase) (100 kDa ileum brush border membrane protein) (I100). [Source:Uniprot/SWISSPROT;Acc:Q9UQQ1]	15	-3.804	-3.616	0.878
NADSYN1	Glutamine-dependent NAD(+) synthetase (EC 6.3.5.1) (NAD(+) synthase [glutamine-hydrolyzing]) (NAD(+) synthetase 1). [Source:Uniprot/SWISSPROT;Acc:Q6IA69]	20	-1.932	-2.008	0.754
NARFL	nuclear prelamin A recognition factor-like [Source:RefSeq_peptide;Acc:NP_071938]	1	-1.586	-1.875	0.875
NBEAL1	Neurobeachin-like 1 (Amyotrophic lateral sclerosis 2 chromosomal region candidate gene 17 protein). [Source:Uniprot/SWISSPROT;Acc:Q6ZS30]	20	-1.829	-1.957	0.917
NCBP1	Nuclear cap-binding protein subunit 1 (80 kDa nuclear cap-binding protein) (NCBP 80 kDa subunit) (CBP80). [Source:Uniprot/SWISSPROT;Acc:Q09161]	20	-1.861	-1.814	0.793
NEB	Nebulin. [Source:Uniprot/SWISSPROT;Acc:P20929]	20	-1.903	-1.925	0.973
NEB	Nebulin. [Source:Uniprot/SWISSPROT;Acc:P20929]	136	-1.764	-1.786	0.973

Gene_Symbol	Gene_Description	AS_Exon	Splicing_index	Log_2_ratio	Correlation_Coefficent
NEBL	Nebulette (Actin-binding Z-disk protein). [Source:Uniprot/SWISSPROT;Acc:O76041]	28	-2.628	-2.505	0.839
NEDD4	E3 ubiquitin-protein ligase NEDD4 (EC 6.3.2.-). [Source:Uniprot/SWISSPROT;Acc:P46934]	20	-1.931	-1.946	0.849
NEK1	Serine/threonine-protein kinase Nek1 (EC 2.7.11.1) (NimA-related protein kinase 1) (Renal carcinoma antigen NY-REN-55). [Source:Uniprot/SWISSPROT;Acc:Q96PY6]	20	-2.175	-2.11	0.88
NELL1	Protein kinase C-binding protein NELL1 precursor (NEL-like protein 1) (Nel-related protein 1). [Source:Uniprot/SWISSPROT;Acc:Q92832]	20	-2.251	-2.123	0.902
NELL2	Protein kinase C-binding protein NELL2 precursor (NEL-like protein 2) (Nel-related protein 2). [Source:Uniprot/SWISSPROT;Acc:Q99435]	20	-2.202	-2.089	0.902
NEO1	Neogenin precursor. [Source:Uniprot/SWISSPROT;Acc:Q92859]	20	-1.897	-1.691	0.909
NF1	Neurofibromin (Neurofibromatosis-related protein NF-1) [Contains: Neurofibromin truncated]. [Source:Uniprot/SWISSPROT;Acc:P21359]	20	-2.025	-1.978	0.847
NFKB1	Nuclear factor NF-kappa-B p105 subunit (DNA-binding factor KBF1) (EBP- 1) [Contains: Nuclear factor NF-kappa-B p50 subunit]. [Source:Uniprot/SWISSPROT;Acc:P19838]	20	-1.786	-1.684	0.874
NFKB2	Nuclear factor NF-kappa-B p100 subunit (DNA-binding factor KBF2) (H2TF1) (Lymphocyte translocation chromosome 10) (Oncogene Lyt-10) (Lyt10) [Contains: Nuclear factor NF-kappa-B p52 subunit]. [Source:Uniprot/SWISSPROT;Acc:Q00653]	20	-1.745	-1.687	0.86
NFKBIL2	I-kappa-B-related protein [Source:RefSeq_peptide;Acc:NP_038460]	20	-1.837	-1.796	0.862
NFRKB	Nuclear factor related to kappa-B-binding protein (DNA-binding protein R kappa-B). [Source:Uniprot/SWISSPROT;Acc:Q6P4R8]	20	-1.785	-1.717	0.863

Gene_Symbol	Gene_Description	AS_Exon	Splicing_index	Log_2_ratio	Correlation_Coefficent
NFX1	Transcriptional repressor NF-X1 (EC 6.3.2.-) (Nuclear transcription factor, X box-binding, 1). [Source:Uniprot/SWISSPROT;Acc:Q12986]	20	-1.821	-1.763	0.861
NID1	Nidogen-1 precursor (Entactin). [Source:Uniprot/SWISSPROT;Acc:P14543]	20	-1.81	-1.708	0.875
NIN	Ninein (hNinein) (Glycogen synthase kinase 3 beta-interacting protein) (GSK3B-interacting protein). [Source:Uniprot/SWISSPROT;Acc:Q8N4C6]	20	-1.668	-1.726	0.786
NOL6	Nucleolar protein 6 (Nucleolar RNA-associated protein) (Nrap). [Source:Uniprot/SWISSPROT;Acc:Q9H6R4]	20	-1.621	-1.725	0.911
NOS1	Nitric-oxide synthase, brain (EC 1.14.13.39) (NOS type I) (Neuronal NOS) (N-NOS) (nNOS) (Constitutive NOS) (NC-NOS) (bNOS). [Source:Uniprot/SWISSPROT;Acc:P29475]	20	-1.947	-1.797	0.878
NOS2A	Nitric oxide synthase, inducible (EC 1.14.13.39) (NOS type II) (Inducible NO synthase) (Inducible NOS) (iNOS) (Hepatocyte NOS) (HEP- NOS). [Source:Uniprot/SWISSPROT;Acc:P35228]	20	-2.138	-1.989	0.887
NOS3	Nitric-oxide synthase, endothelial (EC 1.14.13.39) (EC-NOS) (NOS type III) (NOSIII) (Endothelial NOS) (eNOS) (Constitutive NOS) (cNOS). [Source:Uniprot/SWISSPROT;Acc:P29474]	20	-2.087	-1.96	0.894
NOTCH1	Neurogenic locus notch homolog protein 1 precursor (Notch 1) (hN1) (Translocation-associated notch protein TAN-1) [Contains: Notch 1 extracellular truncation; Notch 1 intracellular domain]. [Source:Uniprot/SWISSPROT;Acc:P46531]	20	-1.967	-1.926	0.867
NOTCH2	Neurogenic locus notch homolog protein 2 precursor (Notch 2) (hN2) [Contains: Notch 2 extracellular truncation; Notch 2 intracellular domain]. [Source:Uniprot/SWISSPROT;Acc:Q04721]	20	-1.849	-1.807	0.909
NOTCH3	Neurogenic locus notch homolog protein 3 precursor (Notch 3) [Contains: Notch 3 extracellular truncation; Notch 3 intracellular domain]. [Source:Uniprot/SWISSPROT;Acc:Q9UM47]	20	-1.832	-1.837	0.856

Gene_Symbol	Gene_Description	AS_Exon	Splicing_index	Log_2_ratio	Correlation_Coefficent
NOTCH4	G-protein-signaling modulator 3 (Activator of G-protein signaling 4) (Protein G18) (G18.1b). [Source:Uniprot/SWISSPROT;Acc:Q9Y4H4]	20	-1.814	-1.843	0.863
NPHP1	Nephrocystin-1 (Juvenile nephronophthisis 1 protein). [Source:Uniprot/SWISSPROT;Acc:O15259]	20	-1.93	-1.882	0.842
NPHS1	Nephrin precursor (Renal glomerulus-specific cell adhesion receptor). [Source:Uniprot/SWISSPROT;Acc:O60500]	20	-1.947	-1.893	0.866
NUP133	Nuclear pore complex protein Nup133 (Nucleoporin Nup133) (133 kDa nucleoporin). [Source:Uniprot/SWISSPROT;Acc:Q8WUM0]	20	-1.69	-1.68	0.81
OBSCN	Obscurin (Obscurin-myosin light chain kinase) (Obscurin-MLCK) (Obscurin-RhoGEF). [Source:Uniprot/SWISSPROT;Acc:Q5VST9]	20	-1.745	-1.915	0.936
ODF2	outer dense fiber of sperm tails 2 isoform 1 [Source:RefSeq_peptide;Acc:NP_002531]	20	-1.701	-1.62	0.837
ODZ1	Teneurin-1 (Ten-1) (Tenascin-M1) (Ten-m1) (Protein Odd Oz/ten-m homolog 1). [Source:Uniprot/SWISSPROT;Acc:Q9UKZ4]	28	-1.705	-1.769	0.712
ODZ2	Teneurin-2 (Ten-2) (Tenascin-M2) (Ten-m2) (Protein Odd Oz/ten-m homolog 2). [Source:Uniprot/SWISSPROT;Acc:Q9NT68]	20	-2.017	-2.115	0.884
ODZ3	Teneurin-3 (Ten-3) (Tenascin-M3) (Ten-m3) (Protein Odd Oz/ten-m homolog 3). [Source:Uniprot/SWISSPROT;Acc:Q9P273]	20	-1.629	-1.673	0.756
OGDH	2-oxoglutarate dehydrogenase E1 component, mitochondrial precursor (EC 1.2.4.2) (Alpha-ketoglutarate dehydrogenase). [Source:Uniprot/SWISSPROT;Acc:Q02218]	20	-1.685	-1.68	0.843
OGDHL	oxoglutarate dehydrogenase-like [Source:RefSeq_peptide;Acc:NP_060715]	20	-1.906	-1.954	0.737
OPA1	Dynamin-like 120 kDa protein, mitochondrial precursor (Optic atrophy protein 1) [Contains: Dynamin-like 120 kDa protein, form S1]. [Source:Uniprot/SWISSPROT;Acc:O60313]	20	-1.597	-1.616	0.867

Gene_Symbol	Gene_Description	AS_Exon	Splicing_index	Log_2_ratio	Correlation_Coefficent
OPHN1	Oligophrenin 1. [Source:Uniprot/SWISSPROT;Acc:O60890]	20	-1.75	-1.734	0.87
OSBPL1A	Oxysterol-binding protein-related protein 1 (OSBP-related protein 1) (ORP-1). [Source:Uniprot/SWISSPROT;Acc:Q9BXW6]	28	-2.174	-2.437	0.781
OTUD4	OTU domain-containing protein 4 (HIV-1-induced protein HIN-1). [Source:Uniprot/SWISSPROT;Acc:Q01804]	20	-1.651	-1.659	0.796
PACS1	Phosphofurin acidic cluster sorting protein 1 (PACS-1). [Source:Uniprot/SWISSPROT;Acc:Q6VY07]	20	-1.743	-1.787	0.741
PAM	Peptidyl-glycine alpha-amidating monooxygenase precursor (PAM) [Includes: Peptidylglycine alpha-hydroxylating monooxygenase (EC 1.14.17.3) (PHM); Peptidyl-alpha-hydroxyglycine alpha-amidating lyase (EC 4.3.2.5) (Peptidylamidoglycolate lyase) (PAL)]. [Source:Uniprot/SWISSPROT;Acc:P19021]	20	-1.678	-1.715	0.844
PAPPA	Pappalysin-1 precursor (EC 3.4.24.79) (Pregnancy-associated plasma protein-A) (PAPP-A) (Insulin-like growth factor-dependent IGF-binding protein 4 protease) (IGF-dependent IGFBP-4 protease) (IGFBP-4ase). [Source:Uniprot/SWISSPROT;Acc:Q13219]	20	-1.676	-1.721	0.835
PAPPA2	Pappalysin-2 precursor (EC 3.4.24.-) (Pregnancy-associated plasma protein-A2) (PAPP-A2) (Pregnancy-associated plasma protein-E1) (PAPP- E). [Source:Uniprot/SWISSPROT;Acc:Q9BXP8]	20	-1.73	-1.781	0.892
PARD3B	Partitioning-defective 3 homolog B (PAR3-beta) (Partitioning-defective 3-like protein) (PAR3-L protein) (Amyotrophic lateral sclerosis 2 chromosome region candidate gene 19 protein). [Source:Uniprot/SWISSPROT;Acc:Q8TEW8]	10	-1.654	-1.632	0.794
PARN	Poly(A)-specific ribonuclease PARN (EC 3.1.13.4) (Polyadenylate- specific ribonuclease) (Deadenylating nuclease) (Deadenylation nuclease). [Source:Uniprot/SWISSPROT;Acc:O95453]	20	-1.663	-1.617	0.839

Gene_Symbol	Gene_Description	AS_Exon	Splicing_index	Log_2_ratio	Correlation_Coefficent
PASD1	PAS domain containing 1 [Source:RefSeq_peptide;Acc:NP_775764]	10	-1.979	-2.13	0.964
PASK	PAS domain-containing serine/threonine-protein kinase (EC 2.7.11.1) (PAS-kinase) (PASKIN) (hPASK). [Source:Uniprot/SWISSPROT;Acc:Q96RG2]	10	-1.636	-1.665	0.899
PB1	Protein polybromo-1	20	-1.912	-1.936	0.77
PCCA	Propionyl-CoA carboxylase alpha chain, mitochondrial precursor (EC 6.4.1.3) (PCCase subunit alpha) (Propanoyl-CoA:carbon dioxide ligase subunit alpha). [Source:Uniprot/SWISSPROT;Acc:P05165]	20	-1.632	-1.622	0.839
PCLKC	Protocadherin LKC precursor	20	-1.852	-1.861	0.797
PCNT	Pericentrin (Pericentrin B) (Kendrin). [Source:Uniprot/SWISSPROT;Acc:O95613]	20	-1.625	-1.706	0.823
PDE11A	Dual 3',5'-cyclic-AMP and -GMP phosphodiesterase 11A (EC 3.1.4.17) (EC 3.1.4.35) (cAMP and cGMP phosphodiesterase 11A). [Source:Uniprot/SWISSPROT;Acc:Q9HCR9]	20	-1.751	-1.731	0.846
PDE2A	cGMP-dependent 3',5'-cyclic phosphodiesterase (EC 3.1.4.17) (Cyclic GMP-stimulated phosphodiesterase) (CGS-PDE) (cGSPDE). [Source:Uniprot/SWISSPROT;Acc:O00408]	20	-1.6	-1.653	0.795
PHIP	Bromodomain and WD repeat domain-containing protein 2 (WD repeat protein 11). [Source:Uniprot/SWISSPROT;Acc:Q9BZH6]	20	-1.769	-1.71	0.753
PITPNM2	Membrane-associated phosphatidylinositol transfer protein 2 (Phosphatidylinositol transfer protein, membrane-associated 2) (Pyk2 N-terminal domain-interacting receptor 3) (NIR-3). [Source:Uniprot/SWISSPROT;Acc:Q9BZ72]	20	-1.825	-2.031	0.915
PIWIL2	Piwi-like protein 2. [Source:Uniprot/SWISSPROT;Acc:Q8TC59]	20	-1.973	-1.99	0.714
PKD1L2	polycystin 1-like 2 isoform a [Source:RefSeq_peptide;Acc:NP_443124]	28	-2.182	-2.416	0.773

Gene_Symbol	Gene_Description	AS_Exon	Splicing_index	Log_2_ratio	Correlation_Coefficent
PLCE1	1-phosphatidylinositol-4,5-bisphosphate phosphodiesterase epsilon 1 (EC 3.1.4.11) (Phospholipase C-epsilon-1) (PLC-epsilon-1) (Phosphoinositide-specific phospholipase C epsilon-1) (Pancreas- enriched phospholipase C). [Source:Uniprot/SWISSPROT;Acc:Q9P212]	20	-1.664	-1.724	0.798
PLD1	Phospholipase D1 (EC 3.1.4.4) (PLD 1) (Choline phosphatase 1) (Phosphatidylcholine-hydrolyzing phospholipase D1) (hPLD1). [Source:Uniprot/SWISSPROT;Acc:Q13393]	21	-2.292	-2.462	0.929
PLEKHA4	Pleckstrin homology domain-containing family A member 4 (Phosphoinositol 3-phosphate-binding protein 1) (PEPP-1). [Source:Uniprot/SWISSPROT;Acc:Q9H4M7]	20	-1.822	-2.025	0.881
PLEKHG5	Tumor necrosis factor receptor superfamily member 25 precursor (WSL-1 protein) (Apoptosis-mediating receptor DR3) (Apoptosis-mediating receptor TRAMP) (Death domain receptor 3) (WSL protein) (Apoptosis-inducing receptor AIR) (Apo-3) (Lymphocyte-associate [Source:Uniprot/SWISSPROT;Acc:Q93038]	20	-1.734	-1.856	0.887
PLEKHH1	pleckstrin homology domain containing, family H (with MyTH4 domain) member 1 [Source:RefSeq_peptide;Acc:NP_065766]	20	-1.921	-2.055	0.924
PLXNA3	Plexin-A3 precursor (Plexin-4) (Semaphorin receptor SEX). [Source:Uniprot/SWISSPROT;Acc:P51805]	20	-1.728	-1.786	0.785
POLQ	DNA polymerase theta (EC 2.7.7.7) (DNA polymerase eta). [Source:Uniprot/SWISSPROT;Acc:O75417]	28	-2.144	-2.199	0.772
PPARGC1B	Peroxisome proliferator-activated receptor gamma coactivator 1-beta (PPAR gamma coactivator-1beta) (PGC-1-related estrogen receptor alpha coactivator) (PPARGC-1-beta) (PGC-1-beta). [Source:Uniprot/SWISSPROT;Acc:Q86YN6]	10	-1.649	-1.864	0.95

Gene_Symbol	Gene_Description	AS_Exon	Splicing_index	Log_2_ratio	Correlation_Coefficent
PPL	Periplakin (195 kDa cornified envelope precursor protein) (190 kDa paraneoplastic pemphigus antigen). [Source:Uniprot/SWISSPROT;Acc:O60437]	20	-1.884	-2.012	0.756
PPP1R12A	Protein phosphatase 1 regulatory subunit 12A (Myosin phosphatase- targeting subunit 1) (Myosin phosphatase target subunit 1) (Protein phosphatase myosin-binding subunit). [Source:Uniprot/SWISSPROT;Acc:O14974]	20	-1.91	-1.813	0.794
PPP1R12C	protein phosphatase 1, regulatory subunit 12C [Source:RefSeq_peptide;Acc:NP_060077]	20	-1.762	-1.872	0.754
PRKDC	DNA-dependent protein kinase catalytic subunit (EC 2.7.11.1) (DNA-PK catalytic subunit) (DNA-PKcs) (DNPK1) (p460). [Source:Uniprot/SWISSPROT;Acc:P78527]	61	-1.645	-2.213	0.845
PRPF8	Pre-mRNA-processing-splicing factor 8 (Splicing factor Prp8) (PRP8 homolog) (220 kDa U5 snRNP-specific protein) (p220). [Source:Uniprot/SWISSPROT;Acc:Q6P2Q9]	28	-1.739	-1.72	0.776
PRSS7	Enteropeptidase precursor (EC 3.4.21.9) (Enterokinase) (Serine protease 7) [Contains: Enteropeptidase non-catalytic heavy chain; Enteropeptidase catalytic light chain]. [Source:Uniprot/SWISSPROT;Acc:P98073]	20	-1.693	-1.852	0.887
PTCH1	Protein patched homolog 1 (PTC1) (PTC). [Source:Uniprot/SWISSPROT;Acc:Q13635]	20	-1.811	-1.855	0.894
PTCHD2	Patched domain-containing protein 2 (Fragment). [Source:Uniprot/SPTREMBL;Acc:Q5VTU9]	20	-1.745	-2.012	0.92
PTK2	Focal adhesion kinase 1 (EC 2.7.10.2) (FADK 1) (pp125FAK) (Protein- tyrosine kinase 2). [Source:Uniprot/SWISSPROT;Acc:Q05397]	20	-1.946	-2.041	0.922
PTK7	Tyrosine-protein kinase-like 7 precursor (Colon carcinoma kinase 4) (CCK-4). [Source:Uniprot/SWISSPROT;Acc:Q13308]	20	-1.91	-2.197	0.915

Gene_Symbol	Gene_Description	AS_Exon	Splicing_index	Log_2_ratio	Correlation_Coefficent
PTPN13	Tyrosine-protein phosphatase non-receptor type 13 (EC 3.1.3.48) (Protein-tyrosine phosphatase 1E) (PTP-E1) (hPTPE1) (PTP-BAS) (Protein-tyrosine phosphatase PTPL1) (Fas-associated protein-tyrosine phosphatase 1) (FAP-1). [Source:Uniprot/SWISSPROT;Acc:Q12923]	20	-1.912	-2.138	0.919
PTPN20A	protein tyrosine phosphatase, non-receptor type 20A isoform 2 [Source:RefSeq_peptide;Acc:NP_001035846]	20	-1.881	-2.037	0.927
PTPN4	Tyrosine-protein phosphatase non-receptor type 4 (EC 3.1.3.48) (Protein-tyrosine phosphatase MEG1) (PTPase-MEG1) (MEG). [Source:Uniprot/SWISSPROT;Acc:P29074]	20	-1.837	-2.088	0.924
PTPRA	Vacuolar protein sorting-associated protein 16 homolog (hVPS16). [Source:Uniprot/SWISSPROT;Acc:Q9H269]	20	-1.796	-2.023	0.929
PTPRB	Receptor-type tyrosine-protein phosphatase beta precursor (EC 3.1.3.48) (Protein-tyrosine phosphatase beta) (R-PTP-beta). [Source:Uniprot/SWISSPROT;Acc:P23467]	20	-1.943	-2.124	0.786
PTPRC	Leukocyte common antigen precursor (EC 3.1.3.48) (L-CA) (T200) (CD45 antigen). [Source:Uniprot/SWISSPROT;Acc:P08575]	20	-1.941	-2.122	0.903
PTPRD	Receptor-type tyrosine-protein phosphatase delta precursor (EC 3.1.3.48) (Protein-tyrosine phosphatase delta) (R-PTP-delta). [Source:Uniprot/SWISSPROT;Acc:P23468]	20	-1.92	-2.188	0.899
PTPRE	Receptor-type tyrosine-protein phosphatase epsilon precursor (EC 3.1.3.48) (Protein-tyrosine phosphatase epsilon) (R-PTP-epsilon). [Source:Uniprot/SWISSPROT;Acc:P23469]	20	-1.943	-2.146	0.885
PTPRG	Receptor-type tyrosine-protein phosphatase gamma precursor (EC 3.1.3.48) (Protein-tyrosine phosphatase gamma) (R-PTP-gamma). [Source:Uniprot/SWISSPROT;Acc:P23470]	20	-1.885	-2.071	0.888

Gene_Symbol	Gene_Description	AS_Exon	Splicing_index	Log_2_ratio	Correlation_Coefficent
PTPRJ	Receptor-type tyrosine-protein phosphatase eta precursor (EC 3.1.3.48) (Protein-tyrosine phosphatase eta) (R-PTP-eta) (HPTP eta) (Protein- tyrosine phosphatase receptor type J) (Density-enhanced phosphatase 1) (DEP-1) (CD148 antigen). [Source:Uniprot/SWISSPROT;Acc:Q12913]	20	-1.905	-2.152	0.879
PTPRM	Receptor-type tyrosine-protein phosphatase mu precursor (EC 3.1.3.48) (Protein-tyrosine phosphatase mu) (R-PTP-mu). [Source:Uniprot/SWISSPROT;Acc:P28827]	20	-1.99	-2.129	0.883
PTPRN	Receptor-type tyrosine-protein phosphatase-like N precursor (R-PTP-N) (PTP IA-2) (Islet cell antigen 512) (ICA 512) (Islet cell autoantigen 3). [Source:Uniprot/SWISSPROT;Acc:Q16849]	20	-1.893	-2.183	0.868
PTPRN2	Receptor-type tyrosine-protein phosphatase N2 precursor (EC 3.1.3.48) (R-PTP-N2) (Islet cell autoantigen-related protein) (ICAAR) (IAR) (Phogrin). [Source:Uniprot/SWISSPROT;Acc:Q92932]	20	-1.862	-2.17	0.882
PTPRO	Receptor-type tyrosine-protein phosphatase O precursor (EC 3.1.3.48) (Glomerular epithelial protein 1) (Protein tyrosine phosphatase U2) (PTPase U2) (PTP-U2). [Source:Uniprot/SWISSPROT;Acc:Q16827]	20	-1.936	-2.189	0.88
PTPRS	Receptor-type tyrosine-protein phosphatase S precursor (EC 3.1.3.48) (R-PTP-S) (Protein-tyrosine phosphatase sigma) (R-PTP-sigma). [Source:Uniprot/SWISSPROT;Acc:Q13332]	20	-1.865	-2.138	0.871
PTPRZ1	Receptor-type tyrosine-protein phosphatase zeta precursor (EC 3.1.3.48) (R-PTP-zeta). [Source:Uniprot/SWISSPROT;Acc:P23471]	20	-1.934	-2.151	0.894
PXDN	peroxidasin homolog [Source:RefSeq_peptide;Acc:NP_036425]	20	-2.338	-2.208	0.904
PYGB	Glycogen phosphorylase, brain form (EC 2.4.1.1). [Source:Uniprot/SWISSPROT;Acc:P11216]	20	-1.614	-1.785	0.894
PYGL	Glycogen phosphorylase, liver form (EC 2.4.1.1). [Source:Uniprot/SWISSPROT;Acc:P06737]	20	-1.619	-1.695	0.891

Gene_Symbol	Gene_Description	AS_Exon	Splicing_index	Log_2_ratio	Correlation_Coefficent
PZP	Pregnancy zone protein precursor. [Source:Uniprot/SWISSPROT;Acc:P20742]	20	-1.861	-1.92	0.869
RALGPS2	Ral GEF with PH domain and SH3 binding motif 2 isoform 2 [Source:RefSeq_peptide;Acc:NP_689876]	20	-1.718	-1.815	0.704
RANBP17	Ran-binding protein 17. [Source:Uniprot/SWISSPROT;Acc:Q9H2T7]	20	-1.933	-2.087	0.945
RET	Proto-oncogene tyrosine-protein kinase receptor ret precursor (EC 2.7.10.1) (C-ret). [Source:Uniprot/SWISSPROT;Acc:P07949]	20	-1.602	-1.732	0.876
RFX1	MHC class II regulatory factor RFX1 (RFX) (Enhancer factor C) (EF-C). [Source:Uniprot/SWISSPROT;Acc:P22670]	20	-1.586	-1.709	0.887
RIF1	Telomere-associated protein RIF1 (Rap1-interacting factor 1 homolog). [Source:Uniprot/SWISSPROT;Acc:Q5UIP0]	20	-1.84	-1.899	0.764
RNF17	RING finger protein 17. [Source:Uniprot/SWISSPROT;Acc:Q9BXT8]	20	-1.7	-1.789	0.841
RNF20	E3 ubiquitin-protein ligase BRE1A (EC 6.3.2.-) (BRE1-A) (hBRE1) (RING finger protein 20). [Source:Uniprot/SWISSPROT;Acc:Q5VTR2]	20	-1.62	-1.791	0.819
RNF31	Transcriptional regulator ISGF3 subunit gamma (Interferon regulatory factor 9) (IRF-9) (IFN-alpha-responsive transcription factor subunit) (Interferon-stimulated gene factor 3 gamma) (ISGF3 p48 subunit) (ISGF- 3 gamma). [Source:Uniprot/SWISSPROT;Acc:Q00978]	20	-1.852	-1.95	0.743
ROBO2	Roundabout homolog 2 precursor. [Source:Uniprot/SWISSPROT;Acc:Q9HCK4]	20	-1.606	-1.683	0.888
ROCK1	Rho-associated protein kinase 1 (EC 2.7.11.1) (Rho-associated, coiled- coil-containing protein kinase 1) (p160 ROCK-1) (p160ROCK) (Renal carcinoma antigen NY-REN-35). [Source:Uniprot/SWISSPROT;Acc:Q13464]	20	-1.635	-1.597	0.889
ROS1	Proto-oncogene tyrosine-protein kinase ROS precursor (EC 2.7.10.1) (c- ros-1). [Source:Uniprot/SWISSPROT;Acc:P08922]	20	-1.615	-1.783	0.894

Gene_Symbol	Gene_Description	AS_Exon	Splicing_index	Log_2_ratio	Correlation_Coefficent
RPS6KA1	Ribosomal protein S6 kinase alpha-1 (EC 2.7.11.1) (S6K-alpha 1) (90 kDa ribosomal protein S6 kinase 1) (p90-RSK 1) (Ribosomal S6 kinase 1) (RSK-1) (pp90RSK1) (p90S6K) (MAP kinase-activated protein kinase 1a) (MAPKAPK1A). [Source:Uniprot/SWISSPROT;Acc:Q15418]	20	-1.745	-1.727	0.902
RPS6KA2	Ribosomal protein S6 kinase alpha-2 (EC 2.7.11.1) (S6K-alpha 2) (90 kDa ribosomal protein S6 kinase 2) (p90-RSK 2) (Ribosomal S6 kinase 3) (RSK-3) (pp90RSK3) (MAP kinase-activated protein kinase 1c) (MAPKAPK1C). [Source:Uniprot/SWISSPROT;Acc:Q15349]	20	-1.766	-1.764	0.901
RSN	Reed-Steinberg cell-expressed intermediate filament-associated protein	20	-1.716	-1.723	0.905
RYR1	Ryanodine receptor 1 (Skeletal muscle-type ryanodine receptor) (RyR1) (RYR-1) (Skeletal muscle calcium release channel). [Source:Uniprot/SWISSPROT;Acc:P21817]	105	-1.752	-1.885	0.963
RYR1	Ryanodine receptor 1 (Skeletal muscle-type ryanodine receptor) (RyR1) (RYR-1) (Skeletal muscle calcium release channel). [Source:Uniprot/SWISSPROT;Acc:P21817]	61	-2.149	-2.282	0.963
RYR2	Ryanodine receptor 2 (Cardiac muscle-type ryanodine receptor) (RyR2) (RYR-2) (Cardiac muscle ryanodine receptor-calcium release channel) (hRYR-2). [Source:Uniprot/SWISSPROT;Acc:Q92736]	61	-3.584	-3.732	0.968
RYR2	Ryanodine receptor 2 (Cardiac muscle-type ryanodine receptor) (RyR2) (RYR-2) (Cardiac muscle ryanodine receptor-calcium release channel) (hRYR-2). [Source:Uniprot/SWISSPROT;Acc:Q92736]	20	-1.604	-1.752	0.968
RYR3	Ryanodine receptor 3 (Brain-type ryanodine receptor) (RyR3) (RYR-3) (Brain ryanodine receptor-calcium release channel). [Source:Uniprot/SWISSPROT;Acc:Q15413]	61	-3.138	-3.282	0.957

Gene_Symbol	Gene_Description	AS_Exon	Splicing_index	Log_2_ratio	Correlation_Coefficent
RYR3	Ryanodine receptor 3 (Brain-type ryanodine receptor) (RyR3) (RYR-3) (Brain ryanodine receptor-calcium release channel). [Source:Uniprot/SWISSPROT;Acc:Q15413]	20	-1.621	-1.766	0.957
RYR3	Ryanodine receptor 3 (Brain-type ryanodine receptor) (RyR3) (RYR-3) (Brain ryanodine receptor-calcium release channel). [Source:Uniprot/SWISSPROT;Acc:Q15413]	30	-3.196	-3.341	0.957
SBNO1	sno, strawberry notch homolog 1 [Source:RefSeq_peptide;Acc:NP_060653]	20	-1.9	-1.788	0.751
SCUBE2	Signal peptide, CUB and EGF-like domain-containing protein 2 precursor (Protein CEGP1). [Source:Uniprot/SWISSPROT;Acc:Q9NQ36]	20	-1.785	-2.119	0.935
SDK2	Protein sidekick-2 precursor. [Source:Uniprot/SWISSPROT;Acc:Q58EX2]	20	-1.713	-1.837	0.839
SENP7	Sentrin-specific protease 7 (EC 3.4.22.-) (Sentrin/SUMO-specific protease SENP7) (SUMO-1-specific protease 2). [Source:Uniprot/SWISSPROT;Acc:Q9BQF6]	20	-1.826	-1.883	0.898
SFMBT2	Scm-like with four MBT domains protein 2. [Source:Uniprot/SWISSPROT;Acc:Q5VUG0]	20	-1.926	-2.127	0.903
SFRS15	Splicing factor, arginine/serine-rich 15 (CTD-binding SR-like protein RA4). [Source:Uniprot/SWISSPROT;Acc:O95104]	20	-1.696	-1.79	0.913
SFTPB	Pulmonary surfactant-associated protein B precursor (SP-B) (6 kDa protein) (Pulmonary surfactant-associated proteolipid SPL(Phe)) (18 kDa pulmonary-surfactant protein). [Source:Uniprot/SWISSPROT;Acc:P07988]	1	-1.703	-1.919	0.886
SGCA	Alpha-sarcoglycan precursor (Alpha-SG) (Adhalin) (50 kDa dystrophin- associated glycoprotein) (50DAG) (Dystroglycan 2). [Source:Uniprot/SWISSPROT;Acc:Q16586]	1	-1.623	-1.876	0.882
SHANK1	Synaptotagmin-3 (Synaptotagmin III) (SytIII). [Source:Uniprot/SWISSPROT;Acc:Q9BQG1]	20	-1.616	-1.619	0.842

Gene_Symbol	Gene_Description	AS_Exon	Splicing_index	Log_2_ratio	Correlation_Coefficent
SIDT1	SID1 transmembrane family member 1 precursor. [Source:Uniprot/SWISSPROT;Acc:Q9NXL6]	20	-2.033	-2.036	0.772
SIGLEC1	Sialoadhesin precursor (Sialic acid-binding Ig-like lectin 1) (Siglec- 1) (CD169 antigen). [Source:Uniprot/SWISSPROT;Acc:Q9BZZ2]	20	-1.773	-1.896	0.923
SKIV2L	superkiller viralicidic activity 2-like homolog [Source:RefSeq_peptide;Acc:NP_008860]	20	-1.775	-1.875	0.92
SLC12A1	Solute carrier family 12 member 1 (Bumetanide-sensitive sodium- (potassium)-chloride cotransporter 2) (Kidney-specific Na-K-Cl symporter). [Source:Uniprot/SWISSPROT;Acc:Q13621]	20	-1.857	-2.026	0.931
SLC12A2	Solute carrier family 12 member 2 (Bumetanide-sensitive sodium- (potassium)-chloride cotransporter 1) (Basolateral Na-K-Cl symporter). [Source:Uniprot/SWISSPROT;Acc:P55011]	20	-1.724	-1.773	0.906
SLC12A3	Solute carrier family 12 member 3 (Thiazide-sensitive sodium-chloride cotransporter) (Na-Cl symporter). [Source:Uniprot/SWISSPROT;Acc:P55017]	20	-1.596	-1.78	0.932
SLC12A4	Solute carrier family 12 member 4 (Electroneutral potassium-chloride cotransporter 1) (Erythroid K-Cl cotransporter 1) (hKCC1). [Source:Uniprot/SWISSPROT;Acc:Q9UP95]	20	-1.668	-1.861	0.955
SLC12A5	Solute carrier family 12 member 5 (Electroneutral potassium-chloride cotransporter 2) (Erythroid K-Cl cotransporter 2) (Neuronal K-Cl cotransporter) (hKCC2). [Source:Uniprot/SWISSPROT;Acc:Q9H2X9]	20	-1.987	-2.184	0.918
SLC15A1	Oligopeptide transporter, small intestine isoform (Peptide transporter 1) (Intestinal H(+)/peptide cotransporter) (Solute carrier family 15 member 1). [Source:Uniprot/SWISSPROT;Acc:P46059]	20	-1.886	-2.1	0.932
SLC15A2	Oligopeptide transporter, kidney isoform (Peptide transporter 2) (Kidney H(+)/peptide cotransporter) (Solute carrier family 15 member 2). [Source:Uniprot/SWISSPROT;Acc:Q16348]	20	-1.872	-2.098	0.936

Gene_Symbol	Gene_Description	AS_Exon	Splicing_index	Log_2_ratio	Correlation_Coefficent
SLC4A10	Sodium-driven chloride bicarbonate exchanger (Solute carrier family 4 member 10). [Source:Uniprot/SWISSPROT;Acc:Q6U841]	20	-2.008	-2.071	0.898
SLC4A3	Anion exchange protein 3 (Neuronal band 3-like protein) (Solute carrier family 4 member 3) (Cardiac/brain band 3-like protein) (CAE3/BAE3). [Source:Uniprot/SWISSPROT;Acc:P48751]	20	-1.622	-1.802	0.91
SLC4A5	sodium bicarbonate transporter 4 isoform c [Source:RefSeq_peptide;Acc:NP_597812]	20	-1.885	-2.094	0.934
SLCO6A1	solute carrier organic anion transporter family, member 6A1 [Source:RefSeq_peptide;Acc:NP_775759]	10	-1.805	-1.971	0.955
SLIT1	Slit homolog 1 protein precursor (Slit-1) (Multiple epidermal growth factor-like domains 4). [Source:Uniprot/SWISSPROT;Acc:O75093]	20	-1.882	-2.03	0.949
SLIT3	Slit homolog 3 protein precursor (Slit-3) (Multiple epidermal growth factor-like domains 5). [Source:Uniprot/SWISSPROT;Acc:O75094]	20	-1.881	-2.018	0.944
SLMAP	Sarcolemmal membrane-associated protein (Sarcolemmal-associated protein). [Source:Uniprot/SWISSPROT;Acc:Q14BN4]	20	-1.973	-1.804	0.901
SMARCAD1	SWI/SNF-related matrix-associated actin-dependent regulator of chromatin subfamily A containing DEAD/H box 1 (EC 3.6.1.-) (ATP- dependent helicase 1) (hHEL1). [Source:Uniprot/SWISSPROT;Acc:Q9H4L7]	20	-1.679	-1.762	0.839
SMARCC2	SWI/SNF-related matrix-associated actin-dependent regulator of chromatin subfamily C member 2 (SWI/SNF complex 170 kDa subunit) (BRG1-associated factor 170). [Source:Uniprot/SWISSPROT;Acc:Q8TAQ2]	20	-1.78	-1.889	0.921

Gene_Symbol	Gene_Description	AS_Exon	Splicing_index	Log_2_ratio	Correlation_Coefficent
SNAPC4	snRNA-activating protein complex subunit 4 (SNAPc subunit 4) (snRNA- activating protein complex 190 kDa subunit) (SNAPc 190 kDa subunit) (Proximal sequence element-binding transcription factor subunit alpha) (PSE-binding factor subunit alpha) (PTF subunit [Source:Uniprot/SWISSPROT;Acc:Q5SXM2]	20	-2.028	-2.141	0.936
SNX14	Sorting nexin-14. [Source:Uniprot/SWISSPROT;Acc:Q9Y5W7]	20	-1.984	-2.076	0.93
SORCS2	VPS10 domain-containing receptor SorCS2 precursor. [Source:Uniprot/SWISSPROT;Acc:Q96PQ0]	20	-1.852	-2.093	0.93
SORL1	Sortilin-related receptor precursor (Sorting protein-related receptor containing LDLR class A repeats) (SorLA) (SorLA-1) (Low-density lipoprotein receptor relative with 11 ligand-binding repeats) (LDLR relative with 11 ligand-binding repeats) (LR11). [Source:Uniprot/SWISSPROT;Acc:Q92673]	20	-1.868	-1.92	0.92
SOS2	Son of sevenless homolog 2 (SOS-2). [Source:Uniprot/SWISSPROT;Acc:Q07890]	20	-1.975	-1.934	0.929
SOX5	Transcription factor SOX-5. [Source:Uniprot/SWISSPROT;Acc:P35711]	20	-2.047	-2.075	0.928
SP100	Nuclear autoantigen Sp-100 (Speckled 100 kDa) (Nuclear dot-associated Sp100 protein) (Lysp100b). [Source:Uniprot/SWISSPROT;Acc:P23497]	20	-2.382	-2.391	0.916
SPAG1	Sperm-associated antigen 1 (Infertility-related sperm protein Spag-1) (HSD-3.8). [Source:Uniprot/SWISSPROT;Acc:Q07617]	20	-1.75	-1.732	0.915
SPAG4L	Sperm-associated antigen 4-like protein (Testis and spermatogenesis- related gene 4 protein). [Source:Uniprot/SWISSPROT;Acc:Q8TC36]	10	-1.763	-2.111	0.91
SPTA1	Spectrin alpha chain, erythrocyte (Erythroid alpha-spectrin). [Source:Uniprot/SWISSPROT;Acc:P02549]	20	-1.753	-1.862	0.936
SPTB	Spectrin beta chain, erythrocyte (Beta-I spectrin). [Source:Uniprot/SWISSPROT;Acc:P11277]	20	-1.714	-1.826	0.95

Gene_Symbol	Gene_Description	AS_Exon	Splicing_index	Log_2_ratio	Correlation_Coefficent
SRGAP1	SLIT-ROBO Rho GTPase-activating protein 1 (srGAP1) (Rho GTPase- activating protein 13). [Source:Uniprot/SWISSPROT;Acc:Q7Z6B7]	20	-1.866	-2.118	0.936
STAB2	Stabilin-2 precursor (Fasciclin, EGF-like, laminin-type EGF-like and link domain-containing scavenger receptor 2) (FEEL-2) (FAS1 EGF-like and X-link domain-containing adhesion molecule 2) (Hyaluronan receptor for endocytosis) [Contains: 190 kDa form stabi [Source:Uniprot/SWISSPROT;Acc:Q8WWQ8]	20	-1.589	-1.756	0.779
STAB2	Stabilin-2 precursor (Fasciclin, EGF-like, laminin-type EGF-like and link domain-containing scavenger receptor 2) (FEEL-2) (FAS1 EGF-like and X-link domain-containing adhesion molecule 2) (Hyaluronan receptor for endocytosis) [Contains: 190 kDa form stabi [Source:Uniprot/SWISSPROT;Acc:Q8WWQ8]	61	-3.265	-3.432	0.779
STAG1	Cohesin subunit SA-1 (Stromal antigen 1) (SCC3 homolog 1). [Source:Uniprot/SWISSPROT;Acc:Q8WVM7]	28	-1.771	-1.786	0.796
STAG2	Cohesin subunit SA-2 (Stromal antigen 2) (SCC3 homolog 2). [Source:Uniprot/SWISSPROT;Acc:Q8N3U4]	28	-2.103	-1.995	0.757
STAG3	Cohesin subunit SA-3 (Stromal antigen 3) (Stromalin 3) (SCC3 homolog 3). [Source:Uniprot/SWISSPROT;Acc:Q9UJ98]	28	-2.149	-2.246	0.752
STAT5A	Signal transducer and activator of transcription 5A. [Source:Uniprot/SWISSPROT;Acc:P42229]	20	-2.134	-2.209	0.915
STAT6	Signal transducer and activator of transcription 6 (IL-4 Stat). [Source:Uniprot/SWISSPROT;Acc:P42226]	20	-1.852	-1.953	0.918
STK31	Serine/threonine-protein kinase 31 (EC 2.7.11.1) (Serine/threonine- protein kinase NYD-SPK) (Sugen kinase 396). [Source:Uniprot/SWISSPROT;Acc:Q9BXU1]	20	-1.63	-1.786	0.858
STK32A	Serine/threonine-protein kinase 32A (EC 2.7.11.1) (YANK1). [Source:Uniprot/SWISSPROT;Acc:Q8WU08]	5	-1.642	-1.9	0.971

Gene_Symbol	Gene_Description	AS_Exon	Splicing_index	Log_2_ratio	Correlation_Coefficent
SUSD1	Sushi domain-containing protein 1 precursor. [Source:Uniprot/SWISSPROT;Acc:Q6UWL2]	1	-1.601	-1.835	0.888
SYCP1	Synaptonemal complex protein 1 (SCP-1). [Source:Uniprot/SWISSPROT;Acc:Q15431]	20	-1.648	-1.728	0.935
SYCP2	Synaptonemal complex protein 2 (SCP-2) (Synaptonemal complex lateral element protein) (hsSCP2). [Source:Uniprot/SWISSPROT;Acc:Q9BX26]	28	-2.259	-2.485	0.848
SYNE1	Nesprin-1 (Nuclear envelope spectrin repeat protein 1) (Synaptic nuclear envelope protein 1) (Syne-1) (Myocyte nuclear envelope protein 1) (Myne-1) (Enaptin). [Source:Uniprot/SWISSPROT;Acc:Q8NF91]	142	-2.607	-2.657	0.955
SYNE2	Nesprin-2 (Nuclear envelope spectrin repeat protein 2) (Syne-2) (Synaptic nuclear envelope protein 2) (Nucleus and actin connecting element protein) (Protein NUANCE). [Source:Uniprot/SWISSPROT;Acc:Q8WXH0]	107	-2.82	-2.868	0.95
SYTL2	Synaptotagmin-like protein 2 (Exophilin-4). [Source:Uniprot/SWISSPROT;Acc:Q9HCH5]	20	-1.953	-1.956	0.758
TAGAP	T-cell activation Rho GTPase-activating protein (T-cell activation GTPase-activating protein). [Source:Uniprot/SWISSPROT;Acc:Q8N103]	10	-1.741	-1.863	0.903
TARBP1	Probable methyltransferase TARBP1 (EC 2.1.1.-) (TAR RNA-binding protein 1) (TAR RNA-binding protein of 185 kDa) (TRP-185). [Source:Uniprot/SWISSPROT;Acc:Q13395]	20	-1.596	-1.647	0.917
TCF4	Transcription factor 4 (Immunoglobulin transcription factor 2) (ITF-2) (SL3-3 enhancer factor 2) (SEF-2). [Source:Uniprot/SWISSPROT;Acc:P15884]	20	-1.826	-1.87	0.91
TCOF1	Treacher Collins-Franceschetti syndrome 1 isoform b [Source:RefSeq_peptide;Acc:NP_000347]	20	-1.832	-1.859	0.901
TDRD1	Tudor domain-containing protein 1. [Source:Uniprot/SWISSPROT;Acc:Q9BXT4]	20	-1.671	-1.79	0.854
TECTA	Alpha-tectorin precursor. [Source:Uniprot/SWISSPROT;Acc:O75443]	20	-1.957	-2.035	0.882

Gene_Symbol	Gene_Description	AS_Exon	Splicing_index	Log_2_ratio	Correlation_Coefficent
TEK	Angiopoietin-1 receptor precursor (EC 2.7.10.1) (Tyrosine-protein kinase receptor TIE-2) (hTIE2) (Tyrosine-protein kinase receptor TEK) (p140 TEK) (Tunica interna endothelial cell kinase) (CD202b antigen). [Source:Uniprot/SWISSPROT;Acc:Q02763]	20	-2.123	-2.051	0.912
TEP1	Telomerase protein component 1 (Telomerase-associated protein 1) (Telomerase protein 1) (p240) (p80 telomerase homolog). [Source:Uniprot/SWISSPROT;Acc:Q99973]	20	-1.911	-1.952	0.903
TEX11	testis expressed sequence 11 isoform 1 [Source:RefSeq_peptide;Acc:NP_001003811]	20	-1.719	-1.781	0.846
TEX14	Testis-expressed protein 14 (Testis-expressed sequence 14) (Protein kinase-like protein SgK307) (Sugen kinase 307). [Source:Uniprot/SWISSPROT;Acc:Q8IWB6]	20	-1.66	-1.718	0.849
TG	Thyroglobulin precursor. [Source:Uniprot/SWISSPROT;Acc:P01266]	20	-1.868	-1.869	0.905
THBS1	Thrombospondin-1 precursor. [Source:Uniprot/SWISSPROT;Acc:P07996]	20	-1.862	-1.839	0.915
THBS2	Thrombospondin-2 precursor. [Source:Uniprot/SWISSPROT;Acc:P35442]	20	-1.954	-1.878	0.919
THBS3	Thrombospondin-3 precursor. [Source:Uniprot/SWISSPROT;Acc:P49746]	20	-1.868	-1.788	0.92
TIAM1	T-lymphoma invasion and metastasis-inducing protein 1 (TIAM-1 protein). [Source:Uniprot/SWISSPROT;Acc:Q13009]	20	-1.944	-1.866	0.918
TIE1	Tyrosine-protein kinase receptor Tie-1 precursor (EC 2.7.10.1). [Source:Uniprot/SWISSPROT;Acc:P35590]	20	-1.967	-1.894	0.921
TLL1	Tolloid-like protein 1 precursor (EC 3.4.24.-). [Source:Uniprot/SWISSPROT;Acc:O43897]	9	-1.633	-1.71	0.917
TMC1	Transmembrane channel-like protein 1 (Transmembrane cochlear-expressed protein 1). [Source:Uniprot/SWISSPROT;Acc:Q8TDI8]	10	-1.771	-1.733	0.845

Gene_Symbol	Gene_Description	AS_Exon	Splicing_index	Log_2_ratio	Correlation_Coefficent
TMC2	Transmembrane channel-like protein 2 (Transmembrane cochlear-expressed protein 2). [Source:Uniprot/SWISSPROT;Acc:Q8TDI7]	10	-1.841	-1.782	0.874
TMEM16G	transmembrane protein 16G isoform NGEP long [Source:RefSeq_peptide;Acc:NP_001001891]	20	-1.667	-1.703	0.841
TNR	Tenascin-R precursor (TN-R) (Restrictin) (Janusin). [Source:Uniprot/SWISSPROT;Acc:Q92752]	20	-1.8	-1.738	0.89
TNS1	Tensin-1. [Source:Uniprot/SWISSPROT;Acc:Q9HBL0]	20	-1.595	-1.588	0.896
TNXB	Cyclic AMP-dependent transcription factor ATF-6 beta (Activating transcription factor 6 beta) (ATF6-beta) (cAMP-responsive element- binding protein-like 1) (cAMP response element-binding protein-related protein) (Creb-rp) (Protein G13). [Source:Uniprot/SWISSPROT;Acc:Q99941]	20	-1.627	-1.702	0.89
TRIM37	Tripartite motif-containing protein 37 (Mulibrey nanism protein). [Source:Uniprot/SWISSPROT;Acc:O94972]	20	-1.898	-1.914	0.808
TRPM1	transient receptor potential cation channel, subfamily M, member 1 [Source:RefSeq_peptide;Acc:NP_002411]	20	-2.079	-1.988	0.792
TRPM4	Transient receptor potential cation channel subfamily M member 4 (Long transient receptor potential channel 4) (hTRPM4) (Melastatin-4) (Calcium-activated non-selective cation channel 1). [Source:Uniprot/SWISSPROT;Acc:Q8TD43]	20	-1.761	-1.836	0.756
TRPM7	Transient receptor potential cation channel subfamily M member 7 (EC 2.7.11.1) (Long transient receptor potential channel 7) (LTrpC7) (Channel-kinase 1). [Source:Uniprot/SWISSPROT;Acc:Q96QT4]	20	-1.841	-1.82	0.8
TRPM8	Transient receptor potential cation channel subfamily M member 8 (Transient receptor potential-p8) (Trp-p8) (Long transient receptor potential channel 6) (LTrpC6). [Source:Uniprot/SWISSPROT;Acc:Q7Z2W7]	20	-2.042	-2.183	0.899

Gene_Symbol	Gene_Description	AS_Exon	Splicing_index	Log_2_ratio	Correlation_Coefficent
TSNARE1	t-SNARE domain-containing protein 1. [Source:Uniprot/SWISSPROT;Acc:Q96NA8]	5	-1.86	-1.868	0.788
TTC12	Tetratricopeptide repeat protein 12 (TPR repeat protein 12). [Source:Uniprot/SWISSPROT;Acc:Q9H892]	20	-1.756	-1.842	0.721
TTC13	Tetratricopeptide repeat protein 13 (TPR repeat protein 13). [Source:Uniprot/SWISSPROT;Acc:Q8NBP0]	20	-1.771	-1.853	0.866
TTC18	tetratricopeptide repeat domain 18 [Source:RefSeq_peptide;Acc:NP_660153]	28	-2.267	-2.522	0.949
TTC7A	Tetratricopeptide repeat protein 7A (TPR repeat protein 7A). [Source:Uniprot/SWISSPROT;Acc:Q9ULT0]	20	-1.886	-2.062	0.924
TTN	Titin (EC 2.7.11.1) (Connectin) (Rhabdomyosarcoma antigen MU-RMS- 40.14). [Source:Uniprot/SWISSPROT;Acc:Q8WZ42]	61	-3.331	-3.511	0.966
TTN	Titin (EC 2.7.11.1) (Connectin) (Rhabdomyosarcoma antigen MU-RMS- 40.14). [Source:Uniprot/SWISSPROT;Acc:Q8WZ42]	146	-1.994	-2.174	0.966
TTN	Titin (EC 2.7.11.1) (Connectin) (Rhabdomyosarcoma antigen MU-RMS- 40.14). [Source:Uniprot/SWISSPROT;Acc:Q8WZ42]	105	-2.342	-2.522	0.966
TTN	Titin (EC 2.7.11.1) (Connectin) (Rhabdomyosarcoma antigen MU-RMS- 40.14). [Source:Uniprot/SWISSPROT;Acc:Q8WZ42]	162	-2.405	-2.585	0.966
UACA	Uveal autoantigen with coiled-coil domains and ankyrin repeats. [Source:Uniprot/SWISSPROT;Acc:Q9BZF9]	20	-1.672	-1.814	0.721
UBTF	Nucleolar transcription factor 1 (Upstream-binding factor 1) (UBF-1) (Autoantigen NOR-90). [Source:Uniprot/SWISSPROT;Acc:P17480]	20	-1.698	-1.596	0.928
UGCGL1	UDP-glucose:glycoprotein glucosyltransferase 1 precursor (EC 2.4.1.-) (UDP-glucose ceramide glucosyltransferase-like 1) (UDP-- Glc:glycoprotein glucosyltransferase) (HUGT1). [Source:Uniprot/SWISSPROT;Acc:Q9NYU2]	20	-1.621	-1.792	0.874

Gene_Symbol	Gene_Description	AS_Exon	Splicing_index	Log_2_ratio	Correlation_Coefficent
UGCGL2	UDP-glucose:glycoprotein glucosyltransferase 2 precursor (EC 2.4.1.-) (UDP-glucose ceramide glucosyltransferase-like 1) (UDP-- Glc:glycoprotein glucosyltransferase 2) (HUGT2). [Source:Uniprot/SWISSPROT;Acc:Q9NYU1]	20	-1.872	-1.937	0.775
ULK1	Serine/threonine-protein kinase ULK1 (EC 2.7.11.1) (Unc-51-like kinase 1). [Source:Uniprot/SWISSPROT;Acc:O75385]	20	-1.658	-1.735	0.847
ULK4	Serine/threonine-protein kinase ULK4 (EC 2.7.11.1) (Unc-51-like kinase 4). [Source:Uniprot/SWISSPROT;Acc:Q96C45]	20	-1.785	-1.843	0.775
UNC13B	Unc-13 homolog B (Munc13-2) (munc13). [Source:Uniprot/SWISSPROT;Acc:O14795]	28	-2.599	-2.398	0.849
UNC13D	Unc-13 homolog D (Munc13-4). [Source:Uniprot/SWISSPROT;Acc:Q70J99]	5	-1.898	-1.743	0.951
UROC1	Probable urocanate hydratase (EC 4.2.1.49) (Urocanase) (Imidazolonepropionate hydrolase). [Source:Uniprot/SWISSPROT;Acc:Q96N76]	10	-2.01	-2.151	0.948
USH2A	Usherin precursor (Usher syndrome type-2A protein) (Usher syndrome type IIa protein). [Source:Uniprot/SWISSPROT;Acc:O75445]	20	-1.85	-1.881	0.918
USP28	Ubiquitin carboxyl-terminal hydrolase 28 (EC 3.1.2.15) (Ubiquitin thioesterase 28) (Ubiquitin-specific-processing protease 28) (Deubiquitinating enzyme 28). [Source:Uniprot/SWISSPROT;Acc:Q96RU2]	20	-1.833	-2.052	0.912
USP37	Ubiquitin carboxyl-terminal hydrolase 37 (EC 3.1.2.15) (Ubiquitin thioesterase 37) (Ubiquitin-specific-processing protease 37) (Deubiquitinating enzyme 37). [Source:Uniprot/SWISSPROT;Acc:Q86T82]	20	-1.731	-1.928	0.928
USP47	Ubiquitin carboxyl-terminal hydrolase 47 (EC 3.1.2.15) (Ubiquitin thioesterase 47) (Ubiquitin-specific-processing protease 47) (Deubiquitinating enzyme 47). [Source:Uniprot/SWISSPROT;Acc:Q96K76]	20	-1.653	-1.607	0.79

Gene_Symbol	Gene_Description	AS_Exon	Splicing_index	Log_2_ratio	Correlation_Coefficent
UST	Uronyl 2-sulfotransferase (EC 2.8.2.-). [Source:Uniprot/SWISSPROT;Acc:Q9Y2C2]	8	-1.59	-1.811	0.918
UTRN	Utrophin (Dystrophin-related protein 1) (DRP1) (DRP). [Source:Uniprot/SWISSPROT;Acc:P46939]	20	-1.728	-1.697	0.912
UTX	Ubiquitously transcribed X chromosome tetratricopeptide repeat protein (Ubiquitously transcribed TPR protein on the X chromosome). [Source:Uniprot/SWISSPROT;Acc:O15550]	20	-1.982	-1.809	0.932
UTY	Ubiquitously transcribed Y chromosome tetratricopeptide repeat protein (Ubiquitously transcribed TPR protein on the Y chromosome). [Source:Uniprot/SWISSPROT;Acc:O14607]	20	-2.001	-1.876	0.932
VAV1	Proto-oncogene vav. [Source:Uniprot/SWISSPROT;Acc:P15498]	20	-1.963	-1.847	0.931
VAV2	Protein vav-2. [Source:Uniprot/SWISSPROT;Acc:P52735]	20	-1.76	-1.588	0.927
VPS13A	Vacuolar protein sorting-associated protein 13A (Chorein) (Chorea- acanthocytosis protein). [Source:Uniprot/SWISSPROT;Acc:Q96RL7]	60	-2.005	-1.939	0.934
VPS13B	Vacuolar protein sorting-associated protein 13B (Cohen syndrome protein 1). [Source:Uniprot/SWISSPROT;Acc:Q7Z7G8]	18	-1.876	-1.751	0.748
VPS13C	Vacuolar protein sorting-associated protein 13C. [Source:Uniprot/SWISSPROT;Acc:Q709C8]	20	-1.715	-1.821	0.883
VPS13D	Vacuolar protein sorting-associated protein 13D. [Source:Uniprot/SWISSPROT;Acc:Q5THJ4]	20	-2.02	-2.064	0.804
VWF	von Willebrand factor precursor (vWF) [Contains: von Willebrand antigen 2 (von Willebrand antigen II)]. [Source:Uniprot/SWISSPROT;Acc:P04275]	20	-1.876	-1.873	0.938
WDR35	WD repeat protein 35. [Source:Uniprot/SWISSPROT;Acc:Q9P2L0]	20	-1.849	-2.089	0.934
WDR60	WD repeat protein 60. [Source:Uniprot/SWISSPROT;Acc:Q8WVS4]	20	-1.711	-1.838	0.856
WDR75	WD repeat protein 75. [Source:Uniprot/SWISSPROT;Acc:Q8IWA0]	20	-1.741	-1.745	0.79

Gene_Symbol	Gene_Description	AS_Exon	Splicing_index	Log_2_ratio	Correlation_Coefficent
WHSC1L1	Histone-lysine N-methyltransferase NSD3 (EC 2.1.1.43) (Nuclear SET domain-containing protein 3) (WHSC1-like protein 1) (Wolf-Hirschhorn syndrome candidate 1-like protein 1) (Whistle) (WHSC1-like 1 isoform 9 with methyltransferase activity to lysine). [Source:Uniprot/SWISSPROT;Acc:Q9BZ95]	20	-1.806	-1.804	0.762
WNK2	Serine/threonine-protein kinase WNK2 (EC 2.7.11.1) (Protein kinase with no lysine 2) (Protein kinase, lysine-deficient 2). [Source:Uniprot/SWISSPROT;Acc:Q9Y3S1]	20	-1.915	-2.021	0.905
WNK3	Serine/threonine-protein kinase WNK3 (EC 2.7.11.1) (Protein kinase with no lysine 3) (Protein kinase, lysine-deficient 3). [Source:Uniprot/SWISSPROT;Acc:Q9BYP7]	20	-2.124	-2.227	0.918
WRN	Werner syndrome ATP-dependent helicase (EC 3.6.1.-). [Source:Uniprot/SWISSPROT;Acc:Q14191]	20	-1.758	-1.69	0.916
XDH	Xanthine dehydrogenase/oxidase [Includes: Xanthine dehydrogenase (EC 1.17.1.4) (XD); Xanthine oxidase (EC 1.17.3.2) (XO) (Xanthine oxidoreductase)]. [Source:Uniprot/SWISSPROT;Acc:P47989]	20	-1.802	-1.79	0.912
XPNPEP1	Xaa-Pro aminopeptidase 1 (EC 3.4.11.9) (X-Pro aminopeptidase 1) (X- prolyl aminopeptidase 1, soluble) (Cytosolic aminopeptidase P) (Soluble aminopeptidase P) (sAmp) (Aminoacylproline aminopeptidase). [Source:Uniprot/SWISSPROT;Acc:Q9NQW7]	20	-1.888	-1.827	0.91
XPNPEP2	Xaa-Pro aminopeptidase 2 precursor (EC 3.4.11.9) (X-Pro aminopeptidase 2) (Membrane-bound aminopeptidase P) (Membrane-bound APP) (Membrane- bound AmP) (mAmP) (Aminoacylproline aminopeptidase). [Source:Uniprot/SWISSPROT;Acc:O43895]	20	-1.854	-1.792	0.917
XPO1	Exportin-1 (Exp1) (Chromosome region maintenance 1 protein homolog). [Source:Uniprot/SWISSPROT;Acc:O14980]	20	-1.925	-1.665	0.913
YEATS2	YEATS domain-containing protein 2. [Source:Uniprot/SWISSPROT;Acc:Q9ULM3]	20	-1.758	-1.793	0.769

Gene_Symbol	Gene_Description	AS_Exon	Splicing_index	Log_2_ratio	Correlation_Coefficent
YLPM1	YLP motif-containing protein 1 (Nuclear protein ZAP3) (ZAP113). [Source:Uniprot/SWISSPROT;Acc:P49750]	20	-1.585	-1.738	0.833
ZAN	Zonadhesin precursor. [Source:Uniprot/SWISSPROT;Acc:Q9Y493]	20	-1.674	-1.75	0.89
ZFP106	Zinc finger protein 106 homolog (Zfp-106) (Zinc finger protein 474). [Source:Uniprot/SWISSPROT;Acc:Q9H2Y7]	1	-1.633	-1.816	0.904
ZNF185	Zinc finger protein 185 (LIM domain protein ZNF185) (P1-A). [Source:Uniprot/SWISSPROT;Acc:O15231]	20	-1.952	-1.863	0.888
ZNF236	Zinc finger protein 236. [Source:Uniprot/SWISSPROT;Acc:Q9UL36]	20	-1.859	-1.812	0.898
ZNF291	Zinc finger protein 291. [Source:Uniprot/SWISSPROT;Acc:Q9BY12]	20	-1.674	-1.672	0.875
ZPLD1	zona pellucida-like domain containing 1 [Source:RefSeq_peptide;Acc:NP_778226]	10	-1.959	-2.234	0.98
ZRANB3	Zinc finger Ran-binding domain-containing protein 3 (EC 3.6.1.-). [Source:Uniprot/SWISSPROT;Acc:Q5FWF4]	20	-1.692	-1.786	0.826
