# Supplementary material for: Small Molecule Amiloride Modulates Oncogenic RNA Alternative Splicing to Devitalize Human Cancer Cells
Source: PLoS One. 2011 Jun 9;6(6):e18643. doi: 10.1371/journal.pone.0018643 (PMC3111415; doi:10.1371/journal.pone.0018643)
Supplement: Table S2 — The significant GO terms of genes with alternative RNA splicing altered in amiloride-treated Huh-7 cells. (PDF) [file pone.0018643.s006.pdf]

Supplemetary Table 2. Significant GO Terms in alternative splicing genes.

Moleuclar Function Category

Term	Count	%	P-Value	Genes
GO:0003774~motor activity	35	6.45%	1.11E-20	MYO7B, DNAH5, MYH7, DNAH8, MYH2, MYO1D, MYH6, MYO7A, DNAH3, MYH10, MYO5A, MYH1, CENPE, MYO1F, MYO5B, MYH4, MYH8, MYH7B, MYH9, DNAH7, MYO9A, MYH3, KIF1A, DNAH1, MYH11, MYO6, MYO1E, MYO1A, CGN, MYO1C, MYO10, KIF13B, C20orf23, DNHD2, DNAH10,
GO:0030554~adenyl nucleotide binding	111	20.44%	3.56E-19	ZRANB3, CAD, MYH7, NOS1, DHX35, MYO7A, ABCC2, MYH1, TEK, MARK2, MYH7B, TRPM7, MYO9A, ACACA, CDC42BPG, AKT3, PTK2, BMX, MYO10, TTN, CACNA1B, ABCC4, KIF13B, NIN, DNAH10, FLJ20035, MINK1, SMARCAD1, RET, STK32A, ULK1, DNAH3, C11orf11, MYH10, DDX54, CENPE, MYO5B, MYH9, PRKDC, XDH, MYH3, MYH11, MYO1E, OBSCN, NOS2A, WNK3, MTHFD1, RPS6KA1, CNGB1, ATP8A1, ACOT12, PTK7, ADCK5, PASK, POLQ, WRN, CAMK2G, BUB1, MYO7B, DHX37, RPS6KA2, INOC1, STK31, MYH6, TEX14, MAP4K1, CHD8, MYO5A, MYO1F, WNK2, DDX21, ROCK1, DNAH7, ATP1A4, ULK4, EP400, ROS1, BRIP1, SKIV2L, MST1R, NADSYN1, FANCM, ATP1A2, DNAH5, ATP2A1, PCCA, DNAH8, MYH2, MYO1D, ATP1A3, TEP1, DUOX2, MYH4, MYH8, NEK1, LRRK2, BUB1B, TIE1, KIF1A, MYO6, MYO1A, MYO1C, ATP12A, ACSS1, CDKL5, C20orf23, TRPM4, NOS3, ATRX, MYLK
GO:0032559~adenyl ribonucleotide binding	106	19.52%	2.25E-18	ZRANB3, CAD, MYH7, DHX35, MYO7A, ABCC2, MYH1, TEK, MARK2, MYH7B, TRPM7, MYO9A, ACACA, CDC42BPG, AKT3, PTK2, BMX, MYO10, TTN, CACNA1B, ABCC4, KIF13B, NIN, DNAH10, FLJ20035, MINK1, SMARCAD1, RET, STK32A, ULK1, DNAH3, C11orf11, MYH10, DDX54, CENPE, MYO5B, MYH9, PRKDC, MYH3, MYH11, MYO1E, OBSCN, WNK3, MTHFD1, RPS6KA1, CNGB1, ATP8A1, ACOT12, PTK7, ADCK5, PASK, POLQ, WRN, CAMK2G, BUB1, MYO7B, DHX37, RPS6KA2, INOC1, MYH6, TEX14, STK31, MAP4K1, CHD8, MYO5A, MYO1F, WNK2, DDX21, ROCK1, DNAH7, ATP1A4, ULK4, EP400, ROS1, BRIP1, SKIV2L, MST1R, NADSYN1, FANCM, ATP1A2, DNAH5, ATP2A1, PCCA, DNAH8, MYH2, MYO1D, ATP1A3, TEP1, MYH4, MYH8, NEK1, LRRK2, BUB1B, TIE1, KIF1A, MYO6, MYO1A, MYO1C, ATP12A, ACSS1

Term	Count	%	P-Value	Genes
GO:0005524~ATP binding	104	19.15%	8.39E-18	ZRANB3, CAD, MYH7, DHX35, MYO7A, ABCC2, MYH1, TEK, MARK2, MYH7B, TRPM7, MYO9A, ACACA, CDC42BPG, AKT3, PTK2, BMX, MYO10, TTN, CACNA1B, ABCC4, KIF13B, NIN, DNAH10, FLJ20035, MINK1, SMARCAD1, RET, STK32A, ULK1, DNAH3, C11orf11, MYH10, DDX54, CENPE, MYO5B, MYH9, PRKDC, MYH3, MYH11, MYO1E, OBSCN, WNK3, MTHFD1, RPS6KA1, ATP8A1, ACOT12, PTK7, ADCK5, PASK, POLQ, WRN, CAMK2G, BUB1, MYO7B, DHX37, RPS6KA2, INOC1, MYH6, TEX14, STK31, MAP4K1, CHD8, MYO5A, MYO1F, WNK2, DDX21, ROCK1, DNAH7, ATP1A4, ULK4, EP400, ROS1, BRIP1, SKIV2L, MST1R, NADSYN1, FANCM, ATP1A2, DNAH5, ATP2A1, PCCA, DNAH8, MYH2, MYO1D, ATP1A3, TEP1, MYH4, MYH8, NEK1, LRRK2, BUB1B, TIE1, KIF1A, MYO6, MYO1A, MYO1C, ATP12A, CDKL5, C20orf23, TRPM4
GO:0005516~calmodulin binding	29	5.34%	6.25E-17	PCNT, MYH7, MYH2, NOS1, MYO1D, MYH6, MYO7A, MYH10, MYO5A, MYH1, MYO1F, MYO5B, MYH4, MYH8, MYH9, MYH3, RYR2, MYH11, MYO6, MYO1A, MYO1E, NOS2A, MYO1C, TTN, CACNA1C, CAMK2G, TRPM4, NOS3, MYLK,
GO:0000146~microfilament motor activity	13	2.39%	9.11E-16	MYH3, MYO6, MYO1E, MYH7, MYH2, MYH6, MYO7A, MYH10, MYH1, MYO5A, MYO1F, MYH4, MYH9,
GO:0017076~purine nucleotide binding	115	21.18%	2.28E-14	ZRANB3, CAD, MYH7, NOS1, DHX35, MYO7A, ABCC2, MYH1, TEK, MARK2, MYH7B, TRPM7, MYO9A, ACACA, CDC42BPG, AKT3, PTK2, BMX, MYO10, TTN, CACNA1B, ABCC4, KIF13B, NIN, DNAH10, FLJ20035, MINK1, SMARCAD1, RET, STK32A, ULK1, DNAH3, C11orf11, MYH10, DDX54, CENPE, MYO5B, MYH9, XDH, PRKDC, MYH3, MYH11, MYO1E, OBSCN, NOS2A, WNK3, MTHFD1, RPS6KA1, CNGB1, ATP8A1, ACOT12, ADCK5, PTK7, RANBP17, PASK, POLQ, WRN, CAMK2G, BUB1, MYO7B, DHX37, RPS6KA2, INOC1, SPAG1, STK31, MYH6, TEX14, MAP4K1, CHD8, MYO5A, MYO1F, WNK2, DDX21, ROCK1, DNAH7, ATP1A4, ULK4, EP400, ROS1, BRIP1, SKIV2L, MST1R, NADSYN1, FANCM, ATP1A2, DNAH5, ATP2A1, PCCA, DNAH8, MYH2, MYO1D, ATP1A3, TEP1, DUOX2, OPA1, MYH4, MYH8, NEK1, LRRK2, BUB1B, TIE1, KIF1A, MYO6, MYO1A, MYO1C, ATP12A, ACSS1, CDKL5, CCDC40, C20orf23, TRPM4
GO:0032555~purine ribonucleotide binding	110	20.26%	1.25E-13	ZRANB3, CAD, MYH7, DHX35, MYO7A, ABCC2, MYH1, TEK, MARK2, MYH7B, TRPM7, MYO9A, ACACA, CDC42BPG, AKT3, PTK2, BMX, MYO10, TTN, CACNA1B, ABCC4, KIF13B, NIN, DNAH10, FLJ20035, MINK1, SMARCAD1, RET, STK32A, ULK1, DNAH3, C11orf11, MYH10, DDX54, CENPE, MYO5B, MYH9, PRKDC, MYH3, MYH11, MYO1E, OBSCN, WNK3, MTHFD1, RPS6KA1, CNGB1, ATP8A1, ACOT12, PTK7, ADCK5, RANBP17, PASK, POLQ, WRN, CAMK2G, BUB1, MYO7B, DHX37, RPS6KA2, INOC1, SPAG1, STK31, MYH6, TEX14, MAP4K1, CHD8, MYO5A, MYO1F, WNK2, DDX21, ROCK1, DNAH7, ATP1A4, ULK4, EP400, ROS1, BRIP1, SKIV2L, MST1R, NADSYN1, FANCM, ATP1A2, DNAH5, ATP2A1, PCCA, DNAH8, MYH2, MYO1D, ATP1A3, TEP1, OPA1, MYH4, MYH8, NEK1, LRRK2, BUB1B, TIE1, KIF1A, MYO6, MYO1A, MYO1C, ATP12A, ACSS1, CDKL5, CCDC40, C20orf23, TRPM4, MYLK, ATRX

Term	Count	%	P-Value	Genes
GO:0032553~ribonucleotide binding	110	20.26%	1.25E-13	ZRANB3, CAD, MYH7, DHX35, MYO7A, ABCC2, MYH1, TEK, MARK2, MYH7B, TRPM7, MYO9A, ACACA, CDC42BPG, AKT3, PTK2, BMX, MYO10, TTN, CACNA1B, ABCC4, KIF13B, NIN, DNAH10, FLJ20035, MINK1, SMARCAD1, RET, STK32A, ULK1, DNAH3, C11orf11, MYH10, DDX54, CENPE, MYO5B, MYH9, PRKDC, MYH3, MYH11, MYO1E, OBSCN, WNK3, MTHFD1, RPS6KA1, CNGB1, ATP8A1, ACOT12, PTK7, ADCK5, RANBP17, PASK, POLQ, WRN, CAMK2G, BUB1, MYO7B, DHX37, RPS6KA2, INOC1, SPAG1, STK31, MYH6, TEX14, MAP4K1, CHD8, MYO5A, MYO1F, WNK2, DDX21, ROCK1, DNAH7, ATP1A4, ULK4, EP400, ROS1, BRIP1, SKIV2L, MST1R, NADSYN1, FANCM, ATP1A2, DNAH5, ATP2A1, PCCA, DNAH8, MYH2, MYO1D, ATP1A3, TEP1, OPA1, MYH4, MYH8, NEK1, LRRK2, BUB1B, TIE1, KIF1A, MYO6, MYO1A, MYO1C, ATP12A, ACSS1, CDKL5, CCDC40, C20orf23, TRPM4, MYLK, ATRX,
GO:0019198~transmembrane receptor protein phosphatase activity	12	2.21%	1.67E-13	PTPRD, PTPRS, PTPRO, PTPRB, PTPRJ, PTPRM, PTPRA, PTPRE, PTPRC, PTPRG, PTPRZ1, PTPRN2, PTPRN,
GO:0008092~cytoskeletal protein binding	44	8.10%	3.25E-13	SPTA1, OPHN1, MYH7, NEB, MYH2, TNS1, MYH6, MYO1D, MYO7A, MYBPC3, MYH10, FMNL1, MYO5A, ARFGEF1, MYH1, MYO1F, MYO5B, BRCA1, MYH4, FRMD4A, MYH8, SPTB, MYH9, SYNE1, TRPM7, RSN, UTRN, MYH3, MYH11, MYO6, CDK5RAP2, MYO1E, MYO1A, PTPN4, NEBL, SYNE2, CGN, MYO1C, TTN, DST, MYO10, MYBPC1,
GO:0003779~actin binding	36	6.63%	6.76E-13	SPTA1, OPHN1, MYH7, NEB, MYH2, TNS1, MYH6, MYO1D, MYO7A, MYBPC3, MYH10, FMNL1, MYO5A, MYH1, MYO1F, MYO5B, MYH4, MYH8, SPTB, MYH9, SYNE1, TRPM7, UTRN, MYH3, MYH11, MYO6, MYO1E, MYO1A, NEBL, SYNE2, CGN, MYO1C, MYO10, DST, MYBPC1, DMD,
GO:0005201~extracellular matrix structural constituent	22	4.05%	1.27E-12	COL4A3, COL3A1, MUC4, MUC2, MUC6, COL4A2, COMP, TNXB, COL4A4, TNR, COL5A1, COL4A1, COL9A1, COL4A5, TECTA, COL1A2, COL5A2, COL2A1, NID1, COL12A1, COL1A1, COL11A2,
GO:0000166~nucleotide binding	121	22.28%	1.75E-12	ZRANB3, CAD, MYH7, NOS1, DHX35, MYO7A, ABCC2, MYH1, TEK, MARK2, MYH7B, TRPM7, MYO9A, ACACA, CDC42BPG, PYGL, AKT3, PTK2, TTN, PARN, BMX, MYO10, CACNA1B, ABCC4, KIF13B, NIN, DNAH10, FLJ20035, MINK1, SMARCAD1, RET, ULK1, STK32A, DNAH3, C11orf11, MYH10, DDX54, CENPE, MYO5B, MYH9, XDH, PRKDC, MYH3, MYH11, MYO1E, OBSCN, NOS2A, WNK3, MTHFD1, RPS6KA1, CNGB1, ATP8A1, ACOT12, ADCK5, PTK7, RANBP17, PASK, POLQ, WRN, CAMK2G, BUB1, SFRS15, PPARGC1B, MYO7B, DHX37, RPS6KA2, INOC1, SPAG1, STK31, MYH6, TEX14, MAP4K1, CHD8, MYO5A, MYO1F, WNK2, DDX21, ROCK1, DNAH7, ATP1A4, ULK4, EP400, ROS1, BRIP1, DRB1, SKIV2L, MST1R, NADSYN1, FANCM, ATP1A2, DNAH5, ATP2A1, PCCA, DNAH8, MYH2, MYO1D, ATP1A3, TEP1, DUOX2, OPA1, MYH4, MYH8, NEK1, LRRK2, BUB1B, TIE1, KIF1A, MYO6, MYO1A, MYO1C, ATP12A, ACSS1, CDKL5, CCDC40, C20orf23, TRPM4, C17orf27, NOS3, MYLK, ATRX, ADRBK2

Term	Count	%	P-Value	Genes
GO:0005515~protein binding	263	48.43%	1.90E-09	COL4A3, BAT5, XPO1, NEB, UGCGL2, BAI1, TNS1, ABCC2, MPP4, MYH1, COL1A2, FAM48A, MARK2, FRMD4A, SLIT3, DIP2B, TRPM7, IL31RA, PLCE1, EXOC2, NEBL, PARN, BMX, MYO10, CACNA1B, KIF13B, IPO11, RNF20, NIN, ARFGEF2, PZP, STAT6, SMARCAD1, TCOF1, VAV1, RET, USH2A, DIP13B, CARD14, CD2BP2, MYH10, FMNL1, ARHGAP17, CENPE, MYH9, SYNE1, RSN, PRKDC, CASKIN1, MYH3, MTR, MYH11, MYO1E, OBSCN, WNK3, PTPN4, NFKBIL2, CACNA1E, DST, PTPRZ1, CACNA1C, PTK7, WRN, RNF31, STAG1, NOTCH3, TCF4, SDK2, SPTA1, PPARGC1B, PXDN, PTPRE, PPL, TIAM1, INADL, CNTNAP5, CHD8, NPHS1, MYO1F, CLEC7A, THBS1, CLCA1, COL20A1, CDK5RAP2, COMP, ALS2, TNR, DSCAML1, KIAA1604, VWF, COG7, PCLKC, DMD, CRHR2, COL12A1, EP300, CLTC, FANCM, SYCP1, MLL3, CDON, PB1, ZAN, MYH2, ROBO2, ANKHD1, STAB2, NUP133, SIGLEC1, SGCA, TEX11, UBTF, SOS2, LRRK2, PTPRN, UTRN, MUC2, KIAA1324, TNXB, MYO1C, ENG, SHANK1, WHSC1L1, THBS2, PPP1R12A, PTPRB, C20orf23, TRPM4, ATM, NOS3, FTS, MYLK, PHIP, PCNT, DCBLD2, MYH7, PAM, RALGPS2, NOS1, MYO7A, SYTL2, UGCGL1, CDC42BPG, ACACA, STAG2
GO:0005262~calcium channel activity	16	2.95%	1.98E-09	RYR2, RYR3, CACNA1S, TRPM1, RYR1, CACNA1E, TRPM8, CACNA1B, CACNA2D1, CACNA1C, CACNB2, TRPM4, CACNA1A, TRPM7, CACNA1F, CACNA1D,
GO:0005509~calcium ion binding	61	11.23%	6.45E-09	SPTA1, EMR1, PKD1L2, ENPEP, NOTCH2, CACNA2D1, CACNB2, SLIT3, TRPM7, THBS1, DNAH7, PLCE1, NOTCH4, MCTP2, CACNA1S, COMP, TTN, PITPNM2, CACNA1B, PCLKC, LRP1, CDH23, DMD, NOTCH1, NELL2, NIN, CACHD1, THBS3, CACNA1F, SLIT1, STAT6, RYR3, ATP2A1, RET, ITSN2, C11orf11, DUOX2, SGCA, SCUBE2, CACNA1A, GNPTAB, UTRN, DCHS2, RYR2, NOS2A, RYR1, NELL1, TLL1, CACNA1E, DST, SUSD1, THBS2, STAT5A, CACNA1C, CDH5, NID1, TRPM4, NOS3,
GO:0005198~structural molecule activity	57	10.50%	7.95E-09	COL4A3, SPTA1, COL17A1, MUC4, MYH7, NEB, PPL, MYH6, COL9A1, TECTA, COL1A2, COL2A1, SPTB, SLIT3, THBS1, COL9A2, COL20A1, COL22A1, NEBL, COMP, SYNE2, TNR, TTN, COL4A1, COL5A1, MYBPC1, COL5A2, DMD, COL12A1, NELL2, CLTC, THBS3, COL11A2, MUC6, USH2A, COL7A1, MYBPC3, PTPN13, MYH8, SYNE1, UTRN, COL3A1, MYH11, MUC2, COL4A2, NPHP1, PTPN4, ODF2, TNXB, NELL1, COL4A4, DST, COL16A1, COL4A5, THBS2, NID1, COL1A1,

Term	Count	%	P-Value	Genes
GO:0005488~binding	404	74.40%	3.22E-07	BAT5, XPO1, NEB, SLC12A2, BAI1, TNS1, CACNA2D1, MYH1, FAM48A, MARK2, SLIT3, DIP2B, TRPM7, FAM65A, MYO9A, IL31RA, PLCE1, C20orf132, EXOC2, RIF1, XPNPEP1, NEBL, FLJ20433, OGDHL, IPO11, RNF20, CACNA1F, PZP, STAT6, SMARCAD1, VAV1, RET, CARD14, CD2BP2, FMNL1, ARHGAP17, ARMC4, SYNE1, RSN, PRKDC, MYH3, CASKIN1, MTR, MYO1E, WNK3, TLL1, NFKBIL2, KHDRBS2, PTPRZ1, ACOT12, CACNA1C, PTK7, POLQ, GON4L, RNF31, SDK2, KIAA1468, PTPRE, TEX14, INADL, SLC12A5, CHD8, THBS1, XPNPEP2, DNAH7, CLCA1, CDK5RAP2, KIAA1524, COMP, CASZ1, DSCAML1, DMD, COL12A1, MST1R, CRHR2, CLTC, EP300, PB1, ROBO2, ANKHD1, STAB2, TTC12, PLEKHA4, TEX11, SOS2, LRRK2, PTPRN, DCHS2, MUC2, KIAA1622, RYR1, TNXB, ATP12A, SLC12A3, SHANK1, PTPRB, ATM, MYLK, ZRANB3, MYH7, PAM, SLC12A1, RALGPS2, NAALAD2, MYO7A, TECTA, FLJ31438, DGKK, SYTL2, MCTP2, PYGL, STAG2, AKT3, ZNF236, TDRD1, COL5A1, MYBPC1, NOTCH1, FLJ36748, DNAH10, ENO2, CACHD1, THBS3, ADAMTS10, SLIT1, HSPG2, ZNF185, MINK1, NFKB1, C20orf12, NF1, C11orf11, SCUBE2, HECW2, CNTN2, SNX14, TTC13,
GO:0016887~ATPase activity	30	5.52%	4.73E-07	FLJ20035, DNAH5, MYH7, DHX37, ATP2A1, DNAH8, INOC1, MYH6, DHX35, ABCC2, DNAH3, ATP1A3, DDX54, MYH4, MYH9, DDX21, DNAH7, MYH3, ATP1A4, MYO1E, ATP12A, BRIP1, ATP8A1, ABCC4, SKIV2L, POLQ, WRN, DNAH10, FANCM, ATP1A2,
GO:0005085~guanyl-nucleotide exchange factor activity	19	3.50%	7.22E-07	VAV2, KIAA1244, PLEKHG5, OBSCN, TAGAP, FGD2, VAV1, RALGPS2, ANKRD27, TIAM1, ALS2, ITSN2, ARFGEF1, ARHGEF10L, SPTB, SOS2, ARFGEF2, FLJ10357, PLCE1,
GO:0004725~protein tyrosine phosphatase activity	15	2.76%	1.00E-06	PTPRD, PTPRS, PTPN4, PTPRE, PTPRA, PTPN20A, PTPRG, PTPRZ1, PTPRN2, PTPN13, PTPRO, PTPRB, PTPRM, PTPRJ, PTPRC, PTPRN,
GO:0005245~voltage-gated calcium channel activity	9	1.66%	1.08E-06	CACNA1B, CACNA2D1, CACNA1S, CACNA1C, CACNB2, CACNA1A, CACNA1E, CACNA1F, CACNA1D,
GO:0008307~structural constituent of	10	1.84%	1.37E-06	MYBPC3, MYH11, MYBPC1, MYH7, NEB, DMD, MYH8, NEBL, TTN, MYH6,
GO:0004672~protein kinase activity	41	7.55%	7.29E-06	MINK1, RPS6KA2, C9orf39, RET, STK31, TEX14, ULK1, STK32A, MAP4K1, TEK, WNK2, MARK2, NEK1, CTTNBP2, LRRK2, TRPM7, ROCK1, PRKDC, BUB1B, TIE1, ULK4, CDC42BPG, OBSCN, WNK3, ROS1, AKT3, PTK2, TTN, BMX, RPS6KA1, CDKL5, ADCK5, PTK7, PASK, MST1R, CAMK2G, ATM, NIN, BUB1, ADRBK2, MYLK,
GO:0004674~protein serine/threonine kinase activity	31	5.71%	7.83E-06	MINK1, RPS6KA2, RET, STK31, STK32A, ULK1, MAP4K1, MARK2, WNK2, NEK1, LRRK2, TRPM7, ROCK1, PRKDC, BUB1B, TIE1, ULK4, CDC42BPG, OBSCN, WNK3, AKT3, TTN, RPS6KA1, CDKL5, ADCK5, PASK, CAMK2G, ATM, BUB1, MYLK, ADRBK2,
GO:0030695~GTPase regulator activity	29	5.34%	8.06E-06	OPHN1, MINK1, ARHGAP27, VAV1, RALGPS2, TIAM1, ITSN2, SRGAP1, NF1, MAP4K1, ARFGEF1, ARHGAP17, CENTD3, ARHGEF10L, SPTB, SOS2, LRRK2, PLCE1, FLJ10357, VAV2, KIAA1244, PLEKHG5, CDC42BPG, OBSCN, TAGAP, FGD2, ANKRD27, ALS2,
GO:0005089~Rho guanyl-nucleotide exchange factor activity	12	2.21%	9.19E-06	VAV2, PLEKHG5, OBSCN, FGD2, VAV1, SPTB, ARHGEF10L, ALS2, TIAM1, SOS2, ITSN2, FLJ10357,

Term	Count	%	P-Value	Genes
GO:0005083~small GTPase regulator activity	22	4.05%	1.45E-05	VAV2, KIAA1244, OPHN1, ARHGAP27, CDC42BPG, PLEKHG5, MINK1, OBSCN, FGD2, VAV1, ALS2, TIAM1, ITSN2, NF1, MAP4K1, ARFGEF1, CENTD3, ARHGEF10L, SPTB, SOS2, ARFGEF2, FLJ10357,
GO:0008324~cation transmembrane transporter activity	35	6.45%	3.02E-05	STAT6, SLC4A5, RYR3, TRPM1, ATP2A1, SLC12A1, SLC12A2, SLC15A2, SLC12A5, ATP1A3, CACNA2D1, CACNB2, CACNA1A, TRPM7, ATP1A4, RYR2, CACNA1S, RYR1, CACNA1E, ATP12A, FLJ20433, SLC12A3, TRPM8, CACNA1B, SLC12A4, CNGB1, LOC133308, ATP8A1, CACNA1C, TRPM4, CACNA1F, ATP6V0A4, SLC15A1, ATP1A2,
GO:0004386~helicase activity	16	2.95%	3.49E-05	FLJ20035, ZRANB3, SMARCAD1, DHX37, EP400, INOC1, DHX35, BRIP1, CHD8, DDX54, SKIV2L, POLQ, WRN, DDX21, FANCM, ATRX,
GO:0042623~ATPase activity, coupled	23	4.24%	3.52E-05	MYH3, FLJ20035, ATP1A4, DHX37, MYO1E, MYH7, ATP2A1, DHX35, MYH6, ATP12A, ABCC2, BRIP1, ATP1A3, ATP8A1, DDX54, ABCC4, SKIV2L, MYH4, POLQ, WRN, DDX21, FANCM, ATP1A2,
GO:0015377~cation:chloride symporter activity	5	0.92%	3.66E-05	SLC12A3, SLC12A5, SLC12A4, SLC12A1, SLC12A2,
GO:0005088~Ras guanyl-nucleotide exchange factor activity	12	2.21%	4.49E-05	VAV2, PLEKHG5, OBSCN, FGD2, VAV1, SPTB, ARHGEF10L, ALS2, TIAM1, SOS2, ITSN2, FLJ10357,
GO:0004721~phosphoprotein phosphatase activity	16	2.95%	7.55E-05	PTPRD, PTPRS, PTPN4, PTPRE, PTPRA, PTPN20A, PTPRG, PTPRZ1, PTPRN2, PTPN13, PTPRO, PTPRB, PTPRM, PTPRJ, CAMK2G, PTPRC, PTPRN,
GO:0016773~phosphotransferase activity, alcohol group as acceptor	42	7.73%	1.44E-04	MINK1, RPS6KA2, C9orf39, RET, STK31, TEX14, ULK1, STK32A, MAP4K1, TEK, WNK2, MARK2, NEK1, DGKK, CTTNBP2, LRRK2, TRPM7, ROCK1, PRKDC, BUB1B, TIE1, ULK4, CDC42BPG, OBSCN, WNK3, ROS1, AKT3, PTK2, TTN, BMX, RPS6KA1, CDKL5, ADCK5, PTK7, PASK, MST1R, CAMK2G, ATM, NIN, BUB1, ADRBK2, MYLK,
GO:0003777~microtubule motor activity	11	2.03%	1.53E-04	KIF1A, DNAH1, DNAH5, CENPE, KIF13B, C20orf23, DNAH8, DNHD2, DNAH10, DNAH7, DNAH3,
GO:0016787~hydrolase activity	95	17.50%	3.80E-04	ZRANB3, CAD, DHX37, MYH7, NAALADL1, PTPRE, INOC1, SPAG1, DHX35, TNS1, MYH6, NAALAD2, ABCC2, ENPEP, PAPPA2, CHD8, PTPRO, PTPRJ, DCLRE1C, HDAC10, DDX21, DNAH7, XPNPEP2, PLCE1, ATP1A4, EP400, XPNPEP1, USP37, TTN, PARN, PTPRN2, ODZ3, MMEL1, FLJ20433, BRIP1, ABCC4, SKIV2L, PDE2A, NADSYN1, DNAH10, FANCM, ATP1A2, ASAH2, ADAMTS10, FLJ20035, DNAH5, FLJ32310, SMARCAD1, ATP2A1, ODZ2, DNAH8, ADAMTS12, PTPRG, PTPN20A, ANKHD1, DNAH3, DPP8, ATP1A3, C11orf11, PTPN13, DDX54, OPA1, PTPRM, MYH4, PTPRC, MYH9, ADAM32, PRSS7, DPP10, PTPRN, MYH3, PAPPA, PTPRD, PTPRS, MYO1E, USP47, PTPN4, PTPRA, TLL1, MTHFD1, USP28, ATP12A, PTPRZ1, ATP8A1, ACOT12, PTPRB, ADCK5, WRN, POLQ, CAMK2G, PDE11A, SENP7, C17orf27, CSMD2, PLD1

Term	Count	%	P-Value	Genes
GO:0015075~ion transmembrane transporter activity	40	7.37%	4.16E-04	STAT6, SLC4A5, RYR3, TRPM1, ATP2A1, SLC12A1, SLC12A2, SLC4A3, ABCC2, SLC15A2, SLC12A5, ATP1A3, CACNA2D1, CACNB2, CACNA1A, TRPM7, CLCA1, ATP1A4, RYR2, CACNA1S, SLC4A10, RYR1, CACNA1E, ATP12A, FLJ20433, SLC12A3, TRPM8, CACNA1B, SLC12A4, CNGB1, LOC133308, ATP8A1, ABCC4, CACNA1C, TRPM4, CACNA1F, ATP6V0A4, ATP1A2, SLC15A1, CACNA1D,
GO:0051015~actin filament binding	7	1.29%	4.79E-04	SPTA1, MYO6, MYO5A, MYO5B, MYH9, SPTB, DST,
GO:0015296~anion:cation symporter	6	1.10%	1.07E-03	SLC12A3, SLC12A5, SLC4A5, SLC12A4, SLC12A1, SLC12A2,
GO:0017111~nucleoside-triphosphatase activity	32	5.89%	1.65E-03	FLJ20035, DNAH5, MYH7, DHX37, ATP2A1, DNAH8, INOC1, MYH6, DHX35, ABCC2, DNAH3, ATP1A3, DDX54, OPA1, MYH4, MYH9, DDX21, DNAH7, MYH3, ATP1A4, MYO1E, ATP12A, BRIP1, ATP8A1, ABCC4, SKIV2L, POLQ, WRN, DNAH10, C17orf27,
GO:0016791~phosphoric monoester hydrolase activity	17	3.13%	1.91E-03	PTPRD, PTPRS, PTPN4, PTPRE, PTPRA, PTPN20A, PTPRG, PTPRZ1, PTPRN2, PTPN13, PTPRO, PTPRB, PTPRM, PTPRJ, CAMK2G, PTPRC, PLD1, PTPRN,
GO:0008238~exopeptidase activity	10	1.84%	2.03E-03	FLJ32310, ODZ2, NAALADL1, XPNPEP1, NAALAD2, XPNPEP2, ODZ3, ENPEP, DPP10, DPP8
GO:0005218~intracellular ligand-gated calcium channel activity	3	0.55%	2.24E-03	RYR2, RYR3, RYR1,
GO:0008026~ATP-dependent helicase activity	10	1.84%	2.48E-03	FLJ20035, DDX54, DHX37, SKIV2L, WRN, POLQ, DDX21, DHX35, FANCM, BRIP1,
GO:0046872~metal ion binding	147	27.07%	2.50E-03	ZRANB3, CAD, PAM, NAALADL1, SLC12A1, NOS1, SLC12A2, NAALAD2, PAPPA2, CACNA2D1, DCLRE1C, MARK2, DGKK, SLIT3, TRPM7, MYO9A, PLCE1, MCTP2, CACNA1S, CDC42BPG, ACACA, CMYA3, XPNPEP1, NEBL, TTN, ZNF236, TDRD1, PARN, BMX, PITPNM2, CACNA1B, LRP1, CDH23, NOTCH1, RNF20, NIN, ENO2, CACHD1, THBS3, CACNA1F, ADAMTS10, SLIT1, STAT6, RYR3, ZNF185, VAV1, C20orf12, RET, PTPRG, STK32A, OSBPL1A, C11orf11, BRCA1, SCUBE2, CACNA1A, RSN, XDH, SORL1, PAPPA, MTR, OBSCN, SLC4A10, NOS2A, NELL1, TLL1, CACNA1E, DST, PTPRZ1, RPS6KA1, SUSD1, ZNF291, DNAJA5, SLC12A4, STAT5A, ATP8A1, CACNA1C, SP100, CDH5, PDE11A, ACSL6, MIB1, RNF31, NOTCH3, SPTA1, PXDN, EMR1, RPS6KA2, PKD1L2, CTCFL, ENPEP, NOTCH2, SLC12A5, MYO5A, CACNB2, CENTD3, HDAC10, NFX1, THBS1, XPNPEP2, DNAH7, ROCK1, NOTCH4, ATP1A4, FGD2, COMP, CASZ1, MMEL1, PCLKC, ACO1, DMD, NELL2, MYT1, EP300, ATP1A2, MLL3, FLJ32310, ATP2A1, ADAMTS12, ITSN2, ATP1A3, DUOX2, SGCA, OPA1, NEK1, GNPTAB, ADAM32, UTRN, VAV2, DCHS2, RYR2, UNC13B, RYR1, TRIM37, ATP12A, WHSC1L1,
GO:0042578~phosphoric ester hydrolase activity	20	3.68%	3.05E-03	PTPRD, PTPRS, PTPN4, PTPRA, PTPRE, PTPN20A, PTPRG, PTPRZ1, PTPRN2, PTPN13, PTPRO, PTPRB, PDE2A, PTPRJ, PTPRM, CAMK2G, PTPRC, PDE11A, PLD1, PLCE1, PTPRN
GO:0005261~cation channel activity	17	3.13%	3.11E-03	RYR2, RYR3, CACNA1S, TRPM1, RYR1, CACNA1E, TRPM8, CACNA1B, CNGB1, CACNA2D1, CACNA1C, CACNB2, TRPM4, CACNA1A, TRPM7, CACNA1F, CACNA1D,

Term	Count	%	P-Value	Genes
GO:0016462~pyrophosphatase activity	32	5.89%	3.12E-03	FLJ20035, DNAH5, MYH7, DHX37, ATP2A1, DNAH8, INOC1, MYH6, DHX35, ABCC2, DNAH3, ATP1A3, DDX54, OPA1, MYH4, MYH9, DDX21, DNAH7, MYH3, ATP1A4, MYO1E, ATP12A, BRIP1, ATP8A1, ABCC4, SKIV2L, POLQ, WRN, DNAH10, C17orf27,
GO:0016818~hydrolase activity, acting on acid anhydrides, in phosphorus-containing anhydrides	32	5.89%	3.47E-03	FLJ20035, DNAH5, MYH7, DHX37, ATP2A1, DNAH8, INOC1, MYH6, DHX35, ABCC2, DNAH3, ATP1A3, DDX54, OPA1, MYH4, MYH9, DDX21, DNAH7, MYH3, ATP1A4, MYO1E, ATP12A, BRIP1, ATP8A1, ABCC4, SKIV2L, POLQ, WRN, DNAH10, C17orf27, FANCM, ATP1A2,
GO:0005217~intracellular ligand-gated ion channel activity	4	0.74%	3.81E-03	RYR2, CNGB1, RYR3, RYR1,
GO:0016817~hydrolase activity, acting on acid anhydrides	32	5.89%	4.14E-03	FLJ20035, DNAH5, MYH7, DHX37, ATP2A1, DNAH8, INOC1, MYH6, DHX35, ABCC2, DNAH3, ATP1A3, DDX54, OPA1, MYH4, MYH9, DDX21, DNAH7, MYH3, ATP1A4, MYO1E, ATP12A, BRIP1, ATP8A1, ABCC4, SKIV2L, POLQ, WRN, DNAH10, C17orf27,
GO:0008509~anion transmembrane transporter activity	11	2.03%	4.24E-03	SLC12A3, SLC12A5, SLC4A5, SLC12A4, ABCC4, SLC4A10, SLC12A1, SLC4A3, SLC12A2, ABCC2, CLCA1,
GO:0015294~solute:cation symporter	8	1.47%	4.58E-03	SLC12A3, SLC12A5, SLC4A5, SLC12A4, SLC12A1, SLC12A2, SLC15A1, SLC15A2,
GO:0022892~substrate-specific transporter activity	47	8.66%	5.02E-03	STAT6, SLC4A5, RYR3, XPO1, TRPM1, ATP2A1, SLC12A1, SLC12A2, SLC4A3, PKD1L2, ABCC2, SLC15A2, SLC12A5, ATP1A3, CACNA2D1, CACNB2, CACNA1A, TRPM7, CLCA1, SORL1, ATP1A4, RYR2, CACNA1S, SLC4A10, RYR1, CACNA1E, ATP12A, FLJ20433, CACNA1B, TRPM8, SLC12A3, LRP1, SLC12A4, CNGB1, LOC133308, ATP8A1, ABCC4, CACNA1C, RANBP17, TRPM4, IPO11, CLTC, CACNA1F, ATP6V0A4, ATP1A2, SLC15A1, CACNA1D,
GO:0022891~substrate-specific transmembrane transporter activity	40	7.37%	5.25E-03	STAT6, SLC4A5, RYR3, TRPM1, ATP2A1, SLC12A1, SLC12A2, SLC4A3, ABCC2, SLC15A2, SLC12A5, ATP1A3, CACNA2D1, CACNB2, CACNA1A, TRPM7, CLCA1, ATP1A4, RYR2, CACNA1S, SLC4A10, RYR1, CACNA1E, ATP12A, FLJ20433, SLC12A3, TRPM8, CACNA1B, SLC12A4, CNGB1, LOC133308, ATP8A1, ABCC4, CACNA1C, TRPM4, CACNA1F, ATP6V0A4, ATP1A2, SLC15A1, CACNA1D,

Term	Count	%	P-Value	Genes
GO:0043167~ion binding	147	27.07%	5.39E-03	ZRANB3, CAD, PAM, NAALADL1, SLC12A1, NOS1, SLC12A2, NAALAD2, PAPPA2, CACNA2D1, DCLRE1C, MARK2, DGKK, SLIT3, TRPM7, MYO9A, PLCE1, MCTP2, CACNA1S, CDC42BPG, ACACA, CMYA3, XPNPEP1, NEBL, TTN, ZNF236, TDRD1, PARN, BMX, PITPNM2, CACNA1B, LRP1, CDH23, NOTCH1, RNF20, NIN, ENO2, CACHD1, THBS3, CACNA1F, ADAMTS10, SLIT1, STAT6, RYR3, ZNF185, VAV1, C20orf12, RET, PTPRG, STK32A, OSBPL1A, C11orf11, BRCA1, SCUBE2, CACNA1A, RSN, XDH, SORL1, PAPPA, MTR, OBSCN, SLC4A10, NOS2A, NELL1, TLL1, CACNA1E, DST, PTPRZ1, RPS6KA1, SUSD1, ZNF291, DNAJA5, SLC12A4, STAT5A, ATP8A1, CACNA1C, SP100, CDH5, PDE11A, ACSL6, MIB1, RNF31, NOTCH3, SPTA1, PXDN, EMR1, RPS6KA2, PKD1L2, CTCFL, ENPEP, NOTCH2, SLC12A5, MYO5A, CACNB2, CENTD3, HDAC10, NFX1, THBS1, XPNPEP2, DNAH7, ROCK1, NOTCH4, ATP1A4, FGD2, COMP, CASZ1, MMEL1, PCLKC, ACO1, DMD, NELL2, MYT1, EP300, ATP1A2, MLL3, FLJ32310, ATP2A1, ADAMTS12, ITSN2, ATP1A3, DUOX2, SGCA, OPA1, NEK1, GNPTAB, ADAM32, UTRN, VAV2, DCHS2, RYR2, UNC13B, RYR1, TRIM37, ATP12A, WHSC1L1,
GO:0016301~kinase activity	43	7.92%	5.85E-03	MINK1, RPS6KA2, C9orf39, RET, STK31, TEX14, ULK1, STK32A, MAP4K1, TEK, WNK2, MARK2, NEK1, DGKK, CTTNBP2, LRRK2, TRPM7, ROCK1, PRKDC, BUB1B, TIE1, ULK4, CDC42BPG, OBSCN, WNK3, ROS1, AKT3, PTK2, NELL1, TTN, BMX, RPS6KA1, CDKL5, ADCK5, PTK7, PASK, MST1R, CAMK2G, ATM, NIN, BUB1, ADRBK2, MYLK,
GO:0004713~protein-tyrosine kinase activity	14	2.58%	6.10E-03	TIE1, OBSCN, ROS1, PTK2, RET, TEX14, BMX, TTN, TEK, PTK7, MST1R, NEK1, NIN, CTTNBP2,
GO:0016829~lyase activity	13	2.39%	8.03E-03	UROC1, PAM, ODZ2, PTPRG, STAB2, PTPRZ1, ODZ3, NOTCH2, CHD8, ACO1, ACMSD, ENO2, KIDINS220,
GO:0046873~metal ion transmembrane transporter activity	18	3.31%	8.33E-03	RYR2, RYR3, CACNA1S, TRPM1, ATP2A1, RYR1, CACNA1E, TRPM8, CACNA1B, CNGB1, CACNA2D1, CACNA1C, CACNB2, TRPM4, CACNA1A, TRPM7, CACNA1F, CACNA1D,
GO:0043169~cation binding	134	24.68%	8.45E-03	ZRANB3, CAD, PAM, SLC12A1, NAALADL1, SLC12A2, NOS1, NAALAD2, PAPPA2, CACNA2D1, DGKK, SLIT3, TRPM7, MYO9A, PLCE1, MCTP2, CACNA1S, CDC42BPG, ACACA, CMYA3, XPNPEP1, NEBL, TTN, ZNF236, TDRD1, BMX, PITPNM2, CACNA1B, LRP1, CDH23, NOTCH1, RNF20, NIN, CACHD1, THBS3, CACNA1F, ADAMTS10, SLIT1, STAT6, RYR3, ZNF185, VAV1, C20orf12, RET, PTPRG, OSBPL1A, C11orf11, BRCA1, SCUBE2, CACNA1A, RSN, XDH, SORL1, PAPPA, MTR, SLC4A10, NOS2A, NELL1, TLL1, CACNA1E, DST, PTPRZ1, SUSD1, ZNF291, DNAJA5, SLC12A4, STAT5A, CACNA1C, SP100, CDH5, MIB1, RNF31, NOTCH3, SPTA1, PXDN, EMR1, PKD1L2, CTCFL, ENPEP, NOTCH2, SLC12A5, MYO5A, CACNB2, CENTD3, HDAC10, NFX1, THBS1, XPNPEP2, DNAH7, ROCK1, NOTCH4, ATP1A4, FGD2, COMP, CASZ1, MMEL1, PCLKC, ACO1, DMD, NELL2, MYT1, EP300, ATP1A2, MLL3, FLJ32310, ATP2A1, ADAMTS12, ITSN2, ATP1A3, DUOX2, SGCA, GNPTAB, ADAM32, UTRN, VAV2, DCHS2, RYR2, UNC13B, RYR1, TRIM37, ATP12A, SLC12A3, WHSC1L1, THBS2, ZFP106, NID1, TRPM4, RNF17

Term	Count	%	P-Value	Genes
GO:0004517~nitric-oxide synthase	3	0.55%	1.45E-02	NOS2A, NOS1, NOS3,
GO:0022857~transmembrane transporter activity	41	7.55%	1.62E-02	STAT6, SLC4A5, RYR3, TRPM1, ATP2A1, SLC12A1, SLC12A2, SLC4A3, ABCC2, SLC15A2, NUP133, SLC12A5, ATP1A3, CACNA2D1, CACNB2, CACNA1A, TRPM7, CLCA1, ATP1A4, RYR2, CACNA1S, SLC4A10, RYR1, CACNA1E, ATP12A, FLJ20433, SLC12A3, TRPM8, CACNA1B, SLC12A4, CNGB1, LOC133308, ATP8A1, ABCC4, CACNA1C, TRPM4, CACNA1F, ATP6V0A4, ATP1A2, SLC15A1, CACNA1D,
GO:0030234~enzyme regulator activity	35	6.45%	1.73E-02	COL4A3, OPHN1, MINK1, ARHGAP27, VAV1, RALGPS2, RET, TIAM1, ITSN2, SRGAP1, NF1, COL7A1, MAP4K1, ARFGEF1, ARHGAP17, CENTD3, ARHGEF10L, SPTB, SOS2, LRRK2, THBS1, BIRC6, FLJ10357, PLCE1, VAV2, KIAA1244, PLEKHG5, CDC42BPG, OBSCN, TAGAP, FGD2, ANKRD27, ALS2, ARFGEF2, PZP,
GO:0004714~transmembrane receptor protein tyrosine kinase activity	7	1.29%	1.73E-02	TIE1, TEK, PTK7, ROS1, MST1R, RET, NIN,
GO:0004274~dipeptidyl-peptidase IV activity	3	0.55%	1.90E-02	NAALAD2, DPP10, DPP8,
GO:0016846~carbon-sulfur lyase	4	0.74%	1.92E-02	ODZ2, STAB2, ODZ3, NOTCH2,
GO:0019899~enzyme binding	15	2.76%	2.01E-02	EXOC2, CDK5RAP2, FGD2, ALS2, CEP250, VWF, FMNL1, KIF13B, BRCA1, PTPRC, HDAC10, CTTNBP2, IL31RA, PLCE1, SYTL2,
GO:0016772~transferase activity, transferring phosphorus-containing groups	46	8.47%	2.05E-02	MINK1, RPS6KA2, C9orf39, RET, STK31, TEX14, ULK1, STK32A, TEP1, MAP4K1, TEK, WNK2, MARK2, NEK1, DGKK, GNPTAB, CTTNBP2, LRRK2, TRPM7, ROCK1, PRKDC, BUB1B, TIE1, ULK4, CDC42BPG, OBSCN, WNK3, ROS1, AKT3, PTK2, NELL1, TTN, BMX, RPS6KA1, CDKL5, ADCK5, PTK7, POLQ, PASK, MST1R, CAMK2G, NIN, ATM,
GO:0016788~hydrolase activity, acting on ester bonds	31	5.71%	2.14E-02	ZRANB3, PTPRE, PTPN20A, PTPRG, C11orf11, PTPN13, PTPRO, PTPRM, DCLRE1C, PTPRJ, PTPRC, PTPRN, PLCE1, PTPRD, PTPRS, USP47, PTPN4, PTPRA, USP37, USP28, PARN, PTPRZ1, PTPRN2, FLJ20433, ACOT12, PTPRB, PDE2A, WRN, CAMK2G, PDE11A, PLD1, FANCM,

Term	Count	%	P-Value	Genes
GO:0003824~catalytic activity	187	34.44%	2.19E-02	ZRANB3, CAD, MYH7, PAM, NAALADL1, UGCGL2, NOS1, DHX35, TNS1, NAALAD2, ABCC2, PAPPA2, PTPRO, TEK, DCLRE1C, ACMSD, MARK2, DGKK, DIP2B, TRPM7, UGCGL1, PLCE1, CDC42BPG, ACACA, PYGL, XPNPEP1, AKT3, PTK2, USP37, TTN, BMX, PARN, PTPRN2, FLJ20433, HERC6, ABCC4, OGDHL, MDH1B, NIN, RNF20, ENO2, DNAH10, ADAMTS10, STAT6, FLJ20035, MINK1, SMARCAD1, PTPRG, PTPN20A, RET, ULK1, STK32A, DNAH3, DPP8, C11orf11, PTPN13, DDX54, BRCA1, TARBP1, HECW2, MYH9, XDH, DPP10, PRKDC, MYH3, SORL1, PAPPA, MTR, OBSCN, MYO1E, NOS2A, PTPN4, WNK3, PTPRA, NELL1, TLL1, MTHFD1, PTPRZ1, RPS6KA1, ATP8A1, ACOT12, ADCK5, PTK7, WRN, POLQ, PASK, CAMK2G, PDE11A, ACSL6, BUB1, PLD1, MIB1, PXDN, CTPS2, DHX37, RPS6KA2, PTPRE, OGDH, C9orf39, INOC1, SPAG1, STK31, TEX14, MYH6, ENPEP, NOTCH2, MAP4K1, HACE1, CHD8, MYO5A, ALDH8A1, PTPRJ, WNK2, HDAC10, DDX21, CTTNBP2, NFX1, DNAH7, XPNPEP2, ROCK1, ATP1A4, ULK4, UROC1, EP400, ROS1, UST, ODZ3, MMEL1, BRIP1, ACO1, SKIV2L, PDE2A, MST1R, NADSYN1, MYT1, EP300, PYGB, FANCM, ATP1A2, ASAH2, NEDD4, MLL3, DNAH5
GO:0019992~diacylglycerol binding	6	1.10%	2.31E-02	VAV2, UNC13B, CDC42BPG, VAV1, DGKK, ROCK1,
GO:0022804~active transmembrane transporter activity	19	3.50%	2.31E-02	SLC4A5, ATP1A4, ATP2A1, SLC4A10, SLC12A1, SLC12A2, SLC4A3, ATP12A, ABCC2, SLC15A2, SLC12A5, SLC12A3, ATP1A3, SLC12A4, LOC133308, ATP8A1, ABCC4, ATP1A2, SLC15A1,
GO:0008235~metalloexopeptidase	6	1.10%	2.63E-02	FLJ32310, NAALADL1, XPNPEP1, NAALAD2, XPNPEP2, ENPEP,
GO:0005216~ion channel activity	19	3.50%	2.83E-02	RYR2, RYR3, CACNA1S, TRPM1, RYR1, CACNA1E, TRPM8, CACNA1B, CNGB1, CACNA2D1, ABCC4, CACNA1C, CACNB2, CACNA1A, TRPM4, TRPM7, CACNA1F,
GO:0017022~myosin binding	3	0.55%	2.95E-02	ARFGEF1, ARFGEF2, TTN,
GO:0005391~sodium:potassium-exchanging ATPase activity	3	0.55%	2.95E-02	ATP1A3, ATP1A4, ATP1A2,
GO:0000287~magnesium ion binding	19	3.50%	3.14E-02	ATP1A4, CDC42BPG, OBSCN, ATP2A1, RPS6KA2, PARN, ATP12A, RPS6KA1, STK32A, ATP1A3, ATP8A1, OPA1, DCLRE1C, MARK2, NEK1, ACSL6, ENO2, ATP1A2, MYLK,
GO:0004180~carboxypeptidase activity	5	0.92%	3.26E-02	FLJ32310, ODZ2, NAALADL1, NAALAD2, ODZ3,
GO:0022838~substrate specific channel activity	19	3.50%	3.36E-02	RYR2, RYR3, CACNA1S, TRPM1, RYR1, CACNA1E, TRPM8, CACNA1B, CNGB1, CACNA2D1, ABCC4, CACNA1C, CACNB2, CACNA1A, TRPM4, TRPM7, CACNA1F, CACNA1D, CLCA1,
GO:0005100~Rho GTPase activator	4	0.74%	3.39E-02	OPHN1, ARHGAP27, CENTD3, ARHGEF10L,
GO:0015662~ATPase activity, coupled to transmembrane movement of ions, phosphorylative mechanism	6	1.10%	3.53E-02	ATP1A3, ATP1A4, ATP8A1, ATP2A1, ATP12A, ATP1A2,

Term	Count	%	P-Value	Genes
GO:0022803~passive transmembrane transporter activity	20	3.68%	3.62E-02	RYR2, RYR3, CACNA1S, TRPM1, RYR1, CACNA1E, NUP133, TRPM8, CACNA1B, CNGB1, CACNA2D1, ABCC4, CACNA1C, CACNB2, CACNA1A, TRPM4, TRPM7, CACNA1F, CACNA1D, CLCA1,
GO:0015267~channel activity	20	3.68%	3.62E-02	RYR2, RYR3, CACNA1S, TRPM1, RYR1, CACNA1E, NUP133, TRPM8, CACNA1B, CNGB1, CACNA2D1, ABCC4, CACNA1C, CACNB2, CACNA1A, TRPM4, TRPM7, CACNA1F, CACNA1D, CLCA1,
GO:0001633~secretin-like receptor	4	0.74%	3.73E-02	BAI3, CRHR2, BAI1, BAI2,
GO:0005178~integrin binding	5	0.92%	4.01E-02	VWF, COL4A3, TNXB, DST, COL16A1,
GO:0042803~protein homodimerization activity	10	1.84%	4.09E-02	VWF, MYO6, MYH7, NOS2A, PTPRE, MYH9, RNF17, ALS2, DSCAML1, RSN,
GO:0031267~small GTPase binding	6	1.10%	4.38E-02	EXOC2, FMNL1, FGD2, ALS2, SYTL2, PLCE1,
GO:0016879~ligase activity, forming carbon-nitrogen bonds	12	2.21%	4.44E-02	CTPS2, CAD, HACE1, HERC6, ACACA, BRCA1, HECW2, NADSYN1, MTHFD1, BIRC6, FTS, NEDD4,
GO:0016874~ligase activity	18	3.31%	5.34E-02	CTPS2, CAD, ACACA, PCCA, MTHFD1, ACSS1, HERC6, HACE1, BRCA1, HECW2, NADSYN1, RNF20, ACSL6, NFX1, MIB1, BIRC6, FTS, NEDD4,
GO:0005215~transporter activity	51	9.39%	5.35E-02	XPO1, SLC12A1, SLC12A2, SLCO6A1, PKD1L2, ABCC2, SLC15A2, SLC12A5, CACNA2D1, CACNB2, TRPM7, SYTL2, CLCA1, ATP1A4, CACNA1S, FLJ20433, CACNA1B, LRP1, LOC133308, ABCC4, IPO11, CLTC, CACNA1F, SLC15A1, ATP1A2, ATP6V0A4, STAT6, SLC4A5, RYR3, TCOF1, ATP2A1, TRPM1, SLC4A3, NUP133, ATP1A3, CACNA1A, SORL1, RYR2, SLC4A10, RYR1, CACNA1E, ATP12A, SLC12A3, TRPM8, SLC12A4, CNGB1, ATP8A1, CACNA1C, RANBP17, TRPM4, CACNA1D,
GO:0003980~UDP-glucose:glycoprotein glucosyltransferase	2	0.37%	5.44E-02	UGCGL2, UGCGL1,
GO:0008511~sodium:potassium:chloride symporter activity	2	0.37%	5.44E-02	SLC12A1, SLC12A2,
GO:0015333~peptide:hydrogen symporter activity	2	0.37%	5.44E-02	SLC15A1, SLC15A2,
GO:0016980~creatinase activity	2	0.37%	5.44E-02	XPNPEP1, XPNPEP2,
GO:0030020~extracellular matrix structural constituent conferring tensile	2	0.37%	5.44E-02	COL12A1, COL11A2,
GO:0022897~proton-dependent peptide secondary active transmembrane transporter activity	2	0.37%	5.44E-02	SLC15A1, SLC15A2,

Term	Count	%	P-Value	Genes
GO:0016806~dipeptidyl-peptidase and tripeptidyl-peptidase activity	3	0.55%	5.55E-02	NAALAD2, DPP10, DPP8,
GO:0008239~dipeptidyl-peptidase	3	0.55%	5.55E-02	NAALAD2, DPP10, DPP8,
GO:0030955~potassium ion binding	8	1.47%	5.60E-02	ATP1A3, SLC12A5, ATP1A4, SLC12A4, SLC12A1, SLC12A2, ATP12A, ATP1A2,
GO:0022843~voltage-gated cation channel activity	9	1.66%	6.18E-02	CACNA1B, CACNA2D1, CACNA1S, CACNA1C, CACNB2, CACNA1A, CACNA1E, CACNA1F, CACNA1D,
GO:0015106~bicarbonate transmembrane transporter activity	3	0.55%	6.29E-02	SLC4A5, SLC4A10, SLC4A3,
GO:0010181~FMN binding	3	0.55%	6.29E-02	NOS2A, NOS1, NOS3,
GO:0031402~sodium ion binding	7	1.29%	6.30E-02	ATP1A3, SLC12A3, ATP1A4, SLC4A10, SLC12A1, SLC12A2, ATP1A2,
GO:0051020~GTPase binding	6	1.10%	6.42E-02	EXOC2, FMNL1, FGD2, ALS2, SYTL2, PLCE1,
GO:0005539~glycosaminoglycan	7	1.29%	7.04E-02	THBS2, TNXB, STAB2, THBS3, COL5A1, THBS1, ODZ1,
GO:0015301~anion:anion antiporter	3	0.55%	7.06E-02	SLC4A5, SLC4A10, SLC4A3,
GO:0046983~protein dimerization activity	15	2.76%	7.15E-02	NOTCH4, MYO6, MYH7, NOS2A, PTPRE, ROBO2, ALS2, DSCAML1, MYO7A, NOTCH2, VWF, MYH9, RNF17, RSN, TCF4,
GO:0015108~chloride transmembrane transporter activity	3	0.55%	7.86E-02	SLC4A5, SLC4A10, SLC4A3,
GO:0008201~heparin binding	6	1.10%	7.92E-02	THBS2, TNXB, THBS3, COL5A1, THBS1, ODZ1,
GO:0043275~glutamate carboxypeptidase II activity	2	0.37%	8.05E-02	NAALADL1, NAALAD2,
GO:0004591~oxoglutarate dehydrogenase (succinyl-transferring)	2	0.37%	8.05E-02	OGDHL, OGDH,
GO:0030247~polysaccharide binding	7	1.29%	8.11E-02	THBS2, TNXB, STAB2, THBS3, COL5A1, THBS1, ODZ1,
GO:0003678~DNA helicase activity	4	0.74%	8.66E-02	WRN, INOC1, ATRX, BRIP1,
GO:0015293~symporter activity	9	1.66%	8.69E-02	SLC12A3, SLC12A5, SLC4A5, SLC12A4, SLC4A10, SLC12A1, SLC12A2, SLC15A1, SLC15A2
GO:0016836~hydro-lyase activity	5	0.92%	8.75E-02	UROC1, ACO1, PTPRG, ENO2, PTPRZ1,
GO:0017016~Ras GTPase binding	5	0.92%	9.16E-02	EXOC2, FMNL1, ALS2, SYTL2, PLCE1,
GO:0004177~aminopeptidase activity	4	0.74%	9.75E-02	XPNPEP1, XPNPEP2, ENPEP, DPP8,
GO:0042626~ATPase activity, coupled to transmembrane movement of	8	1.47%	9.84E-02	ATP1A3, ATP1A4, ATP8A1, ABCC4, ATP2A1, ATP12A, ATP1A2, ABCC2,
GO:0008289~lipid binding	19	3.50%	9.86E-02	VAV2, MCTP2, UNC13B, CDC42BPG, VAV1, TIAM1, PITPNM2, OSBPL1A, TECTA, PLEKHA4, C20orf23, CNTN2, CENTD3, DGKK, PLD1, XPNPEP2, ROCK1, CNTN3,

Term	Count	%	P-Value	Genes
**Biological Process Category**				
GO:0015698~inorganic anion transport	28	5.16%	5.76E-14	COL4A3, COL17A1, SLC4A5, SLC12A1, SLC12A2, COL9A1, COL7A1, SLC12A5, COL1A2, COL2A1, CTTNBP2, COL9A2, COL3A1, COL20A1, SLC4A10, COL4A2, COL22A1, COL4A4, COL4A1, COL5A1, COL16A1, SLC12A3, SLC12A4, COL4A5, COL5A2, COL12A1, COL1A1, COL11A2,
GO:0051179~localization	154	28.36%	1.04E-13	VPS13B, COL4A3, COL17A1, XPO1, MAMDC4, MYH7, SLC12A1, SLC12A2, SLCO6A1, TNS1, MYO7A, ABCC2, CACNA2D1, COL1A2, COL2A1, VPS13D, SLIT3, TRPM7, SYTL2, COL9A2, EXOC2, DNAH1, CACNA1S, NEBL, UNC13D, KTN1, COL5A1, PITPNM2, CACNA1B, LRP1, LOC133308, ABCC4, KIF13B, IPO11, NIN, ARFGEF2, DNAH10, CACHD1, THBS3, CACNA1F, ATP6V0A4, SLIT1, HSPG2, RYR3, ARHGAP27, TCOF1, VAV1, SLC4A3, RET, NF1, COL7A1, DNAH3, OSBPL1A, VPS13A, ARFGEF1, CENPE, CNTN2, CACNA1A, MYH9, SNX14, MYH3, SORL1, MYO1E, SLC4A10, COL4A2, PACS1, COL4A4, NFKBIL2, CACNA1E, DST, PLXNA3, SLC12A4, COL4A5, CNGB1, ATP8A1, CACNA1C, RANBP17, CAMK2G, MIB1, UACA, OPHN1, CHL1, MYH6, PKD1L2, ENPEP, COL9A1, SLC15A2, SLC12A5, MYO5A, NPHS1, CLEC7A, CACNB2, CENTD3, CTTNBP2, THBS1, DNAH7, NCBP1, CLCA1, COL20A1, ATP1A4, VPS13C, COL22A1, LYST, ANKRD27, ALS2, TNR, COL4A1, COG7, TSNARE1, COL5A2, COL12A1, MST1R, CLTC, COL11A2, ATP1A2, SLC15A1, SLC4A5, DNAH5, TRPM1, ATP2A1, DNAH8, ROBO2, ITSN2, STAB2, NUP133, ATP1A3, OPA1, MYH4, PTPRC, VAV2, COL3A1, KIF1A, TIE1
GO:0022610~biological adhesion	61	11.23%	4.26E-13	COL4A3, COL17A1, MUC4, DCBLD2, EMR1, CHL1, BAI1, CNTNAP5, COL9A1, TECTA, NPHS1, TRPM7, THBS1, CLCA1, COL20A1, COMP, COL22A1, TTN, TNR, COL5A1, DSCAML1, VWF, PCLKC, CDH23, MYBPC1, COL12A1, NELL2, THBS3, COL11A2, HSPG2, CDON, VAV1, ZAN, RET, ROBO2, USH2A, STAB2, NF1, COL7A1, MYBPC3, SIGLEC1, PTPRM, CNTN2, CNTN3, ADAM32, DCHS2, PTPRD, PTPRS, NPHP1, TNXB, NELL1, DST, ENG, COL16A1, NEO1, THBS2, STAT5A, PTK7, CDH5, NID1, SDK2,
GO:0007155~cell adhesion	61	11.23%	4.26E-13	COL4A3, COL17A1, MUC4, DCBLD2, EMR1, CHL1, BAI1, CNTNAP5, COL9A1, TECTA, NPHS1, TRPM7, THBS1, CLCA1, COL20A1, COMP, COL22A1, TTN, TNR, COL5A1, DSCAML1, VWF, PCLKC, CDH23, MYBPC1, COL12A1, NELL2, THBS3, COL11A2, HSPG2, CDON, VAV1, ZAN, RET, ROBO2, USH2A, STAB2, NF1, COL7A1, MYBPC3, SIGLEC1, PTPRM, CNTN2, CNTN3, ADAM32, DCHS2, PTPRD, PTPRS, NPHP1, TNXB, NELL1, DST, ENG, COL16A1, NEO1, THBS2, STAT5A, PTK7, CDH5, NID1, SDK2,
GO:0006820~anion transport	29	5.34%	1.32E-12	COL4A3, COL17A1, SLC4A5, SLC12A1, SLC4A3, SLC12A2, COL9A1, COL7A1, SLC12A5, COL1A2, COL2A1, CTTNBP2, COL9A2, COL3A1, COL20A1, SLC4A10, COL4A2, COL22A1, COL4A4, COL5A1, COL4A1, COL16A1, SLC12A3, SLC12A4, COL4A5, COL5A2, COL12A1, COL1A1, COL11A2,

Term	Count	%	P-Value	Genes
GO:0007010~cytoskeleton organization and biogenesis	47	8.66%	5.06E-12	SPTA1, OPHN1, DNAH5, MYH7, NEB, DNAH8, MYH6, MYO7A, NF1, DNAH3, FMNL1, MYO5A, CENPE, BRCA1, OPA1, MYH4, CENTD3, MYH9, ARHGEF10L, SPTB, CTTNBP2, DNAH7, TRPM7, ROCK1, PLCE1, BUB1B, MYH3, KIF1A, MYH11, DNAH1, MYO6, CDC42BPG, MYO1E, FGD2, NPHP1, TNXB, NEBL, CEP250, TTN, DST, KTN1, SHANK1, KIF13B, C20orf23, DMD, DNHD2, DNAH10,
GO:0006941~striated muscle contraction	14	2.58%	9.60E-12	MYH3, MYH11, MYH7, ATP2A1, MYH2, NOS1, MYH6, TTN, MYBPC3, MYBPC1, MYH1, MYH4, MYH8, ATP1A2,
GO:0006817~phosphate transport	20	3.68%	9.74E-12	COL9A2, COL4A3, COL3A1, COL17A1, COL20A1, COL4A2, COL22A1, COL4A4, COL5A1, COL4A1, COL16A1, COL9A1, COL7A1, COL4A5, COL1A2, COL5A2, COL2A1, COL12A1, COL1A1, COL11A2,
GO:0030705~cytoskeleton-dependent intracellular transport	23	4.24%	1.10E-11	MYH3, KIF1A, DNAH1, MYO6, DNAH5, MYO1E, MYH7, DNAH8, DST, KTN1, MYH6, MYO7A, DNAH3, MYO5A, CENPE, KIF13B, OPA1, MYH4, C20orf23, MYH9, DNHD2, DNAH10, DNAH7,
GO:0016043~cellular component organization and biogenesis	133	24.49%	1.21E-11	XPO1, DCBLD2, PCNT, MYH7, NEB, PAM, BAI1, TNS1, MYO7A, PAPPA2, MARK2, SLIT3, TRPM7, SYTL2, PLCE1, EXOC2, DNAH1, CDC42BPG, PTK2, NEBL, TTN, UNC13D, KTN1, SMARCC2, CACNA1B, LRP1, KIF13B, IPO11, RNF20, NIN, ARFGEF2, DNAH10, CACNA1F, SLIT1, ARHGAP27, SMARCAD1, VAV1, NF1, ULK1, DNAH3, MYH10, FMNL1, VPS13A, ARFGEF1, CENPE, BRCA1, MYH9, SYNE1, MYH3, SORL1, MYH11, MYO1E, COL4A2, PACS1, COL4A4, NFKBIL2, DST, PLXNA3, PTPRZ1, PTK7, RANBP17, NFKB2, WRN, MIB1, UACA, SPTA1, OPHN1, CHL1, INOC1, TIAM1, MYH6, PKD1L2, NOTCH2, SYCP2, CHD8, MYO5A, CLEC7A, CACNB2, CENTD3, HDAC10, ARHGEF10L, SPTB, CTTNBP2, DNAH7, ROCK1, NCBP1, STAG3, EP400, FGD2, LYST, ANKRD27, ALS2, CEP250, TNR, DSCAML1, VWF, PCLKC, COL5A2, DMD, COL12A1, EP300, CLTC, COL11A2, SYCP1, MLL3, DNAH5, CDON, PB1, DNAH8, ROBO2, ITSN2, STAB2, NUP133, TEP1, OPA1, MYH4, ARID1B, BUB1B, VAV2, TIE1, KIF1A, MYO6, UNC13B, MYO1A, NPHP1, RYR1, TNXB, NEO1, SHANK1, WHSC1L1, CCDC40, C20orf23
GO:0048731~system development	95	17.50%	2.20E-10	COL4A3, COL17A1, OPHN1, MYH7, NEB, CHL1, PPL, BAI1, TIAM1, MYH6, SFTPB, MYO7A, ODZ1, COL9A1, ENPEP, NOTCH2, MAP4K1, COL1A2, PTPRJ, COL2A1, CACNB2, SLIT3, CTTNBP2, THBS1, IL31RA, PLCE1, TTC7A, NOTCH4, COL9A2, CDK5RAP2, COMP, KIAA1217, ALS2, SOX5, TTN, TNR, BMX, DSCAML1, DRB1, COL5A2, DMD, CRHR2, COL12A1, NOTCH1, MYT1, EP300, COL11A2, CACNA1F, ATP6V0A4, SLIT1, STAT6, TCOF1, CDON, USH2A, ROBO2, RET, STAB2, NF1, ULK1, COL7A1, MYBPC3, OSBPL1A, SGCA, CNTN2, PTPRC, MYH9, UTRN, PRKDC, HHIP, MYH3, COL3A1, TIE1, MYH11, MTR, MYO6, COL4A2, RYR1, NELL1, COL4A4, TLL1, PLXNA3, ENG, PTPRZ1, NEO1, STAT5A, PTK7, CDH5, NFKB2, COL1A1, ACSL6, NOS3,

Term	Count	%	P-Value	Genes
GO:0048856~anatomical structure development	109	20.07%	2.69E-10	COL4A3, COL17A1, PCNT, DCBLD2, MYH7, NEB, BAI1, MYO7A, SFTPB, PAPPA2, COL1A2, COL2A1, MARK2, SLIT3, PLCE1, IL31RA, COL9A2, BMX, TTN, SOX5, NOTCH1, CACNA1F, SLIT1, ATP6V0A4, STAT6, TCOF1, RET, USH2A, NF1, ULK1, COL7A1, OSBPL1A, MYBPC3, MYH10, CNTN2, MYH9, PRKDC, XDH, MYH3, MTR, MYH11, COL4A2, TLL1, COL4A4, NELL1, PTPRZ1, PLXNA3, STAT5A, PTK7, CDH5, NFKB2, ACSL6, NOTCH3, MIB1, L1CAM, SPTA1, OPHN1, CHL1, PPL, TIAM1, MYH6, ODZ1, ENPEP, COL9A1, NOTCH2, MAP4K1, PTPRJ, CENTD3, CACNB2, CTTNBP2, THBS1, TTC7A, NOTCH4, CDK5RAP2, FGD2, KIAA1217, COMP, ALS2, TNR, DSCAML1, DRB1, PCLKC, COL5A2, DMD, COL12A1, CRHR2, MYT1, EP300, COL11A2, CDON, ROBO2, STAB2, DUOX2, SGCA, PTPRC, UTRN, HHIP, VAV2, COL3A1, TIE1, MYO6
GO:0048513~organ development	76	14.00%	4.09E-10	COL4A3, COL17A1, MYH7, NEB, PPL, MYH6, SFTPB, MYO7A, COL9A1, ENPEP, NOTCH2, MAP4K1, COL1A2, PTPRJ, COL2A1, CACNB2, SLIT3, CTTNBP2, THBS1, IL31RA, PLCE1, TTC7A, NOTCH4, COL9A2, CDK5RAP2, COMP, KIAA1217, ALS2, SOX5, TTN, BMX, DSCAML1, COL5A2, DMD, NOTCH1, CRHR2, COL12A1, EP300, COL11A2, ATP6V0A4, SLIT1, TCOF1, CDON, ROBO2, RET, USH2A, STAB2, NF1, COL7A1, MYBPC3, OSBPL1A, SGCA, PTPRC, MYH9, UTRN, PRKDC, HHIP, MYH3, COL3A1, TIE1, MYH11, COL4A2, RYR1, COL4A4, TLL1, PLXNA3, ENG, NEO1, STAT5A,
GO:0006810~transport	127	23.39%	5.33E-10	VPS13B, COL4A3, COL17A1, XPO1, MAMDC4, MYH7, SLC12A1, SLC12A2, SLCO6A1, MYO7A, ABCC2, CACNA2D1, COL1A2, COL2A1, TRPM7, SYTL2, COL9A2, EXOC2, DNAH1, CACNA1S, NEBL, KTN1, UNC13D, COL5A1, PITPNM2, CACNA1B, LRP1, LOC133308, ABCC4, KIF13B, IPO11, ARFGEF2, DNAH10, CACHD1, CACNA1F, ATP6V0A4, RYR3, ARHGAP27, TCOF1, VAV1, SLC4A3, NF1, COL7A1, DNAH3, OSBPL1A, VPS13A, ARFGEF1, CENPE, CACNA1A, MYH9, SNX14, MYH3, SORL1, MYO1E, SLC4A10, COL4A2, PACS1, COL4A4, NFKBIL2, CACNA1E, DST, SLC12A4, COL4A5, CNGB1, ATP8A1, CACNA1C, RANBP17, CAMK2G, MIB1, UACA, MYH6, PKD1L2, COL9A1, SLC15A2, SLC12A5, MYO5A, CLEC7A, CACNB2, CENTD3, CTTNBP2, DNAH7, NCBP1, CLCA1, ATP1A4, COL20A1, COL22A1, LYST, ANKRD27, ALS2, COL4A1, COG7, TSNARE1, COL5A2, COL12A1, CLTC, COL11A2, SLC15A1, ATP1A2, SLC4A5, DNAH5, TRPM1, ATP2A1, DNAH8, ITSN2, STAB2, NUP133, ATP1A3, OPA1, MYH4, COL3A1, KIF1A, RYR2, MYO6, UNC13B, RYR1, MYO1C, ATP12A, ENG,
GO:0006811~ion transport	57	10.50%	8.32E-10	COL4A3, COL17A1, SLC12A1, SLC12A2, COL9A1, SLC12A5, CACNA2D1, COL1A2, COL2A1, CACNB2, CTTNBP2, TRPM7, COL9A2, ATP1A4, COL20A1, CACNA1S, COL22A1, NEBL, COL5A1, COL4A1, CACNA1B, ABCC4, COL5A2, COL12A1, CACHD1, CACNA1F, COL11A2, ATP1A2, ATP6V0A4, SLC4A5, RYR3, TRPM1, ATP2A1, SLC4A3, COL7A1, ATP1A3, CACNA1A, COL3A1, RYR2, SLC4A10, COL4A2, RYR1, CACNA1E, COL4A4, ATP12A, COL16A1, SLC12A3, TRPM8, SLC12A4, COL4A5, CNGB1, ATP8A1, CACNA1C, CAMK2G, COL1A1, TRPM4, CACNA1D,

Term	Count	%	P-Value	Genes
GO:0051234~establishment of localization	129	23.76%	1.14E-09	VPS13B, COL4A3, COL17A1, XPO1, MAMDC4, MYH7, SLC12A1, SLC12A2, SLCO6A1, MYO7A, ABCC2, CACNA2D1, COL1A2, COL2A1, TRPM7, SYTL2, COL9A2, EXOC2, DNAH1, CACNA1S, NEBL, KTN1, UNC13D, COL5A1, PITPNM2, CACNA1B, LRP1, LOC133308, ABCC4, KIF13B, IPO11, ARFGEF2, DNAH10, CACHD1, CACNA1F, ATP6V0A4, RYR3, ARHGAP27, TCOF1, VAV1, SLC4A3, NF1, COL7A1, DNAH3, OSBPL1A, VPS13A, ARFGEF1, CENPE, CACNA1A, MYH9, SNX14, MYH3, SORL1, MYO1E, SLC4A10, COL4A2, PACS1, COL4A4, NFKBIL2, CACNA1E, DST, SLC12A4, COL4A5, CNGB1, ATP8A1, CACNA1C, RANBP17, CAMK2G, MIB1, UACA, MYH6, PKD1L2, COL9A1, SLC15A2, SLC12A5, MYO5A, NPHS1, CLEC7A, CACNB2, CENTD3, CTTNBP2, DNAH7, NCBP1, CLCA1, ATP1A4, COL20A1, COL22A1, LYST, ANKRD27, ALS2, COL4A1, COG7, TSNARE1, COL5A2, COL12A1, CLTC, COL11A2, SLC15A1, ATP1A2, SLC4A5, DNAH5, TRPM1, ATP2A1, DNAH8, ITSN2, STAB2, NUP133, ATP1A3, OPA1, MYH4, COL3A1, KIF1A, RYR2, MYO6, UNC13B, NPHP1, RYR1, MYO1C, ATP12A, ENG, COL16A1, SLC12A3, TRPM8, CCDC40, C20orf23, COL1A1, TRPM4,
GO:0003012~muscle system process	23	4.24%	1.40E-09	MYH3, RYR2, MYH11, CACNA1S, MYH7, ATP2A1, RYR1, NOS1, MYH2, TTN, MYH6, MYBPC3, PPP1R12A, MYBPC1, MYH1, SLMAP, SGCA, MYH4, DMD, MYH8, ATP1A2, UTRN, PLCE1,
GO:0006936~muscle contraction	23	4.24%	1.40E-09	MYH3, RYR2, MYH11, CACNA1S, MYH7, ATP2A1, RYR1, NOS1, MYH2, TTN, MYH6, MYBPC3, PPP1R12A, MYBPC1, MYH1, SLMAP, SGCA, MYH4, DMD, MYH8, ATP1A2, UTRN, PLCE1,
GO:0030029~actin filament-based process	26	4.79%	4.26E-09	SPTA1, OPHN1, MYH7, NEB, MYH6, NF1, MYO7A, FMNL1, MYO5A, MYH4, SPTB, ARHGEF10L, MYH9, TRPM7, ROCK1, MYH3, MYH11, MYO6, CDC42BPG, MYO1E, NPHP1, FGD2, TNXB, NEBL, DST, TTN,
GO:0030048~actin filament-based movement	9	1.66%	1.93E-08	MYH3, MYO6, MYO5A, MYH7, MYO1E, MYH4, MYH9, MYH6, MYO7A,
GO:0006996~organelle organization and biogenesis	67	12.34%	5.21E-08	SPTA1, OPHN1, MYH7, NEB, PAM, INOC1, MYH6, MYO7A, SYCP2, CHD8, MYO5A, CENTD3, HDAC10, ARHGEF10L, SPTB, CTTNBP2, TRPM7, DNAH7, ROCK1, PLCE1, STAG3, DNAH1, CDC42BPG, EP400, FGD2, NEBL, ALS2, CEP250, TTN, KTN1, SMARCC2, KIF13B, DMD, RNF20, DNAH10, EP300, SYCP1, MLL3, DNAH5, SMARCAD1, PB1, DNAH8, NF1, DNAH3, TEP1, FMNL1, CENPE, BRCA1, OPA1, MYH4, MYH9, ARID1B, SYNE1, BUB1B, MYH3, KIF1A, MYH11, MYO6, MYO1E, NPHP1, TNXB, DST,

Term	Count	%	P-Value	Genes
GO:0032502~developmental process	137	25.23%	1.42E-07	COL4A3, COL17A1, PCNT, DCBLD2, MYH7, NEB, BAI1, SFTPB, MYO7A, PAPPA2, COL1A2, COL2A1, MARK2, SLIT3, PLCE1, IL31RA, COL9A2, SOX5, TTN, BMX, LRP1, MYBPC1, NOTCH1, CACNA1F, ATP6V0A4, SLIT1, STAT6, MINK1, TCOF1, NFKB1, RET, USH2A, NF1, ULK1, CARD14, COL7A1, MYBPC3, OSBPL1A, MYH10, BRCA1, CNTN2, CACNA1A, MYH9, SYNE1, XDH, PRKDC, MYH3, PAPPA, MTR, MYH11, OBSCN, COL4A2, ODF2, NELL1, COL4A4, TLL1, PLXNA3, PTPRZ1, STAT5A, CCAR1, PTK7, CDH5, NFKB2, WRN, ACSL6, MIB1, NOTCH3, L1CAM, UACA, SPTA1, OPHN1, CHL1, PPL, TIAM1, MYH6, KIAA1967, ODZ1, ENPEP, COL9A1, NOTCH2, MAP4K1, CHD8, PTPRJ, CACNB2, CENTD3, CTTNBP2, THBS1, ROCK1, TTC7A, NOTCH4, CDK5RAP2, FGD2, COMP, KIAA1217, ALS2, TNR, DSCAML1, DRB1, PCLKC, COL5A2, DMD, CRHR2, COL12A1, MST1R, MYT1, EP300, COL11A2, ASAH2, CDON, ROBO2, STAB2, DUOX2, OPA1, SGCA, PTPRC, GNPTAB, BIRC6, UTRN, BUB1B, HHIP, VAV2, COL3A1, TIE1, MYO6, UNC13B, MUC2, PIWIL2, MYO1A, RYR1, ENG, NEO1, WHSC1L1
GO:0006816~calcium ion transport	17	3.13%	1.48E-07	RYR2, RYR3, CACNA1S, ATP2A1, RYR1, CACNA1E, CACNA1B, CACNA2D1, CACNA1C, CACNB2, CAMK2G, CACNA1A, TRPM4, CACHD1, TRPM7, CACNA1F,
GO:0007605~sensory perception of sound	18	3.31%	2.29E-07	COL4A3, ALMS1, MYO6, MYO1A, TMC1, USH2A, MYO7A, TECTA, CDH23, COL1A2, OPA1, COL2A1, COL1A1, MYH9, COL11A2, ATP6V0A4, CACNA1D, ATRX,
GO:0050954~sensory perception of mechanical stimulus	18	3.31%	2.55E-07	COL4A3, ALMS1, MYO6, MYO1A, TMC1, USH2A, MYO7A, TECTA, CDH23, COL1A2, OPA1, COL2A1, COL1A1, MYH9, COL11A2, ATP6V0A4, CACNA1D, ATRX,
GO:0007275~multicellular organismal development	106	19.52%	2.61E-07	COL4A3, COL17A1, MYH7, NEB, BAI1, MYO7A, SFTPB, COL1A2, COL2A1, MARK2, SLIT3, PLCE1, IL31RA, COL9A2, BMX, TTN, SOX5, LRP1, MYBPC1, NOTCH1, CACNA1F, SLIT1, ATP6V0A4, STAT6, MINK1, TCOF1, RET, USH2A, NF1, ULK1, COL7A1, OSBPL1A, MYBPC3, CNTN2, MYH9, PRKDC, MYH3, MTR, MYH11, OBSCN, COL4A2, ODF2, TLL1, COL4A4, NELL1, PTPRZ1, PLXNA3, STAT5A, PTK7, CDH5, WRN, NFKB2, ACSL6, NOTCH3, MIB1, L1CAM, OPHN1, CHL1, PPL, TIAM1, MYH6, ENPEP, COL9A1, ODZ1, NOTCH2, MAP4K1, CHD8, PTPRJ, CACNB2, CTTNBP2, THBS1, TTC7A, NOTCH4, CDK5RAP2, COMP, KIAA1217, ALS2, TNR, DSCAML1, DRB1, COL5A2, DMD, COL12A1, MST1R, CRHR2, MYT1, EP300, COL11A2, CDON, ROBO2, STAB2, SGCA, PTPRC, UTRN, HHIP, COL3A1, TIE1, MYO6, PIWIL2, RYR1, ENG, NEO1, AFF3, COL1A1
GO:0043687~post-translational protein modification	76	14.00%	5.77E-07	PTPRE, RPS6KA2, STK31, TEX14, MAP4K1, HACE1, PTPRO, TEK, PTPRJ, WNK2, MARK2, HDAC10, CTTNBP2, NFX1, TRPM7, ROCK1, IL31RA, ULK4, CDC42BPG, ROS1, AKT3, PTK2, USP37, UST, TTN, BMX, PTPRN2, HERC6, MST1R, RNF20, NIN, EP300, NEDD4, MINK1, PTPN20A, RET, PTPRG, ULK1, CARD14, STK32A, PTPN13, BRCA1, PTPRM, HECW2, PTPRC, NEK1, LRRK2, BIRC6, PTPRN, BUB1B, TIE1, PTPRD, PTPRS, OBSCN, USP47, WNK3, PTPN4, RYR1, PTPRA, USP28, PTPRZ1, RPS6KA1, WHSC1L1, CDKL5, PTPRB, ADCK5, PTK7, NID1, PASK, CAMK2G, SENP7, BUB1, C17orf27, MIB1,

Term	Count	%	P-Value	Genes
GO:0035023~regulation of Rho protein signal transduction	14	2.58%	7.79E-07	VAV2, PLEKHG5, ARHGAP27, OBSCN, VAV1, FGD2, TIAM1, ALS2, ITSN2, CENTD3, ARHGEF10L, SPTB, SOS2, FLJ10357,
GO:0051674~localization of cell	32	5.89%	8.33E-07	OPHN1, DNAH5, CHL1, DNAH8, ROBO2, RET, TNS1, NF1, ENPEP, DNAH3, ATP1A3, CNTN2, CENTD3, MYH9, SLIT3, CTTNBP2, DNAH7, THBS1, VAV2, ATP1A4, TIE1, DNAH1, MUC2, RYR1, TNR, PLXNA3, NEO1, MST1R, NOS3, THBS3, SLIT1, ATP1A2,
GO:0006928~cell motility	32	5.89%	8.33E-07	OPHN1, DNAH5, CHL1, DNAH8, ROBO2, RET, TNS1, NF1, ENPEP, DNAH3, ATP1A3, CNTN2, CENTD3, MYH9, SLIT3, CTTNBP2, DNAH7, THBS1, VAV2, ATP1A4, TIE1, DNAH1, MUC2, RYR1, TNR, PLXNA3, NEO1, MST1R, NOS3, THBS3, SLIT1, ATP1A2,
GO:0009653~anatomical structure morphogenesis	61	11.23%	9.46E-07	COL4A3, SPTA1, OPHN1, PCNT, DCBLD2, MYH7, CHL1, PPL, BAI1, TIAM1, MYH6, MYO7A, SFTPB, ENPEP, COL9A1, NOTCH2, PAPPA2, PTPRJ, CENTD3, MARK2, SLIT3, CTTNBP2, THBS1, PLCE1, NOTCH4, FGD2, COMP, ALS2, TNR, DSCAML1, PCLKC, NOTCH1, CRHR2, EP300, CACNA1F, SLIT1, CDON, ROBO2, RET, STAB2, NF1, ULK1, MYBPC3, MYH10, MYH9, XDH, HHIP, VAV2, COL3A1, TIE1, MYO6, MYO1A, COL4A2, PLXNA3, PTPRZ1, ENG, WHSC1L1, STAT5A, PTK7, MIB1, NOTCH3,
GO:0046578~regulation of Ras protein signal transduction	20	3.68%	2.57E-06	VAV2, KIAA1244, ARHGAP27, PLEKHG5, OBSCN, FGD2, VAV1, ALS2, TIAM1, ITSN2, NF1, NOTCH2, ARFGEF1, CENTD3, ARHGEF10L, SPTB, SOS2, ARFGEF2, FLJ10357, PLCE1,
GO:0006796~phosphate metabolic process	56	10.31%	2.65E-06	PTPRE, RPS6KA2, STK31, TEX14, MAP4K1, PTPRO, TEK, PTPRJ, MARK2, WNK2, CTTNBP2, ROCK1, TRPM7, IL31RA, ULK4, CDC42BPG, ROS1, AKT3, PTK2, BMX, TTN, PTPRN2, MST1R, NIN, MINK1, PTPN20A, RET, PTPRG, CARD14, STK32A, ULK1, PTPN13, PTPRM, PTPRC, NEK1, LRRK2, PTPRN, BUB1B, TIE1, PTPRD, PTPRS, OBSCN, WNK3, PTPN4, PTPRA, RPS6KA1, PTPRZ1, CDKL5, PTPRB, PTK7, ADCK5, PASK, CAMK2G, BUB1, FTS, MYLK, ADRBK2,
GO:0006793~phosphorus metabolic process	56	10.31%	2.65E-06	PTPRE, RPS6KA2, STK31, TEX14, MAP4K1, PTPRO, TEK, PTPRJ, MARK2, WNK2, CTTNBP2, ROCK1, TRPM7, IL31RA, ULK4, CDC42BPG, ROS1, AKT3, PTK2, BMX, TTN, PTPRN2, MST1R, NIN, MINK1, PTPN20A, RET, PTPRG, CARD14, STK32A, ULK1, PTPN13, PTPRM, PTPRC, NEK1, LRRK2, PTPRN, BUB1B, TIE1, PTPRD, PTPRS, OBSCN, WNK3, PTPN4, PTPRA, RPS6KA1, PTPRZ1, CDKL5, PTPRB, PTK7, ADCK5, PASK, CAMK2G, BUB1, FTS, MYLK, ADRBK2,
GO:0007265~Ras protein signal transduction	23	4.24%	4.24E-06	VAV2, KIAA1244, ARHGAP27, PLEKHG5, OBSCN, FGD2, VAV1, ALS2, TIAM1, ITSN2, NF1, ULK1, NOTCH2, ARFGEF1, CENTD3, ARHGEF10L, SPTB, SOS2, ARFGEF2, PLD1, ROCK1, FLJ10357, PLCE1,
GO:0007266~Rho protein signal transduction	15	2.76%	4.99E-06	VAV2, PLEKHG5, ARHGAP27, OBSCN, VAV1, FGD2, TIAM1, ALS2, ITSN2, CENTD3, ARHGEF10L, SPTB, SOS2, ROCK1, FLJ10357,
GO:0015674~di-, tri-valent inorganic cation transport	17	3.13%	6.14E-06	RYR2, RYR3, CACNA1S, ATP2A1, RYR1, CACNA1E, CACNA1B, CACNA2D1, CACNA1C, CACNB2, CAMK2G, CACNA1A, TRPM4, CACHD1, TRPM7, CACNA1F, CACNA1D

Term	Count	%	P-Value	Genes
GO:0048699~generation of neurons	24	4.42%	6.29E-06	HHIP, OPHN1, MYO6, CDK5RAP2, CHL1, BAI1, RET, ROBO2, ALS2, TIAM1, TNR, PLXNA3, PTPRZ1, DSCAML1, NF1, ULK1, CNTN2, SLIT3, ACSL6, CTTNBP2, MIB1, NOTCH3, CACNA1F, SLIT1,
GO:0007517~muscle development	18	3.31%	9.13E-06	MYH3, MYH11, MYH7, NEB, CDON, COL4A4, ALS2, TTN, MYH6, NF1, NEO1, MYBPC3, SGCA, DMD, CACNB2, NOTCH1, EP300, UTRN,
GO:0051641~cellular localization	49	9.02%	1.28E-05	UACA, XPO1, MYH7, MYH6, MYO7A, PKD1L2, MYO5A, DNAH7, NCBP1, SYTL2, EXOC2, DNAH1, LYST, ALS2, ANKRD27, KTN1, UNC13D, CACNA1B, KIF13B, IPO11, NIN, ARFGEF2, DNAH10, CLTC, DNAH5, DNAH8, NF1, DNAH3, NUP133, VPS13A, ARFGEF1, CENPE, OPA1, MYH4, MYH9, MYH3, KIF1A, UNC13B, MYO6, MYO1A, MYO1E, RYR1, PACS1, NFKBIL2, DST, CCDC40, RANBP17, C20orf23, DNHD2,
GO:0006464~protein modification process	81	14.92%	1.36E-05	PAM, RPS6KA2, PTPRE, UGCGL2, STK31, TEX14, MAP4K1, HACE1, PTPRO, TEK, PTPRJ, WNK2, MARK2, HDAC10, CTTNBP2, NFX1, TRPM7, ROCK1, UGCGL1, IL31RA, ULK4, CDC42BPG, ROS1, AKT3, PTK2, UST, USP37, TTN, BMX, PTPRN2, HERC6, MST1R, RNF20, NIN, MYT1, EP300, NEDD4, MINK1, PTPN20A, RET, PTPRG, ULK1, CARD14, STK32A, PTPN13, BRCA1, PTPRM, HECW2, PTPRC, NEK1, LRRK2, BIRC6, PTPRN, PRKDC, BUB1B, TIE1, PTPRD, PTPRS, OBSCN, USP47, PTPN4, WNK3, PTPRA, RYR1, USP28, PTPRZ1, RPS6KA1, WHSC1L1, CDKL5, PTPRB, ADCK5, PTK7, NID1, PASK, CAMK2G, SENP7, BUB1, C17orf27, MIB1, FTS, ADRBK2, MYLK,
GO:0007018~microtubule-based movement	14	2.58%	1.38E-05	KIF1A, DNAH1, DNAH5, DNAH8, KTN1, DST, DNAH3, KIF13B, CENPE, OPA1, C20orf23, DNHD2, DNAH10, DNAH7,
GO:0006468~protein amino acid phosphorylation	41	7.55%	1.46E-05	MINK1, RPS6KA2, RET, STK31, TEX14, ULK1, CARD14, STK32A, MAP4K1, TEK, WNK2, MARK2, NEK1, CTTNBP2, LRRK2, TRPM7, ROCK1, IL31RA, BUB1B, TIE1, ULK4, CDC42BPG, OBSCN, WNK3, ROS1, AKT3, PTK2, TTN, BMX, RPS6KA1, CDKL5, ADCK5, PTK7, PASK, MST1R, CAMK2G, NIN, BUB1, FTS, ADRBK2, MYLK,
GO:0051056~regulation of small GTPase mediated signal transduction	21	3.87%	1.75E-05	VAV2, KIAA1244, ARHGAP27, CDC42BPG, PLEKHG5, OBSCN, FGD2, VAV1, ALS2, TIAM1, ITSN2, NF1, NOTCH2, ARFGEF1, CENTD3, ARHGEF10L, SPTB, SOS2, ARFGEF2, FLJ10357, PLCE1,
GO:0043412~biopolymer modification	83	15.29%	1.80E-05	PAM, RPS6KA2, PTPRE, UGCGL2, STK31, TEX14, MAP4K1, HACE1, PTPRO, TEK, PTPRJ, WNK2, MARK2, HDAC10, CTTNBP2, NFX1, TRPM7, ROCK1, UGCGL1, IL31RA, ULK4, CDC42BPG, ROS1, AKT3, PTK2, UST, USP37, TTN, PARN, BMX, PTPRN2, HERC6, MST1R, RNF20, NIN, MYT1, EP300, NEDD4, MINK1, PTPN20A, RET, PTPRG, ULK1, STK32A, CARD14, PTPN13, BRCA1, PTPRM, HECW2, PTPRC, NEK1, LRRK2, BIRC6, PTPRN, PRKDC, BUB1B, TIE1, PTPRD, PTPRS, OBSCN, USP47, PTPN4, WNK3, PTPRA, RYR1, USP28, PTPRZ1, RPS6KA1, WHSC1L1, CDKL5, PTPRB, ADCK5, PTK7, NID1, PASK, CAMK2G, SENP7, BUB1, C17orf27, MIB1, FTS, ADRBK2, MYLK, ATRX,
GO:0043062~extracellular structure organization and biogenesis	13	2.39%	1.90E-05	MYH11, MYO6, COL4A2, TNXB, ALS2, COL4A4, TNR, NF1, COL5A2, CACNB2, NFKB2, COL12A1, COL11A2,

Term	Count	%	P-Value	Genes
GO:0032501~multicellular organismal process	143	26.34%	2.12E-05	COL4A3, COL17A1, ALMS1, MYH7, NEB, NOS1, BAI1, SFTPB, NAALAD2, MYO7A, TECTA, MYH1, COL1A2, COL2A1, MARK2, SLIT3, MYO9A, PLCE1, IL31RA, COL9A2, CACNA1S, SOX5, TTN, BMX, CACNA1B, LRP1, CDH23, MYBPC1, SLMAP, NOTCH1, CACNA1F, ATP6V0A4, SLIT1, STAT6, BBS5, MINK1, TCOF1, NFKB1, RET, USH2A, NF1, ULK1, COL7A1, MYBPC3, OSBPL1A, CNTN2, MYH9, XDH, PRKDC, MYH3, MTR, MYH11, OBSCN, COL4A2, PRPF8, ODF2, NELL1, COL4A4, TLL1, CACNA1E, PLXNA3, PTPRZ1, STAT5A, CNGB1, CACNA1C, PTK7, CDH5, NFKB2, WRN, ACSL6, MIB1, RNF31, NOTCH3, L1CAM, OPHN1, CHL1, PPL, TIAM1, MYH6, ODZ1, ENPEP, COL9A1, NOTCH2, MAP4K1, CHD8, NPHS1, PTPRJ, CACNB2, CTTNBP2, THBS1, TTC7A, NOTCH4, CDK5RAP2, COMP, KIAA1217, ALS2, TNR, DSCAML1, VWF, DRB1, COL5A2, DMD, CRHR2, MST1R, COL12A1, MYT1, EP300, COL11A2, SLC15A1, ATP1A2, MUC6, ATP2A1, CDON, MYH2, ROBO2, STAB2, SGCA, OPA1, MYH4, MYH8, PTPRC, UTRN, HHIP, COL3A1, TIE1, RYR2, MYO6, UNC13B, MUC2, PIWIL2, MYO1A, TMC1, NPHP1, RYR1, ENG, NEO1, TRPM8, AFF3, PPP1R12A, COL1A1, NOS3, CACNA1D, ATRX,
GO:0022008~neurogenesis	24	4.42%	2.18E-05	HHIP, OPHN1, MYO6, CDK5RAP2, CHL1, BAI1, RET, ROBO2, ALS2, TIAM1, TNR, PLXNA3, PTPRZ1, DSCAML1, NF1, ULK1, CNTN2, SLIT3, ACSL6, CTTNBP2, MIB1, NOTCH3, CACNA1F, SLIT1,
GO:0006470~protein amino acid dephosphorylation	15	2.76%	2.37E-05	PTPRD, PTPRS, PTPN4, PTPRE, PTPRA, PTPN20A, PTPRG, PTPRZ1, PTPRN2, PTPN13, PTPRO, PTPRB, PTPRM, PTPRJ, PTPRC, PTPRN,
GO:0000902~cell morphogenesis	32	5.89%	2.62E-05	SPTA1, OPHN1, DCBLD2, PCNT, CHL1, BAI1, ROBO2, TIAM1, MYO7A, ULK1, NOTCH2, PAPPA2, MYH10, MARK2, CENTD3, MYH9, SLIT3, PLCE1, VAV2, MYO6, MYO1A, FGD2, ALS2, TNR, PLXNA3, DSCAML1, PTPRZ1, WHSC1L1, PCLKC, PTK7, CACNA1F,
GO:0032989~cellular structure morphogenesis	32	5.89%	2.62E-05	SPTA1, OPHN1, DCBLD2, PCNT, CHL1, BAI1, ROBO2, TIAM1, MYO7A, ULK1, NOTCH2, PAPPA2, MYH10, MARK2, CENTD3, MYH9, SLIT3, PLCE1, VAV2, MYO6, MYO1A, FGD2, ALS2, TNR, PLXNA3, DSCAML1, PTPRZ1, WHSC1L1, PCLKC, PTK7, CACNA1F,
GO:0051649~establishment of cellular localization	47	8.66%	2.81E-05	UACA, XPO1, DNAH5, MYH7, DNAH8, MYH6, PKD1L2, MYO7A, NF1, NUP133, DNAH3, VPS13A, MYO5A, ARFGEF1, CENPE, OPA1, MYH4, MYH9, DNAH7, NCBP1, SYTL2, MYH3, KIF1A, EXOC2, DNAH1, MYO6, UNC13B, MYO1E, RYR1, PACS1, LYST, ANKRD27, ALS2, NFKBIL2, UNC13D, DST, KTN1, CACNA1B, CCDC40, KIF13B, RANBP17, C20orf23, IPO11, ARFGEF2, DNHD2, CLTC, DNAH10,
GO:0030154~cell differentiation	80	14.73%	4.44E-05	UACA, COL4A3, OPHN1, NEB, CHL1, PPL, BAI1, TIAM1, KIAA1967, MYO7A, NOTCH2, PAPPA2, MAP4K1, PTPRJ, MARK2, CACNB2, SLIT3, CTTNBP2, ROCK1, IL31RA, NOTCH4, CDK5RAP2, ALS2, SOX5, TTN, TNR, DSCAML1, DRB1, MYBPC1, NOTCH1, MYT1, EP300, CACNA1F, ASAH2, SLIT1, CDON, NFKB1, ROBO2, RET, USH2A, NF1, ULK1, CARD14, BRCA1, OPA1, CNTN2, PTPRC, CACNA1A, MYH9, GNPTAB, SYNE1, BIRC6, XDH, PRKDC, BUB1B, HHIP, PAPPA, MYH11, MYO6, UNC13B, MUC2, PIWIL2, OBSCN, RYR1, ODF2, COL4A4, TLL1, PLXNA3, PTPRZ1, NEO1, WHSC1L1, STAT5A, CCAR1, PTK7, WRN, RNF17, ACSL6, MIB1, NOTCH3, L1CAM,

Term	Count	%	P-Value	Genes
GO:0048869~cellular developmental process	80	14.73%	4.44E-05	UACA, COL4A3, OPHN1, NEB, CHL1, PPL, BAI1, TIAM1, KIAA1967, MYO7A, NOTCH2, PAPPA2, MAP4K1, PTPRJ, MARK2, CACNB2, SLIT3, CTTNBP2, ROCK1, IL31RA, NOTCH4, CDK5RAP2, ALS2, SOX5, TTN, TNR, DSCAML1, DRB1, MYBPC1, NOTCH1, MYT1, EP300, CACNA1F, ASAH2, SLIT1, CDON, NFKB1, ROBO2, RET, USH2A, NF1, ULK1, CARD14, BRCA1, OPA1, CNTN2, PTPRC, CACNA1A, MYH9, GNPTAB, SYNE1, BIRC6, XDH, PRKDC, BUB1B, HHIP, PAPPA, MYH11, MYO6, UNC13B, MUC2, PIWIL2, OBSCN, RYR1, ODF2, COL4A4, TLL1, PLXNA3, PTPRZ1, NEO1, WHSC1L1, STAT5A, CCAR1, PTK7, WRN, RNF17, ACSL6, MIB1, NOTCH3, L1CAM,
GO:0042592~homeostatic process	28	5.16%	4.48E-05	RYR3, NARFL, USH2A, NF1, SFTPB, SLC12A5, ATP1A3, PTPRC, CACNA1A, TTC7A, PLCE1, IL31RA, ATP1A4, RYR2, SLC4A10, RYR1, CACNA1E, TRPM8, SLC12A4, STAT5A, CNGB1, LOC133308, CDH23, CACNA1C, EP300, CACNA1F, ATP1A2,
GO:0046907~intracellular transport	40	7.37%	4.50E-05	UACA, XPO1, DNAH5, MYH7, DNAH8, MYH6, PKD1L2, MYO7A, NF1, NUP133, DNAH3, VPS13A, MYO5A, CENPE, OPA1, MYH4, MYH9, DNAH7, NCBP1, SYTL2, MYH3, KIF1A, DNAH1, MYO6, MYO1E, PACS1, LYST, ANKRD27, ALS2, NFKBIL2, DST, KTN1, CCDC40, KIF13B, RANBP17, C20orf23, IPO11, DNHD2, CLTC, DNAH10,
GO:0016311~dephosphorylation	15	2.76%	8.22E-05	PTPRD, PTPRS, PTPN4, PTPRE, PTPRA, PTPN20A, PTPRG, PTPRZ1, PTPRN2, PTPN13, PTPRO, PTPRB, PTPRM, PTPRJ, PTPRC, PTPRN,
GO:0030036~actin cytoskeleton organization and biogenesis	18	3.31%	8.72E-05	SPTA1, OPHN1, MYH11, CDC42BPG, NEB, FGD2, NPHP1, NEBL, TNXB, TTN, DST, NF1, FMNL1, ARHGEF10L, SPTB, MYH9, TRPM7, ROCK1,
GO:0030030~cell projection organization and biogenesis	19	3.50%	1.04E-04	VAV2, OPHN1, MYO6, PCNT, MYO1A, CHL1, FGD2, BAI1, ROBO2, TIAM1, ALS2, TNR, PLXNA3, PTPRZ1, DSCAML1, ULK1, SLIT3, CACNA1F, SLIT1,
GO:0032990~cell part morphogenesis	19	3.50%	1.04E-04	VAV2, OPHN1, MYO6, PCNT, MYO1A, CHL1, FGD2, BAI1, ROBO2, TIAM1, ALS2, TNR, PLXNA3, PTPRZ1, DSCAML1, ULK1, SLIT3, CACNA1F, SLIT1,
GO:0048858~cell projection morphogenesis	19	3.50%	1.04E-04	VAV2, OPHN1, MYO6, PCNT, MYO1A, CHL1, FGD2, BAI1, ROBO2, TIAM1, ALS2, TNR, PLXNA3, PTPRZ1, DSCAML1, ULK1, SLIT3, CACNA1F, SLIT1,
GO:0000904~cellular morphogenesis during differentiation	15	2.76%	1.34E-04	OPHN1, CHL1, BAI1, ROBO2, TIAM1, ALS2, TNR, PLXNA3, PTPRZ1, DSCAML1, MYO7A, ULK1, SLIT3, CACNA1F, SLIT1,
GO:0030198~extracellular matrix organization and biogenesis	9	1.66%	1.47E-04	MYH11, COL5A2, COL4A2, NFKB2, TNXB, COL12A1, TNR, NF1, COL11A2,
GO:0048468~cell development	57	10.50%	2.23E-04	COL4A3, UACA, OPHN1, CHL1, NEB, BAI1, TIAM1, KIAA1967, MYO7A, NOTCH2, CACNB2, SLIT3, CTTNBP2, ROCK1, IL31RA, CDK5RAP2, ALS2, TNR, TTN, DSCAML1, NOTCH1, EP300, CACNA1F, SLIT1, ASAH2, CDON, NFKB1, RET, ROBO2, NF1, CARD14, ULK1, OPA1, BRCA1, CNTN2, PTPRC, CACNA1A, BIRC6, PRKDC, BUB1B, HHIP, MYH11, MYO6, UNC13B, MUC2, RYR1, COL4A4, PTPRZ1, PLXNA3, NEO1, STAT5A, CCAR1, WRN, RNF17, ACSL6, NOTCH3, MIB1,

Term	Count	%	P-Value	Genes
GO:0030182~neuron differentiation	19	3.50%	2.26E-04	OPHN1, MYO6, CDK5RAP2, CHL1, BAI1, RET, ROBO2, TIAM1, ALS2, TNR, PLXNA3, PTPRZ1, DSCAML1, ULK1, SLIT3, MIB1, CACNA1F, NOTCH3, SLIT1,
GO:0048812~neurite morphogenesis	14	2.58%	2.35E-04	OPHN1, CHL1, BAI1, ROBO2, TIAM1, ALS2, TNR, PTPRZ1, DSCAML1, PLXNA3, ULK1, SLIT3, CACNA1F, SLIT1,
GO:0048667~neuron morphogenesis during differentiation	14	2.58%	2.35E-04	OPHN1, CHL1, BAI1, ROBO2, TIAM1, ALS2, TNR, PTPRZ1, DSCAML1, PLXNA3, ULK1, SLIT3, CACNA1F, SLIT1,
GO:0031175~neurite development	15	2.76%	2.39E-04	OPHN1, MYO6, CHL1, BAI1, ROBO2, TIAM1, ALS2, TNR, PTPRZ1, DSCAML1, PLXNA3, ULK1, SLIT3, CACNA1F, SLIT1,
GO:0001539~ciliary or flagellar	5	0.92%	2.44E-04	DNAH1, DNAH5, DNAH8, DNAH7, DNAH3,
GO:0009887~organ morphogenesis	26	4.79%	2.54E-04	COL4A3, MYH7, MYH6, STAB2, NF1, MYO7A, SFTPB, ENPEP, COL9A1, NOTCH2, MYBPC3, PTPRJ, MYH9, SLIT3, THBS1, NOTCH4, HHIP, COL3A1, TIE1, COL4A2, COMP, DSCAML1, ENG, CRHR2, EP300, MIB1,
GO:0030001~metal ion transport	27	4.97%	2.87E-04	RYR3, ATP2A1, SLC12A1, SLC12A2, SLC12A5, ATP1A3, CACNA2D1, CACNB2, CACNA1A, TRPM7, ATP1A4, RYR2, CACNA1S, SLC4A10, RYR1, CACNA1E, ATP12A, SLC12A3, CACNA1B, SLC12A4, CACNA1C, CAMK2G, TRPM4, CACHD1, CACNA1F, ATP1A2, CACNA1D,
GO:0048666~neuron development	16	2.95%	3.31E-04	OPHN1, MYO6, CHL1, BAI1, RET, ROBO2, TIAM1, ALS2, TNR, PLXNA3, PTPRZ1, DSCAML1, ULK1, SLIT3, CACNA1F, SLIT1,
GO:0007519~skeletal muscle	10	1.84%	3.35E-04	NEO1, MYH11, CDON, CACNB2, DMD, NOTCH1, COL4A4, ALS2, TTN, EP300,
GO:0050793~regulation of developmental process	19	3.50%	3.48E-04	NOTCH4, SPTA1, CDK5RAP2, FGD2, ROBO2, USH2A, TIAM1, PLXNA3, NF1, NOTCH2, STAT5A, MAP4K1, MYH10, CENTD3, NOTCH1, MYH9, MIB1, NOTCH3, XDH,
GO:0048747~muscle fiber development	8	1.47%	4.34E-04	NEO1, MYH11, CDON, CACNB2, NOTCH1, COL4A4, ALS2, TTN,
GO:0048741~skeletal muscle fiber development	8	1.47%	4.34E-04	NEO1, MYH11, CDON, CACNB2, NOTCH1, COL4A4, ALS2, TTN,
GO:0007017~microtubule-based process	17	3.13%	4.45E-04	BUB1B, KIF1A, DNAH1, DNAH5, DNAH8, KTN1, DST, DNAH3, KIF13B, CENPE, BRCA1, OPA1, C20orf23, DNHD2, DNAH10, CTTNBP2, DNAH7,
GO:0007409~axonogenesis	13	2.39%	5.04E-04	OPHN1, CHL1, BAI1, ROBO2, TIAM1, TNR, PTPRZ1, DSCAML1, PLXNA3, ULK1, SLIT3, CACNA1F, SLIT1,
GO:0048878~chemical homeostasis	19	3.50%	7.08E-04	ATP1A4, RYR2, RYR3, NARFL, SLC4A10, RYR1, CACNA1E, SLC12A5, ATP1A3, TRPM8, LOC133308, CACNA1C, PTPRC, CACNA1A, CACNA1F, ATP6V0A4, ATP1A2, PLCE1,
GO:0003008~system process	62	11.42%	8.25E-04	COL4A3, ALMS1, MYH7, NOS1, MYH6, MYO7A, NAALAD2, TECTA, MYH1, COL1A2, NPHS1, COL2A1, CTTNBP2, MYO9A, PLCE1, CACNA1S, ALS2, TTN, CACNA1B, MYBPC1, CDH23, SLMAP, DMD, CACNA1F, COL11A2, ATP6V0A4, ATP1A2, BBS5, MUC6, ATP2A1, MYH2, USH2A, NF1, MYBPC3, OPA1, SGCA, MYH4, MYH8, MYH9, UTRN, MYH3, COL3A1, RYR2, MYH11, MYO6, UNC13B, MUC2, MYO1A, TMC1, PRPF8, NPHP1, RYR1, COL4A4, CACNA1E, ENG, TRPM8, PPP1R12A, CNGB1, CACNA1C,

Term	Count	%	P-Value	Genes
GO:0065008~regulation of biological quality	42	7.73%	8.46E-04	SPTA1, RYR3, DCBLD2, NARFL, NEB, USH2A, SFTPB, NF1, NOTCH2, SLC12A5, ATP1A3, PAPPA2, PTPRC, CACNA1A, SPTB, CTTNBP2, THBS1, PLCE1, IL31RA, TTC7A, ATP1A4, RYR2, SLC4A10, RYR1, NEBL, ALS2, CACNA1E, WHSC1L1, VWF, TRPM8, CACNA1B, PCLKC, SLC12A4, STAT5A, CNGB1, CDH23, LOC133308, CACNA1C, EP300, CACNA1F, ATP6V0A4, ATP1A2,
GO:0007507~heart development	12	2.21%	8.88E-04	OSBPL1A, MYH11, MYH7, PTPRJ, NOTCH1, MYH6, EP300, MIB1, ENG, NF1, PRKDC, PLCE1,
GO:0050801~ion homeostasis	17	3.13%	9.55E-04	ATP1A4, RYR2, RYR3, SLC4A10, RYR1, SLC12A5, ATP1A3, TRPM8, LOC133308, CACNA1C, PTPRC, CACNA1A, CACNA1F, ATP1A2, ATP6V0A4, PLCE1, TTC7A,
GO:0007399~nervous system development	38	7.00%	1.08E-03	OPHN1, CHL1, BAI1, ROBO2, RET, TIAM1, NF1, ODZ1, ULK1, NOTCH2, CNTN2, SLIT3, CTTNBP2, THBS1, PRKDC, HHIP, NOTCH4, MTR, MYO6, CDK5RAP2, NELL1, COL4A4, ALS2, TNR, PLXNA3, DSCAML1, PTPRZ1, DRB1, PTK7, ACSL6, MYT1, EP300, MIB1, CACNA1F, NOTCH3, SLIT1, L1CAM, ATRX,
GO:0031589~cell-substrate adhesion	10	1.84%	1.11E-03	VWF, COL17A1, MUC4, TECTA, SIGLEC1, NID1, TNXB, NF1, THBS3, TRPM7,
GO:0016310~phosphorylation	41	7.55%	1.15E-03	MINK1, RPS6KA2, RET, STK31, TEX14, ULK1, CARD14, STK32A, MAP4K1, TEK, WNK2, MARK2, NEK1, CTTNBP2, LRRK2, TRPM7, ROCK1, IL31RA, BUB1B, TIE1, ULK4, CDC42BPG, OBSCN, WNK3, ROS1, AKT3, PTK2, TTN, BMX, RPS6KA1, CDKL5, ADCK5, PTK7, PASK, MST1R, CAMK2G, NIN, BUB1, FTS, ADRBK2, MYLK,
GO:0016477~cell migration	18	3.31%	1.56E-03	VAV2, TIE1, OPHN1, MUC2, CHL1, RET, ROBO2, TNR, TNS1, PLXNA3, NF1, ENPEP, CNTN2, CENTD3, MYH9, SLIT3, CTTNBP2, SLIT1,
GO:0016525~negative regulation of angiogenesis	5	0.92%	1.64E-03	COL4A3, TIE1, COL4A2, CRHR2, THBS1,
GO:0006812~cation transport	29	5.34%	1.65E-03	RYR3, ATP2A1, SLC12A1, SLC12A2, SLC12A5, ATP1A3, CACNA2D1, CACNB2, CACNA1A, TRPM7, ATP1A4, RYR2, CACNA1S, SLC4A10, RYR1, CACNA1E, ATP12A, SLC12A3, CACNA1B, SLC12A4, ATP8A1, CACNA1C, CAMK2G, TRPM4, CACHD1, CACNA1F, ATP1A2, ATP6V0A4, CACNA1D,
GO:0014706~striated muscle	10	1.84%	1.99E-03	NEO1, MYH11, CDON, CACNB2, DMD, NOTCH1, COL4A4, ALS2, TTN, EP300,
GO:0016192~vesicle-mediated transport	27	4.97%	2.19E-03	ARHGAP27, VAV1, ITSN2, STAB2, MYO7A, PKD1L2, OSBPL1A, VPS13A, ARFGEF1, CLEC7A, CENTD3, SYTL2, EXOC2, SORL1, MYO6, UNC13B, RYR1, LYST, PACS1, ALS2, ANKRD27, UNC13D, LRP1, TSNARE1, ARFGEF2, CLTC, MIB1,
GO:0001568~blood vessel development	14	2.58%	2.34E-03	NOTCH4, COL4A3, TIE1, COL4A2, STAB2, NF1, ENG, ENPEP, PTPRJ, CDH5, CRHR2, MYH9, THBS1, MIB1,
GO:0001944~vasculature development	14	2.58%	2.69E-03	NOTCH4, COL4A3, TIE1, COL4A2, STAB2, NF1, ENG, ENPEP, PTPRJ, CDH5, CRHR2, MYH9, THBS1, MIB1,
GO:0007169~transmembrane receptor protein tyrosine kinase signaling	14	2.58%	3.07E-03	HHIP, PHIP, ROS1, PTK2, PTPRG, TIAM1, ZFP106, COL1A2, TEK, PTK7, PTPRJ, NIN, IL31RA, PLCE1,
GO:0006821~chloride transport	7	1.29%	3.08E-03	SLC12A3, SLC12A5, SLC12A4, SLC4A10, SLC12A1, SLC12A2, CTTNBP2,

Term	Count	%	P-Value	Genes
GO:0007264~small GTPase mediated signal transduction	26	4.79%	3.21E-03	ARHGAP27, VAV1, RALGPS2, TIAM1, ITSN2, NF1, ULK1, NOTCH2, ARFGEF1, CENTD3, SPTB, ARHGEF10L, SOS2, ROCK1, LRRK2, PLCE1, FLJ10357, VAV2, KIAA1244, PLEKHG5, CDC42BPG, OBSCN, FGD2, ALS2, ARFGEF2, PLD1,
GO:0007160~cell-matrix adhesion	9	1.66%	3.31E-03	COL17A1, MUC4, TECTA, SIGLEC1, NID1, TNXB, NF1, THBS3, TRPM7,
GO:0051093~negative regulation of developmental process	9	1.66%	3.55E-03	NOTCH4, STAT5A, NOTCH1, USH2A, MIB1, PLXNA3, NOTCH3, NF1, NOTCH2,
GO:0045596~negative regulation of cell differentiation	8	1.47%	4.43E-03	NOTCH4, STAT5A, NOTCH1, USH2A, MIB1, NOTCH3, NF1, NOTCH2,
GO:0030003~cellular cation homeostasis	13	2.39%	4.76E-03	ATP1A4, RYR3, RYR2, RYR1, ATP1A3, TRPM8, CACNA1C, CACNA1A, PTPRC, CACNA1F, ATP1A2, PLCE1, TTC7A,
GO:0045765~regulation of	6	1.10%	4.79E-03	COL4A3, TIE1, COL4A2, CRHR2, NF1, THBS1,
GO:0048628~myoblast maturation	4	0.74%	4.96E-03	NEO1, MYH11, CDON, TTN,
GO:0030199~collagen fibril	4	0.74%	4.96E-03	COL5A2, COL12A1, NF1, COL11A2,
GO:0055080~cation homeostasis	13	2.39%	4.97E-03	ATP1A4, RYR3, RYR2, RYR1, ATP1A3, TRPM8, CACNA1C, CACNA1A, PTPRC, CACNA1F, ATP1A2, PLCE1, TTC7A,
GO:0048627~myoblast development	4	0.74%	6.18E-03	NEO1, MYH11, CDON, TTN,
GO:0007167~enzyme linked receptor protein signaling pathway	17	3.13%	6.22E-03	HHIP, PHIP, PTPRD, PTPRS, ROS1, PTPRE, PTK2, PTPRG, TIAM1, ENG, ZFP106, COL1A2, TEK, PTK7, PTPRJ, NIN, IL31RA, PLCE1,
GO:0001525~angiogenesis	11	2.03%	6.66E-03	NOTCH4, COL4A3, TIE1, COL4A2, CRHR2, MYH9, STAB2, ENG, NF1, THBS1, ENPEP,
GO:0048514~blood vessel	12	2.21%	6.76E-03	NOTCH4, COL4A3, TIE1, PTPRJ, COL4A2, CRHR2, MYH9, STAB2, ENG, NF1, THBS1, ENPEP
GO:0019725~cellular homeostasis	18	3.31%	6.77E-03	ATP1A4, RYR2, RYR3, RYR1, USH2A, SLC12A5, ATP1A3, TRPM8, SLC12A4, CNGB1, CDH23, CACNA1C, PTPRC, CACNA1A, CACNA1F, ATP1A2, PLCE1, TTC7A,
GO:0055082~cellular chemical homeostasis	14	2.58%	6.98E-03	ATP1A4, RYR2, RYR3, RYR1, SLC12A5, ATP1A3, TRPM8, CACNA1C, CACNA1A, PTPRC, CACNA1F, ATP1A2, PLCE1, TTC7A,
GO:0006873~cellular ion homeostasis	14	2.58%	6.98E-03	ATP1A4, RYR2, RYR3, RYR1, SLC12A5, ATP1A3, TRPM8, CACNA1C, CACNA1A, PTPRC, CACNA1F, ATP1A2, PLCE1, TTC7A,
GO:0007130~synaptonemal complex assembly	3	0.55%	7.29E-03	STAG3, SYCP2, SYCP1,
GO:0007127~meiosis I	5	0.92%	9.11E-03	STAG3, SYCP2, PIWIL2, ATM, SYCP1,
GO:0009966~regulation of signal transduction	27	4.97%	9.99E-03	ARHGAP27, VAV1, TIAM1, ITSN2, NF1, NOTCH2, ARFGEF1, CENTD3, SPTB, ARHGEF10L, PTPRC, SOS2, PLCE1, IL31RA, FLJ10357, HHIP, VAV2, KIAA1244, PLEKHG5, CDC42BPG, OBSCN, FGD2, CC2D1A, ALS2, ENG, LRP1, ARFGEF2,
GO:0007129~synapsis	3	0.55%	1.07E-02	STAG3, SYCP2, SYCP1,

Term	Count	%	P-Value	Genes
GO:0007154~cell communication	137	25.23%	1.13E-02	COL4A3, PHIP, PASD1, DCBLD2, NOS1, RALGPS2, BAI1, TNS1, NAALAD2, TEK, COL1A2, MARK2, DGKK, MYO9A, PLCE1, IL31RA, MCTP2, CDC42BPG, AKT3, PTK2, BMX, MYO10, CACNA1B, LRP1, KIF13B, NOTCH1, NIN, ARFGEF2, CACNA1F, ADAMTS10, STAT6, ARHGAP27, MINK1, VAV1, NFKB1, RET, PTPRG, SRGAP1, DIP13B, NF1, ULK1, CARD14, DDX54, ARFGEF1, ARHGAP17, BRCA1, MYH9, FLJ10357, SNX14, CASKIN1, KIAA1244, OBSCN, WNK3, CC2D1A, COL4A4, CACNA1E, DST, PLXNA3, RPS6KA1, SORCS2, STAT5A, PTK7, NFKB2, PASK, CAMK2G, PDE11A, PLD1, MIB1, RNF31, NOTCH3, PPARGC1B, OPHN1, EMR1, CHL1, PTPRE, RPS6KA2, C9orf39, TG, TIAM1, PKD1L2, INADL, CNTNAP5, ENPEP, NOTCH2, MAP4K1, CLEC7A, PTPRJ, CENTD3, WNK2, ARHGEF10L, SPTB, BAI2, CTTNBP2, ROCK1, NOTCH4, FGD2, ROS1, LYST, ALS2, TNR, PDE2A, MST1R, CRHR2, EP300, ASAH2, ATP1A2, MLL3, CDON, ITSN2, ATP1A3, DUOX2, PTPRC, SOS2, LRRK2, ADAM32, BUB1B, HHIP, VAV2, TIE1, RYR2, PTPRD, PTPRS, UNC13B, PLEKHG5, TAGAP, NPHP1, RYR1, TNXB, BAI3, ENG
GO:0007411~axon guidance	7	1.29%	1.14E-02	OPHN1, CHL1, ROBO2, SLIT3, TNR, PLXNA3, SLIT1,
GO:0001501~skeletal development	14	2.58%	1.15E-02	COL9A2, TCOF1, COMP, KIAA1217, TLL1, SOX5, DSCAML1, NF1, COL1A2, COL2A1, COL12A1, COL1A1, COL11A2, ATP6V0A4,
GO:0007165~signal transduction	126	23.20%	1.22E-02	COL4A3, PASD1, PHIP, DCBLD2, RALGPS2, BAI1, TNS1, COL1A2, TEK, MARK2, DGKK, MYO9A, PLCE1, IL31RA, MCTP2, CDC42BPG, AKT3, PTK2, BMX, MYO10, LRP1, KIF13B, NOTCH1, NIN, ARFGEF2, ADAMTS10, STAT6, MINK1, ARHGAP27, VAV1, NFKB1, RET, PTPRG, SRGAP1, DIP13B, NF1, CARD14, ULK1, DDX54, ARFGEF1, ARHGAP17, BRCA1, MYH9, FLJ10357, SNX14, CASKIN1, KIAA1244, OBSCN, WNK3, CC2D1A, DST, PLXNA3, RPS6KA1, SORCS2, STAT5A, PTK7, NFKB2, PASK, CAMK2G, PDE11A, PLD1, MIB1, RNF31, NOTCH3, PPARGC1B, OPHN1, EMR1, CHL1, PTPRE, RPS6KA2, C9orf39, TG, TIAM1, PKD1L2, INADL, CNTNAP5, NOTCH2, MAP4K1, CLEC7A, PTPRJ, CENTD3, WNK2, SPTB, ARHGEF10L, BAI2, ROCK1, NOTCH4, FGD2, ROS1, LYST, ALS2, TNR, PDE2A, MST1R, CRHR2, EP300, ASAH2, MLL3, CDON, ITSN2, ATP1A3, DUOX2, PTPRC, SOS2, LRRK2, ADAM32, BUB1B, HHIP, VAV2, TIE1, RYR2, PTPRD, PTPRS, UNC13B, PLEKHG5, TAGAP, NPHP1, RYR1, TNXB, BAI3, ENG
GO:0045445~myoblast differentiation	5	0.92%	1.27E-02	NEO1, MYH11, CDON, NOTCH1, TTN,
GO:0007242~intracellular signaling cascade	57	10.50%	1.30E-02	COL4A3, PPARGC1B, DCBLD2, RPS6KA2, RALGPS2, TIAM1, TNS1, INADL, NOTCH2, MAP4K1, CLEC7A, MARK2, CENTD3, WNK2, ARHGEF10L, SPTB, DGKK, ROCK1, MYO9A, IL31RA, PLCE1, MCTP2, CDC42BPG, FGD2, ALS2, BMX, CRHR2, ARFGEF2, STAT6, MLL3, MINK1, ARHGAP27, VAV1, RET, ITSN2, NF1, CARD14, ULK1, ARFGEF1, DDX54, BRCA1, SOS2, LRRK2, FLJ10357, SNX14, BUB1B, VAV2, KIAA1244, UNC13B, PLEKHG5, OBSCN, WNK3, RYR1, CC2D1A, STAT5A, PLD1, CACNA1D,
GO:0042692~muscle cell differentiation	6	1.10%	1.33E-02	NEO1, MYH11, CDON, NOTCH1, TTN, SYNE1,

Term	Count	%	P-Value	Genes
GO:0045595~regulation of cell differentiation	11	2.03%	1.35E-02	NOTCH4, STAT5A, MAP4K1, CDK5RAP2, NOTCH1, USH2A, MIB1, NOTCH3, NF1, XDH, NOTCH2,
GO:0007420~brain development	11	2.03%	1.40E-02	NOTCH4, CDK5RAP2, ROBO2, CTTNBP2, PLXNA3, NOTCH3, NF1, DSCAML1, SLIT1, ATRX, PRKDC,
GO:0032320~positive regulation of Ras GTPase activity	3	0.55%	1.48E-02	ARHGAP27, ALS2, NF1,
GO:0007229~integrin-mediated signaling pathway	7	1.29%	1.49E-02	VAV1, PTK2, MYH9, DST, ADAM32, COL16A1, ADAMTS10,
GO:0051493~regulation of cytoskeleton organization and biogenesis	6	1.10%	1.56E-02	SPTA1, NEB, NEBL, SPTB, ARHGEF10L, CEP250,
GO:0033043~regulation of organelle organization and biogenesis	6	1.10%	1.56E-02	SPTA1, NEB, NEBL, SPTB, ARHGEF10L, CEP250,
GO:0051239~regulation of multicellular organismal process	16	2.95%	1.68E-02	STAT6, RYR2, MYH7, ATP2A1, COL4A4, MYH6, NF1, CACNA1B, MYBPC3, PPP1R12A, STAT5A, CACNA1C, PTPRC, ATP1A2, IL31RA, PLCE1,
GO:0006937~regulation of muscle contraction	5	0.92%	1.89E-02	MYBPC3, PPP1R12A, ATP2A1, ATP1A2, PLCE1,
GO:0055010~ventricular cardiac muscle morphogenesis	3	0.55%	1.93E-02	MYBPC3, MYH7, MYH6,
GO:0006942~regulation of striated muscle contraction	3	0.55%	1.93E-02	MYBPC3, ATP2A1, ATP1A2,
GO:0030317~sperm motility	4	0.74%	1.96E-02	ATP1A3, ATP1A4, CTTNBP2, ATP1A2,
GO:0055074~calcium ion homeostasis	9	1.66%	1.97E-02	TRPM8, RYR2, RYR3, CACNA1C, RYR1, PTPRC, CACNA1A, CACNA1F, PLCE1,
GO:0006874~cellular calcium ion homeostasis	9	1.66%	1.97E-02	TRPM8, RYR2, RYR3, CACNA1C, RYR1, PTPRC, CACNA1A, CACNA1F, PLCE1,
GO:0030336~negative regulation of cell migration	4	0.74%	2.22E-02	TIE1, MUC2, CENTD3, NF1,
GO:0006814~sodium ion transport	9	1.66%	2.24E-02	ATP1A3, SLC12A3, SLC12A5, ATP1A4, SLC12A4, SLC4A10, SLC12A1, SLC12A2, ATP1A2
GO:0048644~muscle morphogenesis	3	0.55%	2.44E-02	MYBPC3, MYH7, MYH6,
GO:0055008~cardiac muscle	3	0.55%	2.44E-02	MYBPC3, MYH7, MYH6,
GO:0016568~chromatin modification	12	2.21%	2.52E-02	WHSC1L1, MLL3, CHD8, EP400, SMARCAD1, PB1, HDAC10, INOC1, RNF20, ARID1B, EP300, SMARCC2,
GO:0006310~DNA recombination	10	1.84%	2.53E-02	STAT6, TEP1, SMARCAD1, BRCA1, DCLRE1C, WRN, ATM, SYCP1, ATRX, PRKDC,

Term	Count	%	P-Value	Genes
GO:0045935~positive regulation of nucleobase, nucleoside, nucleotide and nucleic acid metabolic process	17	3.13%	2.61E-02	NOTCH4, STAT6, PPARGC1B, MYO6, SMARCAD1, RYR1, NFKB1, SMARCC2, STAT5A, BRCA1, NOTCH1, ARHGEF10L, EP300, ARID1B, NCBP1, TCF4, IL31RA,
GO:0051276~chromosome organization and biogenesis	19	3.50%	2.74E-02	MLL3, STAG3, SMARCAD1, EP400, PAM, PB1, INOC1, SMARCC2, WHSC1L1, SYCP2, TEP1, CHD8, CENPE, WRN, HDAC10, RNF20, EP300, ARID1B, SYCP1,
GO:0048646~anatomical structure	11	2.03%	2.82E-02	NOTCH4, COL4A3, TIE1, COL4A2, CRHR2, MYH9, STAB2, ENG, NF1, THBS1, ENPEP,
GO:0055065~metal ion homeostasis	9	1.66%	2.88E-02	TRPM8, RYR2, RYR3, CACNA1C, RYR1, PTPRC, CACNA1A, CACNA1F, PLCE1,
GO:0006875~cellular metal ion	9	1.66%	2.88E-02	TRPM8, RYR2, RYR3, CACNA1C, RYR1, PTPRC, CACNA1A, CACNA1F, PLCE1,
GO:0007632~visual behavior	3	0.55%	2.99E-02	ATP1A3, NPHP1, NF1,
GO:0000279~M phase	15	2.76%	3.02E-02	BUB1B, STAG3, PIWIL2, STAG2, PAM, PB1, TTN, SYCP2, CENPE, NEK1, ATM, BUB1, STAG1, RSN, SYCP1,
GO:0007417~central nervous system development	14	2.58%	3.02E-02	NOTCH4, MTR, CDK5RAP2, ROBO2, PTPRZ1, DSCAML1, NF1, PLXNA3, SLIT3, CTTNBP2, NOTCH3, SLIT1, PRKDC, ATRX,
GO:0007059~chromosome segregation	6	1.10%	3.05E-02	STAG3, CENPE, BRCA1, STAG2, PAM, STAG1,
GO:0051271~negative regulation of cell motility	4	0.74%	3.45E-02	TIE1, MUC2, CENTD3, NF1,
GO:0030005~cellular di-, tri-valent inorganic cation homeostasis	10	1.84%	3.49E-02	TRPM8, RYR2, RYR3, CACNA1C, RYR1, PTPRC, CACNA1A, CACNA1F, TTC7A, PLCE1,
GO:0032535~regulation of cellular component size	5	0.92%	3.58E-02	SPTA1, NEB, NEBL, SPTB, ALS2,
GO:0030641~cellular hydrogen ion homeostasis	3	0.55%	3.59E-02	ATP1A3, ATP1A4, ATP1A2,
GO:0055066~di-, tri-valent inorganic cation homeostasis	10	1.84%	3.61E-02	TRPM8, RYR2, RYR3, CACNA1C, RYR1, PTPRC, CACNA1A, CACNA1F, TTC7A, PLCE1,
GO:0007126~meiosis	6	1.10%	3.63E-02	STAG3, SYCP2, PIWIL2, STAG2, ATM, SYCP1,
GO:0051327~M phase of meiotic cell	6	1.10%	3.63E-02	STAG3, SYCP2, PIWIL2, STAG2, ATM, SYCP1,
GO:0016044~membrane organization and biogenesis	15	2.76%	3.66E-02	TIE1, SORL1, MYO6, ARHGAP27, CDON, VAV1, RYR1, STAB2, ITSN2, MYO7A, NEO1, LRP1, OPA1, CLEC7A, MIB1,
GO:0045165~cell fate commitment	7	1.29%	3.75E-02	NOTCH4, CDON, NOTCH1, SOX5, NOTCH3, DSCAML1, NOTCH2,
GO:0051128~regulation of cellular component organization and biogenesis	7	1.29%	3.75E-02	SPTA1, NEB, NEBL, SPTB, ARHGEF10L, CEP250, CTTNBP2,

Term	Count	%	P-Value	Genes
GO:0040013~negative regulation of locomotion	4	0.74%	3.81E-02	TIE1, MUC2, CENTD3, NF1,
GO:0022604~regulation of cell morphogenesis	5	0.92%	3.84E-02	SPTA1, MYH10, FGD2, CENTD3, MYH9,
GO:0022603~regulation of anatomical structure morphogenesis	5	0.92%	3.84E-02	SPTA1, MYH10, FGD2, CENTD3, MYH9,
GO:0008360~regulation of cell shape	5	0.92%	3.84E-02	SPTA1, MYH10, FGD2, CENTD3, MYH9,
GO:0008016~regulation of heart	5	0.92%	3.84E-02	CACNA1B, RYR2, MYH7, CACNA1C, MYH6,
GO:0044260~cellular macromolecule metabolic process	115	21.18%	3.92E-02	XPO1, PAM, UGCGL2, NAALADL1, TNS1, NAALAD2, PAPPA2, PTPRO, TEK, MARK2, TRPM7, IL31RA, UGCGL1, DNAH1, CDC42BPG, PYGL, AKT3, XPNPEP1, PTK2, USP37, BMX, TTN, PTPRN2, HERC6, SLMAP, IPO11, RNF20, NIN, ADAMTS10, MINK1, NFKB1, RET, PTPN20A, PTPRG, CARD14, STK32A, ULK1, DPP8, C11orf11, PTPN13, CENPE, BRCA1, HECW2, MYH9, PRKDC, DPP10, PAPPA, OBSCN, WNK3, PTPN4, PTPRA, TLL1, RPS6KA1, PTPRZ1, STAT5A, ADCK5, PTK7, RANBP17, PASK, CAMK2G, BUB1, MIB1, RNF31, SPTA1, PTPRE, RPS6KA2, STK31, TEX14, ENPEP, MAP4K1, HACE1, PTPRJ, WNK2, HDAC10, SPTB, CTTNBP2, NFX1, XPNPEP2, ROCK1, ULK4, ROS1, UST, ALS2, MMEL1, MST1R, MYT1, EP300, PYGB, NEDD4, FLJ32310, DNAH5, ADAMTS12, PTPRM, PTPRC, NEK1, LRRK2, ADAM32, BIRC6, PRSS7, PTPRN, BUB1B, TIE1, PTPRD, PTPRS, USP47, RYR1, USP28, WHSC1L1, CDKL5, PTPRB, NID1, SENP7, C17orf27, FTS, MYLK
GO:0045941~positive regulation of transcription	16	2.95%	3.95E-02	NOTCH4, STAT6, PPARGC1B, MYO6, SMARCAD1, RYR1, NFKB1, SMARCC2, STAT5A, BRCA1, NOTCH1, ARHGEF10L, EP300, ARID1B, TCF4, IL31RA,
GO:0051321~meiotic cell cycle	6	1.10%	4.06E-02	STAG3, SYCP2, PIWIL2, STAG2, ATM, SYCP1,
GO:0022403~cell cycle phase	17	3.13%	4.11E-02	BUB1B, STAG3, PIWIL2, STAG2, PAM, PB1, TTN, SYCP2, CENPE, CAMK2G, PTPRC, NEK1, ATM, BUB1, STAG1, RSN, SYCP1,
GO:0032319~regulation of Rho GTPase activity	3	0.55%	4.23E-02	ARHGAP27, FGD2, ALS2,
GO:0031325~positive regulation of cellular metabolic process	19	3.50%	4.36E-02	NOTCH4, STAT6, PPARGC1B, MYO6, SMARCAD1, RYR1, NFKB1, CARD14, SMARCC2, STAT5A, BRCA1, NOTCH1, ARHGEF10L, EP300, ARID1B, FTS, NCBP1, TCF4, IL31RA,
GO:0001822~kidney development	5	0.92%	4.39E-02	OSBPL1A, ROBO2, RET, NF1, PLCE1,

Term	Count	%	P-Value	Genes
GO:0044267~cellular protein metabolic process	113	20.81%	4.41E-02	XPO1, PAM, UGCGL2, NAALADL1, TNS1, NAALAD2, PAPPA2, PTPRO, TEK, MARK2, TRPM7, IL31RA, UGCGL1, DNAH1, CDC42BPG, AKT3, XPNPEP1, PTK2, USP37, BMX, TTN, PTPRN2, HERC6, SLMAP, IPO11, RNF20, NIN, ADAMTS10, MINK1, NFKB1, RET, PTPN20A, PTPRG, CARD14, STK32A, ULK1, DPP8, C11orf11, PTPN13, CENPE, BRCA1, HECW2, MYH9, PRKDC, DPP10, PAPPA, OBSCN, WNK3, PTPN4, PTPRA, TLL1, RPS6KA1, PTPRZ1, STAT5A, ADCK5, PTK7, RANBP17, PASK, CAMK2G, BUB1, MIB1, RNF31, SPTA1, PTPRE, RPS6KA2, STK31, TEX14, ENPEP, MAP4K1, HACE1, PTPRJ, WNK2, HDAC10, SPTB, CTTNBP2, NFX1, XPNPEP2, ROCK1, ULK4, ROS1, UST, ALS2, MMEL1, MST1R, MYT1, EP300, NEDD4, FLJ32310, DNAH5, ADAMTS12, PTPRM, PTPRC, NEK1, LRRK2, ADAM32, BIRC6, PRSS7, PTPRN, BUB1B, TIE1, PTPRD, PTPRS, USP47, RYR1, USP28, WHSC1L1, CDKL5, PTPRB, NID1, SENP7, C17orf27, ETS, MYLK
GO:0019538~protein metabolic process	119	21.92%	4.51E-02	XPO1, PAM, UGCGL2, NAALADL1, TNS1, NAALAD2, PAPPA2, PTPRO, TEK, MARK2, TRPM7, IL31RA, UGCGL1, DNAH1, CDC42BPG, AKT3, XPNPEP1, PTK2, USP37, BMX, TTN, PTPRN2, HERC6, SLMAP, IPO11, RNF20, NIN, ADAMTS10, MINK1, SMARCAD1, NFKB1, RET, PTPN20A, PTPRG, ULK1, CARD14, STK32A, DPP8, C11orf11, PTPN13, CENPE, BRCA1, HECW2, MYH9, DPP10, PRKDC, PAPPA, MYH11, OBSCN, WNK3, PTPN4, PTPRA, TLL1, RPS6KA1, PTPRZ1, STAT5A, ADCK5, PTK7, RANBP17, PASK, CAMK2G, BUB1, MIB1, RNF31, SPTA1, PTPRE, RPS6KA2, STK31, TEX14, PKD1L2, ENPEP, MAP4K1, HACE1, PTPRJ, WNK2, HDAC10, SPTB, CTTNBP2, NFX1, XPNPEP2, ROCK1, ULK4, ROS1, UST, ALS2, MMEL1, VWF, MST1R, MYT1, EP300, CLTC, NEDD4, FLJ32310, DNAH5, ADAMTS12, PTPRM, PTPRC, NEK1, LRRK2, ADAM32, BIRC6, PRSS7, PTPRN, BUB1B, TIE1, PTPRD, PTPRS, USP47, RYR1, TNXB, USP28, WHSC1L1, CDKL5, PTPRB, NID1, SENP7, C17orf27, ETS, MYLK, ADRBK2
GO:0009628~response to abiotic	10	1.84%	4.67E-02	UACA, TRPM8, ATP1A3, CNGB1, DNAH1, NPHP1, COL1A1, ATM, NF1, CACNA1F,
GO:0010324~membrane invagination	11	2.03%	4.89E-02	SORL1, LRP1, MYO6, ARHGAP27, CLEC7A, VAV1, RYR1, ITSN2, STAB2, MIB1, MYO7A
GO:0006897~endocytosis	11	2.03%	4.89E-02	SORL1, LRP1, MYO6, ARHGAP27, CLEC7A, VAV1, RYR1, ITSN2, STAB2, MIB1, MYO7A
GO:0042308~negative regulation of protein import into nucleus	3	0.55%	4.91E-02	UACA, NFKBIL2, NF1,
GO:0042992~negative regulation of transcription factor import into nucleus	3	0.55%	4.91E-02	UACA, NFKBIL2, NF1,
GO:0006979~response to oxidative	7	1.29%	5.11E-02	PXDN, DUOX2, MYH7, COL1A1, ALS2, DGKK, TTN,
GO:0021700~developmental maturation	6	1.10%	5.23E-02	NEO1, MYH11, CDON, CDH5, RET, TTN,
GO:0007219~Notch signaling pathway	5	0.92%	5.29E-02	NOTCH4, NOTCH1, MIB1, NOTCH3, NOTCH2,
GO:0060047~heart contraction	5	0.92%	5.29E-02	CACNA1B, RYR2, MYH7, CACNA1C, MYH6,
GO:0003015~heart process	5	0.92%	5.29E-02	CACNA1B, RYR2, MYH7, CACNA1C, MYH6,

Term	Count	%	P-Value	Genes
GO:0046823~negative regulation of nucleocytoplasmic transport	3	0.55%	5.63E-02	UACA, NFKBIL2, NF1,
GO:0008088~axon cargo transport	3	0.55%	5.63E-02	KIF1A, OPA1, DST,
GO:0007528~neuromuscular junction development	3	0.55%	5.63E-02	CACNB2, COL4A4, ALS2,
GO:0001656~metanephros	4	0.74%	5.84E-02	OSBPL1A, ROBO2, RET, NF1,
GO:0046777~protein amino acid autophosphorylation	5	0.92%	5.94E-02	CDKL5, CAMK2G, TTN, CTTNBP2, TRPM7,
GO:0009314~response to radiation	7	1.29%	6.01E-02	UACA, ATP1A3, CNGB1, NPHP1, ATM, NF1, CACNA1F,
GO:0001655~urogenital system	5	0.92%	6.28E-02	OSBPL1A, ROBO2, RET, NF1, PLCE1,
GO:0031032~actomyosin structure organization and biogenesis	3	0.55%	6.38E-02	MYH11, TTN, TRPM7,
GO:0051224~negative regulation of protein transport	3	0.55%	6.38E-02	UACA, NFKBIL2, NF1,
GO:0045893~positive regulation of transcription, DNA-dependent	13	2.39%	6.43E-02	STAT6, NOTCH4, PPARGC1B, MYO6, SMARCC2, STAT5A, BRCA1, NOTCH1, ARHGEF10L, EP300, ARID1B, TCF4, IL31RA,
GO:0016540~protein autoprocessing	5	0.92%	6.63E-02	CDKL5, CAMK2G, TTN, CTTNBP2, TRPM7,
GO:0009416~response to light stimulus	6	1.10%	6.88E-02	UACA, ATP1A3, CNGB1, NPHP1, NF1, CACNA1F,
GO:0009893~positive regulation of metabolic process	19	3.50%	7.10E-02	NOTCH4, STAT6, PPARGC1B, MYO6, SMARCAD1, RYR1, NFKB1, CARD14, SMARCC2, STAT5A, BRCA1, NOTCH1, ARHGEF10L, EP300, ARID1B, FTS, NCBP1, TCF4, IL31RA,
GO:0030004~cellular monovalent inorganic cation homeostasis	3	0.55%	7.16E-02	ATP1A3, ATP1A4, ATP1A2,
GO:0045026~plasma membrane fusion	3	0.55%	7.16E-02	NEO1, TIE1, CDON,
GO:0055067~monovalent inorganic cation homeostasis	3	0.55%	7.16E-02	ATP1A3, ATP1A4, ATP1A2,
GO:0051640~organelle localization	4	0.74%	7.76E-02	CENPE, MYO1A, NIN, MYO7A,
GO:0045494~photoreceptor cell maintenance	3	0.55%	7.97E-02	CNGB1, CDH23, USH2A,
GO:0048251~elastic fiber assembly	2	0.37%	8.11E-02	MYH11, TNXB,
GO:0002520~immune system development	11	2.03%	8.38E-02	NOTCH4, STAT6, STAT5A, MAP4K1, NFKB2, RYR1, PTPRC, MYH9, TTC7A, NOTCH2, IL31RA,

Term	Count	%	P-Value	Genes
GO:0043623~cellular protein complex assembly	5	0.92%	8.53E-02	XPO1, CENPE, RANBP17, PTK2, IPO11,
GO:0009987~cellular process	372	68.51%	8.70E-02	COL4A3, PASD1, XPO1, NEB, UGCGL2, BAI1, TNS1, PAPPA2, COL1A2, DCLRE1C, MARK2, SLIT3, TRPM7, MYO9A, IL31RA, PLCE1, EXOC2, RIF1, XPNPEP1, NEBL, MYO10, KTN1, PARN, BMX, PTPRN2, FLJ20433, CACNA1B, HERC6, KIF13B, OGDHL, MDH1B, IPO11, ARFGEF2, NIN, RNF20, CACNA1F, STAT6, SMARCAD1, VAV1, USH2A, PTPRG, PTPN20A, RET, DIP13B, STK32A, CARD14, DNAH3, DPP8, OSBPL1A, MYH10, FMNL1, VPS13A, CENPE, ARHGAP17, TARBP1, MYH9, SYNE1, RSN, PRKDC, CASKIN1, MYH3, MYH11, MTR, OBSCN, MYO1E, PTPN4, WNK3, PTPRA, CC2D1A, CACNA1E, NFKBIL2, TLL1, DST, KHDRBS2, PTPRZ1, ACOT12, CACNA1C, PTK7, WRN, POLQ, GON4L, NOTCH3, STAG1, RNF31, TCF4, SDK2, SPTA1, PPARGC1B, CTPS2, EMR1, PPL, RPS6KA2, PTPRE, TIAM1, TEX14, CNTNAP5, CTCFL, INADL, COL9A1, SLC12A5, HACE1, CHD8, NPHS1, ALDH8A1, PTPRJ, CLEC7A, WNK2, DNAH7, XPNPEP2, THBS1, CLCA1, STAG3, COL20A1, UROC1, CDK5RAP2, EP400, COMP, LYST, CASZ1, ALS2, UST, TNR, DSCAML1, MMEL1, KIAA1604, VWF, PCLKC, COL5A2, DMD, CRHR2, MST1R, COL12A1, EP300, CLTC, FANCM, COL11A2, ATP1A2, ASAH2, SYCP1, MLL3
GO:0000059~protein import into nucleus, docking	3	0.55%	8.80E-02	XPO1, RANBP17, IPO11,
GO:0006084~acetyl-CoA metabolic	4	0.74%	8.81E-02	ACSS1, ACOT12, ACO1, MDH1B,
GO:0048469~cell maturation	5	0.92%	9.36E-02	NEO1, MYH11, CDON, RET, TTN,
GO:0000075~cell cycle checkpoint	5	0.92%	9.36E-02	BUB1B, BRCA1, ATM, BUB1, BRIP1,
GO:0006813~potassium ion transport	9	1.66%	9.47E-02	ATP1A3, SLC12A5, ATP1A4, SLC12A4, SLC12A1, SLC12A2, ATP12A, ATP1A2, CACNA1D
GO:0008015~blood circulation	9	1.66%	9.47E-02	CACNA1B, COL4A3, COL3A1, RYR2, MYH7, CACNA1C, CTTNBP2, MYH6, ENG,
GO:0003013~circulatory system	9	1.66%	9.47E-02	CACNA1B, COL4A3, COL3A1, RYR2, MYH7, CACNA1C, CTTNBP2, MYH6, ENG,
GO:0006606~protein import into	6	1.10%	9.48E-02	UACA, XPO1, RANBP17, IPO11, NFKBIL2, NF1,
**Cellular Component Category**				
GO:0016459~myosin complex	26	4.79%	2.94E-21	MYO7B, MYH7, MYH2, MYO1D, MYH6, MYO7A, MYBPC3, MYH10, MYH1, MYO5A, MYO1F, MYO5B, MYH4, MYH8, MYH9, MYH7B, MYO9A, MYH3, MYH11, MYO6, MYO1A, MYO1E, CGN, MYO1C, MYO10, MYBPC1,
GO:0005581~collagen	18	3.31%	4.58E-16	COL9A2, COL4A3, COL3A1, COL4A2, TNXB, COL4A4, COL5A1, COL4A1, COL16A1, COL9A1, COL7A1, COL4A5, COL1A2, COL2A1, COL5A2, COL12A1, COL1A1, COL11A2,
GO:0044420~extracellular matrix part	24	4.42%	9.63E-14	COL9A2, HSPG2, COL4A3, COL3A1, COL4A2, TNXB, USH2A, COL4A4, DST, COL5A1, COL4A1, COL16A1, COL9A1, COL7A1, COL4A5, COL1A2, SGCA, COL5A2, COL2A1, NID1, DMD, COL12A1, COL1A1, COL11A2,

Term	Count	%	P-Value	Genes
GO:0005578~proteinaceous extracellular matrix	40	7.37%	1.39E-13	HSPG2, COL4A3, COL17A1, MUC4, CHL1, ADAMTS12, USH2A, SFTPB, COL9A1, COL7A1, TECTA, COL1A2, SGCA, COL2A1, CLCA1, COL9A2, COL3A1, COL20A1, MUC2, COL4A2, TNXB, COMP, COL4A4, TNR, DST, COL5A1, PTPRZ1, COL4A1, COL16A1, VWF, THBS2, COL4A5, COL5A2, NID1, DMD, COL12A1, COL1A1, THBS3,
GO:0005856~cytoskeleton	79	14.55%	2.01E-13	UACA, SPTA1, ALMS1, OPHN1, MYO7B, PCNT, MYH7, NEB, PPL, MYH6, TNS1, MYO7A, MYO5A, MYH1, MYO1F, MARK2, CENTD3, FRMD4A, MYH7B, SPTB, DNAH7, ROCK1, MYO9A, DNAH1, CDK5RAP2, PLEKHH1, FGD2, PTK2, NEBL, SYNE2, LYST, ALS2, CEP250, MYO10, CDH23, MYBPC1, KIF13B, DMD, MST1R, NIN, DNAH10, DNAH5, DNAH8, MYH2, PTPN20A, MYO1D, DNAH3, MYBPC3, MYH10, PTPN13, CENPE, MYO5B, BRCA1, SGCA, MYH4, MYH8, MYH9, SYNE1, RSN, UTRN, BUB1B, MYH3, KIF1A, MYH11, MYO6, MYO1E, MYO1A, NOS2A, PTPN4, ODF2, CGN, MYO1C, DST, PPP1R12A, CACNA1C, C20orf23, DNHD2, BUB1, NOS3,
GO:0031012~extracellular matrix	40	7.37%	2.42E-13	HSPG2, COL4A3, COL17A1, MUC4, CHL1, ADAMTS12, USH2A, SFTPB, COL9A1, COL7A1, TECTA, COL1A2, SGCA, COL2A1, CLCA1, COL9A2, COL3A1, COL20A1, MUC2, COL4A2, TNXB, COMP, COL4A4, TNR, DST, COL5A1, PTPRZ1, COL4A1, COL16A1, VWF, THBS2, COL4A5, COL5A2, NID1, DMD, COL12A1, COL1A1, THBS3,
GO:0015629~actin cytoskeleton	33	6.08%	4.30E-12	SPTA1, OPHN1, MYO7B, MYH7, NEB, MYH2, MYO1D, MYH6, MYO7A, MYBPC3, MYH10, MYO5A, MYH1, MYO1F, MYO5B, MYH4, MYH8, SPTB, MYH9, MYH7B, MYO9A, MYH3, MYH11, MYO6, MYO1E, MYO1A, NEBL, CGN, MYO1C, MYO10,
GO:0042995~cell projection	36	6.63%	7.85E-11	BBS5, ALMS1, IFT122, DNAH5, CHL1, DNAH8, NOS1, ROBO2, USH2A, NF1, MYO7A, ULK1, ENPEP, DNAH3, MYO5A, OPA1, CNTN2, CENTD3, CACNA1A, MYH9, CTTNBP2, DNAH7, TRPM7, PRSS7, DNAH1, MYO6, MYO1A, FGD2, ODF2, MYO1C, ALS2, CEP250, SHANK1, CDH23, DNAH10, ATP6V0A4,
GO:0005863~striated muscle thick	10	1.84%	1.04E-10	MYBPC3, MYH3, MYH11, MYBPC1, MYH1, MYH7, MYH4, MYH2, MYH8, MYH6,
GO:0032982~myosin filament	10	1.84%	1.04E-10	MYBPC3, MYH3, MYH11, MYBPC1, MYH1, MYH7, MYH4, MYH2, MYH8, MYH6,
GO:0044430~cytoskeletal part	54	9.94%	1.66E-10	SPTA1, ALMS1, MYO7B, PCNT, MYH7, NEB, MYH6, MYO7A, MYH1, MYO5A, MYO1F, SPTB, MYH7B, DNAH7, MYO9A, DNAH1, CDK5RAP2, ALS2, CEP250, MYO10, MYBPC1, KIF13B, MST1R, NIN, DNAH10, DNAH5, DNAH8, MYH2, PTPN20A, MYO1D, DNAH3, MYBPC3, MYH10, CENPE, BRCA1, MYO5B, MYH4, MYH8, MYH9, RSN, BUB1B, MYH3, KIF1A, MYH11, MYO6, MYO1A, MYO1E, ODF2, CGN, MYO1C,

Term	Count	%	P-Value	Genes
GO:0043232~intracellular non-membrane-bound organelle	99	18.23%	8.04E-09	ALMS1, PCNT, MYH7, NEB, PAM, TNS1, MYO7A, MYH1, FRMD4A, MARK2, MYH7B, MYO9A, RIF1, DNAH1, NEBL, PTK2, MYO10, TDRD1, TTN, SMARCC2, MYBPC1, CDH23, KIF13B, NIN, RNF20, DNAH10, TCOF1, PTPN20A, DNAH3, MYBPC3, MYH10, PTPN13, DDX54, CENPE, BRCA1, MYO5B, MYH9, SYNE1, RSN, MYH3, MYH11, MYO1E, NOS2A, PTPN4, ODF2, CGN, DST, CACNA1C, SP100, WRN, BUB1, SPTA1, UACA, OPHN1, MYO7B, PPL, MYH6, SYCP2, CHD8, MYO5A, MYO1F, CENTD3, SPTB, DDX21, ROCK1, DNAH7, STAG3, CDK5RAP2, PLEKHH1, FGD2, LYST, SYNE2, ALS2, CEP250, DMD, MST1R, SYCP1, DNAH5, PB1, MYH2, DNAH8, MYO1D, TEP1, SGCA, UBTF, MYH4, MYH8, UTRN, BUB1B, KIF1A, MYO6, MYO1A, NOL6, MYO1C,
GO:0043228~non-membrane-bound organelle	99	18.23%	8.04E-09	ALMS1, PCNT, MYH7, NEB, PAM, TNS1, MYO7A, MYH1, FRMD4A, MARK2, MYH7B, MYO9A, RIF1, DNAH1, NEBL, PTK2, MYO10, TDRD1, TTN, SMARCC2, MYBPC1, CDH23, KIF13B, NIN, RNF20, DNAH10, TCOF1, PTPN20A, DNAH3, MYBPC3, MYH10, PTPN13, DDX54, CENPE, BRCA1, MYO5B, MYH9, SYNE1, RSN, MYH3, MYH11, MYO1E, NOS2A, PTPN4, ODF2, CGN, DST, CACNA1C, SP100, WRN, BUB1, SPTA1, UACA, OPHN1, MYO7B, PPL, MYH6, SYCP2, CHD8, MYO5A, MYO1F, CENTD3, SPTB, DDX21, ROCK1, DNAH7, STAG3, CDK5RAP2, PLEKHH1, FGD2, LYST, SYNE2, ALS2, CEP250, DMD, MST1R, SYCP1, DNAH5, PB1, MYH2, DNAH8, MYO1D, TEP1, SGCA, UBTF, MYH4, MYH8, UTRN, BUB1B, KIF1A, MYO6, MYO1A, NOL6, MYO1C,
GO:0005859~muscle myosin complex	10	1.84%	9.68E-09	MYBPC3, MYH3, MYH11, MYBPC1, MYH1, MYH7, MYH4, MYH2, MYH8, MYH6,
GO:0005583~fibrillar collagen	8	1.47%	9.76E-09	COL3A1, COL1A2, COL5A2, COL2A1, TNXB, COL1A1, COL5A1, COL11A2,
GO:0016460~myosin II complex	10	1.84%	1.48E-08	MYBPC3, MYH3, MYH11, MYBPC1, MYH1, MYH7, MYH4, MYH2, MYH8, MYH6,
GO:0030016~myofibril	13	2.39%	6.28E-08	MYH3, MYH11, MYH7, NEB, MYH2, MYH6, TTN, MYBPC3, MYBPC1, MYH1, DMD, MYH4, MYH8,
GO:0005891~voltage-gated calcium channel complex	9	1.66%	1.31E-07	CACNA1B, CACNA2D1, CACNA1S, CACNA1C, CACNB2, CACNA1A, CACNA1E, CACNA1F, CACNA1D,
GO:0030017~sarcomere	12	2.21%	1.76E-07	MYBPC3, MYH3, MYH11, MYBPC1, MYH1, MYH7, NEB, MYH4, MYH2, MYH8, TTN, MYH6,
GO:0044449~contractile fiber part	13	2.39%	1.98E-07	MYH3, MYH11, MYH7, NEB, MYH2, MYH6, TTN, MYBPC3, MYBPC1, MYH1, DMD, MYH4, MYH8,
GO:0043292~contractile fiber	13	2.39%	4.68E-07	MYH3, MYH11, MYH7, NEB, MYH2, MYH6, TTN, MYBPC3, MYBPC1, MYH1, DMD, MYH4, MYH8,

Term	Count	%	P-Value	Genes
GO:0044459~plasma membrane part	94	17.31%	1.51E-06	COL17A1, MUC4, DCBLD2, EMR1, NAALADL1, PPL, PTPRE, BAI1, SLC12A2, TNS1, INADL, MYO7A, ABCC2, ENPEP, SLC15A2, NOTCH2, CACNA2D1, PTPRO, TEK, NPHS1, PTPRJ, CACNB2, FLJ31438, CTTNBP2, CLCA1, SYTL2, NOTCH4, ATP1A4, CACNA1S, ROS1, PTK2, TNR, KTN1, PTPRN2, CACNA1B, LRP1, PCLKC, SLMAP, DMD, CRHR2, MST1R, CLTC, CACNA1F, ATP6V0A4, ATP1A2, SLC15A1, RYR3, TRPM1, SLC4A3, PTPRG, ROBO2, STAB2, ATP1A3, DUOX2, ARHGAP17, SGCA, PTPRM, CNTN2, PTPRC, CACNA1A, MYH9, UTRN, PTPRN, HHIP, SORL1, TIE1, RYR2, PTPRD, PTPRS, MYO6, UNC13B, TMEM16G, MYO1A, NPHP1, PTPRA, RYR1, CGN, MYO1C, CACNA1E, DST, ENG, ATP12A, PTPRZ1, NEO1, SLC12A3, SHANK1, SLC12A4, CNGB1, CACNA1C, PTPRB, PTK7, CDH5, NOTCH3, RNF31, CACNA1D,
GO:0005604~basement membrane	12	2.21%	6.07E-06	COL4A3, HSPG2, COL4A5, SGCA, COL4A2, NID1, DMD, USH2A, COL4A4, DST, COL4A1, COL7A1,
GO:0005587~collagen type IV	5	0.92%	9.10E-06	COL4A3, COL4A5, COL4A2, COL4A4, COL4A1,
GO:0030935~sheet-forming collagen	5	0.92%	2.07E-05	COL4A3, COL4A5, COL4A2, COL4A4, COL4A1,
GO:0030934~anchoring collagen	5	0.92%	7.14E-05	COL9A2, COL12A1, COL16A1, COL9A1, COL7A1,
GO:0044421~extracellular region part	44	8.10%	7.24E-05	HSPG2, COL4A3, COL17A1, MUC4, CHL1, ADAMTS12, USH2A, SFTPB, COL9A1, COL7A1, TECTA, COL1A2, SGCA, COL2A1, SLIT3, THBS1, CLCA1, COL9A2, COL3A1, COL20A1, PAPPA, MUC2, COL4A2, TNXB, COMP, COL4A4, TNR, DST, PTPRZ1, COL5A1, COL4A1, COL16A1, VWF, THBS2, COL4A5, COL5A2, NID1, DMD, COL12A1, COL1A1, COL11A2, THBS3, SLIT1, ADAMTS10,
GO:0045177~apical part of cell	12	2.21%	1.01E-04	PCLKC, DUOX2, MYO1A, CHL1, NAALADL1, NOS3, CTTNBP2, MYO7A, INADL, ATP6V0A4, ABCC2, ENPEP,
GO:0045178~basal part of cell	6	1.10%	1.12E-04	COL17A1, MYO1A, MARK2, MYO1C, DST, CTTNBP2,
GO:0044463~cell projection part	9	1.66%	1.47E-04	DNAH1, CDH23, DNAH5, DNAH8, ROBO2, ALS2, DNAH7, ATP6V0A4, DNAH3,
GO:0000793~condensed chromosome	9	1.66%	2.15E-04	BUB1B, STAG3, SYCP2, CENPE, BRCA1, PAM, NOL6, TTN, SYCP1,
GO:0015630~microtubule cytoskeleton	26	4.79%	2.42E-04	ALMS1, DNAH5, PCNT, DNAH8, PTPN20A, DNAH3, CENPE, BRCA1, MARK2, DNAH7, RSN, BUB1B, KIF1A, DNAH1, CDK5RAP2, ODF2, LYST, ALS2, CEP250, CDH23, KIF13B, C20orf23, NIN, DNHD2, DNAH10, BUB1,
GO:0016324~apical plasma membrane	10	1.84%	3.16E-04	PCLKC, DUOX2, MYO1A, NAALADL1, CTTNBP2, MYO7A, INADL, ATP6V0A4, ABCC2, ENPEP,
GO:0005887~integral to plasma membrane	57	10.50%	3.69E-04	COL17A1, MUC4, DCBLD2, EMR1, PTPRE, BAI1, SLC12A2, ENPEP, ABCC2, NOTCH2, SLC15A2, PTPRO, NPHS1, TEK, PTPRJ, CACNB2, CLCA1, NOTCH4, ATP1A4, ROS1, KTN1, PTPRN2, LRP1, SLMAP, MST1R, CRHR2, SLC15A1, ATP1A2, RYR3, TRPM1, SLC4A3, PTPRG, STAB2, ATP1A3, PTPRM, CNTN2, PTPRC, MYH9, PTPRN, HHIP, TIE1, SORL1, RYR2, PTPRD, PTPRS, RYR1, PTPRA, ATP12A, PTPRZ1, ENG, NEO1, SLC12A3, SLC12A4, CNGB1, PTPRB, PTK7, NOTCH3, CACNA1D,
GO:0005593~FACIT collagen	4	0.74%	4.23E-04	COL9A2, COL12A1, COL16A1, COL9A1,

Term	Count	%	P-Value	Genes
GO:0031226~intrinsic to plasma membrane	57	10.50%	5.13E-04	COL17A1, MUC4, DCBLD2, EMR1, PTPRE, BAI1, SLC12A2, ENPEP, ABCC2, NOTCH2, SLC15A2, PTPRO, NPHS1, TEK, PTPRJ, CACNB2, CLCA1, NOTCH4, ATP1A4, ROS1, KTN1, PTPRN2, LRP1, SLMAP, MST1R, CRHR2, SLC15A1, ATP1A2, RYR3, TRPM1, SLC4A3, PTPRG, STAB2, ATP1A3, PTPRM, CNTN2, PTPRC, MYH9, PTPRN, HHIP, TIE1, SORL1, RYR2, PTPRD, PTPRS, RYR1, PTPRA, ATP12A, PTPRZ1, ENG, NEO1, SLC12A3, SLC12A4, CNGB1, PTPRB, PTK7, NOTCH3, CACNA1D,
GO:0030286~dynein complex	7	1.29%	5.50E-04	DNAH1, DNAH5, DNAH8, DNHD2, DNAH10, DNAH7, DNAH3,
GO:0005858~axonemal dynein	5	0.92%	6.75E-04	DNAH1, DNAH5, DNAH8, DNAH7, DNAH3,
GO:0016461~unconventional myosin complex	4	0.74%	7.25E-04	MYO6, MYO1F, MYO1C, MYO9A,
GO:0044447~axoneme part	5	0.92%	8.81E-04	DNAH1, DNAH5, DNAH8, DNAH7, DNAH3,
GO:0005886~plasma membrane	122	22.47%	1.12E-03	COL17A1, DCBLD2, SLC12A1, NAALADL1, SLC12A2, BAI1, TNS1, MYO7A, ABCC2, CACNA2D1, TECTA, PTPRO, TEK, MARK2, FLJ31438, DGKK, TRPM7, SYTL2, PLCE1, IL31RA, CACNA1S, PTK2, KTN1, PTPRN2, CACNA1B, LRP1, CDH23, ABCC4, SLMAP, NOTCH1, ENO2, CACNA1F, ATP6V0A4, RYR3, SLC4A3, PTPRG, CARD14, C11orf11, ARHGAP17, CNTN2, CACNA1A, MYH9, SORL1, SLC4A10, PTPRA, CGN, CACNA1E, DST, PTPRZ1, SLC12A4, CNGB1, CACNA1C, PTK7, CDH5, ACSL6, MIB1, RNF31, NOTCH3, MUC4, EMR1, PTPRE, PPL, TIAM1, INADL, ENPEP, SLC15A2, NOTCH2, NPHS1, PTPRJ, CACNB2, CENTD3, BAI2, CTTNBP2, XPNPEP2, CLCA1, NOTCH4, ATP1A4, ROS1, TNR, PCLKC, DMD, MST1R, CRHR2, CLTC, SLC15A1, ASAH2, ATP1A2, TRPM1, ZAN, ROBO2, STAB2, ATP1A3, DUOX2, SIGLEC1, PTPRM, SGCA, PTPRC, CNTN3, PTPRN, UTRN, HHIP, VAV2, TIE1, RYR2, PTPRD, PTPRS, MYO6, TMEM16G, UNC13B, MYO1A, NPHP1, RYR1, BAI3, MYO1C, ATP12A, ENG, NEO1, SHANK1
GO:0009925~basal plasma membrane	5	0.92%	1.13E-03	COL17A1, MYO1A, MYO1C, DST, CTTNBP2,
GO:0005890~sodium:potassium-exchanging ATPase complex	4	0.74%	1.14E-03	ATP1A3, ATP1A4, ROS1, ATP1A2,
GO:0005929~cilium	7	1.29%	1.59E-03	BBS5, ALMS1, CDH23, ODF2, CEP250, MYO7A, DNAH7,
GO:0005938~cell cortex	8	1.47%	1.79E-03	SPTA1, MYH10, MYO6, MYO1A, NOS2A, MARK2, MYH9, SPTB,
GO:0001726~ruffle	7	1.29%	2.00E-03	MYO6, MYO5A, FGD2, CENTD3, MYH9, ALS2, TRPM7,
GO:0031252~leading edge	9	1.66%	2.05E-03	MYO6, MYO5A, CDC42BPG, FGD2, CENTD3, MYH9, ROBO2, ALS2, TRPM7,
GO:0005624~membrane fraction	36	6.63%	2.56E-03	UACA, SPTA1, RYR3, TRPM1, SLC12A1, SLC4A3, SLC12A2, NOS1, ULK1, CORO7, SLC15A2, SPTB, CTTNBP2, BIRC6, DPP10, SYTL2, PLCE1, UTRN, MCTP2, CACNA1S, ROS1, ALS2, KTN1, SHANK1, SLC12A3, SLC12A4, LRP1, ZFP106, CNGB1, ABCC4, PTPRB, CDH5, DMD, ACSL6, SLC15A1, ATP1A2,

Term	Count	%	P-Value	Genes
GO:0005875~microtubule associated complex	11	2.03%	2.60E-03	KIF1A, DNAH1, DNAH5, CENPE, KIF13B, C20orf23, DNAH8, DNHD2, DNAH10, DNAH7, DNAH3,
GO:0005930~axoneme	5	0.92%	3.09E-03	DNAH1, DNAH5, DNAH8, DNAH7, DNAH3,
GO:0030055~cell-matrix junction	6	1.10%	3.25E-03	COL17A1, PTK2, PTPRC, DST, CTTNBP2, TNS1,
GO:0043234~protein complex	81	14.92%	3.80E-03	UACA, PPARGC1B, BAT5, MYO7B, XPO1, MYH7, MYH6, MYO7A, CACNA2D1, MYO5A, MYH1, MYO1F, CACNB2, HDAC10, MYH7B, SPTB, DNAH7, MYO9A, ATP1A4, DNAH1, CACNA1S, ROS1, ALS2, MYO10, SMARCC2, CACNA1B, MYBPC1, SLMAP, KIF13B, DMD, IPO11, DNAH10, ENO2, EP300, CACNA1F, ATP6V0A4, ATP1A2, SNAPC4, DNAH5, DNAH8, MYH2, RET, MYO1D, DIP13B, NUP133, DNAH3, MYBPC3, ATP1A3, MYH10, CENPE, MYO5B, BRCA1, SGCA, MYH4, MYH8, CACNA1A, MYH9, ARID1B, KIDINS220, RSN, BUB1B, MYH3, KIF1A, MYH11, MYO6, MYO1E, MYO1A, CGN, MYO1C, CACNA1E, ATP12A, CNGB1, CACNA1C, RANBP17, NFKB2, C20orf23,
GO:0005584~collagen type I	3	0.55%	4.62E-03	COL1A2, COL2A1, COL1A1,
GO:0005903~brush border	5	0.92%	5.00E-03	MYO1A, MYO1C, ATP6V0A4, PRSS7, ENPEP,
GO:0044448~cell cortex part	6	1.10%	7.07E-03	SPTA1, MYO1A, NOS2A, MARK2, MYH9, SPTB,
GO:0043005~neuron projection	9	1.66%	9.37E-03	SHANK1, MYO5A, OPA1, CHL1, CNTN2, ROBO2, ALS2, NF1, ULK1,
GO:0001725~stress fiber	4	0.74%	9.60E-03	MYH10, MST1R, MYH9, MYO1C,
GO:0032432~actin filament bundle	4	0.74%	9.60E-03	MYH10, MST1R, MYH9, MYO1C,
GO:0016323~basolateral plasma	8	1.47%	1.31E-02	COL17A1, MYO1A, PTK2, PTPRC, MYO1C, DST, CTTNBP2, TNS1,
GO:0000794~condensed nuclear chromosome	5	0.92%	1.36E-02	STAG3, SYCP2, NOL6, TTN, SYCP1,
GO:0005694~chromosome	20	3.68%	1.38E-02	BUB1B, STAG3, RIF1, PAM, PB1, NOL6, ODF2, TTN, SMARCC2, SYCP2, TEP1, CHD8, CENPE, BRCA1, SP100, RNF20, BUB1, RSN, ATRX, SYCP1,

Term	Count	%	P-Value	Genes
GO:0005622~intracellular	321	59.12%	1.60E-02	COL4A3, ALMS1, PASD1, BAT5, XPO1, NEB, UGCGL2, TNS1, PAPPA2, MPP4, MYH1, COL1A2, DCLRE1C, COL2A1, MARK2, FRMD4A, DIP2B, MYO9A, PLCE1, RIF1, WDR75, XPNPEP1, NEBL, KTN1, PARN, BMX, MYO10, FLJ20433, HERC6, KIF13B, OGDHL, IPO11, RNF20, NIN, ARFGEF2, STAT6, BBS5, SMARCAD1, TCOF1, VAV1, RET, PTPN20A, USH2A, DIP13B, CARD14, DPP8, DNAH3, OSBPL1A, CD2BP2, MYH10, FMNL1, VPS13A, ARHGAP17, CENPE, TARBP1, MYH9, SYNE1, RSN, PRKDC, CASKIN1, MYH3, MTR, MYH11, MYO1E, OBSCN, PTPN4, CC2D1A, NFKBIL2, KHDRBS2, DST, ZNF291, COL4A5, ACOT12, CACNA1C, POLQ, WRN, GON4L, RNF31, STAG1, NOTCH3, SFRS15, TCF4, SPTA1, PPARGC1B, PTPRE, RPS6KA2, PPL, TIAM1, TEX14, CTCFL, COL9A1, HACE1, CHD8, MYO1F, ALDH8A1, CLEC7A, DNAH7, STAG3, COL20A1, CDK5RAP2, PLEKHH1, EP400, KIAA1524, LYST, CASZ1, ALS2, UST, KIAA1604, COG7, VWF, COL5A2, DMD, MST1R, COL12A1, EP300, CLTC, FANCM, COL11A2, ATP1A2, ASAH2, SYCP1, MLL3, IFT122, DNAH5, PB1, MYH2, ANKHD1, NUP133, YEATS2, PLEKHA4, SGCA, UBTF, SOS2, LRRK2, UTRN, KIF1A, UNC13B, TMEM16G, PLEKHG5
GO:0044464~cell part	436	80.29%	1.86E-02	BAT5, XPO1, NEB, SLC12A2, BAI1, TNS1, CACNA2D1, MYH1, MARK2, DIP2B, TRPM7, MYO9A, C10orf76, IL31RA, PLCE1, RIF1, XPNPEP1, NEBL, KTN1, FLJ20433, C19orf15, HERC6, OGDHL, IPO11, RNF20, CACNA1F, STAT6, SMARCAD1, VAV1, RET, PTPN20A, CARD14, DPP8, CD2BP2, GRAMD1B, VPS13A, FMNL1, ARHGAP17, SYNE1, RSN, PRKDC, MYH3, CASKIN1, MTR, MYO1E, SIDT1, NFKBIL2, KHDRBS2, PTPRZ1, ACOT12, CACNA1C, PTK7, POLQ, GON4L, RNF31, SDK2, PTPRE, TEX14, INADL, SLC12A5, CHD8, ALDH8A1, XPNPEP2, DNAH7, CLCA1, CDK5RAP2, KIAA1524, CASZ1, UST, DSCAML1, DMD, COL12A1, MST1R, CRHR2, CLTC, EP300, COL11A2, SLC15A1, IFT122, PB1, ROBO2, ANKHD1, STAB2, PLEKHA4, SOS2, LRRK2, PTPRN, DCHS2, TMEM16G, PLEKHG5, RYR1, TNXB, ATP12A, SLC12A3, SHANK1, PTPRB, ATM, DNHD2, COL17A1, ZRANB3, MAMDC4, MYH7, PAM, SLC12A1, RALGPS2, NAALAD2, MYO7A, TECTA, PTPRO, ACMSD, SFMBT2, FLJ31438, DGKK, SYTL2, COL9A2, MCTP2, DNAH1, GRAMD1A, STAG2, AKT3, USP37, ZNF236, TDRD1, COL5A1, LOC133308, MYBPC1, NOTCH1, YLPM1, DNAH10, ENO2, CACHD1, ZNF185, SLC4A3, NFKB1,

Term	Count	%	P-Value	Genes
GO:0005623~cell	436	80.29%	1.88E-02	BAT5, XPO1, NEB, SLC12A2, BAI1, TNS1, CACNA2D1, MYH1, MARK2, DIP2B, TRPM7, MYO9A, C10orf76, IL31RA, PLCE1, RIF1, XPNPEP1, NEBL, KTN1, FLJ20433, C19orf15, HERC6, OGDHL, IPO11, RNF20, CACNA1F, STAT6, SMARCAD1, VAV1, RET, PTPN20A, CARD14, DPP8, CD2BP2, GRAMD1B, VPS13A, FMNL1, ARHGAP17, SYNE1, RSN, PRKDC, MYH3, CASKIN1, MTR, MYO1E, SIDT1, NFKBIL2, KHDRBS2, PTPRZ1, ACOT12, CACNA1C, PTK7, POLQ, GON4L, RNF31, SDK2, PTPRE, TEX14, INADL, SLC12A5, CHD8, ALDH8A1, XPNPEP2, DNAH7, CLCA1, CDK5RAP2, KIAA1524, CASZ1, UST, DSCAML1, DMD, COL12A1, MST1R, CRHR2, CLTC, EP300, COL11A2, SLC15A1, IFT122, PB1, ROBO2, ANKHD1, STAB2, PLEKHA4, SOS2, LRRK2, PTPRN, DCHS2, TMEM16G, PLEKHG5, RYR1, TNXB, ATP12A, SLC12A3, SHANK1, PTPRB, ATM, DNHD2, COL17A1, ZRANB3, MAMDC4, MYH7, PAM, SLC12A1, RALGPS2, NAALAD2, MYO7A, TECTA, PTPRO, ACMSD, SFMBT2, FLJ31438, DGKK, SYTL2, COL9A2, MCTP2, DNAH1, GRAMD1A, STAG2, AKT3, USP37, ZNF236, TDRD1, COL5A1, LOC133308, MYBPC1, NOTCH1, YLPM1, DNAH10, ENO2, CACHD1, ZNF185, SLC4A3, NFKB1,
GO:0005815~microtubule organizing	10	1.84%	1.90E-02	ALMS1, CDH23, CDK5RAP2, PCNT, BRCA1, ODF2, ALS2, NIN, CEP250, BUB1,
GO:0005737~cytoplasm	198	36.46%	2.32E-02	COL4A3, ALMS1, BAT5, XPO1, NEB, UGCGL2, TNS1, MPP4, MYH1, COL1A2, COL2A1, MARK2, FRMD4A, PLCE1, XPNPEP1, KTN1, PARN, BMX, HERC6, KIF13B, OGDHL, IPO11, NIN, ARFGEF2, STAT6, PTPN20A, USH2A, DIP13B, CARD14, DPP8, CD2BP2, MYH10, FMNL1, ARHGAP17, MYH9, SYNE1, RSN, MYH3, CASKIN1, MTR, MYH11, OBSCN, PTPN4, CC2D1A, NFKBIL2, DST, COL4A5, ACOT12, RNF31, SPTA1, PTPRE, PPL, TEX14, COL9A1, HACE1, CLEC7A, COL20A1, CDK5RAP2, KIAA1524, LYST, UST, ALS2, VWF, COG7, COL5A2, DMD, COL12A1, CLTC, COL11A2, ASAH2, ATP1A2, IFT122, MYH2, ANKHD1, PLEKHA4, SGCA, LRRK2, UTRN, TMEM16G, UNC13B, PLEKHG5, RYR1, MYO1C, SHANK1, PPP1R12A, ACSS1, ATM, NOS3, COL17A1, CAD, PCNT, MYH7, PAM, MYO7A, SFTPB, ACMSD, FLJ31438, DGKK, SYTL2, UGCGL1, COL9A2, ACACA, CDC42BPG, AKT3, TDRD1, UNC13D, TTN, COL5A1, LRP1, MYBPC1, CDH23, ABCC4, SLMAP, NOTCH1, ENO2, ATP6V0A4, NFKB1, NF1, ULK1, COL7A1, MYBPC3, PTPN13, BRCA1, HECW2, CACNA1A, XDH, NOS2A, COL4A2, ODF2, PACS1, MTHFD1, COL4A4, STAT5A, ATP8A1, CCAR1, RANBP17, PASK, NFKB2, ACSL6,
GO:0031941~filamentous actin	3	0.55%	2.52E-02	MYO6, MYO1A, MYO1C,
GO:0000267~cell fraction	39	7.18%	2.69E-02	UACA, SPTA1, RYR3, TRPM1, SLC12A1, PTPRE, SLC4A3, SLC12A2, NOS1, ULK1, CORO7, SLC15A2, TEP1, SPTB, CTTNBP2, BIRC6, DPP10, SYTL2, PLCE1, UTRN, MCTP2, CACNA1S, ROS1, ALS2, KTN1, SHANK1, SLC12A3, LRP1, SLC12A4, ZFP106, CNGB1, ABCC4, PTPRB, CDH5, DMD, ACSL6, NOS3, SLC15A1, ATP1A2,
GO:0000228~nuclear chromosome	8	1.47%	2.70E-02	STAG3, SYCP2, PAM, PB1, NOL6, TTN, SYCP1, ATRX,
GO:0030054~cell junction	19	3.50%	3.34E-02	COL17A1, UNC13B, TMEM16G, NPHP1, PPL, PTK2, BAI1, CGN, TNS1, DST, INADL, ABCC2, SHANK1, PCLKC, ARHGAP17, CDH5, PTPRC, CTTNBP2, UTRN,

Term	Count	%	P-Value	Genes
GO:0030425~dendrite	5	0.92%	3.53E-02	SHANK1, OPA1, CHL1, ALS2, NF1,
GO:0044446~intracellular organelle part	116	21.36%	3.89E-02	ALMS1, XPO1, PCNT, MYH7, NEB, PAM, UGCGL2, DHX35, MYO7A, MYH1, MYH7B, MYO9A, PLCE1, UGCGL1, DNAH1, RIF1, KTN1, MYO10, TTN, SMARCC2, MYBPC1, ABCC4, KIF13B, OGDHL, YLPM1, IPO11, NIN, ARFGEF2, DNAH10, ATP6V0A4, SMARCAD1, TCOF1, PTPN20A, DIP13B, ULK1, DNAH3, MYBPC3, MYH10, DDX54, CENPE, BRCA1, MYO5B, MYH9, SYNE1, RSN, MYH3, MYH11, MYO1E, PRPF8, ODF2, CGN, PACS1, CACNA1C, RANBP17, SP100, POLQ, WRN, ACSL6, BUB1, TCF4, UACA, SPTA1, PPARGC1B, MYO7B, OGDH, MYH6, KIAA1967, SYCP2, CHD8, MYO5A, MYO1F, HDAC10, SPTB, DDX21, CTTNBP2, ROCK1, DNAH7, NCBP1, STAG3, CDK5RAP2, SYNE2, UST, ALS2, CEP250, KIAA1604, MST1R, CLTC, EP300, SYCP1, SNAPC4, DNAH5, ATP2A1, PCCA, PB1, DNAH8, MYH2, MYO1D, NUP133, TEP1, OPA1, UBTF, MYH4, MYH8, ARID1B, KIDINS220, BUB1B, KIF1A, MYO6, GPAM, MYO1A,
GO:0005576~extracellular region	50	9.21%	4.19E-02	COL4A3, UACA, COL17A1, MUC4, CHL1, TG, SFTPB, COL9A1, ODZ1, TECTA, COL1A2, COL2A1, SLIT3, THBS1, CLCA1, COL9A2, COL20A1, COMP, TNR, COL4A1, COL5A1, VWF, COL5A2, DMD, COL12A1, NELL2, THBS3, PZP, COL11A2, ADAMTS10, SLIT1, HSPG2, MUC6, USH2A, ADAMTS12, COL7A1, SGCA, COL3A1, PAPPA, MUC2, COL4A2, TNXB, COL4A4, DST, PTPRZ1, COL16A1, COL4A5, THBS2, NID1, COL1A1,
GO:0044422~organelle part	116	21.36%	4.21E-02	ALMS1, XPO1, PCNT, MYH7, NEB, PAM, UGCGL2, DHX35, MYO7A, MYH1, MYH7B, MYO9A, PLCE1, UGCGL1, DNAH1, RIF1, KTN1, MYO10, TTN, SMARCC2, MYBPC1, ABCC4, KIF13B, OGDHL, YLPM1, IPO11, NIN, ARFGEF2, DNAH10, ATP6V0A4, SMARCAD1, TCOF1, PTPN20A, DIP13B, ULK1, DNAH3, MYBPC3, MYH10, DDX54, CENPE, BRCA1, MYO5B, MYH9, SYNE1, RSN, MYH3, MYH11, MYO1E, PRPF8, ODF2, CGN, PACS1, CACNA1C, RANBP17, SP100, POLQ, WRN, ACSL6, BUB1, TCF4, UACA, SPTA1, PPARGC1B, MYO7B, OGDH, MYH6, KIAA1967, SYCP2, CHD8, MYO5A, MYO1F, HDAC10, SPTB, DDX21, CTTNBP2, ROCK1, DNAH7, NCBP1, STAG3, CDK5RAP2, SYNE2, UST, ALS2, CEP250, KIAA1604, MST1R, CLTC, EP300, SYCP1, SNAPC4, DNAH5, ATP2A1, PCCA, PB1, DNAH8, MYH2, MYO1D, NUP133, TEP1, OPA1, UBTF, MYH4, MYH8, ARID1B, KIDINS220, BUB1B, KIF1A, MYO6, GPAM, MYO1A,

Term	Count	%	P-Value	Genes
GO:0044424~intracellular part	299	55.06%	4.56E-02	COL4A3, ALMS1, PASD1, BAT5, XPO1, NEB, UGCGL2, TNS1, MPP4, MYH1, COL1A2, DCLRE1C, COL2A1, MARK2, FRMD4A, DIP2B, MYO9A, PLCE1, RIF1, WDR75, XPNPEP1, NEBL, KTN1, PARN, BMX, MYO10, HERC6, KIF13B, OGDHL, IPO11, RNF20, NIN, ARFGEF2, STAT6, SMARCAD1, TCOF1, VAV1, RET, PTPN20A, USH2A, DIP13B, CARD14, DPP8, DNAH3, OSBPL1A, CD2BP2, MYH10, FMNL1, ARHGAP17, CENPE, TARBP1, MYH9, SYNE1, RSN, PRKDC, CASKIN1, MYH3, MTR, MYH11, MYO1E, OBSCN, PTPN4, CC2D1A, NFKBIL2, KHDRBS2, DST, COL4A5, ACOT12, CACNA1C, POLQ, WRN, GON4L, RNF31, STAG1, NOTCH3, SFRS15, TCF4, SPTA1, PPARGC1B, PTPRE, RPS6KA2, PPL, TEX14, CTCFL, COL9A1, CHD8, HACE1, MYO1F, CLEC7A, DNAH7, STAG3, COL20A1, CDK5RAP2, EP400, PLEKHH1, KIAA1524, CASZ1, LYST, UST, ALS2, KIAA1604, VWF, COG7, COL5A2, DMD, MST1R, COL12A1, EP300, CLTC, FANCM, COL11A2, ATP1A2, ASAH2, SYCP1, MLL3, IFT122, DNAH5, PB1, MYH2, ANKHD1, NUP133, YEATS2, PLEKHA4, SGCA, UBTF, LRRK2, UTRN, KIF1A, UNC13B, TMEM16G, PLEKHG5, RYR1, MYO1C, WHSC1L1, SHANK1, PPP1R12A, ACSS1,
GO:0005925~focal adhesion	4	0.74%	4.78E-02	PTK2, PTPRC, CTTNBP2, TNS1,
GO:0030863~cortical cytoskeleton	4	0.74%	4.78E-02	SPTA1, MYO1A, NOS2A, SPTB,
GO:0009986~cell surface	10	1.84%	5.46E-02	HHIP, NOTCH4, DCBLD2, EMR1, BAI3, ROBO2, TNR, STAB2, DSCAML1, NOTCH2,
GO:0044428~nuclear part	40	7.37%	5.58E-02	UACA, PPARGC1B, SNAPC4, XPO1, SMARCAD1, TCOF1, PAM, PB1, DIP13B, DHX35, NUP133, SYCP2, TEP1, DDX54, BRCA1, UBTF, HDAC10, DDX21, ARID1B, SYNE1, NCBP1, BUB1B, STAG3, MYO6, PRPF8, NOL6, SYNE2, TTN, SMARCC2, KIAA1604, RANBP17, SP100, POLQ, WRN, YLPM1, IPO11, EP300, TCF4, ATRX, SYCP1,
GO:0000795~synaptonemal complex	3	0.55%	5.82E-02	STAG3, SYCP2, SYCP1,
GO:0000776~kinetochore	4	0.74%	6.10E-02	BUB1B, CENPE, BUB1, RSN,
GO:0005924~cell-substrate adherens junction	4	0.74%	6.58E-02	PTK2, PTPRC, CTTNBP2, TNS1,
GO:0032991~macromolecular complex	87	16.02%	6.75E-02	UACA, PPARGC1B, BAT5, MYO7B, XPO1, MYH7, DHX35, MYH6, MYO7A, CACNA2D1, MYO5A, MYH1, MYO1F, CACNB2, HDAC10, MYH7B, SPTB, DNAH7, MYO9A, ATP1A4, DNAH1, CACNA1S, ROS1, ALS2, MYO10, SMARCC2, KIAA1604, CACNA1B, MYBPC1, SLMAP, KIF13B, DMD, IPO11, DNAH10, ENO2, EP300, CACNA1F, ATP6V0A4, ATP1A2, SNAPC4, DNAH5, DNAH8, MYH2, RET, MYO1D, DIP13B, NUP133, DNAH3, MYBPC3, ATP1A3, TEP1, MYH10, CENPE, MYO5B, BRCA1, SGCA, MYH4, MYH8, CACNA1A, MYH9, ARID1B, KIDINS220, RSN, BUB1B, MYH3, KIF1A, MYH11, MYO6, MYO1E, MYO1A, PRPF8, NOL6, CGN, MYO1C, CACNA1E, ATP12A, CNGB1, CACNA1C, CCDC40, RANBP17, NFKB2, C20orf23, CAMK2G, DNHD2, BUB1, TCF4, CACNA1D,
GO:0044427~chromosomal part	15	2.76%	8.08E-02	BUB1B, STAG3, RIF1, PAM, ODF2, SMARCC2, SYCP2, TEP1, CHD8, CENPE, SP100, BUB1, RSN, ATRX, SYCP1,

Term	Count	%	P-Value	Genes
GO:0030056~hemidesmosome	2	0.37%	8.26E-02	COL17A1, DST,
GO:0005594~collagen type IX	2	0.37%	8.26E-02	COL9A2, COL9A1,
GO:0005588~collagen type V	2	0.37%	8.26E-02	COL5A2, COL5A1,
GO:0000940~outer kinetochore of condensed chromosome	2	0.37%	8.26E-02	BUB1B, CENPE,
GO:0032154~cleavage furrow	2	0.37%	8.26E-02	MYH10, MYH9,
GO:0005902~microvillus	3	0.55%	9.97E-02	CDH23, MYO1A, MYO1C,
GO:0030864~cortical actin cytoskeleton	3	0.55%	9.97E-02	SPTA1, MYO1A, SPTB,
