# Supplementary material for: Small Molecule Amiloride Modulates Oncogenic RNA Alternative Splicing to Devitalize Human Cancer Cells
Source: PLoS One. 2011 Jun 9;6(6):e18643. doi: 10.1371/journal.pone.0018643 (PMC3111415; doi:10.1371/journal.pone.0018643)
Supplement: Table S3 — The splicing factor-binding exonic and intronic elements of genes showing altered alternative RNA splicing in amiloride-treated Huh-7 cells. (PDF) [file pone.0018643.s007.pdf]

Supplementary Table 3. The exonic and intronic elements of alterntive splicing regions.

Binding Factors	Gene numbers	Percentage	Gene_Exon
**Regulatory binding factors of exonic sequences in alternative splicing regions. (46 genes)**			
ASF/SF2	16	34.78%	DCLRE1C_Exon1,C10orf6_Exon20,GPAM_Exon20,CLEC7A_Exon1,FARP1_Exon28,MLLT6_Exon20,WDR35_Exon20,PTPN4_Exon20,CHL1_Exon28,LARP2_Exon20,BAI3_Exon20,HACE1_Exon20,SUSD1_Exon1,CXorf20_Exon10,DMD_Exon44,FLJ36180_Exon3,
SRp55	19	41.30%	MYH6_Exon6,MYH7_Exon6,MYH8_Exon6,MYH1_Exon6,MYH2_Exon6,ZNF24_Exon4,MYO5B_Exon20,CMYA3_Exon10,HHIP_Exon1,TSNARE1_Exon5,ODZ1_Exon28,ARHGEF10L_Exon20,FARP1_Exon28,FTS_Exon1,FLJ32310_Exon10,ATP2A1_Exon20,C17orf27_Exon20,MYLK_Exon20,BAI3_Exon20,
SC35	15	32.61%	DCLRE1C_Exon1,C10orf6_Exon20,GPAM_Exon20,CLEC7A_Exon1,FARP1_Exon28,MLLT6_Exon20,WDR35_Exon20,PTPN4_Exon20,CHL1_Exon28,LARP2_Exon20,BAI3_Exon20,HACE1_Exon20,SUSD1_Exon1,CXorf20_Exon10,DMD_Exon44,
CUG-BP*	8	17.39%	DCLRE1C_Exon1,C10orf6_Exon20,FARP1_Exon28,FANCM_Exon20,MYH8_Exon6,MLLT6_Exon20,FGD2_Exon1,NCBP1_Exon20,
PTB	3	6.52%	TTC7A_Exon20,FGD2_Exon1,FLJ36180_Exon3,
Others	22	47.83%	
**Regulatory binding factors of 5'-flanking intronic sequences in alternative splicing regions. (233 genes)**			
ASF/SF2	31	13.30%	CCDC46_Exon5,MYBPC1_Exon6,CENTD3_Exon1,RYR2_Exon61,ULK4_Exon20,INADL_Exon28,RYR2_Exon61,C10orf18_Exon20,C10orf6_Exon20,COL17A1_Exon28,LMO7_Exon4,MTHFD1_Exon20,MYH1_Exon20,MLLT6_Exon20,ABCA10_Exon28,NPC1_Exon24,PLEKHH2_Exon10,NEB_Exon20,SENP7_Exon20,SIDT1_Exon20,STAG1_Exon28,HERC6_Exon20,FLJ40243_Exon28,SLCO6A1_Exon10,C6orf107_Exon20,FGD2_Exon1,COL9A1_Exon28,DENND1A_Exon20,MYBPC2_Exon6,DNAH7_Exon61,KDR_Exon4,
CUG-BP	57	24.46%	CTTN_Exon5,GRAMD1B_Exon20,NELL2_Exon20,MYBPC1_Exon20,COG7_Exon10,MYH4_Exon20,MYH1_Exon20,MYH3_Exon20,ABCA8_Exon28,C19orf15_Exon20,RYR1_Exon61,EHBP1_Exon14,SEPT10_Exon5,COL3A1_Exon28,COL5A2_Exon28,TMC2_Exon10,ZPLD1_Exon10,PPARGC1B_Exon10,FGD2_Exon1,BAI3_Exon20,COL12A1_Exon28,MYB_Exon6,COL1A2_Exon28,STAG3_Exon28,PTPRN2_Exon20,VPS13A_Exon60,

Binding Factors	Gene numbers	Percentage	Gene_Exon
Nova-1	2	0.86%	CLEC7A_Exon1,PTPN13_Exon20,
PTB	7	3.00%	PTPN4_Exon20,ACSS1_Exon13,MYBPC1_Exon6,MYBPC2_Exon6,DNAH7_Exon61,KDR_Exon4,
SC35	26	11.16%	INADL_Exon28,RYR2_Exon61,C10orf18_Exon20,C10orf6_Exon20,COL17A1_Exon28,LMO7_Exon4,MTHFD1_Exon20,MYH1_Exon20,MLLT6_Exon20,ABCA10_Exon28,NPC1_Exon24,PLEKHH2_Exon10,NEB_Exon20,SENP7_Exon20,SIDT1_Exon20,STAG1_Exon28,HERC6_Exon20,FLJ40243_Exon28,SLCO6A1_Exon10,C6orf107_Exon20,FGD2_Exon1,COL9A1_Exon28,DENND1A_Exon20,COL6A3_Exon28,
SRp75	26	11.16%	INADL_Exon28,RYR2_Exon61,C10orf18_Exon20,C10orf6_Exon20,COL17A1_Exon28,LMO7_Exon4,MTHFD1_Exon20,MYH1_Exon20,MLLT6_Exon20,ABCA10_Exon28,NPC1_Exon24,PLEKHH2_Exon10,NEB_Exon20,SENP7_Exon20,SIDT1_Exon20,STAG1_Exon28,HERC6_Exon20,FLJ40243_Exon28,SLCO6A1_Exon10,C6orf107_Exon20,FGD2_Exon1,COL9A1_Exon28,DENND1A_Exon20,CCDC46_Exon5,
Others	141	60.52%	
**Regulatory binding factors of 3'-flanking intronic sequences in alternative splicing regions. (207 genes)**			
ASF/SF2	33	15.94%	C10orf6_Exon20,COL5A2_Exon28,MYO5B_Exon20,C20orf12_Exon20,MYT1_Exon20,MSH4_Exon20,LRP1_Exon4,DNAH10_Exon46,LMO7_Exon4,MYH7_Exon6,FTS_Exon1,SDK2_Exon20,C17orf27_Exon20,C19orf15_Exon20,BIRC6_Exon61,PLEKHH2_Exon28,EHBP1_Exon14,DPP10_Exon20,DRB1_Exon10,DNAH7_Exon61,PARD3B_Exon10,C20orf23_Exon20,KIAA1524_Exon20,STAG1_Exon28,KIAA1345_Exon20,PTPN13_Exon20,DNAJA5_Exon10,IL31RA_Exon10,COL19A1_Exon28,KIF13B_Exon14,WHSC1L1_Exon20,MYBL1_Exon6,C9orf52_Exon18,KIAA1797_Exon20,SRGAP1_Exon20,MCTP2_Exon20,CPNE4_Exon10,MYO5B_Exon20,C20orf12_Exon20,MYT1_Exon20,

Binding Factors	Gene numbers	Percentage	Gene_Exon
CUG-BP	47	22.71%	COL11A1_Exon28,MTR_Exon20,RNF31_Exon20,SYNE2_Exon107,HYDIN_Exon20,NF1_Exon20,NPC1_Exon24,LAMA3_Exon4,EMR1_Exon5,BIRC6_Exon61,SEPT10_Exon5,DPP10_Exon20,COL3A1_Exon28,PARD3B_Exon10,DCBLD2_Exon10,PLD1_Exon21,CPZ_Exon4,TLL1_Exon9,ODZ3_Exon20,FLJ36180_Exon3,DNAJA5_Exon10,SLCO6A1_Exon10,COL9A1_Exon28,COL12A1_Exon28,COL12A1_Exon10,ARID1B_Exon20,LPA_Exon4,WHSC1L1_Exon20,KIAA1797_Exon20,VPS13A_Exon60,MAP3K15_Exon4,LOC728343_Exon61,NXF2_Exon61,
Nova-1	1	0.48%	LAMA3_Exon4,
PTB	8	3.86%	COL17A1_Exon28,PTPRO_Exon20,MAP4K1_Exon28,LRRK2_Exon28,OTUD4_Exon20,SRGAP1_Exon20,MCTP2_Exon20,CPNE4_Exon10,
SC35	30	14.49%	ARHGEF10L_Exon20,MSH4_Exon20,LRP1_Exon4,DNAH10_Exon46,LMO7_Exon4,MYH7_Exon6,FTS_Exon1,SDK2_Exon20,C17orf27_Exon20,C19orf15_Exon20,BIRC6_Exon61,PLEKHH2_Exon28,EHBP1_Exon14,DPP10_Exon20,DRB1_Exon10,DNAH7_Exon61,PARD3B_Exon10,C20orf23_Exon20,KIAA1524_Exon20,STAG1_Exon28,KIAA1345_Exon20,PTPN13_Exon20,DNAJA5_Exon10,IL31RA_Exon10,COL19A1_Exon28,KIF13B_Exon14,WHSC1L1_Exon20,MYBL1_Exon6,C9orf52_Exon18,KIAA1797_Exon20,
SRp75	30	14.49%	MSH4_Exon20,LRP1_Exon4,DNAH10_Exon46,LMO7_Exon4,MYH7_Exon6,FTS_Exon1,SDK2_Exon20,C17orf27_Exon20,C19orf15_Exon20,BIRC6_Exon61,PLEKHH2_Exon28,EHBP1_Exon14,DPP10_Exon20,DRB1_Exon10,DNAH7_Exon61,PARD3B_Exon10,C20orf23_Exon20,KIAA1524_Exon20,STAG1_Exon28,KIAA1345_Exon20,PTPN13_Exon20,DNAJA5_Exon10,IL31RA_Exon10,COL19A1_Exon28,KIF13B_Exon14,WHSC1L1_Exon20,MYBL1_Exon6,C9orf52_Exon18,KIAA1797_Exon20,
Others	122	58.94%	
